# Biosynthesis of the Red Algal Diterpene Peyssonnosol in Bacteria

**DOI:** 10.1002/anie.202507752

**Published:** 2025-07-02

**Authors:** Zhiyong Yin, Zhehui Hu, Kexin Yang, Guihu Lu, Georges B. Tabekoueng, Pingping Wu, Qi Luo, Juan Xu, Bernd Goldfuss, Jeroen S. Dickschat, Guangkai Bian

**Affiliations:** ^1^ Kekulé‐Institute for Organic Chemistry and Biochemistry University of Bonn Gerhard‐Domagk‐Straße 1 53121 Bonn Germany; ^2^ Center of Materials Synthetic Biology Shenzhen Institute of Synthetic Biology Shenzhen Institutes of Advanced Technology Shenzhen 518055 P.R. China; ^3^ Guangdong Provincial Key Laboratory of Chinese Medicine Pharmaceutics School of Traditional Chinese Medicine Southern Medical University Guangzhou 510515 P.R. China; ^4^ National Key Laboratory for Germplasm Innovation & Utilization of Horticultural Crops College of Horticulture and Forestry Huazhong Agricultural University Wuhan 430070 P.R. China; ^5^ Department for Chemistry University of Cologne Greinstrasse 4 50939 Cologne Germany

**Keywords:** Biosynthesis, Cyclopropanes, Diterpenes, Enzyme mechanisms, Polycycles

## Abstract

Two diterpene synthases (DTSs) for peyssonnosol and peyssonnosol B were discovered in bacteria. Their enzyme mechanisms were investigated through isotopic labelling experiments, density functional theory (DFT) calculations and site‐directed mutagenesis, yielding biosynthetically related molecules that gave important insights into the terpene cyclisation cascade. In the present case, several mechanisms in line with the isotopic labelling experiments could be formulated. Only an extended experimental approach in combination with computational chemistry ultimately resulted in a refined mechanistic model.

## Graphical abstract

Two terpene synthases derived from *Anaerolineae bacteria* catalyse the biosynthesis of peyssonnosene‐type diterpenes from red algae. Site‐directed mutagenesis enabled a partial functional switch, resulting in the formation of additional byproducts. The intricate cyclisation mechanisms were investigated through isotopic labelling experiments and density functional theory calculations.

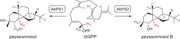

The fascinating structural diversity of terpenes is largely attributed to the action of terpene synthases (TSs) that can introduce a level of structural complexity into a natural product that is not seen with any other type of enzyme. Structurally terpenes constitute one of the most remarkable and diverse classes of natural products, and their potent bioactivity makes them an interesting subject of study. For instance, the plant derived sesquiterpenoid artemisinin^[^ ^1^ ^]^ and the diterpenoid polyanthellin A^[^ ^2^ ^]^ from soft corals are active against *Plasmodium falciparum*, and the diterpenoids taxol^[^ ^3^ ^]^ and ingenol mebutate^[^ ^4^ ^]^ show pronounced cytotoxicity. All these compounds are accessible through total synthesis,^[^ ^5^, ^6^, ^7^, ^8^ ^]^ and biotechnological approaches have been established for artemisinin^[^ ^9^ ^]^ and taxol^[^ ^10^ ^]^ production, requiring knowledge about the biosynthetic genes and enzymes including the TSs for parent hydrocarbon biosynthesis.^[^ ^11^, ^12^, ^13^, ^14^, ^15^ ^]^

Recently, the structurally remarkable peyssonnosides A (**1**) and B (**2**) have been discovered from a marine red alga attributed to the genus *Peyssonnelia* (Figure 1).^[^ ^16^ ^]^ In addition to their unique 6–3–6–5 tetracyclic skeleton with two adjacent quaternary centres embedded in a cyclopropane motif, these sulphated diterpene glycosides show a pronounced antifungal activity and are active against *Plasmodium berghei*. Peyssonnoside A and its aglycone peyssonnosol (**3**), a putative DTS product, have been accessed through total synthesis,^[^ ^17^, ^18^ ^]^ but a DTS for **3** is currently unknown. Here, we report on the identification and mechanistic characterisation of two DTSs for **3** and its regioisomer peyssonnosol B (**4**).

**Figure 1:**
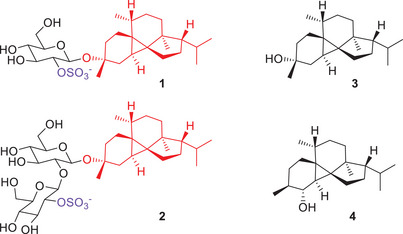
Structures of peyssonnosides A (**1**) and B (**2**), peyssonnosol (**3**) and peyssonnosol B (**4**).

During a functional screening of 312 bacterial TS homologs, two DTSs were discovered that are phylogenetically distant to other characterised bacterial enzymes (Figure S1). The closest characterised TSs with a sequence identity of ∼15% are cyclooctat‐9‐en‐7‐ol synthase (SmCotB2) from *Streptomyces melanosporofaciens* ^[^ ^19^ ^]^ and collinodiene synthase (SiCotB2) from *S. iakyrus*.^[^ ^20^ ^]^ The function of the first enzyme from *Anaerolineae bacterium* HKST‐UBA68 was investigated through heterologous overexpression of ERG20^F96C^ (geranylgeranyl pyrophosphate synthase, GGPPS)^[^ ^21^ ^]^ and the DTS in the engineered *Saccharomyces cerevisiae* YZL141 for an enhanced terpene precursor supply,^[^ ^22^ ^]^ allowing for the isolation of the diterpene alcohol peyssonnosol (**3**) (Figures 1, S2–S9, Table S2). Using the same approach, the product of the second DTS from *Anaerolineaceae bacterium* NAT117 was identified as peyssonnosol B (**4**) (Table S3, Figures S10–S17). These results identified the DTSs as *Anaerolineae bacterium* Peyssonnosol Synthase (AbPS1) and *Anaerolineaceae bacterium* Peyssonnosol B Synthase (AbPS2). The second enzyme is identical to PeyS that was recently reported to produce peyssonnosene,^[^ ^23^, ^24^ ^]^ a compound that we observed as a minor enzyme product. The difference regarding the main product observed in vivo may originate from different expression strains, plasmids or culture conditions.

The absolute configuration of **3** was assigned by comparison of the optical rotation ([α]_D_^25^ = –24.8, *c* 0.58, CH_2_Cl_2_) to data for the synthetic compound ([α]_D_^22^ = –31.2, *c* 0.3, CHCl_3_),^[^ ^18^ ^]^ revealing identical absolute configurations for bacterial and algal **3**. This was confirmed through anomalous dispersion (Cu‐Kα) X‐ray cyrstallography for both compounds **3** and **4** (Scheme 1, Figures S18 and S19, Tables S4 and S5).

**Scheme 1:**
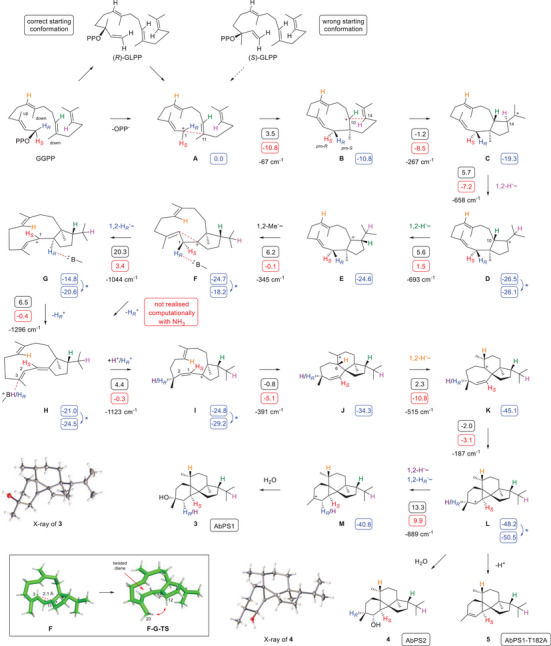
Cyclisation mechanism of AbPS1 and AbPS2 and their enzyme variants from GGPP to **3** – **5**. Blue boxes: computed relative energies to **A** (set to 0.0 kcal mol^−1^), black boxes: reaction barriers, red boxes: Gibbs free energies [in kcal mol^−1^, mPW1PW91/6–311 + G(d,p)//B97D3/6–31G(d,p), 298 K]. For the sequence **G**‐**H**‐**I,** NH_3_ was used as a surrogate base and the computed Gibbs energies were reduced by the energy of NH_3_ to allow for a direct comparison. Blue arrows and asterisks indicate minor conformational changes between the product of one computed step and the starting material for the next step. Imaginary frequencies of transition states are given in cm^−1^. Boxes next to compound numbers indicate the source enzyme for compound isolation. Large box: computed structures of intermediate **F** and the transition state **F‐G‐TS**.

The recombinant enzymes AbPS1 and AbPS2 (PeyS) were made accessible through heterologous expression in *Escherichia coli* BL21(DE3), purified (Figure S20) and incubated with geranyl (GPP), farnesyl (FPP), geranylgeranyl (GGPP) and geranylfarnesyl pyrophosphate (GFPP), showing the efficient conversion of GGPP into **3** and **4**, respectively, while all other substrates were not accepted (Figures S21 and S22).

The availability of the recombinant enzymes also allowed to confirm the absolute configurations of **3** and **4** through an isotopic labelling approach (labelling experiments are summarised in Table S6). The incubation of DMAPP and (*E*)‐ or (*Z*)‐(4–^13^C,4–^2^H)IPP^[^ ^25^ ^]^ with *Streptomyces cyaneofuscatus* GGPPS^[^ ^26^ ^]^ and AbPS1 allowed to install stereoselective deuterations at C4, C8 and C12 of **3** with known configurations.^[^ ^27^ ^]^ Additional stereoselective deuterations at C5, C9 and C13 were introduced through conversion of (*R*)‐ and (*S*)‐(1–^13^C,1–^2^H)IPP^[^ ^28^ ^]^ with GGPPS, *Escherichia coli* isopentenyl diphosphate isomerase (IDI)^[^ ^29^ ^]^ and AbPS1. The absolute configuration of **3** was then deduced through solving the relative configurations of the deuterated molecules (Figures S23 and S24). Corresponding experiments revealed the absolute configuration of **4** (Figures S25 and S26), confirming the results of the X‐ray analyses.

A biosynthetic proposal for **3** by Kubanek (Scheme S1)^[^ ^16^ ^]^ was refined during the course of this study (Scheme 1). Our first hypothesis starts with a GGPP conformation with Me18 and Me19 pointing down and Me20 up. The same conformation can be adopted by (*R*)‐geranyllinalyl pyrophosphate (GLPP), but not by (*S*)‐GLPP, assuming an *anti*‐S_N_2’ reaction in the initial 1,11‐cyclisation to **B**. This was confirmed through the incubation of both enantiomers of GLPP^[^ ^30^ ^]^ with AbPS1, showing a higher efficiency for the conversion of (*R*)‐GLPP into **3** than for (*S*)‐GLPP (Figure S27). A second 10,14‐cyclisation to **C** is followed by two 1,2‐hydride shifts to **D** and **E** and a 1,2‐Me group migration to **F**. Its deprotonation may directly lead to **H**, and a reprotonation at C3 can yield **I** with a *cisoid* allyl system that sets the stage for the cyclisation to **J**. A subsequent 1,2‐hydride shift to **K** enables cyclopropanation to **L** and water capture to **4**. Alternatively, **L** can undergo another 1,2‐hydride shift to **M** as the precursor of **3**.

This mechanism was supported by extensive isotopic labelling experiments. The AbPS1 catalysed conversion of all 20 isotopomers of (^13^C)GGPP, made accessible through synthesis^[^ ^26^, ^31^ ^]^ or from shorter oligoprenyl diphosphate^[^ ^32^ ^]^ and IPP isotopomers^[^ ^26^, ^33^, ^34^ ^]^ using GGPPS, revealed the origin of the skeleton of **3** and supported the 1,2‐Me group migration from **E** to **F** (Figures S28 and S29). Feeding of sodium (1–^13^C)acetate to the production strain for **4** confirmed the same origin of its carbon framework (Figure S30). The 1,2‐hydride shift from **C** to **D** was investigated using (3–^13^C,2–^2^H)DMAPP^[^ ^35^ ^]^ in conjunction with IPP, GGPPS and AbPS1. The obtained product exhibited an upfield shifted triplet for C7 of **3** (Δδ = –0.54 ppm, ^1^ *J*_C,D_ = 19.1 Hz), confirming a covalent ^13^C‐^2^H bond in the product (Figure S31). Employing the same strategy, the 1,2‐hydride shift from **D** to **E** was shown through incubation of (2–^13^C)DMAPP^[^ ^36^ ^]^ and (2,2–^2^H_2_)IPP (synthesised as in Scheme S2) with GGPPS and AbPS1 (Δδ = –0.64 ppm, ^1^ *J*_C,D_ = 19.1 Hz, Figure S32). The deprotonation‐reprotonation sequence **G**‐**H**‐**I** was investigated through several experiments. The AbPS1 catalysed conversion of (*S*)‐(1–^13^C,1–^2^H)GGPP, prepared with GGPPS from FPP and (*S*)‐(1–^13^C,1–^2^H)IPP, resulted in an upfield shifted triplet for C1 (Δδ = –0.38 ppm, ^1^ *J*_C,D_ = 23.7 Hz), confirming that the 1‐*pro*‐*S* hydrogen of GGPP stays at C1 (Figure S33). The analogous experiment with (*R*)‐(1–^13^C,1–^2^H)IPP produced two singlet signals for C1, one of which showed the exact same chemical shift as C1 of unlabelled **3**, in agreement with loss of the 1‐*pro*‐*R* hydrogen of GGPP. The second singlet exhibited a small upfield shift (Δδ = –0.10 ppm), which is typical for a deuterium residing in a neighbouring position (C2). This observation is explainable by a partial reincorporation of the same proton that is abstracted from C1 at C3 in **I**, and the later 1,2‐hydride shift from **L** to **M** defines its target position at C2. In a complementary experiment with (2–^13^C)GGPP in D_2_O buffer, the partial deuterium incorporation from the medium was localised at C2 (Δδ = –0.41 ppm, ^1^ *J*_C,D_ = 19.1 Hz, Figure S34), while the fraction of the 1‐*pro*‐*R* hydrogen remaining in the molecule was likewise localised at C2 in an enzymatic transformation of FPP and (2–^13^C,1,1–^2^H_2_)DMAPP^[^ ^37^ ^]^ with IDI, GGPPS and AbPS1 (Δδ = –0.54 ppm, ^1^ *J*_C,D_ = 19.4 Hz, Figure S35). Finally, the 1,2‐hydride migration from **J** to **K** was evident from the conversion of (3–^13^C,2–^2^H)FPP^[^ ^38^ ^]^ and IPP with GGPPS and AbPS1 (Δδ = –0.47 ppm, ^1^ *J*_C,D_ = 19.2 Hz, Figure S36).

This mechanism was further investigated through extensive DFT calculations. Most proposed transformations showed low reaction barriers, and the overall process is with –50.5 kcal mol^−1^ strongly exergonic (Table S7 and Figure S37). The deprotonation‐reprotonation sequence from **F** to **I** was realised using NH_3_ as a surrogate base.^[^ ^39^, ^40^ ^]^ However, a direct transformation of **F** to **H** was not possible (or at least not found computationally with the weak, but realistic surrogate base NH_3_), but instead, trials to remove H*_R_* ^+^ triggered a 1,2‐hydride migration to **G**, which can then be deprotonated to **H** (note that both hydrogens at C1 are not in hyperconjugation with the empty p‐orbital in **F**, box in Scheme 1). A high activation barrier of 20.3 kcal mol^−1^ was found for the 1,2‐hydride shift from **F** to **G** that results from different phenomena: First, **F** is a species of comparably low energy because it is well stabilised through a homoallylic cation‐π interaction between C1 and C3 with a distance of 2.1 Å that needs to be broken up towards the transition state **F‐G‐TS**, and second, this transition state encounters stereoelectronic destabilisation, because of a high strain in its twisted protonated diene unit and increasing steric hindrance between Me20 and C12. The maximum difference between a local minimum and a subsequent local maximum for the whole mechanism was found to be +28.7 kcal mol^−1^ and is associated with this **F**‐to‐**G** transformation (Figure S37). For the same reasons as discussed above for this step, a similarly high barrier can be expected for the deprotonation of **F** by an active site residue that will cause similar molecular mechanics as the 1,2‐hydride shift in **F**, questioning the relevance of the proposed and suggesting the need for an alternative mechanism.

Minor products of TSs can give additional insights into the catalytic mechanism and may be obtained with higher yields from enzyme variants. Structural models of AbPS1 and AbPS2 were generated using AlphaFold2^[^ ^41^ ^]^ (Figures 2a and S38; these structural models are generally considered to have a high reliability, but should of course be taken with care), with three Mg^2+^ ions added based on the structure of VenA (PDB: 7Y9G).^[^ ^42^ ^]^ Subsequently, GGPP was docked using Autodock Vina,^[^ ^43^ ^]^ resulting in the complex models of AbPS1/AbPS2‐GGPP‐Mg^2+^. The hydrophobic active site residues of AbPS1 were identified and investigated through Ala scanning (Figures S39 and S40, Tables S8 and S9). Most exchanges showed little effect or an abolished activity, but the AbPS1‐T182A variant exhibited a good production of a diterpene hydrocarbon that was also found in traces in the wildtype. Isolation from an incubation of GGPP with the purified enzyme (Figure S41) identified this compound as peyssonnosene (**5**) (Table S10, Figures S42–S51). In addition, anaerol (**6**) was isolated from a production strain expressing AbPS1‐T182A (Table S11, Figures S52–S59), and its absolute configuration and the loss of the 1‐*pro*‐*R* hydrogen were determined using the stereoselective deuteration strategy (Figures S60 and S61). This compound may be formed from **H** through reprotonation‐induced water addition (Scheme 1).

**Figure 2:**
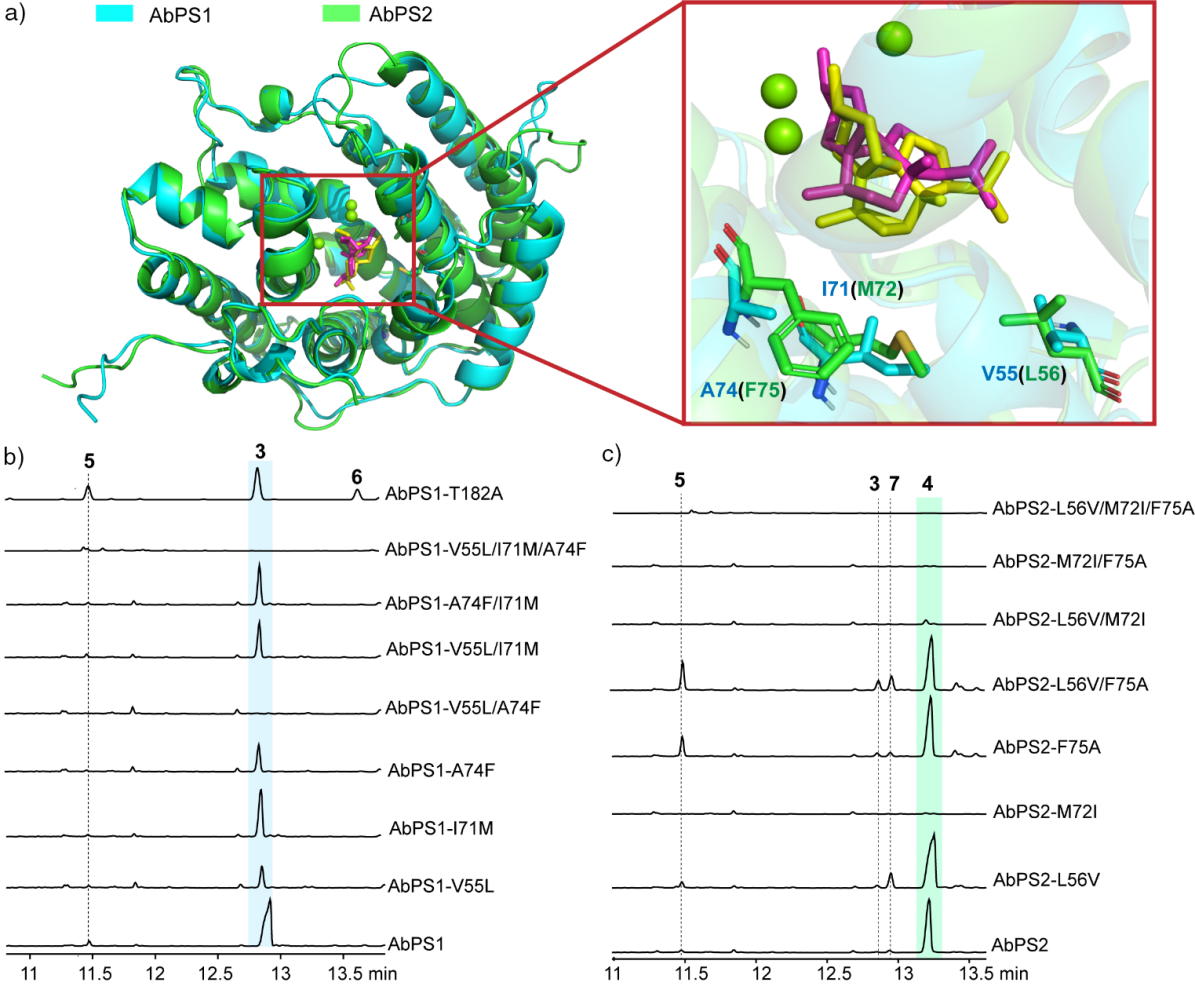
Functional analysis of AbPS1 and AbPS2 enzyme variants. a) Alignment of the modelled structures of AbPS1 (blue) and AbPS2 (green) docked with **L** and three Mg^2+^ added from the structure of VenA (PDB: 7Y9G), and close‐up view on the active site residues differing in the two enzymes. Total ion chromatograms from GC‐MS analyses of extracts of *S. cerevisiae* production cultures harbouring b) AbPS1 and c) AbPS2 and their enzyme variants.

Despite their low sequence identity, AbPS1 and AbPS2 share a common mechanism, differing only in the final hydroxylations to **3** versus **4**. A comparison of the two models docked with **L** revealed a high structural similarity between the two enzymes (root mean square deviation, RMSD = 0.831), with only three different residues in the active sites. To test their role for product selectivity, site‐directed mutagenesis experiments with residue swapping were conducted (Figure 2b,c). For both enzymes, the three single exchanges, three combinations of double exchanges and the triple variant were created. In only three cases, a partial swap of the production from **4** to **3** by AbPS2 was observed (AbPS2‐L56V, AbPS2‐F75A and AbPS2‐L56V‐F75A), but no full functional change could be achieved. Instead, the AbPS2‐L56V‐F75A variant showed the formation of a diterpene alcohol that was also observed in traces in the wildtype enzymes, and structurally characterised as anaerol B (**7**, Table S12, Figures S63–S70). Its absolute configuration was determined through stereoselective labelling experiments (Figure S71). Compound **7** is a stereoisomer of 11‐hydroxyvulgarisane, a compound for which the configurations of several stereogenic centres were not determined, with different NMR data to **7**.^[^ ^44^ ^]^

The biosynthesis of **7** is explainable from GGPP with a different starting conformation as discussed in the first mechanistic hypothesis, with all three Me groups pointing down, that can explain a late‐stage double cyclisation from **I′** to **O′** (Scheme 2a). After ionisation to **A′** a 1,11–10,14‐cyclisation results in **C′**, and two subsequent 1,2‐hydride shifts and a 1,2‐methyl migration lead via **D′** and **E′** to **F′**. A subsequent 1,2‐hydride shift to **G′**, deprotonation to **H′** and reprotonation result in **I′**. A terminal 2,6‐cyclisation to **N′**, a 1,7‐cyclisation to **O′** and capture with water yield **7**. A recently proposed pathway to tetraisoquinene (**8**, box in Scheme 2) proceeds through similar steps to a stereoisomer of **O′**.^[^ ^45^, ^46^ ^]^ DFT calculations (Table S13, Figure S72) revealed low activation barriers for all elementary steps and in particular resolved the situation for the problematic step (**F** to **G**) of Scheme 1. The corresponding 1,2‐hydride shift **F′** to **G′** (Scheme 2a) is not associated with steric hindrance causing a twisted allyl system and thus shows a much lower activation barrier. A conformational change in **H′**, leading to intermediate **H** of Scheme 1, could open a pathway to **3** (Scheme 2b). This biosynthetic scenario is also in agreement with all labelling experiments, but the DFT calculations revealed a high barrier for the conformational change (27.2 kcal mol^−1^). Furthermore, the starting conformer of GGPP should be substitutable by (*S*)‐GLPP, and not (*R*)‐GLPP, which questions the relevance of this second biosynthetic mechanism for **3**.

**Scheme 2:**
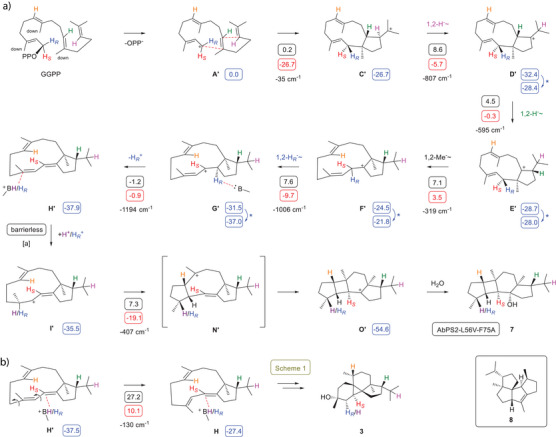
Cyclisation mechanism of AbPS1‐L56V‐F75A. a) Cyclisation mechanism from GGPP to **7**. ^a)^ Because of the high reactivity of diene **H′** towards NH_4_^+^, the starting structure of **H′** for the reaction to **I′** could not be localised. The approximated transition state for this step did not fully converge, but the reaction was estimated to be barrierless. B) Conformational change from **H′** to **H** that connects the steps from GGPP to **H′** (Scheme 2ab) with the steps from **H** to **3** – **5** (Scheme 1). Cf. legend of Scheme 1 for explanations regarding computational data. Box: Structure of biosynthetically related tetraisoquinene (**8**).

The cyclisation mechanism of Scheme 1 explains the observed product formation from (*R*)‐GLPP, but does not require GLPP as an intermediate, because no conformational change by rotation around the C2─C3 bond before cyclisation is considered in this pathway.^[^ ^47^ ^]^ A third alternative mechanism that is also in line with all labelling experiments may proceed through (*R*)‐GLPP and involve such a conformational change (Scheme 3). This pathway starts with the isomerisation of GGPP to (*R*)‐GLPP and its conformational change by vinyl group rotation. Ionisation to **A″** allows for a 1,11–10,14‐cyclisation to **C″** with installation of a *Z*‐configured double bond. The hydrogens at C1 in **C″** now reside in different positions as compared to the previous mechanisms. This is followed by two 1,2‐hydride migrations and a 1,2‐Me group shift towards **F″** that is associated with a major conformational change caused by a cation‐π interaction, turning Me20 from the front to the back side (Scheme 3b). The base in the deprotonation‐reprotonation sequence operates now on the other side of the diene portion for selective migration or removal of H*_R_*, respectively. Notably, the deprotonation in **F″** with NH_3_ was computationally achievable without a prior 1,2‐hydride shift. The reprotonation of **H″** then resulted in **I**. The terminal steps to **3** – **5** are the same as in Scheme 1. DFT computations for this pathway (Table S14, Figure S73) revealed a smooth passage with low activation barriers for all steps, and also anaerol (**6**) can be explained through reprotonation of **H″** to **P″** and capture with water (Scheme 3).

**Scheme 3:**
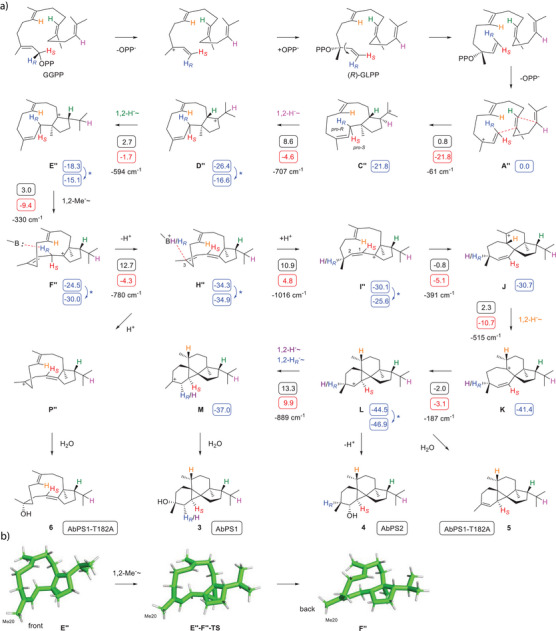
Improved model for the AbPS1/AbPS2 mechanism. a) Cyclisation mechanism from GGPP through the intermediate (*R*)‐GLPP to **3** – **5**. Cf. legend of Scheme 1 for explanations regarding computational data. b) Computed structures of intermediate **E″**, transition state **E’’‐F’’‐TS**, and intermediate **F″**.

In extension of a study on the biosynthesis of typical coral‐derived diterpenes,^[^ ^48^ ^]^ we have identified two bacterial DTSs for the red algal compound peyssonnosol (**3**) and its regioisomer peyssonnosol B (**4**). The identical absolute configurations of bacterial and algal **3** may question the biosynthetic origin of the diterpenes in *Peyssonnelia*. However, recent studies on TSs from corals and sponges have demonstrated that the animals are indeed the natural producers of diterpenes,^[^ ^14^, ^15^, ^49^ ^]^ contradicting a longstanding debate in which the production of secondary metabolites was attributed to microbial symbionts. Also, algal TSs have recently been identified.^[^ ^50^, ^51^ ^]^

The mechanisms of AbPS1 and AbPS2 (PeyS) were investigated through isotopic labelling experiments, DFT calculations and site‐directed mutagenesis. Notably, the isotopic labelling experiments were in line with several plausible mechanisms. A refinement is possible through DFT calculations in which the highest reaction barrier along the cascade should be lower than ∼20 kcal mol^−1^. In the present case, a deep stereochemical analysis and probing with both enantiomers of GLPP gave additional information, and site‐directed mutagenesis gave access to pathway intermediates for further conclusions. The third mechanistic hypothesis presented in this study currently seems to be the best model for the biosynthesis of **3** and **4**, while the biosynthesis of the side product anaerol B (**7**) may not directly be connected to this mechanism, but rather start from a different GGPP conformation. Extensive trial and error is usually required to reach conclusive evidence in mechanistic investigations on TSs. At the end, it should be emphasised that any mechanistic model, in terpene biosynthesis and in chemistry, only holds true as long as it has not been falsified.

## Conflict of Interests

The authors declare no conflict of interest.

## Supporting information



Supporting information

## Data Availability

The data that support the findings of this study are available in the Supporting Information of this article.
