# Supplementary material for: Biosynthesis of the Red Algal Diterpene Peyssonnosol in Bacteria
Source: Angew Chem Int Ed Engl. 2025 Jul 2;64(34):e202507752. doi: 10.1002/anie.202507752 (PMC12363642; doi:10.1002/anie.202507752)
Supplement: Supplementary file 1 — Supporting information [file ANIE-64-e202507752-s001.pdf]

Table of Contents

Phylogenetic tree	2
Strains and culture conditions, construction of yeast production strains, compound isolation and spectroscopic data of **3** and **4**	3
Spectroscopic methods	4
Oligonucleotid primers	5
Structure elucidation and NMR data of **3**	6
Structure elucidation and NMR data of **4**	11
X-ray analysis of **3** and **4**	16
Gene cloning, expression and protein purification	21
In vitro incubations with AbPS1 and AbPS2	22
EI mass spectra of **3** and **4**	24
List of isotopic labelling experiments	25
The absolute configuration of **3**	26
The absolute configuration of **4**	28
Biosynthetic proposal for peyssonnoside A by Kubanek	30
Incubation experiments with (*R*)- and (*S*)-GLPP	31
^{13}C Labelling of individual carbons	32
The 1,2-hydride shift from **C** to **D** in the formation of **3**	35
Synthesis of (2,2-$^{2}H_2$)IPP	36
The 1,2-hydride shift from **D** to **E** in the formation of **3**	39
The deprotonation-reprotonation sequence in the formation of **3**	40
The 1,2-hydride shift from **J** to **K** in the formation of **3**	43
Computational methods	44
Results of DFT calculations for Scheme 1 of main text	45
Molecular simulations for AbPS1 and AbPS2	47
Site-directed mutagenesis	48
Heterologous expression, purification and incubation of enzyme variants	52
EI mass spectra of **5** – **7**	54
Isolation and spectroscopic data of **5** – **7**	55
Structure elucidation and NMR data of **5**	56
Structure elucidation and NMR data of **6**	61
The absolute configuration of **6**	66
The loss of the 1-*pro*-*R* hydrogen in the biosynthesis of **6**	68
Structure elucidation and NMR data of **7**	69
The absolute configuration of **7**	74
Results of DFT calculations for Scheme 2 of main text	75
Results of DFT calculations for Scheme 3 of main text	77
Cartesian coordinates of computed structures for Scheme 1 of main text	79
Cartesian coordinates of computed structures for Scheme 2 of main text	118
Cartesian coordinates of computed structures for Scheme 3 of main text	147
References	167

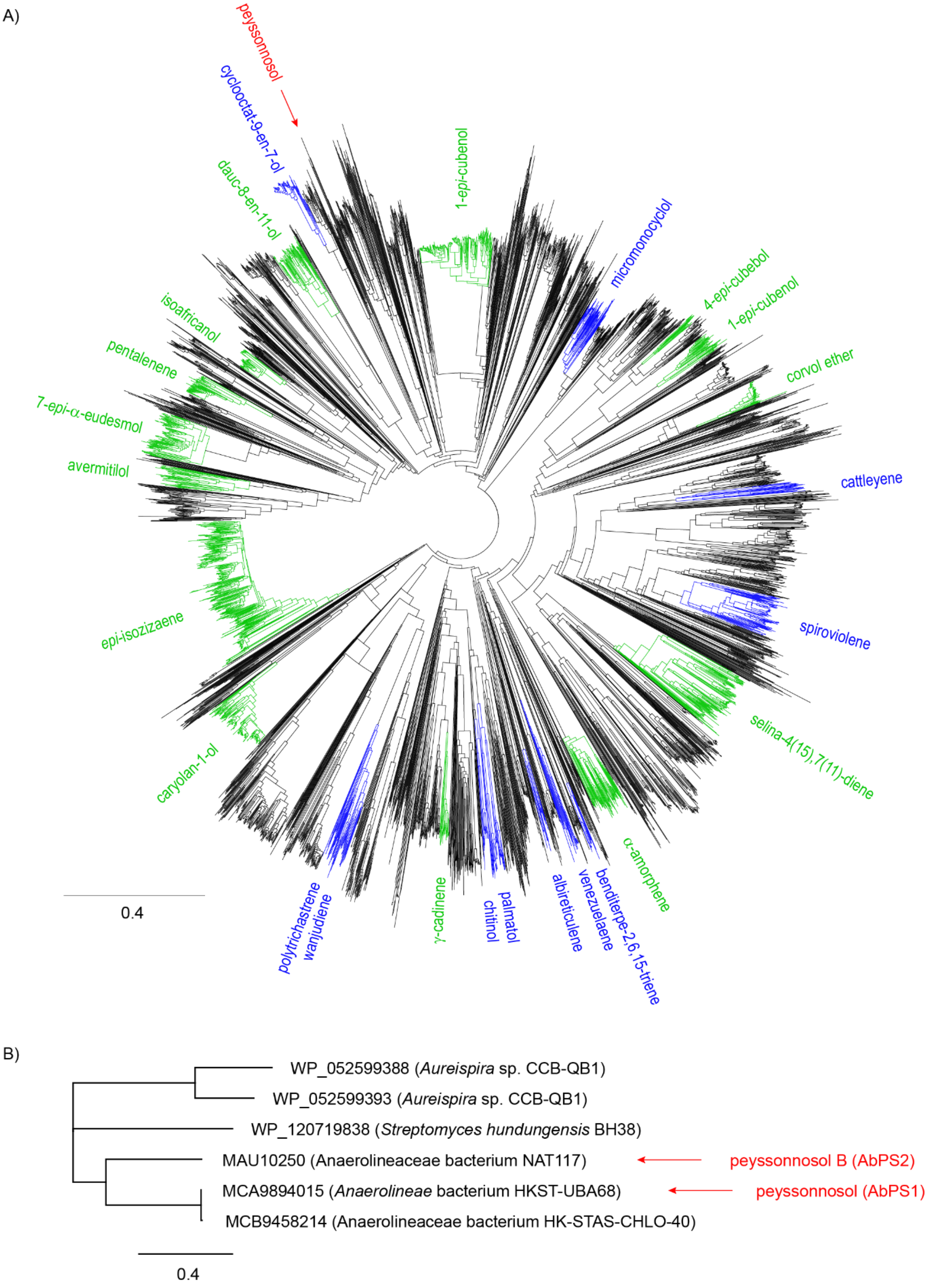

Figure S1. A) Phylogenetic tree constructed from the amino acid sequences of 5000 terpene synthase homologs. Characterised sesquiterpene synthases and their homologs are shown in green, characterised diterpene synthases and their homologs shown in blue. B) Close-up view on the branch containing the newly characterised diterpene synthases for peyssonnosol (**3**) and peyssonnosol B (**4**). The scale bars indicate substitutions per site.

Strains, culture conditions and media

E. coli BL21 (DE3) *F⁻dcm ompT hsdSB (rB⁻mB⁻) gal* was obtained from Invitrogen (Carlsbad, CA, USA). *Saccharomyces cerevisiae* YZL141 (*CEN.PK2-1D, gal1Δ, gal7Δ, gal10Δ:: TRP1*) was used as the host for heterologous expression. Transformants of the *Saccharomyces cerevisia*e strain were grown in shaking cultures in YPD medium at 30 °C and 220 rpm.

Construction of a *Saccharomyces cerevisiae* strain for the production of 3 and 4

The coding sequence of *AbPS1* and *AbPS2* was synthesized by Beijing Tsingke Biotech Co., Ltd., and codon optimized for *S. cerevisiae.* The yeast transformation fragment was amplified using primers AbPS1-SC-F/R and AbPS2-SC-F/R (Table S1), followed by cloning into pESC-URA using the ClonExpress® II One Step Cloning Kit C112 (Vazyme, Nanjing, China) to yield plasmid pESC-AbPS1 and pESC-AbPS1. Gene integrations for the construction of engineered yeast strains were conducted using the LiOAc/salmon sperm carrier DNA/polyethyleneglycol method[1] and verified using diagnostic polymerase chain reaction.

Cultivation of the *Saccharomyces cerevisiae* production strain and compound isolation of 3 and 4

A culture of *S. cerevisiae* expressing AbPS1 in YPD medium (3 L) was grown for 3 days at 30 °C and 220 rpm. The culture was extracted hexane/ethyl acetate = 4/1 (3x 1.5 L) and the combined extracts were concentrated to obtain a dark brown oil (560 mg). Purification by semipreparative HPLC (Prevail C18 column, ACN/H_2O=100/0, flow rate=3.0 mL/min, λ=210 nm) afforded **3** (25.4 mg, t_R = 22.1 min, yield 8.4 mg/L).

A culture of *S. cerevisiae* expressing AbPS2 in YPD medium (3 L) was grown for 3 days at 30 °C and 220 rpm. The culture was extracted hexane/ethyl acetate = 4/1 (3x 1.5L) and the combined extracts were concentrated to obtain a dark brown oil (560 mg). Purification by semipreparative HPLC (Prevail C18 column, ACN/H_2O=100/0, flow rate=3.0 mL/min, λ=210 nm) afforded **4** (14.2 mg, t_R = 19.5 min, yield 4.7 mg/L).

Peyssonnosol (3). TLC (petrol ether/ethyl acetate = 5/1): R_f = 0.59. GC (HP-5MS): *I* = 2100. IR (diamond ATR): ṽ = 3345 (br), 2955 (s), 2916 (s), 2866 (s), 1460 (m), 1373 (m), 1246 (w), 1162 (w), 1128 (w), 1097 (w), 1080 (w), 1031 (m), 1000 (w), 923 (w) cm^{-1}. HR-MS (EI): calc. $[C_{20}H_{34}O]^+$ *m*/*z* = 290.2604; found: *m*/*z* = 290.2611. Optical rotation: $[\alpha]_D^{25}$ = –24.8 (*c* 0.58, CH_2Cl_2), lit.: $[\alpha]_D^{22}$ = –31.2 (*c* 0.3, $CHCl_3$).[2] NMR data are given in Table S2.

Peyssonnosol B (4). TLC (petrol ether/ethyl acetate= 5/1): R_f = 0.64. GC (HP-5MS): *I* = 2135. IR (diamond ATR): ṽ = 3330 (br), 2952 (s), 2867 (s), 1462 (m), 1375 (m), 1258 (w), 1091 (w), 1066 (w), 1028 (m) cm^{-1}. HR-MS (EI): calc. $[C_{20}H_{34}O]^+$ *m*/*z* = 290.2604; found: *m*/*z* = 290.2613. Optical rotation: $[\alpha]_D^{25}$ = –44.1 (*c* 0.17, CH_2Cl_2). NMR data are given in Table S3.

Chemicals, solvents and chromatographic methods

Chemicals were purchased from Sigma Aldrich Chemie GmbH (Steinheim, Germany), Carbolution Chemicals GmbH (St. Ingbert, Germany), or Carl Roth (Karlsruhe, Germany) and used without purification. Solvents for column chromatography were purchased in p.a. grade and purified by distillation. Thin-layer chromatography (TLC) was performed with 0.2 mm precoated plastic sheets Polygram Sil G/UV254 purchased from Machery-Nagel (Düren, Germany). Column chromatography was performed using silica gel 60 purchased from Merck (Darmstadt, Germany).

NMR spectroscopy

NMR spectra were recorded on a Bruker (Billerica, MA, USA) Avance I (300 MHz), Avance I (400 MHz), Avance I (500 MHz), Avance III HD Prodigy (500 MHz) or an Avance III HD Cryo (700 MHz) NMR spectrometer. Spectra were measured in C_6D_6 and referenced against solvent signals (^{1}H-NMR, residual proton signal: δ = 7.16; ^{13}C-NMR: δ = 128.06).[3]

GC/MS and GC/MS-QTOF analyses

GC/MS analyses were performed on a 5977A GC/MSD system (Agilent, Santa Clara, CA, USA) with a 7890B GC and a 5977A mass selective detector. The GC was equipped with a HP5-MS fused silica capillary column (30 m, 0.25 mm i. d., 0.50 μm film). Specific GC settings were 1) inlet pressure: 77.1 kPa, He at 23.3 mL min^{-1}, 2) injection volume: 1 μL, 3) temperature program: 5 min at 50 °C increasing at 10 °C min^{-1} to 320 °C, 4) 60 s valve time, and 5) carrier gas: He at 1.2 mL min^{-1}. MS settings were 1) source: 230 °C, 2) transfer line: 250 °C, 3) quadrupole: 150 °C and 4) electron energy: 70 eV. Retention indices (*I*) were determined from retention times in comparison to the retention times of *n*-alkanes (C_7-C_{40}). GC/MS-QTOF analyses were performed on a 7890B GC equipped with a HP5-MS fused silica capillary column (30 m, 0.25 mm i. d., 0.50 µm film) connected to a 7200 accurate-mass QTOF detector (Agilent). GC parameters were 1) inlet pressure: 83.2 kPa, He at 24.6 mL min^{-1}, 2) injection volume: 1 µL, 3) split ratio: 50:1, 60 s valve time, 4) temperature program: 5 min at 50 °C increasing at 5 °C min^{-1} to 320 °C, 5) carrier gas: He at 1 mL min^{-1}. MS parameters were 1) transfer line: 250 °C, 2) electron energy 70 eV.

IR spectroscopy

IR spectra were recorded on a Bruker α infrared spectrometer with a diamond ATR probehead. Peak intensities are given as s (strong), m (medium), w (weak) and br (broad).

Optical rotations

Optical rotations were recorded on a Modular Compact Polarimeter MCP 100 (Anton Paar, Graz, Austria). The temperature setting was 25 °C; the wavelength of the light used was 589 nm (sodium D line); the path-length was 10 cm; the compound concentrations *c* are given in g 100 mL^{-1}.

Table S1. Oligonucleotide primers for the construction of plasmids used in this study.[a]

Primers	Sequence 5' → 3'
AbPS1-SC-F	GAGAAAAAACCCCGGATCCATGGTTTGGTCTGGTTTGTTG
AbPS1-SC-R	CGGTACCAAGCTTACTCGAGTCATAGAACTTGTCTCAATTCTTG
AbPS1-32a-F	AGGGCGCTGGTGCTGGTGCTATGGTTTGGAGCGGCCTGCT
AbPS1-32a-R	TCAGCCATGGCAAGCTTTTACAGAACCTGACGCAGTTCCT
AbPS2-SC-F	GAGAAAAAACCCCGGATCCATGGTTCAATCTGGTTTGCTAA
AbPS2-SC-R	GTACCAAGCTTACTCGAGTAGTAGATGTTTCAACTCACTGAATGG
AbPS2-C6T-F	TGCTGATGGGCGGCCGCATGGTTCAGTCCGGTCTGCTGAC
AbPS2-C6T-R	CGTTTTATTTGAAGCTTTCACAGCAGGTGTTTCAGTTCAG
AbPS1-M51A-F	CACTACT**GCT**TCTCCATTCGTCTTTCCAAGAAC
AbPS1-M51A-R	CACTACT**GCT**TCTCCATTCGTCTTTCCAAGAAC
AbPS1-V55A-F	CTCCATTC**GCT**TTTCCAAGAACTTCTACTAAGAGACTGTT
AbPS1-V55A-R	TGGAAA**AGC**GAATGGAGACATAGTAGTGTAGTTGTCATAG
AbPS1-I71A-F	TGTTG**GCT**AATTTCGCCTTCTATGTCGATGAT
AbPS1-I71A-R	GGCGAAATT**AGC**CAACAAATCGATAGCCAACAGTCT
AbPS1-F75A-F	TTTCGCC**GCT**TATGTCGATGATGATATCAACGACG
AbPS1-F75A-R	CGACATA**AGC**GGCGAAATTGATCAACAAATCG
AbPS1-S177A-F	GAGAACATTTG**GCT**GGTATGTTGATGACTGTTGACTTGACT
AbPS1-S177A-R	ACC**AGC**CAAATGTTCTCTGAAGTAGATGTACTTCTG
AbPS1-T182A-F	GTTGATG**GCT**GTTGACTTGACTGAATTTGCCTTC
AbPS1-T182A-R	AGTCAAC**AGC**CATCAACATACCAGACAAATGTTCTC
AbPS1-M181A-F	GGTATGTTG**GCT**ACTGTTGACTTGACTGAATTTGCC
AbPS1-M181A-R	ACAGT**AGC**CAACATACCAGACAAATGTTCTCTGA
AbPS1-G215A-F	CCTCTCAAATT**GCT**TGCTTGTCTAACGACATCTTTAGCT
AbPS1-G215A-R	GCA**AGC**AATTTGAGAGGTGTTTCTGTACATTTG
AbPS1-W303A-F	TTGGCAT**GCT**CAAGCTACTACTAACAGATATAGGGACCC
AbPS1-W303A-R	TAGCTTG**AGC**ATGCCAAGCAGCGGTGATAATA
AbPS1-Q304A-F	GCATTGG**GCT**GCTACTACTAACAGATATAGGGACCCAG
AbPS1-Q304A-R	TAGTAGC**AGC**CCAATGCCAAGCAGCGGTGATA
AbPS1-V55L-F	CTCCATTC**TTG**TTTCCAAGAACTTCTACTAAGAGACTGTT
AbPS1-V55L-R	TGGAAA**CAA**GAATGGAGACATAGTAGTGTAGTTGTCATA
AbPS1-I71M-F	TGTTG**ATG**AATTTCGCCTTCTATGTCGATGAT
AbPS1-I71M-R	GGCGAAATT**CAT**CAACAAATCGATAGCCAACAGTCT
AbPS1-A74F-F	C**TTC**TTCTATGTCGATGATGATATCAACGACG
AbPS1-A74F-R	CATCGACATAGAA**GAA**GAAATTGATCAACAAATCGATAGCC
AbPS2-L56V-F	TGCATAC**GTT**TATCCACATACTTCTGAAGCTAAGTTAGTT
AbPS2-L56V-R	GTGGATA**AAC**GTATGCAGACATAGTAGCGTATTCGTT
AbPS2-M72I-F	CTGTTAACGTTCTA**ATC**AATATGTTCTTCTACGTTGACGATGT
AbPS2-M72I-R	**GAT**TAGAACGTTAACAGCAACTAACTTAGCTT
AbPS2-F75A-F	TG**GCT**TTCTACGTTGACGATGTCATCAATGAT
AbPS2-F75A-R	GTCAACGTAGAA**AGC**CATATTCATTAGAACGTTAACAGCAAC

[a] Homology arms for homologous recombination in yeast are underlined. Modified triplet codons in mutational primers are highlighted in bold.

Table S2. NMR data of peyssonnosol (**3**) in C_6D_6 recorded at 298 K.

C[a]	type	^{13}C[b]	^{1}H[b]
1	CH	13.53	0.46 (dd, J = 9.7, 2.7)
2	CH_2	33.49	1.78 (ddd, J = 15.0, 9.7, 2.6, H_α)<br>1.08 (m, H_β)
3	C_q	66.88	–
4	CH_2	34.30	1.34 (ddd, J = 13.8, 7.7, 2.5, H_α)<br>0.83 (ddd, J = 13.6, 12.5, 7.2, H_β)
5	CH_2	22.10	1.96 (ddd, J = 14.7, 12.6, 7.8, H_α)<br>1.38 (dd, J = 14.7, 7.2, H_β)
6	C_q	25.30	–
7	CH	35.22	1.45 (m)
8	CH_2	27.04	1.20 (m, H_β)<br>0.67 (m, H_α)
9	CH_2	39.55	1.52 (ddd, J = 12.9, 4.5, 2.2, H_α)<br>0.91 (ddd, J = 13.2, 13.2, 2.6, H_α)
10	C_q	42.05	–
11	C_q	37.86	–
12	CH_2	25.38	1.25 (m, H_β)<br>1.20 (m, H_α)
13	CH_2	28.63	1.74 (m, H_α)<br>1.24 (m, H_α)
14	CH	62.16	1.18 (m)
15	CH	29.29	1.59 (m)
16	CH_3	23.34	0.96 (d, J = 5.9)
17	CH_3	23.66	0.97 (d, J = 5.9)
18	CH_3	19.02	0.68 (s)
19	CH_3	19.81	1.08 (d, J = 6.4)
20	CH_3	30.26	1.10 (s)

[a] Carbon numbering as shown in Figure S2 indicates the origin of each carbon from GGPP by same number. [b] Chemical shifts δ in ppm. Multiplicity: s = singlet, d = doublet, m = multiplet. Coupling constants J are given in Hertz.

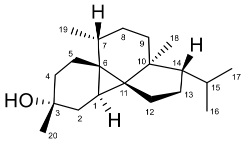

3

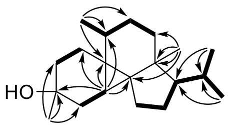

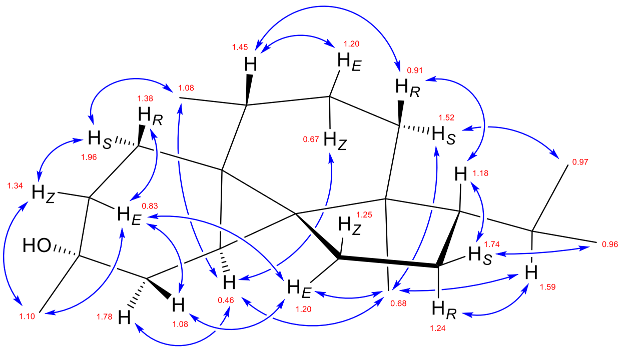

Figure S2. Structure elucidation of peyssonnosol (**3**). Bold: $^{1}H,^{1}H$-COSY, single headed arrows: key HMBC, and blue double headed arrows: NOESY correlations. H_R, H_S, H_E and H_Z indicate the results from stereoselective labelling experiments (Figures S23 and S24).

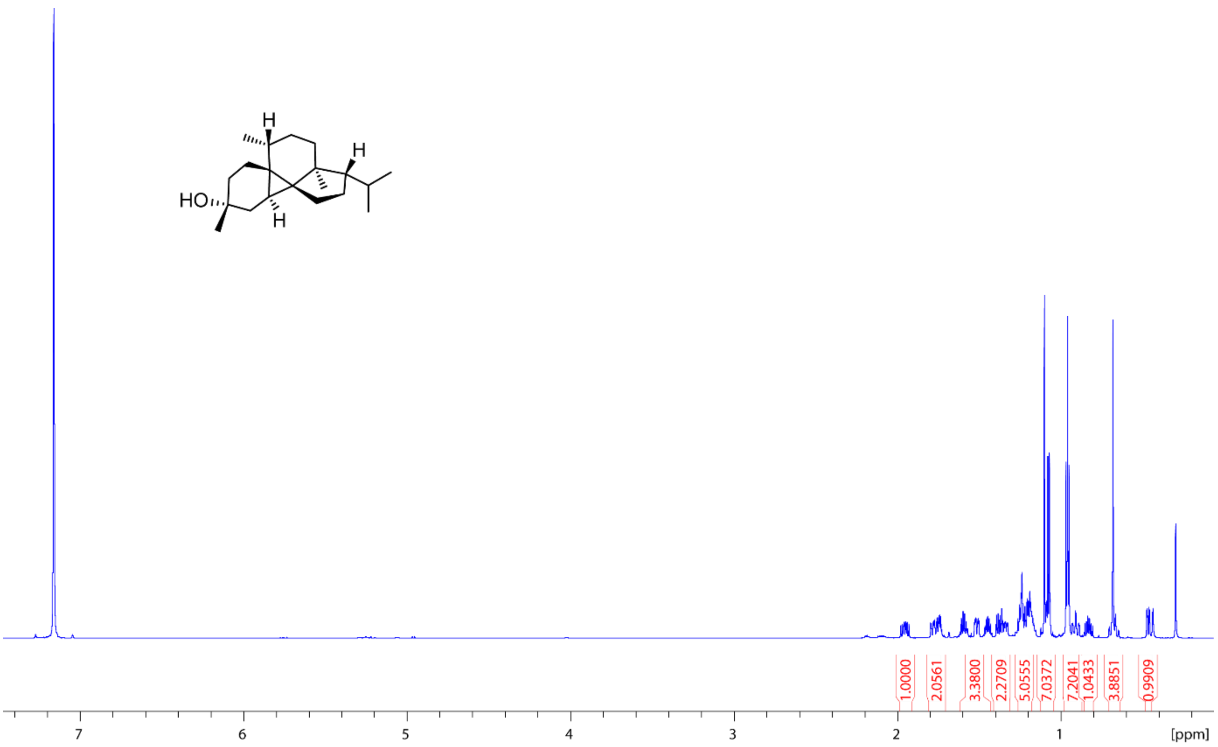

Figure S3. ^{1}H-NMR spectrum of **3** (700 MHz, C_6D_6).

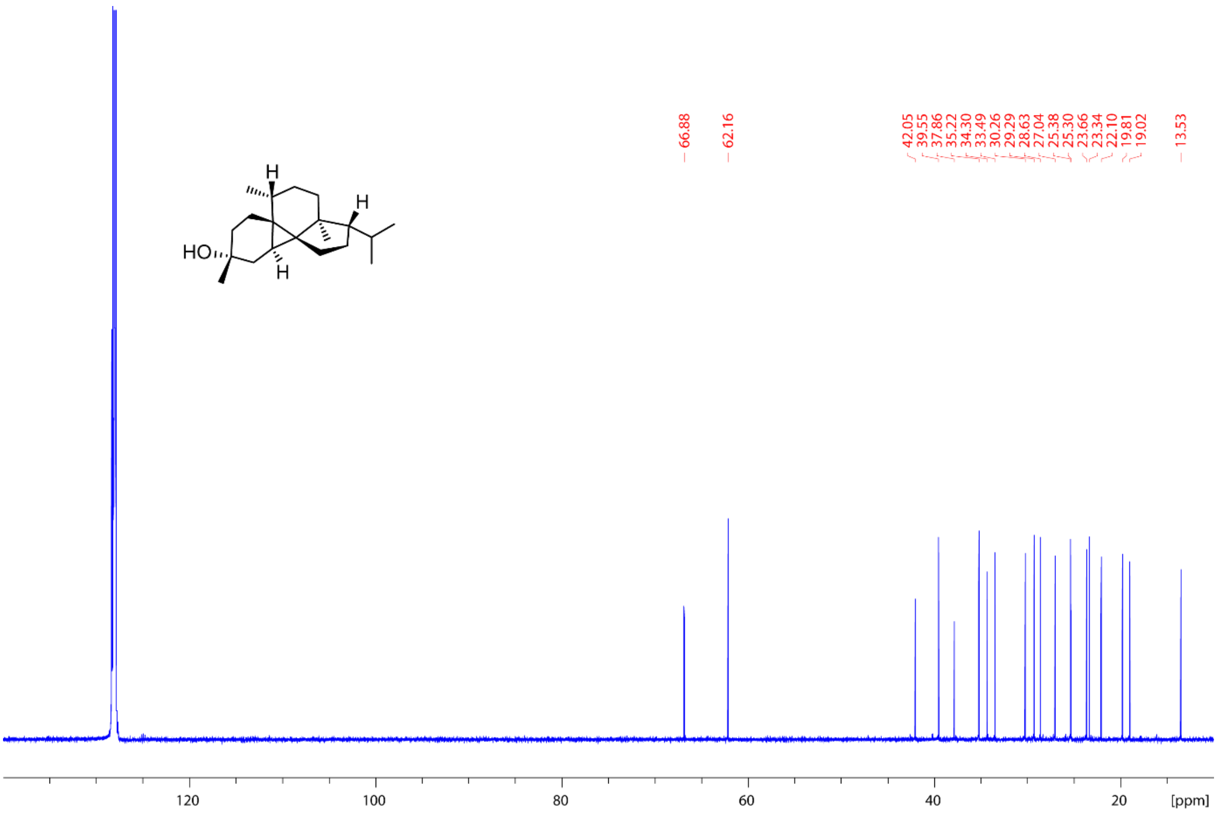

Figure S4. ^{13}C-NMR spectrum of **3** (176 MHz, C_6D_6).

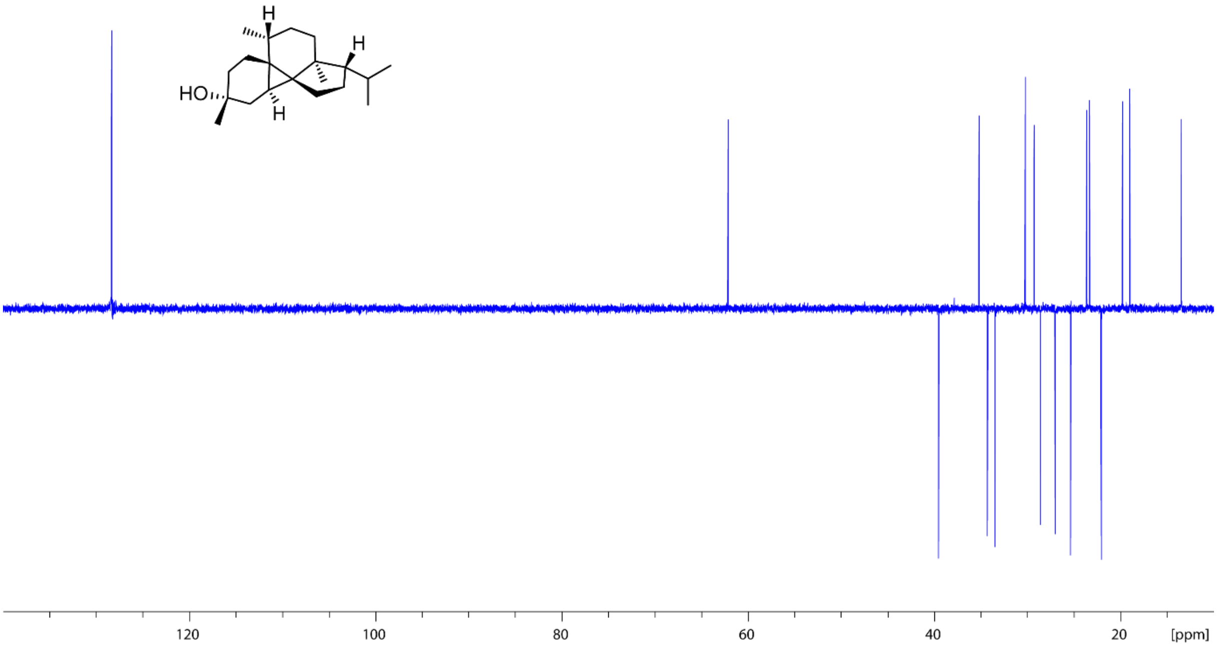

Figure S5. ^{13}C-DEPT135 spectrum of **3** (176 MHz, C_6D_6).

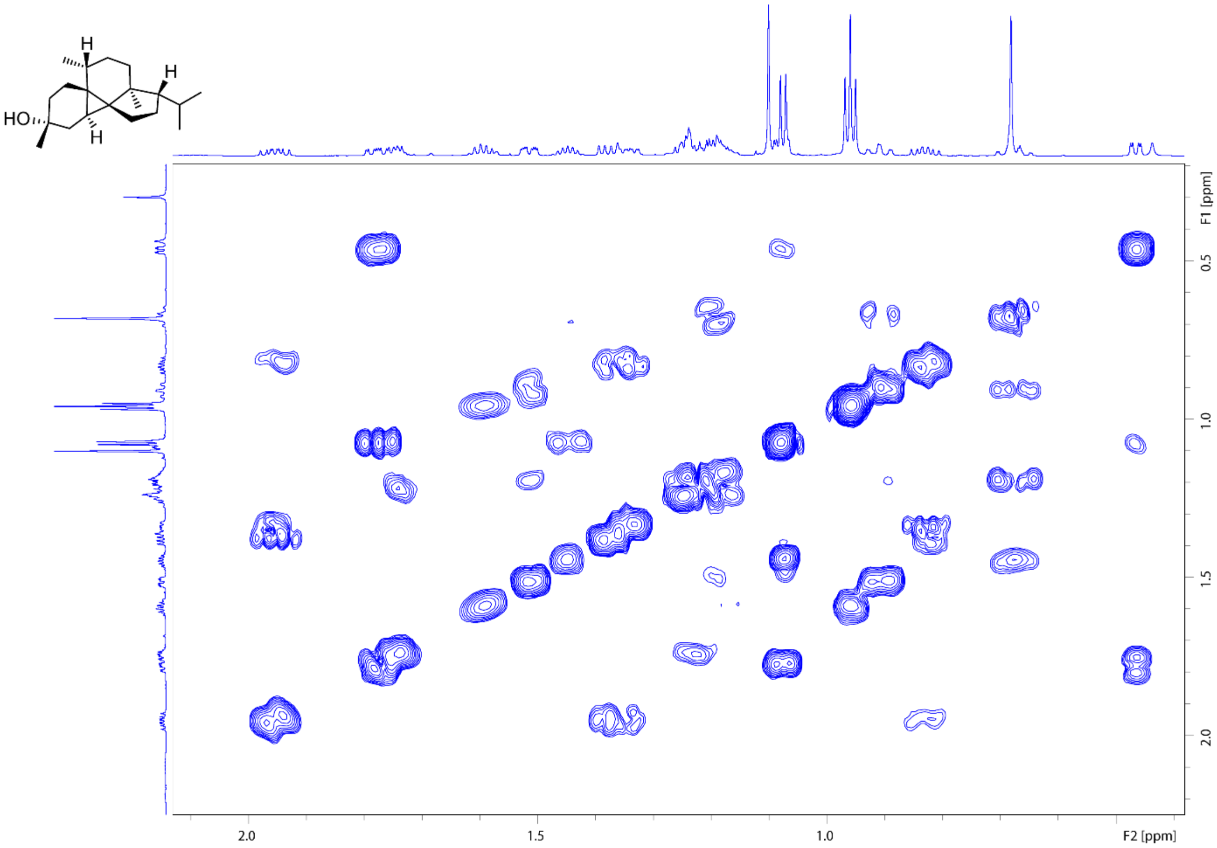

Figure S6. ^{1}H,^{1}H-COSY spectrum (C_6D_6) of **3**.

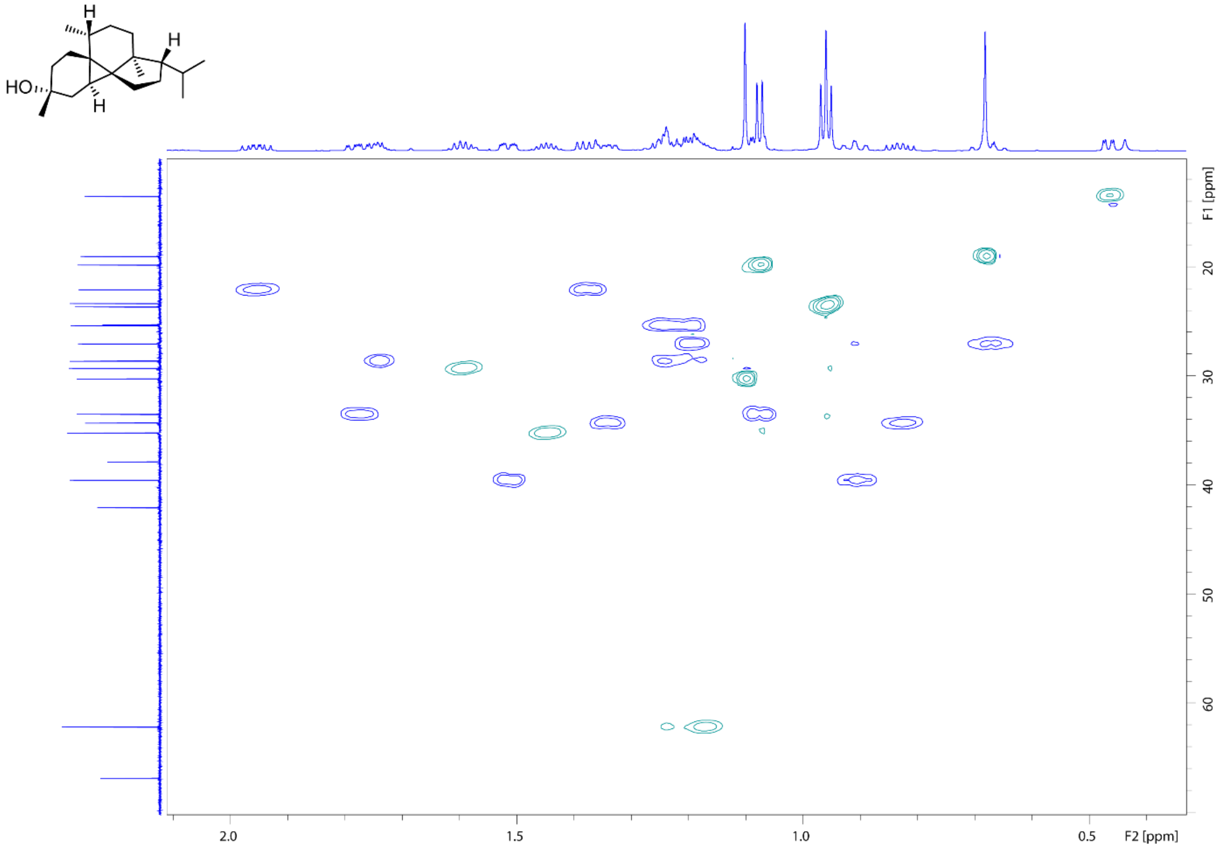

Figure S7. HSQC spectrum (C_6D_6) of **3**.

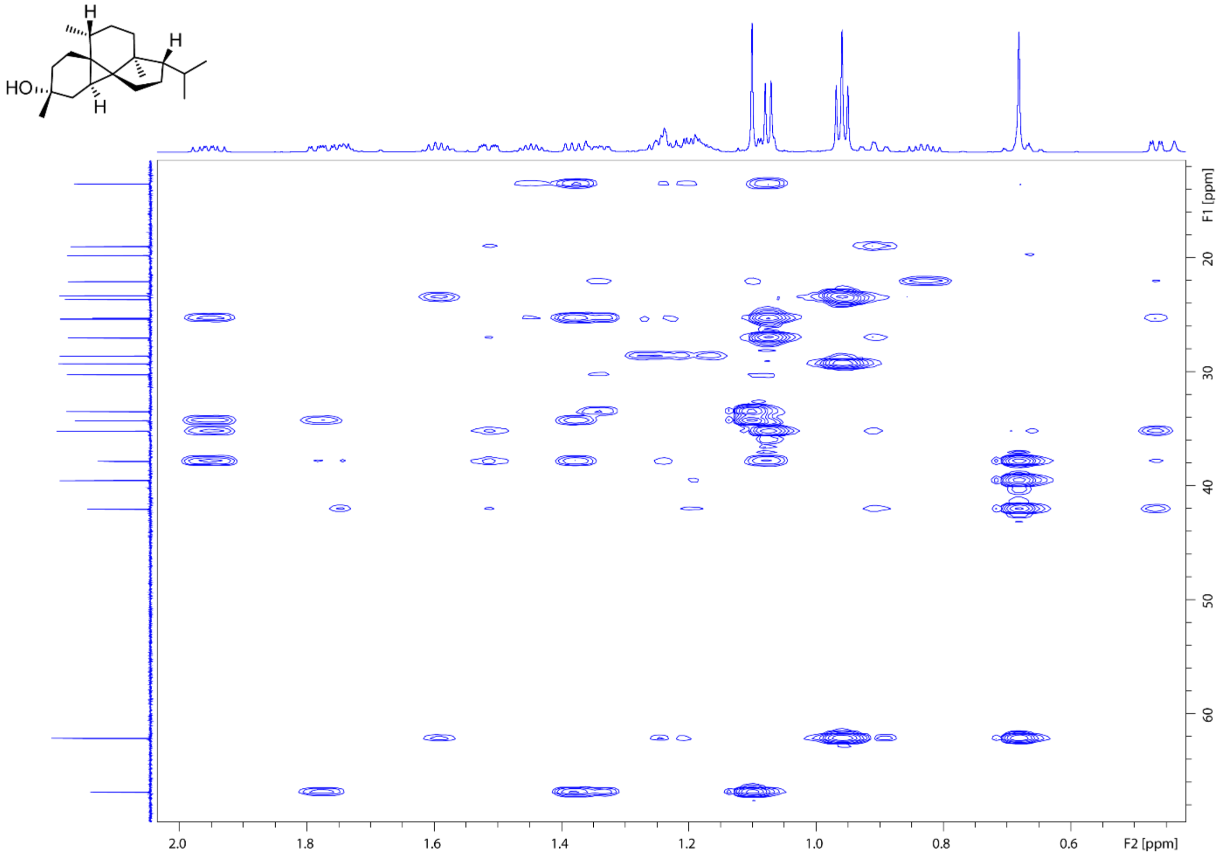

Figure S8. HMBC spectrum (C_6D_6) of **3**.

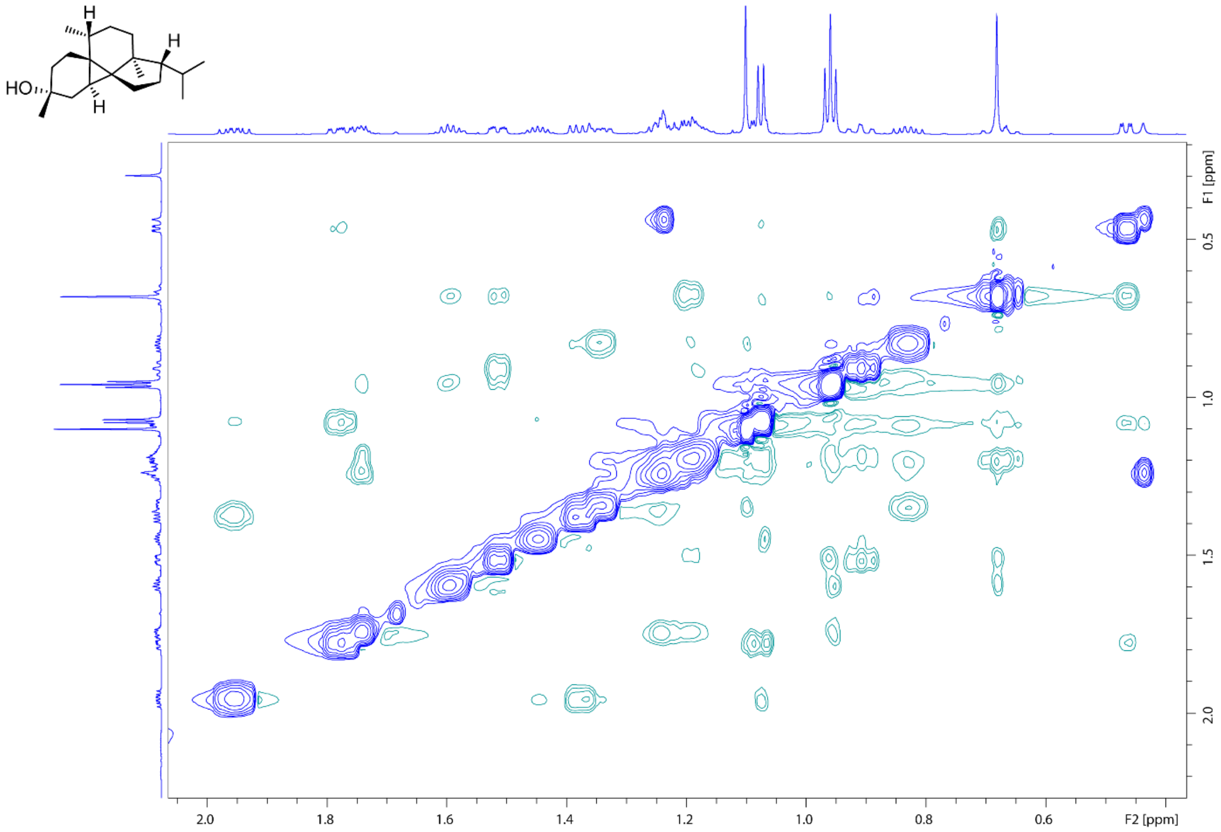

Figure S9. NOESY spectrum (C_6D_6) of **3**.

Table S3. NMR data of peyssonnosol B (**4**) in C_6D_6 recorded at 298 K.

C[a]	type	^{13}C[b]	^{1}H[b]
1	CH	26.88	0.61 (d, J = 1.5)
2	CH	72.84	2.91 (d, J = 9.8)
3	CH	38.36	1.06 (m)
4	CH_2	29.78	1.24 (m, H_α)<br>0.57 (dddd, J = 13.5, 13.0, 6.7, H_β)
5	CH_2	25.29	1.66 (dddd, J = 13.5, 13.0, 6.7, H_α)<br>1.42 (m, H_β)
6	C_q	28.47	–
7	CH	34.50	1.40 (m)
8	CH_2	27.02	0.70 (m, H_α)<br>1.22 (m, H_β)
9	CH_2	39.45	1.51 (ddd, J = 13.0, 4.6, 2.2, H_α)<br>0.89 (ddd, J = 13.7, 13.7, 2.6, H_β)
10	C_q	42.12	–
11	C_q	37.86	–
12	CH_2	26.16	1.29 (m, 2H)
13	CH_2	28.75	1.74 (m, H_α)<br>1.29 (m, H_β)
14	CH	61.74	1.14 (m)
15	CH	29.31	1.60 (m)
16	CH_3	23.35	0.95 (d, J = 6.6)
17	CH_3	23.64	0.95 (d, J = 6.6)
18	CH_3	19.39	0.78 (s)
19	CH_3	19.90	1.01 (d, J = 6.3)
20	CH_3	18.78	0.93 (d, J = 6.4)

[a] Carbon numbering as shown in Figure S10 indicates the origin of each carbon from GGPP by same number. [b] Chemical shifts δ in ppm. Multiplicity: s = singlet, d = doublet, t = triplet, m = multiplet. Coupling constants J are given in Hertz.

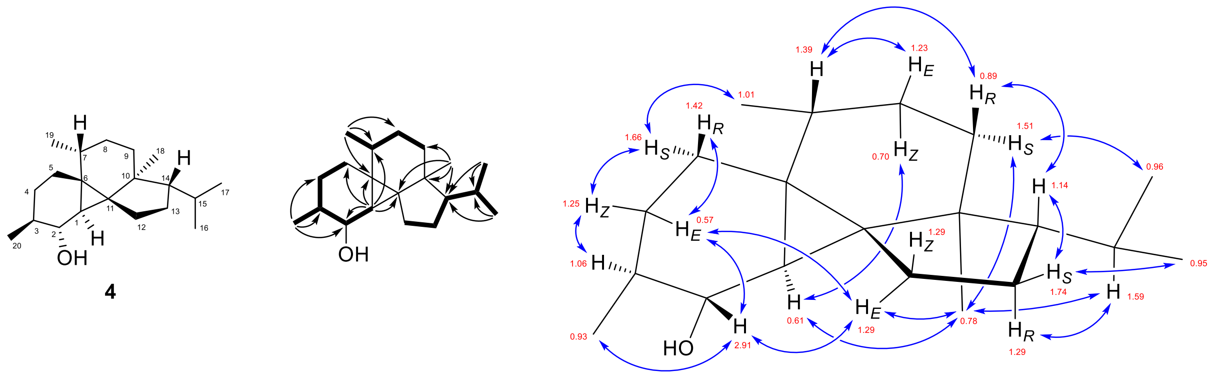

Figure S10. Structure elucidation of peyssonnosol B (**4**). Bold: ^{1}H,^{1}H-COSY, single headed arrows: key HMBC, and blue double headed arrows: NOESY correlations. H_R, H_S, H_E and H_Z indicate the results from stereoselective labelling experiments (Figures S25 and S26).

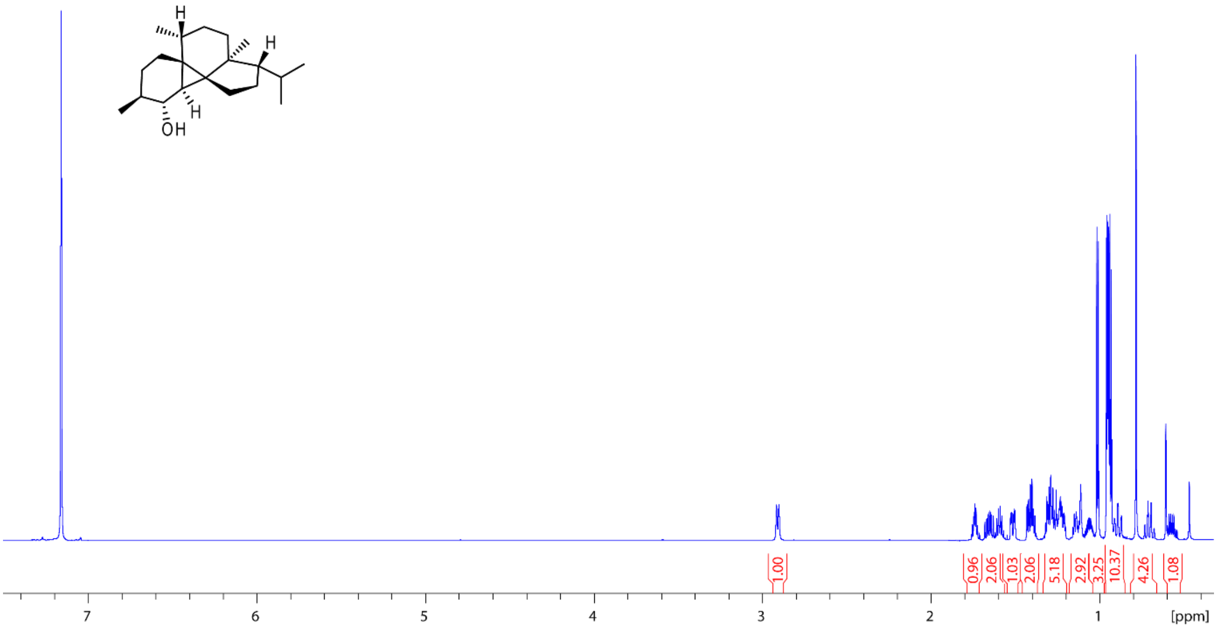

Figure S11. ^{1}H-NMR spectrum of **4** (700 MHz, C_6D_6).

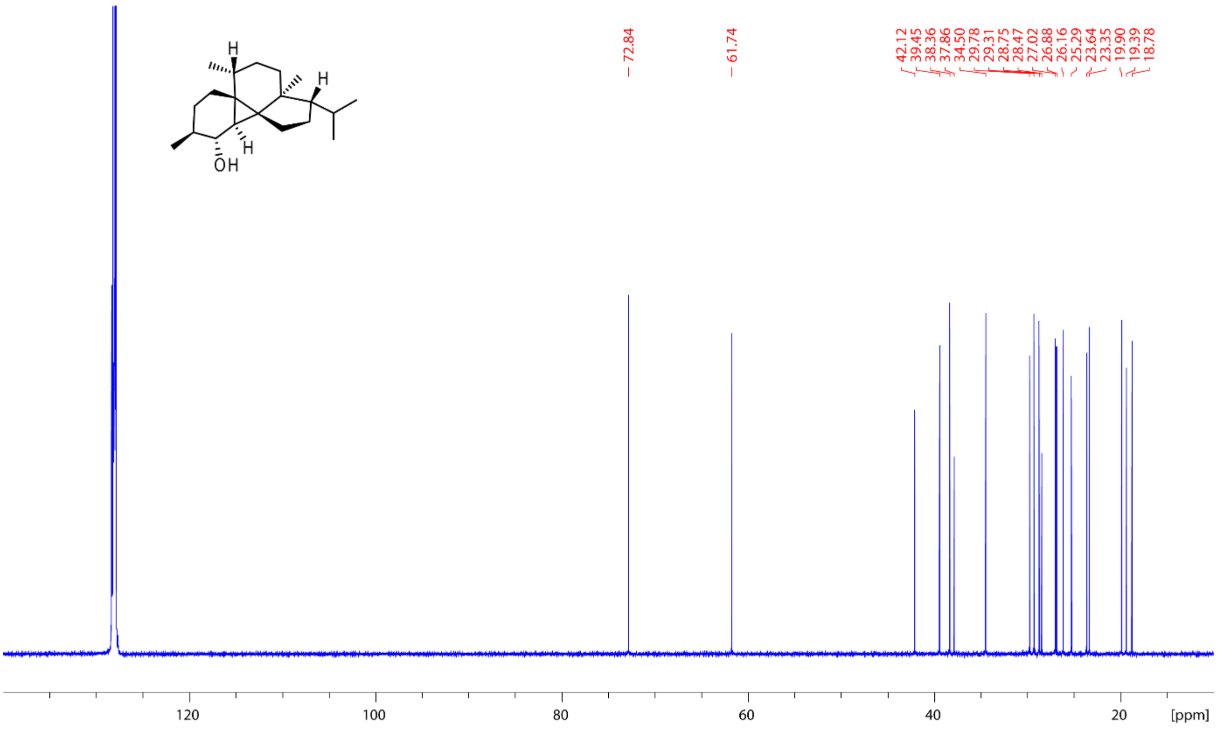

Figure S12. ^{13}C-NMR spectrum of **4** (176 MHz, C_6D_6).

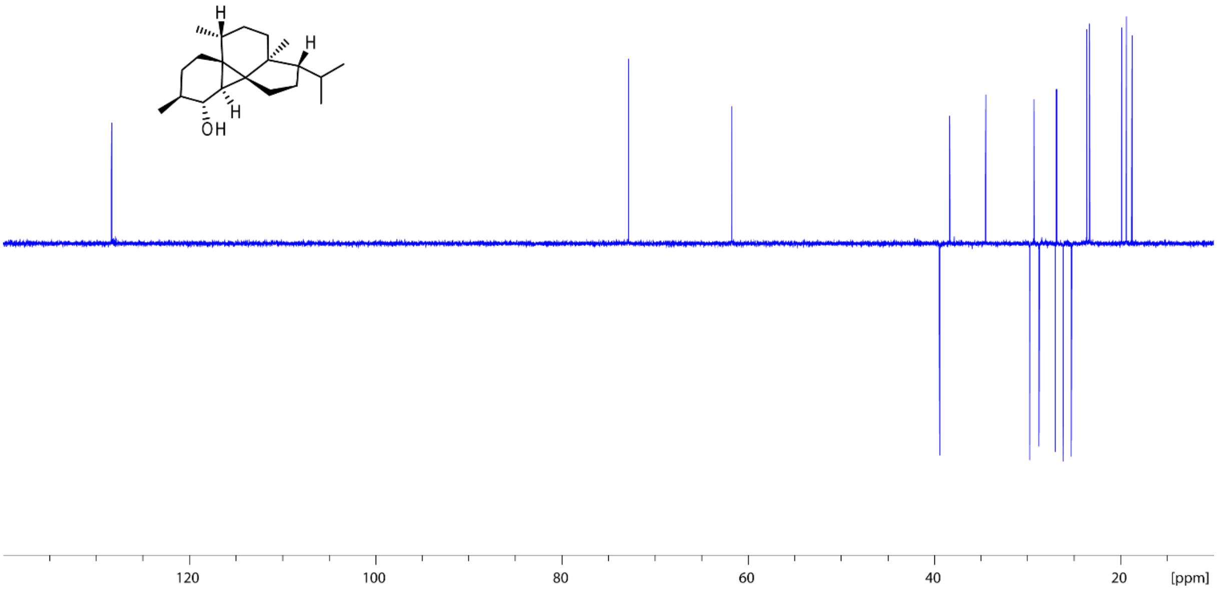

Figure S13. ^{13}C-DEPT135 spectrum of **4** (176 MHz, C_6D_6).

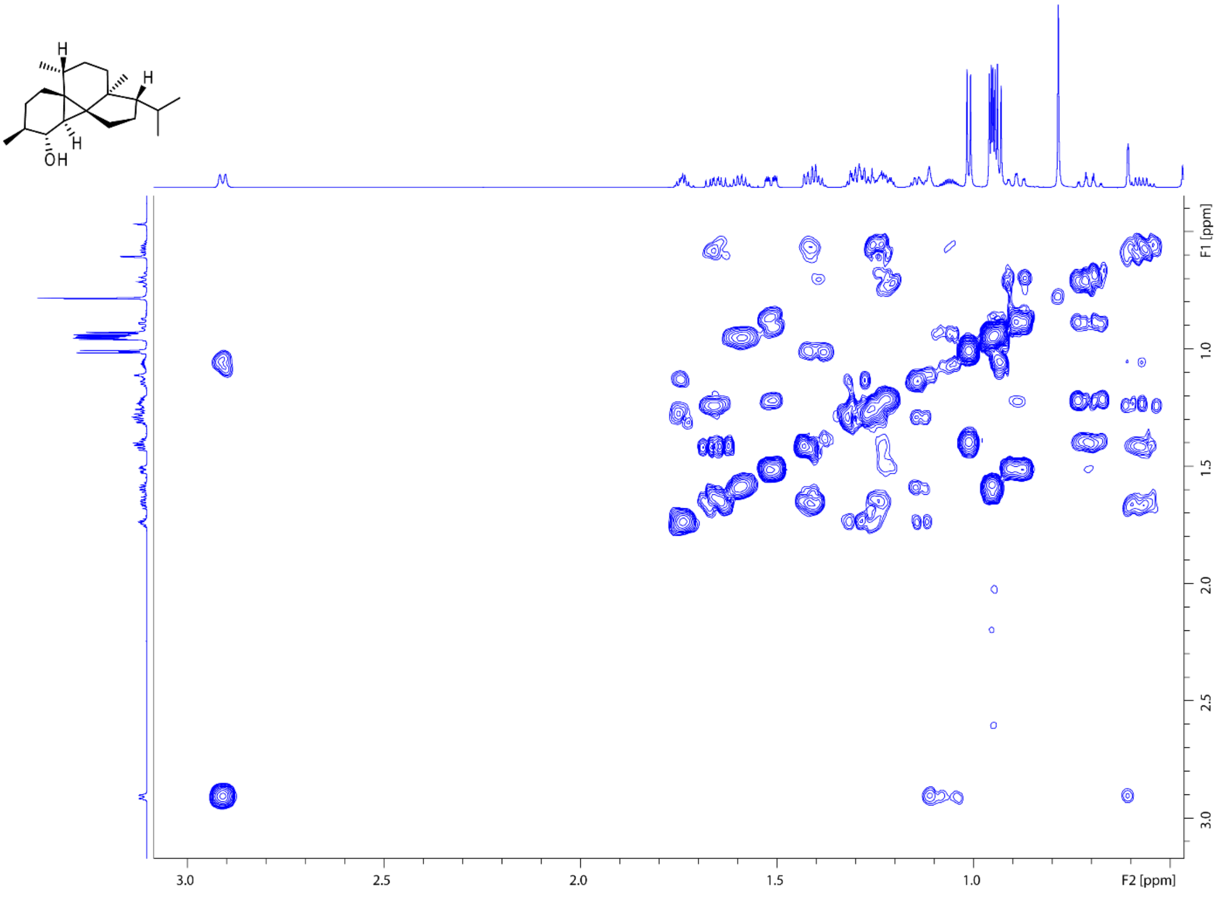

Figure S14. ^{1}H,^{1}H-COSY spectrum of **4** (700 MHz, C_6D_6).

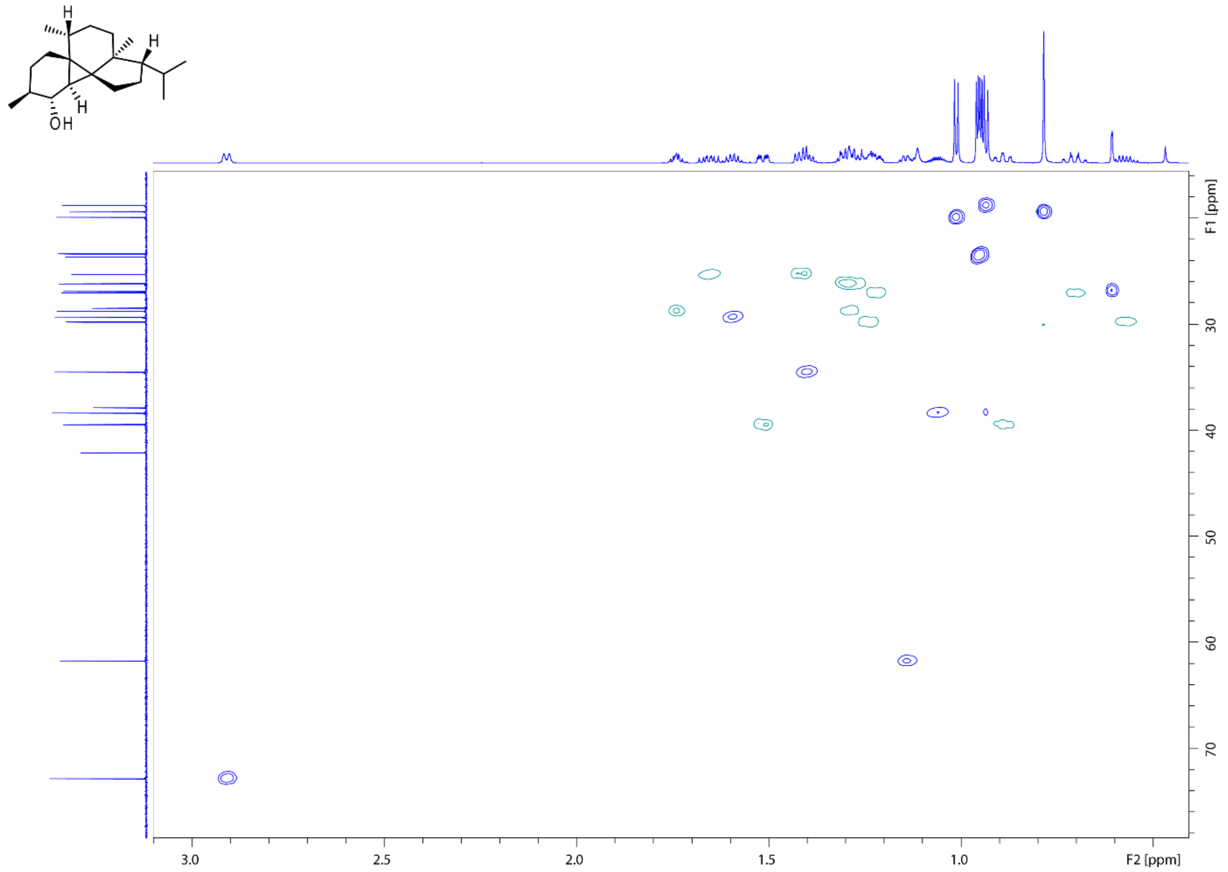

Figure S15. HSQC spectrum of **4** (700 MHz, C_6D_6).

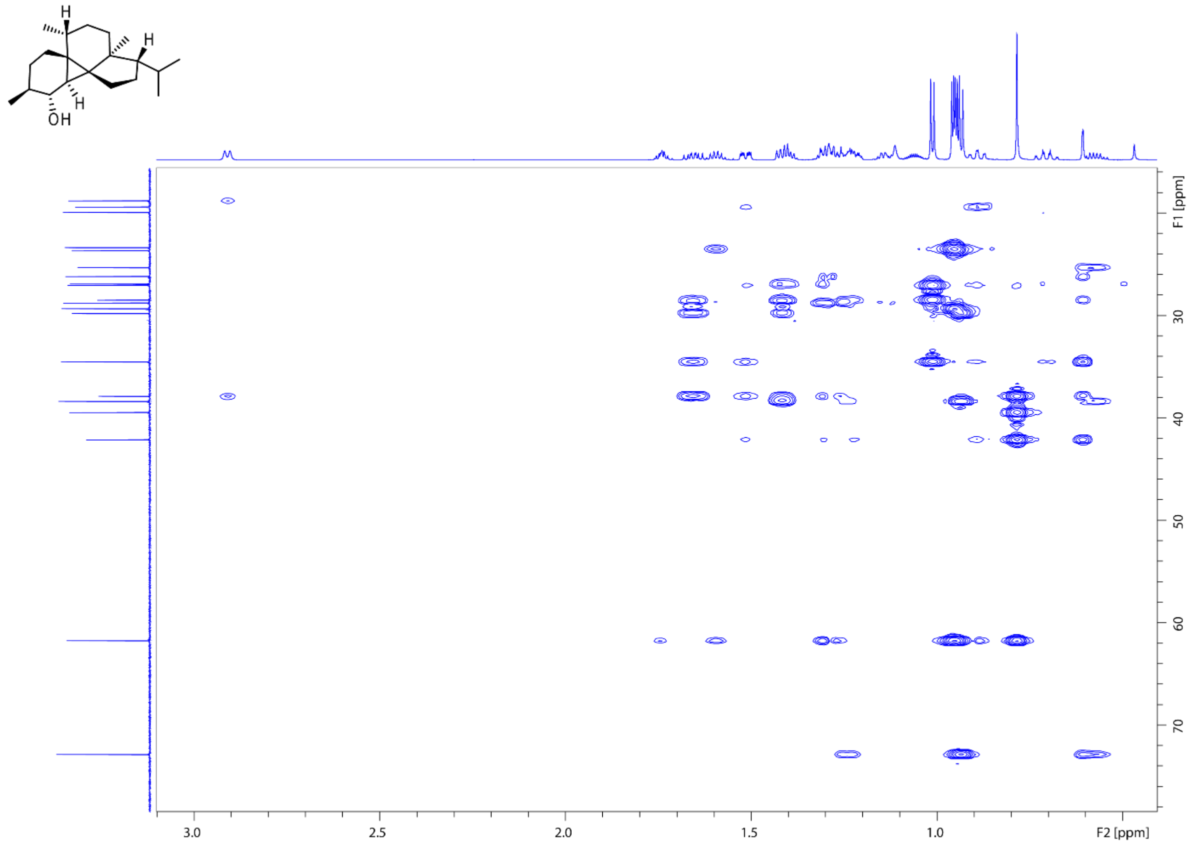

Figure S16. HMBC spectrum of **4** (700 MHz, C_6D_6).

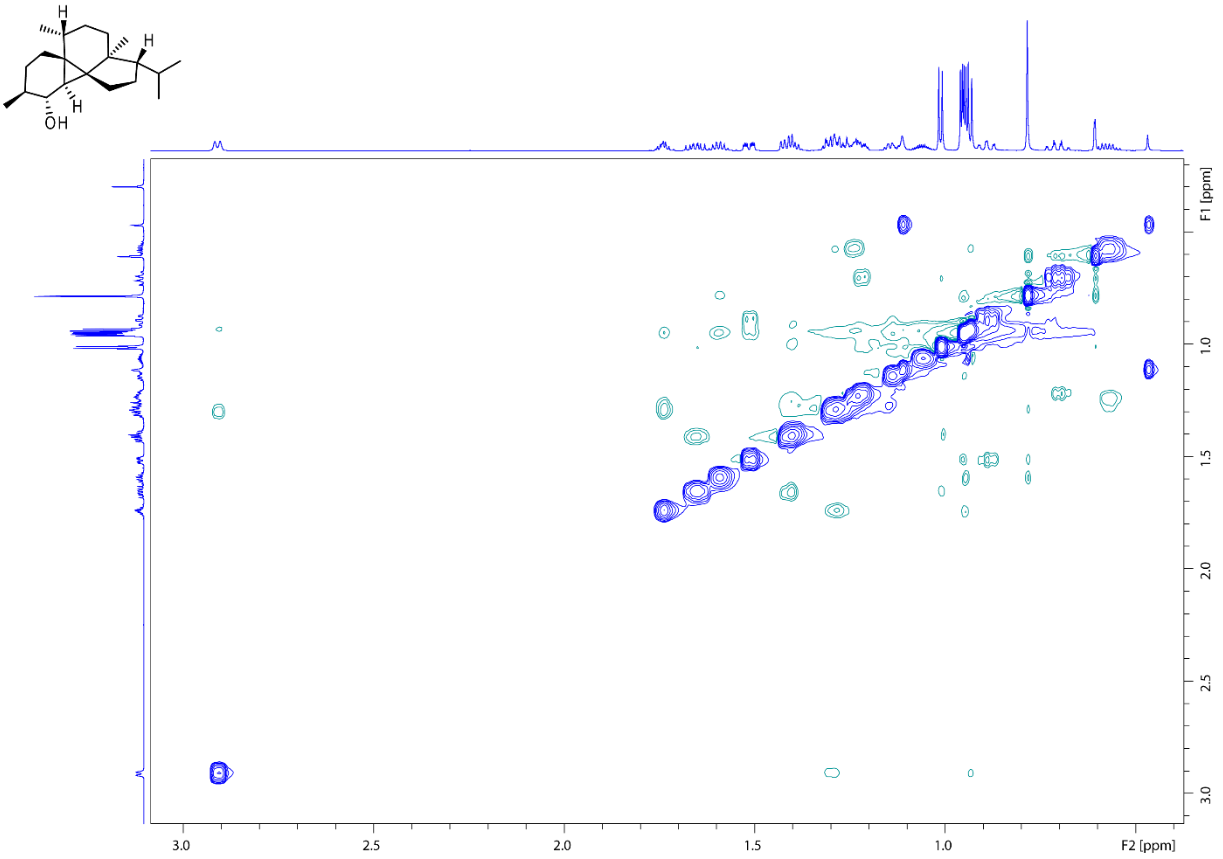

Figure S17. NOESY spectrum of **4** (700 MHz, C_6D_6).

X-ray analysis of 3 and 4

Square single crystals of peyssonnosol (**3**) and peyssonnosol B (**4**) were obtained by recrystallization from chloroform/acetonitrile = 1:1. Suitable crystals were selected and X-ray data were collected using Cu-*K*α-radiation (λ=1.54184) on a XtaLAB AFC12 (RINC): Kappa single diffractometer (for **3**) or on a XtaLAB Synergy R, DW system, HyPix diffractometer (for **4**). The crystals were kept at 111(16) K (for **3**) or at 100.02(10) K (for **4**) during data collection. The structures were solved using Olex2[4] with the SHELXT[5] structure solution program with intrinsic phasing and refined with the SHELXL[6] refinement package using least squares minimisation.

Peyssonnosol (3). Crystal data for $C_{40}H_{70}O_3$ (M =598.96 g/mol): orthorhombic, space group $P2_12_12_1$ (no. 19), a = 6.33009(6) Å, b = 13.37507(17) Å, c = 42.9241(4) Å, V = 3634.19(7) Å^3, Z = 4, T = 100.02(10) K, μ(Cu Kα) = 0.501 mm^{-1}, *Dcalc* = 1.095 g/cm^3, 34564 reflections measured (6.922° ≤ 2Θ ≤ 146.598°), 7108 unique (R_{int} = 0.0329, R_{sigma} = 0.0231) which were used in all calculations. The final R_1 was 0.0323 (I > 2σ(I)) and wR_2 was 0.0800 (all data).

Peyssonnosol B (4). Crystal data for $C_{20}H_{34}O$ (M =290.47 g/mol): tetragonal, space group $P4_3$ (no. 78), a = 15.11180(10) Å, c = 7.92230(10) Å, V = 1809.19(3) Å^3, Z = 4, T = 111(16) K, μ(Cu Kα) = 0.470 mm^{-1}, *Dcalc* = 1.066 g/cm^3, 11332 reflections measured (8.274° ≤ 2Θ ≤ 148.702°), 3479 unique (R_{int} = 0.0214, R_{sigma} = 0.0160) which were used in all calculations. The final R_1 was 0.0302 (I > 2σ(I)) and wR_2 was 0.0810 (all data).

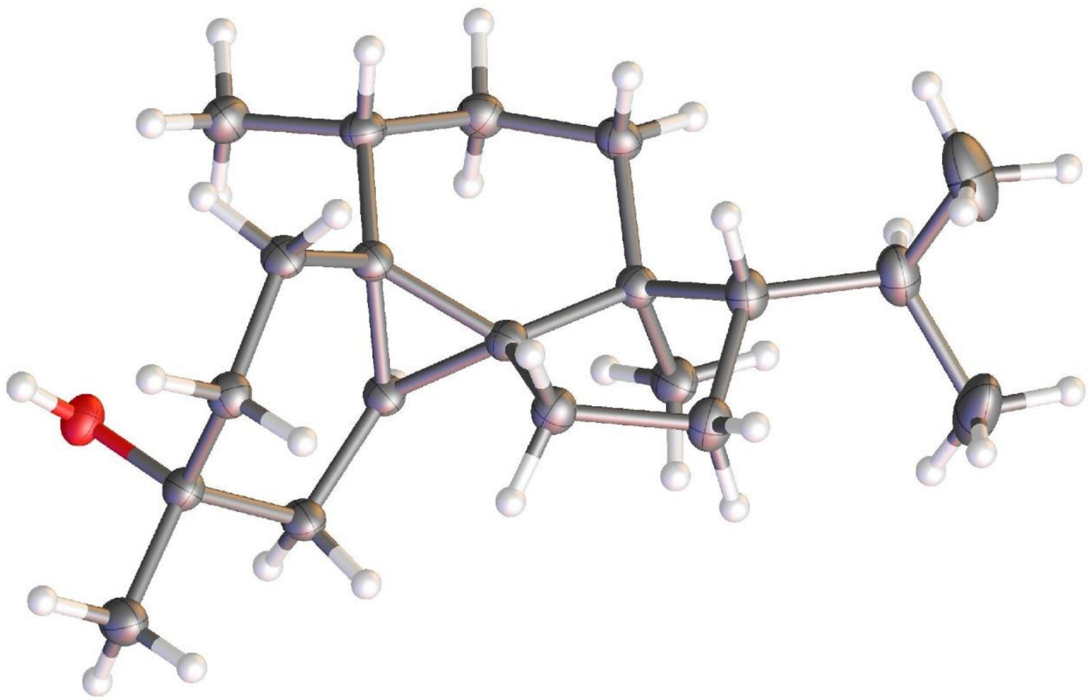

Figure S18. ORTEP illustration of **3**.

Table S4. Crystal data and structure refinement for **3**.

device type	XtaLAB Synergy R, DW system, HyPix
empirical formula	$C_{40}H_{70}O_3$
moiety formula	$2(C_{20}H_{34}O)$, H_2O
formula weight	598.96
temperature / K	100.02(10)
crystal system	orthorhombic
space group	$P2_12_12_1$
a / Å	6.33009(6)
b / Å	13.37507(17)
c / Å	42.9241(4)
α = β = γ / °	90 = 90 = 90
volume / $Å^3$	3634.19(7)
Z	4
ρ_{calc} / g cm^{-3}	1.095
μ / mm^{-1}	0.501
F(000)	1336.0
crystal size/mm^3	0.15 × 0.12 × 0.1
absorption correction	MULTI-SCAN
Tmin; Tmax	0.930; 0.951
radiation	Cu Kα (λ = 1.54184)
2Θ range for data collection	6.922 to 146.598
completeness to Θ	1.70/0.97
index ranges	-4 ≤ h ≤ 7, -16 ≤ k ≤ 14, -52 ≤ l ≤ 53
reflections collected	34564
independent reflections	7108 [R_{int} = 0.0329, R_{sigma} = 0.0231]
data / restraints / parameters	7108/384/406
goodness-of-fit on F^2	1.036
final R indexes [I>=2σ (I)]	R_1 = 0.0323, wR_2 = 0.0788
final R indexes [all data]	R_1 = 0.0337, wR_2 = 0.0800
Largest diff. peak/hole / e $Å^{-3}$	0.17/-0.20
Flack parameter	0.07(6)
CCDC accession no.	2411143

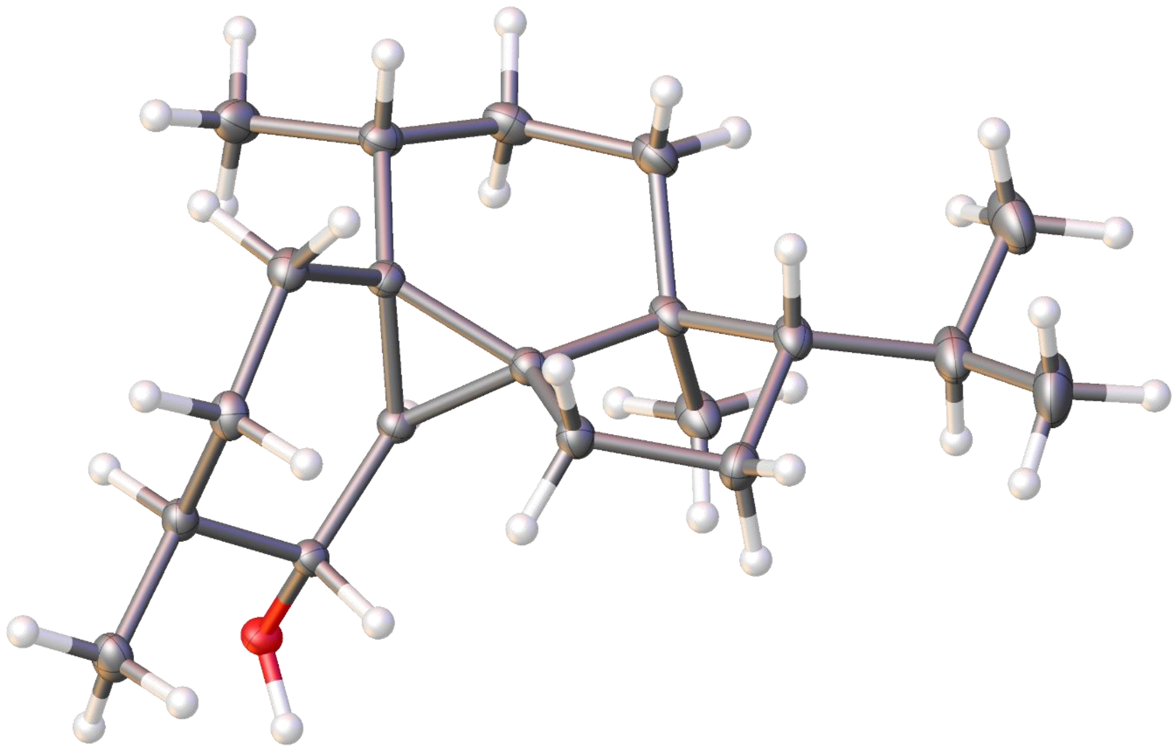

Figure S19. ORTEP illustration of **4**.

Table S5. Crystal data and structure refinement for **4**.

device type	XtaLAB Synergy R, DW system, HyPix
empirical formula	$C_{20}H_{34}O$
moiety formula	$C_{20}H_{34}O$
formula weight	290.47
temperature / K	111(16)
crystal system	tetragonal
space group	$P4_3$
a / Å	15.11180(10)
b / Å	15.11180(10)
c / Å	7.92230(10)
α = β = γ / °	90 = 90 = 90
volume / $Å^3$	1809.19(3)
Z	4
ρ_{calc} / g cm^{-3}	1.066
μ / mm^{-1}	0.470
F(000)	648.0
crystal size/mm^3	0.15 × 0.13 × 0.12
absorption correction	MULTI-SCAN
Tmin; Tmax	0.932; 0.945
radiation	Cu Kα (λ = 1.54184)
2Θ range for data collection	8.274 to 148.702
completeness to Θ	1.75/0.94
index ranges	-17 ≤ h ≤ 18, -18 ≤ k ≤ 16, -9 ≤ l ≤ 9
reflections collected	11332
independent reflections	3479 [R_{int} = 0.0214, R_{sigma} = 0.0160]
data / restraints / parameters	3479/1/199
goodness-of-fit on F^2	1.034
final R indexes [I>=2σ (I)]	R_1 = 0.0302, wR_2 = 0.0807
final R indexes [all data]	R_1 = 0.0304, wR_2 = 0.0810
Largest diff. peak/hole / e $Å^{-3}$	0.14/-0.17
Flack parameter	0.00(9)
CCDC accession no.	2411142

Gene cloning

The *E. coli* codon optimised genes for *AbPS1* and *AbPS2* were synthesised by Beijing Tsingke Biotech Co., Ltd. Primers AbPS1-32a-F/R and AbPS2-C6T-F/R (Table S1) were used to amplify the genes and cloned through homologous recombination in yeast as described above into the pET32a or pMAL-c6T vector, respectively, to obtain plasmids pET32a-AbPS1 and pMALc6T-AbPS2.

Gene expression and protein purification

E. coli BL21 (DE3) cells harboring the expression plasmid (pET32a-AbPS1, pMALc6T-AbPS2) were used to inoculate a starter culture in LB medium (10 mL) supplied with ampicillin (50 μg/mL), which was grown with shaking at 37 °C overnight. The starter culture was used to inoculate the expression culture (1/100 v/v) in LB medium (1 L) with ampicillin and the cells were grown with shaking at 37 °C until OD_{600} = 0.4 – 0.6 was reached. The culture was cooled to 18 °C, before IPTG (0.4 mM final concentration) was added to induce expression. The culture was shaken at the same temperature overnight and then centrifuged (3500 x g, 40 min, 4 °C). For the preparation scale of peyssonnosol and peyssonnosol B synthase, the medium was discarded and the cell pellet was resuspended in binding buffer (10 mL L^{-1} culture; 20 mM Na_2HPO_4, 500 mM NaCl, 20 mM imidazole, 1 mM $MgCl_2$, pH 7.4, 4 °C). The cells were lysed by ultrasonication (10 x 1 min) under ice cooling. The cell debris was spun down (14600 x g, 10 min, 4 °C), the protein solution was filtered with disposable syringe filter (MACHEREY-NAGEL GmbH & Co. KG), and loaded onto a Ni^{2+}-NTA affinity chromatography column (10 mL column volume; Ni-NTA superflow, Qiagen, Venlo, Netherlands). The column was washed with two column volumes of binding buffer (10 mL L^{-1} culture) to elute non-binding proteins, followed by desorption of the target protein from the stationary phase with two column volumes of elution buffer (10 mL L^{-1} culture; 20 mM Na_2HPO_4, 500 mM NaCl, 500 mM imidazole, 1 mM $MgCl_2$, pH 7.4, 4 °C) with fractionation. The fractions were analysed by SDS-PAGE and fractions containing pure protein were pooled (Figure S20) and used for incubation experiments. The protein concentration was determined through Bradford assay[7] and adjusted to 5.0 mg/mL.

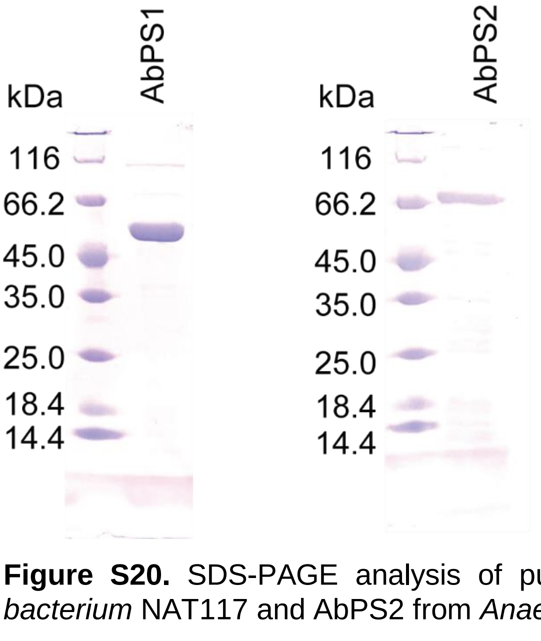

Figure S20. SDS-PAGE analysis of purified recombinant AbPS1 from *Anaerolineaceae bacterium* NAT117 and AbPS2 from *Anaerolineae bacterium* HKST-UBA68.

Small scale incubation of GGPP with recombinant AbPS1 and AbPS2

Analytical scale incubations were performed with the substrates GPP, FPP, GGPP and GFPP (0.5 mg each) dissolved in substrate buffer (0.1 mL, 25 mM NH_4HCO_3). After dilution with incubation buffer (0.8 mL, 50 mM Tris, 10 mM $MgCl_2$, 20% glycerol, pH = 7.4), enzyme elution fraction (0.1 mL, containing 5.0 mg mL^{-1} enzyme) was added. The reaction mixture was incubated at 30 °C with shaking for 15 h, followed by extraction with n-hexane (200 μL). The organic layer was dried with $MgSO_4$ and analysed by GC/MS (Figures S21 and S22).

Preparative scale incubations with recombinant AbPS1 and AbPS2 and compound isolation

For preparative scale incubations, GGPP (50 mg, 111 μmol) in NH_4HCO_3 (25 mM, 5 mL) and an enzyme preparation of AbPS1 or AbPS2 (5 mL; from 2 L expression culture, 5.0 mg mL^{-1}) were added to incubation buffer (40 mL). The reaction mixture was stirred overnight at 30 °C and then extracted with Et_2O (3 x 15 mL). The combined extracts were dried with $MgSO_4$ and concentrated in vacuo. Peyssonnosol (**3**, 5.0 mg, 17.2 μmol, 15 %) was obtained through column chromatography on silica gel with petroleum ether and EtOAc (3 : 1) from the AbPS1 enzyme reaction, and peyssonnosol B (**4**, 5.8 mg, 20.0 μmol, 18 %) was isolated by column chromatography on silica gel with petroleum ether and EtOAc (3 : 1) from the AbPS2 enzyme reaction.

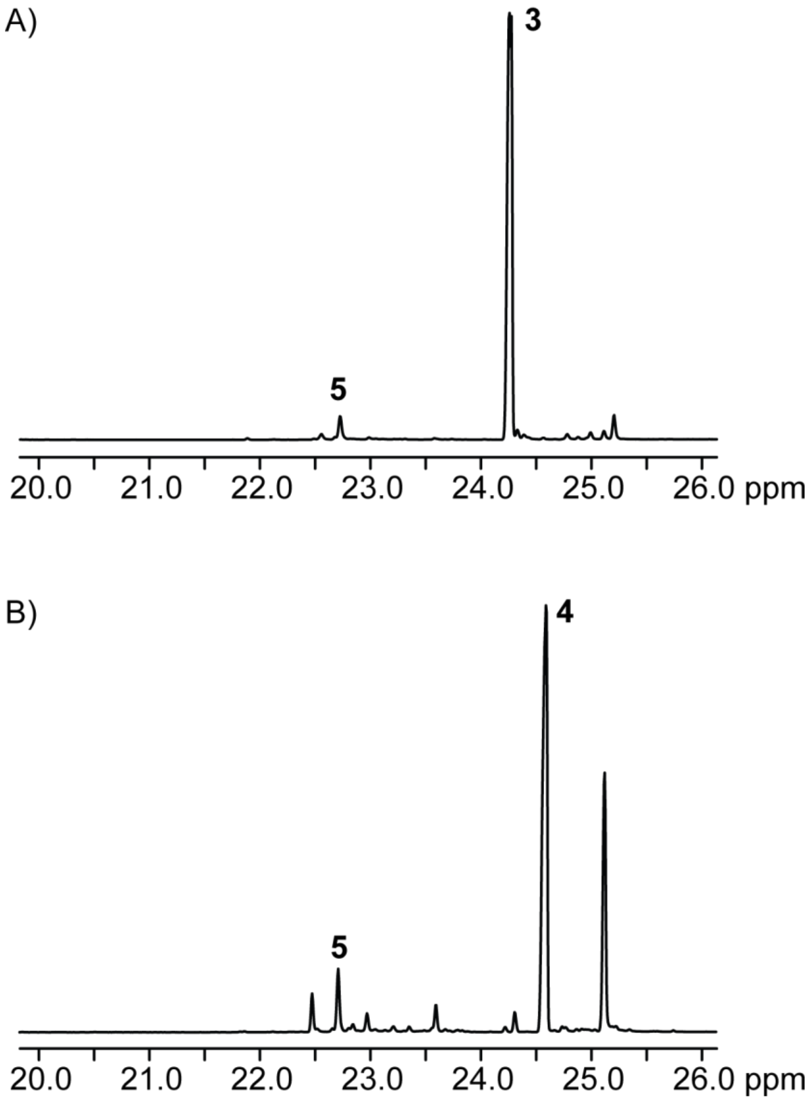

Figure S21. Total ion chromatograms of extracts from enzyme incubations of A) GGPP with AbPS1, and B) GGPP with AbPS2. No products were obtained from incubations with GPP, FPP, and GFPP.

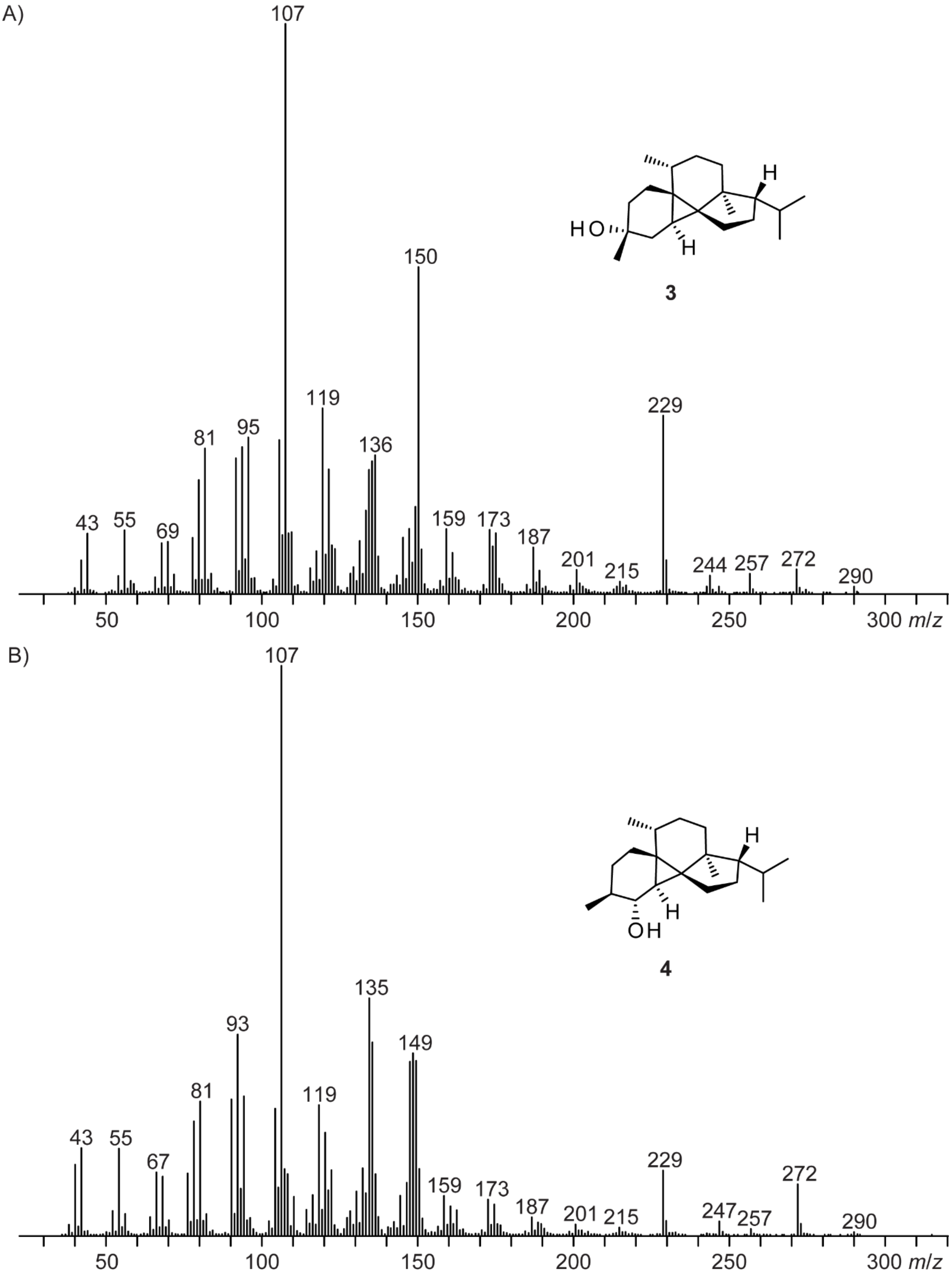

Figure S22. EI mass spectra of A) peyssonnosol (**3**) and B) peyssonnosol B (**4**).

Incubation experiments with labelled substrates

Isotopic labelling experiments were performed with the precursors of GGPP (ca. 1.0 mg, in 100 µL 25 mM NH_4HCO_3), incubation buffer (850 µL) and preparations of purified enzymes (each 50 µL) as listed in Table S6. After incubation at 30 °C overnight, the products were extracted with C_6D_6 (600 µL) or n-hexane (200 µL), then the extracts were dried with $MgSO_4$ and analysed by NMR and/or GC/MS.

Table S6. Labelling experiments with AbPS1 and AbPS2.

entry	substrates	enzymes	results shown in
1	DMAPP + (*E*)-(4-^{13}C,4-^{2}H)IPP[8]	GGPPS,[9] AbPS1	Figure S23
2	DMAPP + (*Z*)-(4-^{13}C,4-^{2}H)IPP[8]	GGPPS, AbPS1	Figure S23
3	(*R*)-(1-^{13}C,1-^{2}H)IPP[10]	IDI,[11] GGPPS, AbPS1	Figure S24
4	(*S*)-(1-^{13}C,1-^{2}H)IPP[10]	IDI, GGPPS, AbPS1	Figure S24
5	DMAPP + (*E*)-(4-^{13}C,4-^{2}H)IPP	GGPPS, AbPS2	Figure S25
6	DMAPP + (*Z*)-(4-^{13}C,4-^{2}H)IPP	GGPPS, AbPS2	Figure S25
7	(*R*)-(1-^{13}C,1-^{2}H)IPP	IDI, GGPPS, AbPS2	Figure S26
8	(*S*)-(1-^{13}C,1-^{2}H)IPP	IDI, GGPPS, AbPS2	Figure S26
9	(*R*)-GLPP[12]	AbPS1	Figure S27
10	(*S*)-GLPP[12]	AbPS1	Figure S27
11	(1-^{13}C)-GGPP[13]	AbPS1	Figure S28
12	(2-^{13}C)-GGPP[14]	AbPS1	Figure S28
13	(3-^{13}C)-GGPP[13]	AbPS1	Figure S28
14	(4-^{13}C)-GGPP[13]	AbPS1	Figure S28
15	(1-^{13}C)FPP[15] + IPP	GGPPS, AbPS1	Figure S28
16	(2-^{13}C)FPP[15] + IPP	GGPPS, AbPS1	Figure S28
17	(3-^{13}C)FPP[15] + IPP	GGPPS, AbPS1	Figure S28
18	(4-^{13}C)FPP[15] + IPP	GGPPS, AbPS1	Figure S28
19	(5-^{13}C)FPP[15] + IPP	GGPPS, AbPS1	Figure S28
20	(6-^{13}C)FPP[15] + IPP	GGPPS, AbPS1	Figure S28
21	(3-^{13}C)GPP[16] + IPP	GGPPS, AbPS1	Figure S29
22	(8-^{13}C)FPP[15] + IPP	GGPPS, AbPS1	Figure S29
23	(5-^{13}C)GPP[17] + IPP	GGPPS, AbPS1	Figure S29
24	(10-^{13}C)FPP[15] + IPP	GGPPS, AbPS1	Figure S29
25	(11-^{13}C)FPP[15] + IPP	GGPPS, AbPS1	Figure S29
26	(12-^{13}C)FPP[15] + IPP	GGPPS, AbPS1	Figure S29
27	(9-^{13}C)GPP[18] + IPP	GGPPS, AbPS1	Figure S29
28	(14-^{13}C)FPP[15] + IPP	GGPPS, AbPS1	Figure S29
29	(15-^{13}C)FPP[15] + IPP	GGPPS, AbPS1	Figure S29
30	(20-^{13}C)-GGPP[13]	AbPS1	Figure S29
31	sodium (1-^{13}C)acetate	AbPS2	Figure S30
32	(3-^{13}C,2-^{2}H)DMAPP[19] + IPP	GGPPS, AbPS1	Figure S31
33	(2-^{13}C)DMAPP[20] + (2,2-^{2}H)IPP	GGPPS, AbPS1	Figure S32
34	FPP + (*R*)-(1-^{13}C,1 -^{2}H)IPP	GGPPS, AbPS1	Figure S33
35	FPP + (*S*)-(1-^{13}C,1 -^{2}H)IPP	GGPPS, AbPS1	Figure S33
36	(2-^{13}C)-GGPP[14] + D_2O	AbPS1	Figure S34
37	FPP + (2-^{13}C,1,1-^{2}H)DMAPP[21]	IDI, GGPPS, AbPS1	Figure S35
38	(3-^{13}C,2-^{2}H)FPP[22] + IPP	GGPPS, AbPS1	Figure S36
39	DMAPP + (*E*)-(4-^{13}C,4-^{2}H)IPP	GGPPS, AbPS1-T182A	Figure S60
40	DMAPP + (*Z*)-(4-^{13}C,4-^{2}H)IPP	GGPPS, AbPS1-T182A	Figure S60
41	(*R*)-(1-^{13}C,1-^{2}H)IPP	IDI, GGPPS, AbPS1-T182A	Figures S61 and S62
42	(*S*)-(1-^{13}C,1-^{2}H)IPP	IDI, GGPPS, AbPS1-T182A	Figures S61 and S62
43	DMAPP + (*E*)-(4-^{13}C,4-^{2}H)IPP	GGPPS, AbPS2-L56V/F75A	Figure S71
44	DMAPP + (*Z*)-(4-^{13}C,4-^{2}H)IPP	GGPPS, AbPS2-L56V/F75A	Figure S71

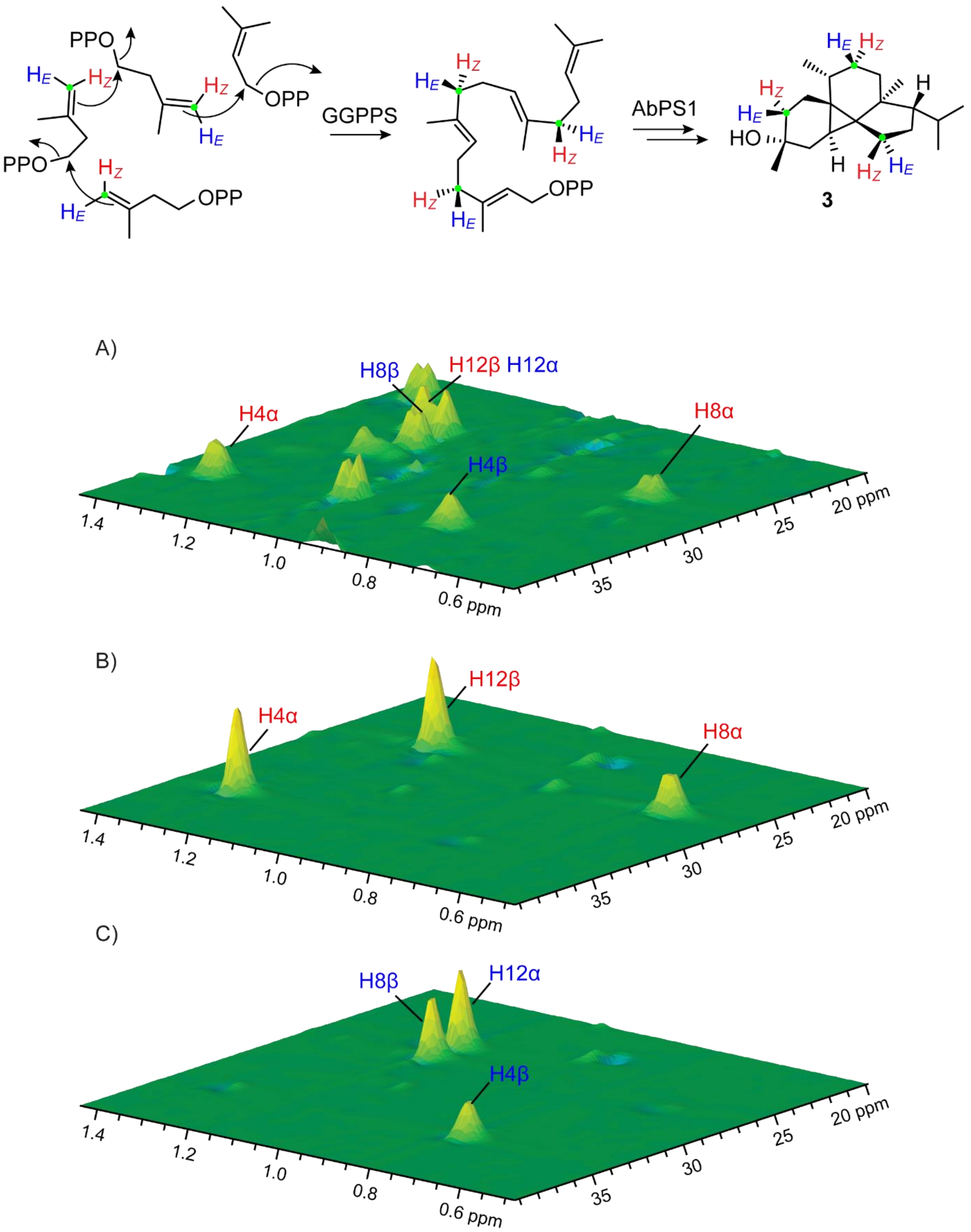

Figure S23. The absolute configuration of **3**. Partial HSQC spectra of A) unlabelled **3**, B) labelled **3** obtained from DMAPP and (*E*)-(4-^{13}C,4-^{2}H)IPP (blue H = ^{2}H) incubated with GGPPS and AbPS1 and C) labelled **3** obtained from DMAPP and (*Z*)-(4-^{13}C,4-^{2}H)IPP (red H = ^{2}H) incubated with GGPPS and AbPS1. The specific incorporation at C4, C8 and C12 with known configuration at these carbons in experiments B) and C) together with the NOESY based assignments of relative orientations of H4α, H4β, H8α, H8β, H12α and H12β (Figure S2) with respect to the naturally present stereogenic centers in **3** allows to assign the shown absolute configuration. Green dots represent ^{13}C-labelled carbons.

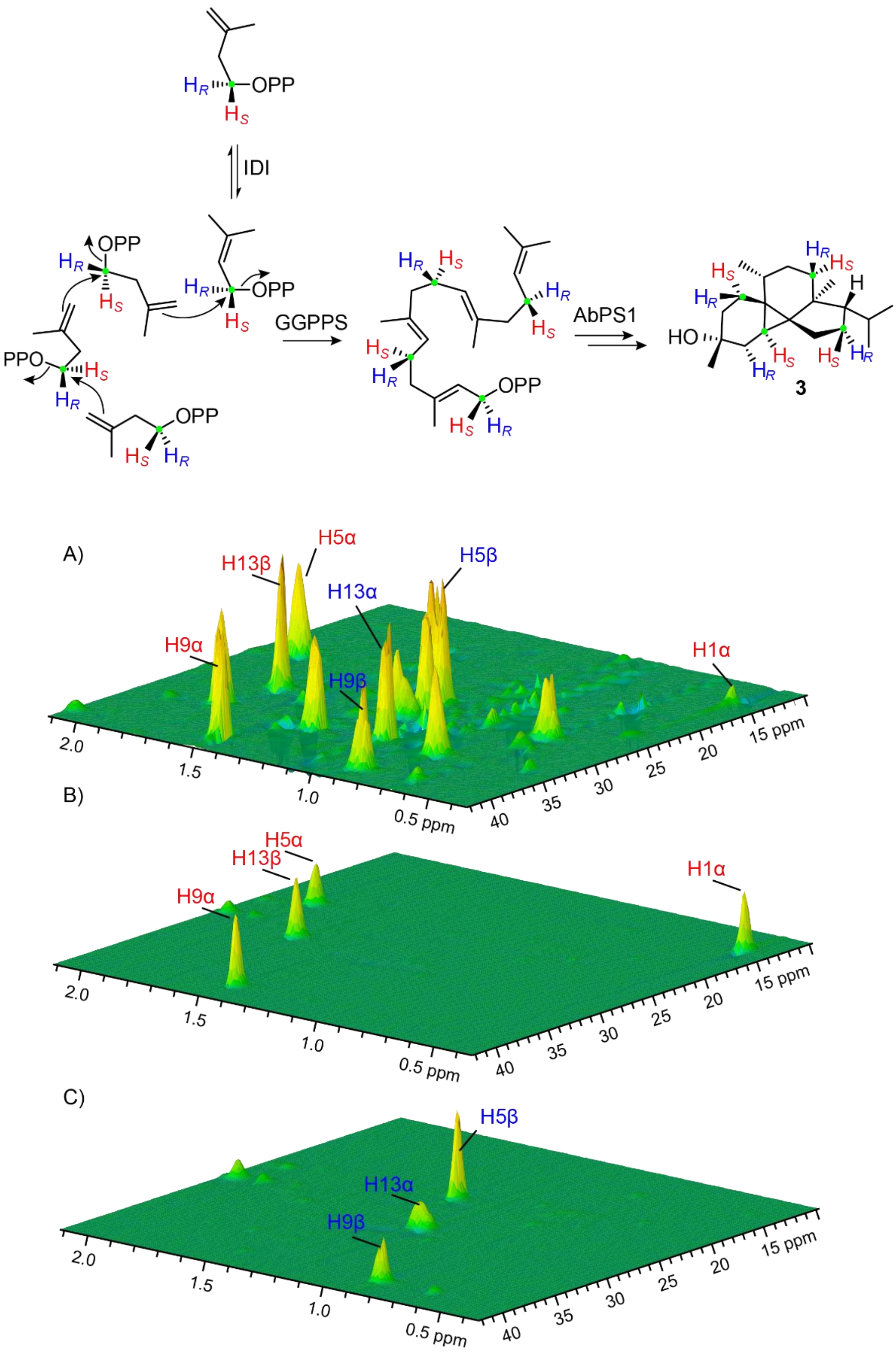

Figure S24. The absolute configuration of **3**. Partial HSQC spectra of A) unlabelled **3**, B) labelled **3** obtained from (*R*)-(1-^{13}C,1-^{2}H)IPP (blue H = ^{2}H) incubated with IDI, GGPPS and AbPS1 and C) labelled **3** obtained from (*S*)-(1-^{13}C,1-^{2}H)IPP (red H = ^{2}H) incubated with IDI, GGPPS and AbPS1. The specific incorporation at C5, C9 and C13 with known configuration at these carbons in experiments B) and C) together with the NOESY based assignments of relative orientations of H5α, H5β, H9α, H9β, H13α and H13β (Figure S2) with respect to the naturally present stereogenic centers in **3** allows to assign the shown absolute configuration. Green dots represent ^{13}C-labelled carbons.

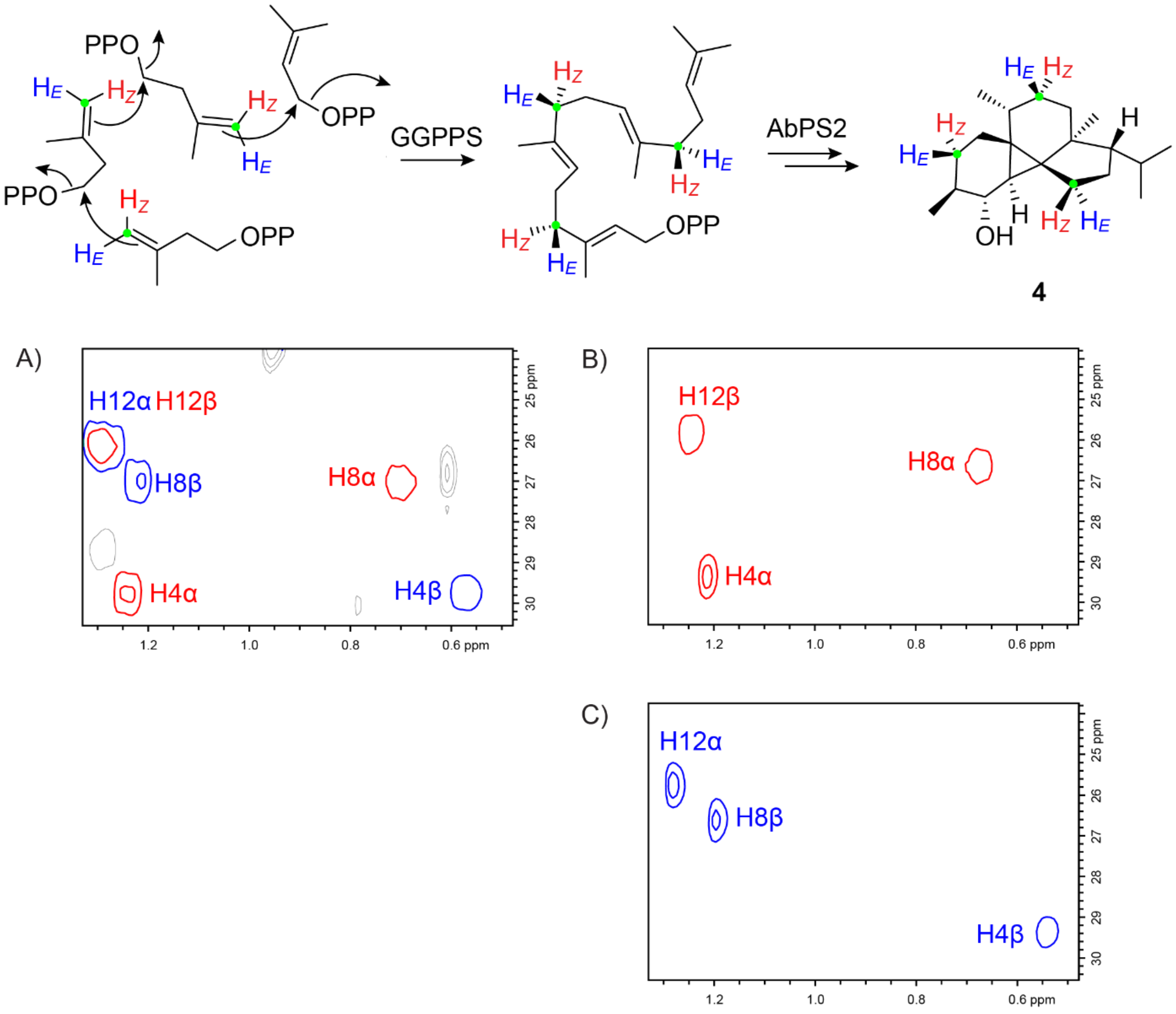

Figure S25. The absolute configuration of **4**. Partial HSQC spectra of A) unlabelled **4**, B) labelled **4** obtained from DMAPP and (*E*)-(4-^{13}C,4-^{2}H)IPP (blue H = ^{2}H) incubated with GGPPS and AbPS2 and C) labelled **4** obtained from DMAPP and (*Z*)-(4-^{13}C,4-^{2}H)IPP (red H = ^{2}H) incubated with GGPPS and AbPS2. The specific incorporation at C4, C8 and C12 with known configuration at these carbons in experiments B) and C) together with the NOESY based assignments of relative orientations of H4α, H4β, H8α, H8β, H12α and H12β (Figure S10) with respect to the naturally present stereogenic centers in **4** allows to assign the shown absolute configuration. Green dots represent ^{13}C-labelled carbons.

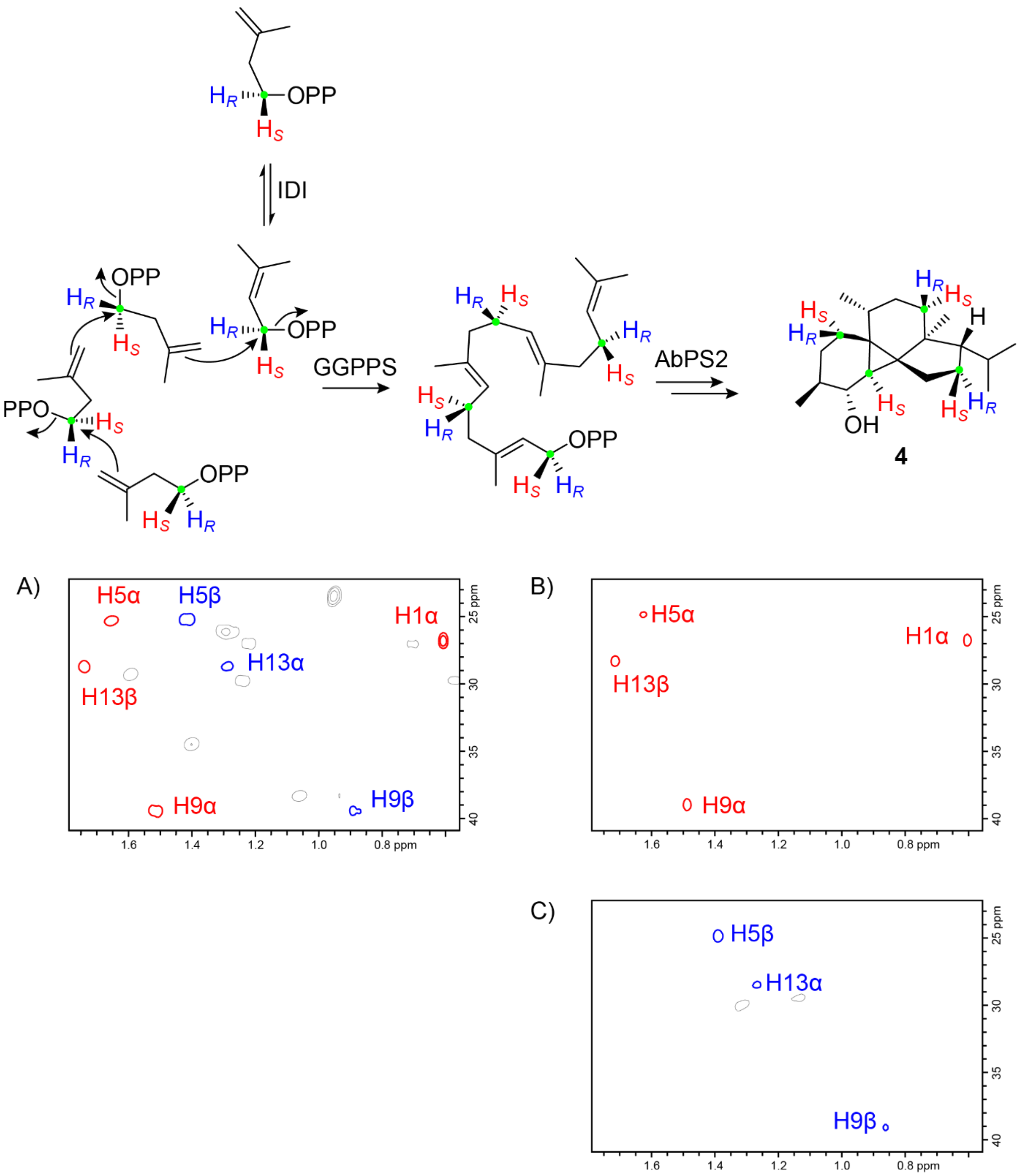

Figure S26. The absolute configuration of **4**. Partial HSQC spectra of A) unlabelled **4**, B) labelled **4** obtained from (*R*)-(1-^{13}C,1-^{2}H)IPP (blue H = ^{2}H) incubated with IDI, GGPPS and AbPS2 and C) labelled **4** obtained from (*S*)-(1-^{13}C,1-^{2}H)IPP (red H = ^{2}H) incubated with IDI, GGPPS and AbPS2. The specific incorporation at C5, C9 and C13 with known configuration at these carbons in experiments B) and C) together with the NOESY based assignments of relative orientations of H5α, H5β, H9α, H9β, H13α and H13β (Figure S10) with respect to the naturally present stereogenic centers in **4** allows to assign the shown absolute configuration. Green dots represent ^{13}C-labelled carbons.

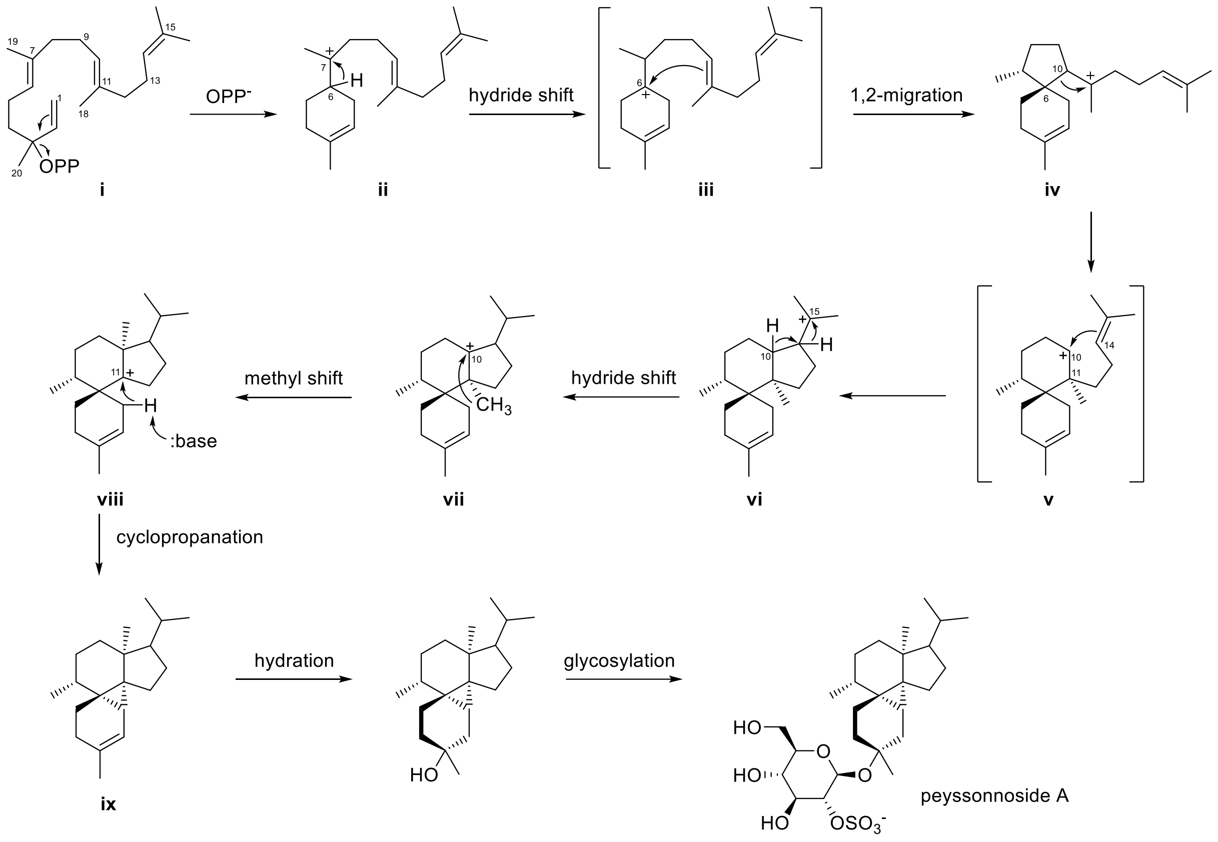

Scheme S1. Biosynthetic proposal by Kubanek.[23]

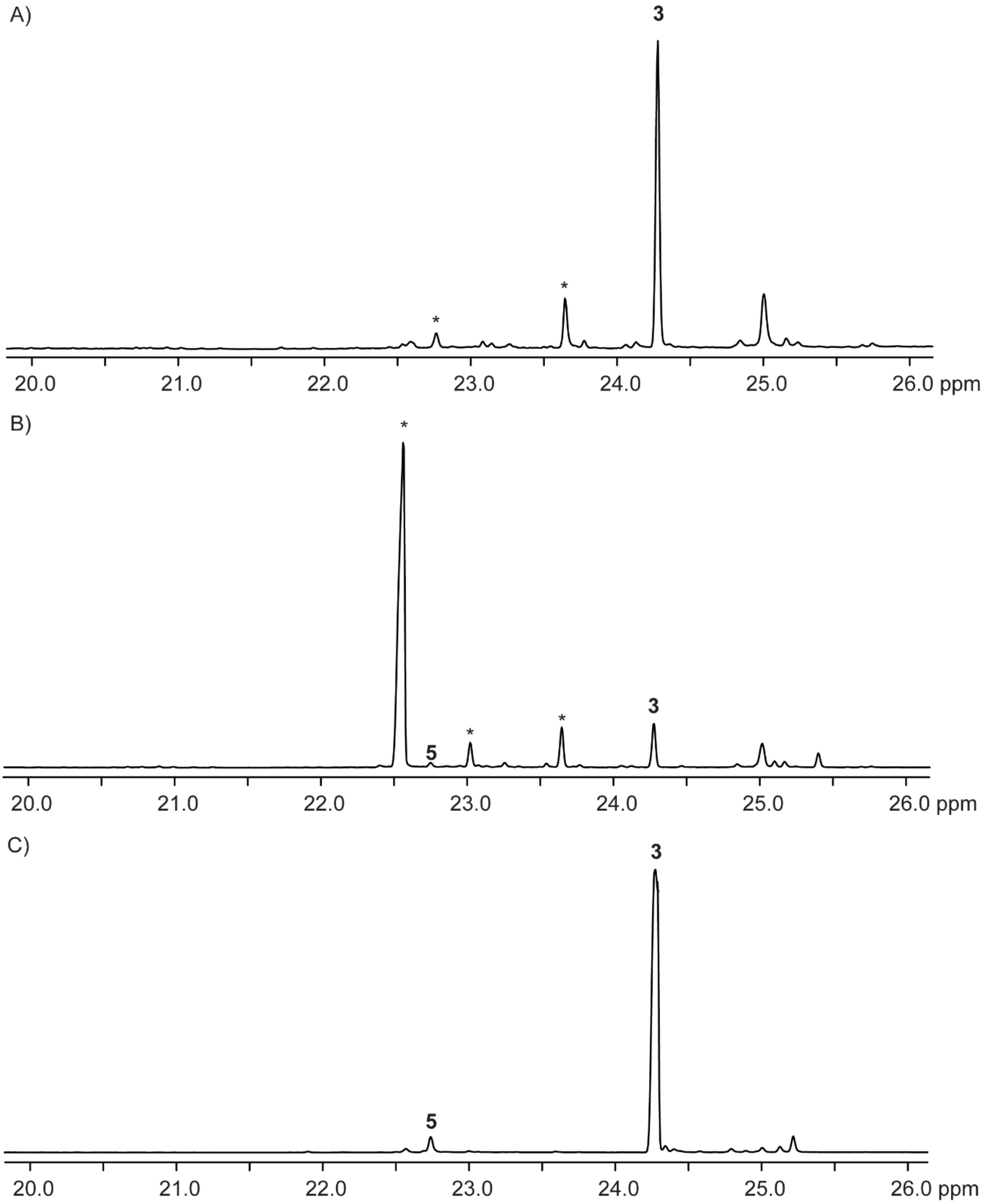

Figure S27. Total ion chromatogram of an extract from an enzyme incubation of A) (*R*)-GLPP with AbPS1; B) (*S*)-GLPP with AbPS1 and C) GGPP with AbPS1. Asterisks indicate lysis and hydrolysis products of the substrates that are also spontaneously formed in the presence of Mg^{2+}.

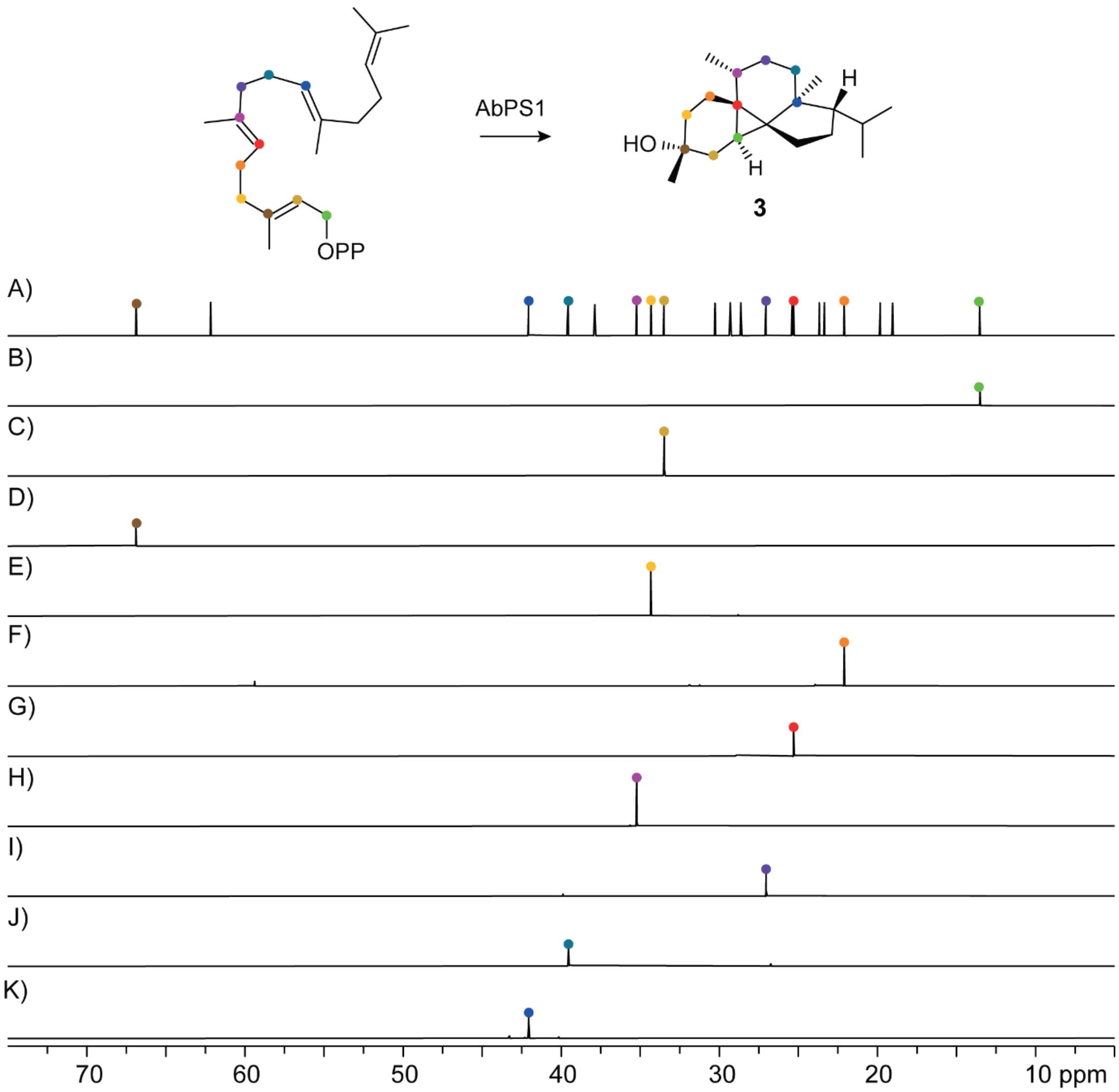

Figure S28. ^{13}C-NMR spectra of A) unlabelled peyssonnosol (**3**), and B) – K) the products obtained with AbPS1 from (1-^{13}C)GGPP – (10-^{13}C)GGPP. The coloured dots show the site of incorporation into **3** and indicate the corresponding signal in the ^{13}C-NMR spectra.

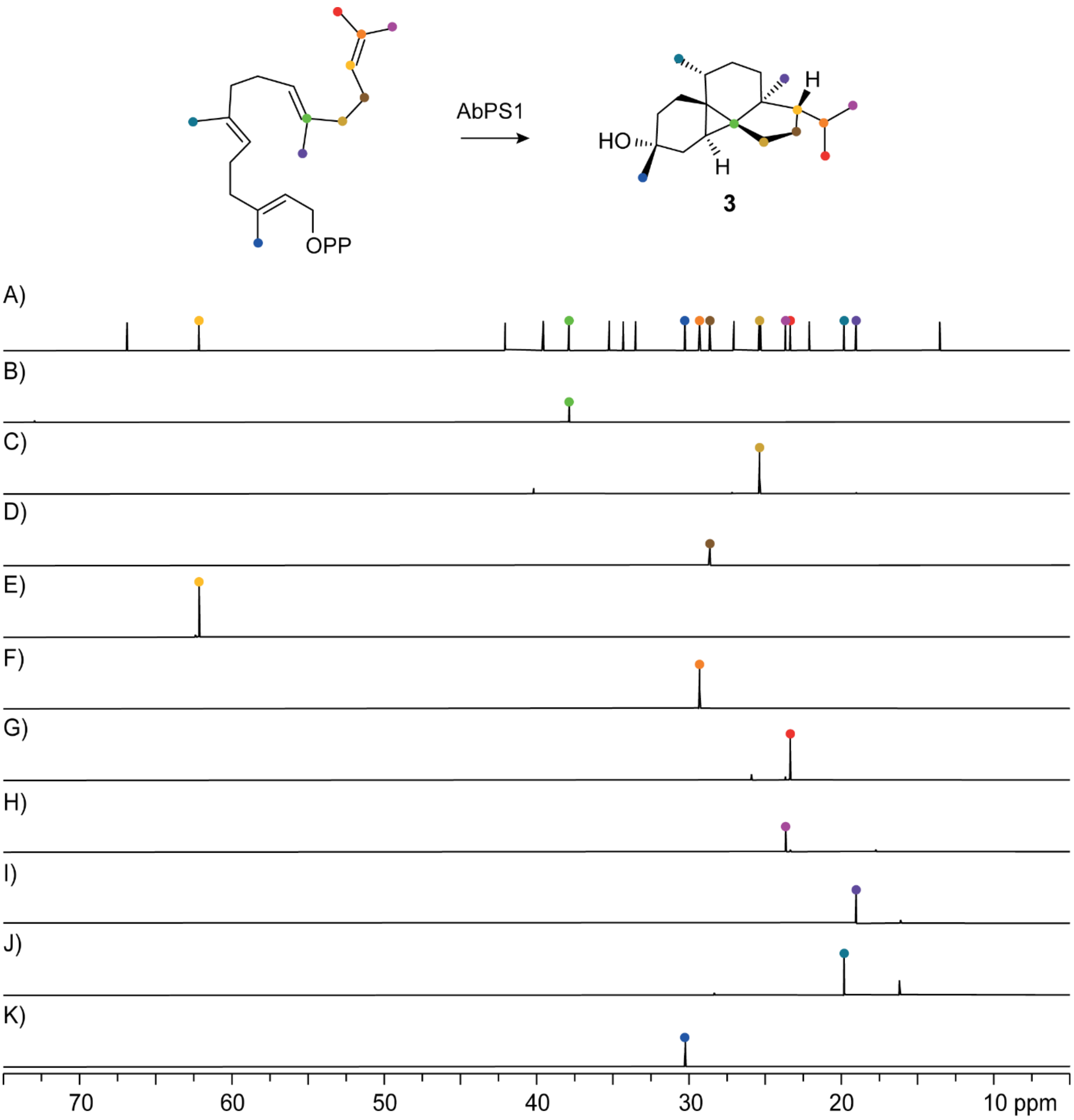

Figure S29. ^{13}C-NMR spectra of A) unlabelled peyssonnosol (**3**), and B) – K) the products obtained with AbPS1 from (11-^{13}C)GGPP – (20-^{13}C)GGPP. The coloured dots show the site of incorporation into **3** and indicate the corresponding signal in the ^{13}C-NMR spectra.

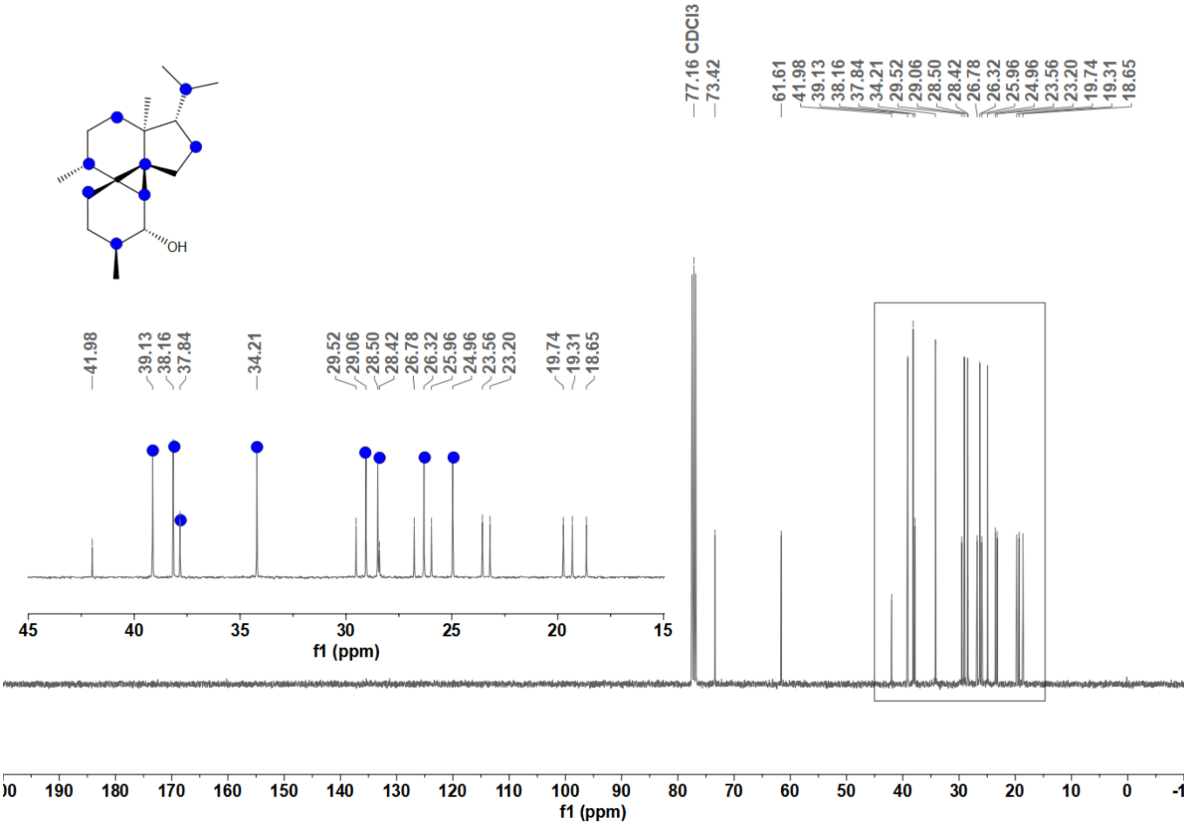

Figure S30. The biosynthetic origin of the skeleton of **4**. The ^{13}C-NMR spectrum ($CDCl_3$, 101 MHz) after feeding of sodium (1-^{13}C)acetate to the *S. cerevisiae* production strain for **4** is in agreement with the same skeletal origin as for **3**.

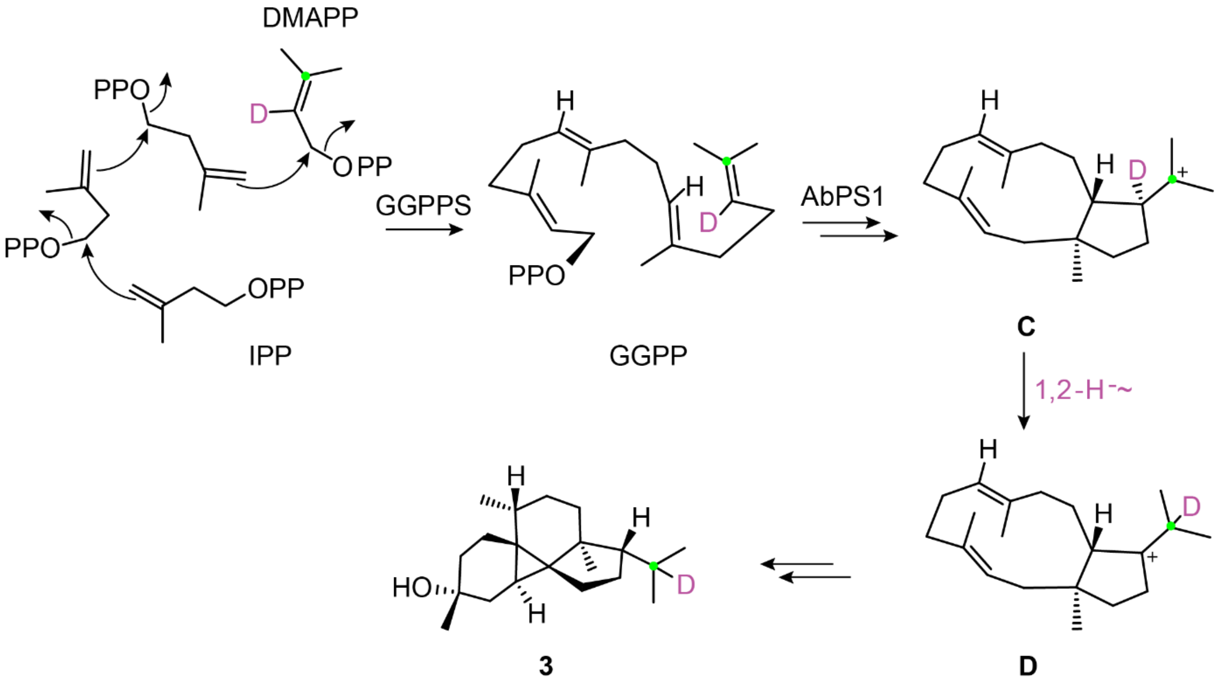

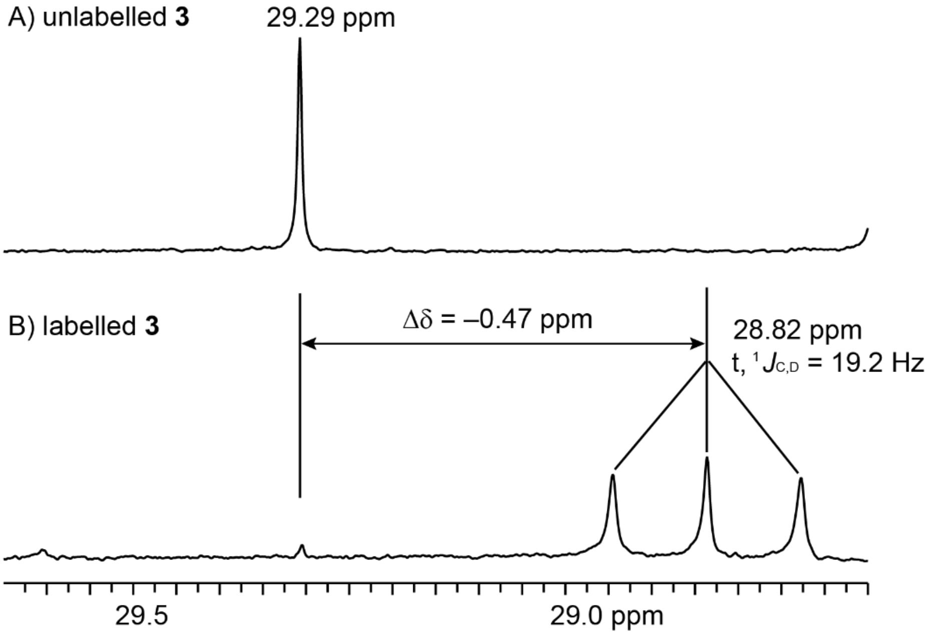

Figure S31. The 1,2-hydride shift from **C** to **D** in the formation of **3**. A) ^{13}C-NMR signal of C15 of unlabelled **3**. B) ^{13}C-NMR signal for C15 of labelled **3** obtained from (3-^{13}C,2-^{2}H)DMAPP and IPP with GGPPS and AbPS1. Green dots represent ^{13}C-labelled carbons. The slightly upfield shifted triplet in B) is indicative for a direct ^{13}C-^{2}H bond and supports the proposed 1,2-hydride shift.

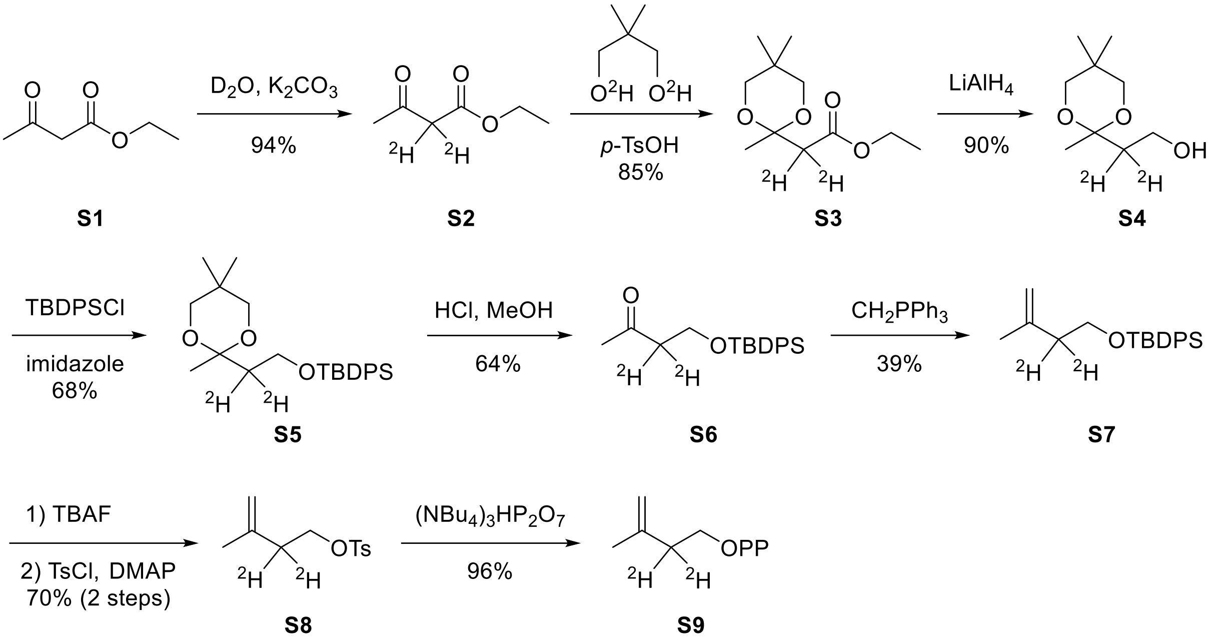

Scheme S2. Synthesis of (2,2-$^2H_2$)IPP.

Synthesis of (2,2-$^2H_2$)IPP[24]

Synthesis of ethyl (2,2-$^2H_2$)acetoacetate (S2)[25]
Ethyl acetoacetate **S1** (2.11 g, 16.2 mmol, 1.0 eq) was dissolved in potassium carbonate solution (5% in D_2O, 10 mL) and the mixture was stirred vigorously for 2 min. The mixture was extracted with diethyl ether, the organic phase was dried with $MgSO_4$ and evaporated. The stirring and extraction were repeated four times until full deuteration (98% by ^{1}H NMR) was achieved. The product **S2** was obtained as a colourless oil (1.99 g, 15.1 mmol, 94%). GC (HP5-MS): *I* = 945. ^{1}H-NMR (500 MHz, $CDCl_3$): δ = 4.15 (q, $^3J_{H,C}$ = 7.2 Hz, 2H), 2.22 (s, 3H), 1.23 (t, $^3J_{H,C}$ = 7.2 Hz, 3H) ppm. ^{13}C-NMR (126 MHz, $CDCl_3$): δ = 200.7 (C_q), 167.1 (C_q), 61.3 (CH_2), 49.6 (quin, $^1J_{C,D}$ = 13.1 Hz, C^2H_2), 29.0 (CH_3), 14.1 (CH_3) ppm. EI-MS (70 eV): *m/z* (%) = 132 (15), 124 (4), 116 (8), 103 (10), 90 (35), 89 (40), 88 (32), 70 (10), 61 (13), 43 (100).

Synthesis of ethyl 2-(2,5,5-trimethyl-1,3-dioxan-2-yl)-(2,2-$^2H_2$)acetoacetate (S3)
Deuterated 2,2-dimethylpropanediol was prepared by dissolving 2,2-dimethylpropanediol (5.0 g, 48.1 mmol) in D_2O (30 mL) followed by freeze drying for three cycles. To a solution of **S2** (1.98 g, 15.0 mmol, 1.0 eq) in dichloromethane (35 mL) was added *p*-TsOH (42.7 mg, 22.5 mmol, 0.015 eq) and deuterated 2,2-dimethylpropanediol (4.7 g, 45.0 mmol, 3.0 eq). The mixture was stirred for 24 h under reflux conditions. A saturated $NaHCO_3$ solution (in D_2O) was added and the aqueous phase was extracted three times with hexane (10 mL). The organic layer was dried with $MgSO_4$ and concentrated under reduced pressure. The residue was purified through column chromatography (petroleum ether/ethyl acetate, 85:15) to yield **S3** (2.80 g, 12.8 mmol, 85%) as a colourless oil. GC (HP5-MS): *I* = 1338. ^{1}H NMR (500 MHz, $CDCl_3$): δ = 4.16 (q, $^3J_{H,H}$ = 11.0 Hz, 2H), 3.56 (d, $^2J_{H,H}$ = 11.4 Hz, 2H), 3.50 (d, $^2J_{H,H}$ = 11.5 Hz, 2H), 1.54 (s, 3H), 0.98 (s, 3H), 0.94 (s, 3H), 0.88 (t, $^3J_{H,H}$ = 7.2 Hz, 3H) ppm. ^{13}C NMR (126 MHz, $CDCl_3$): δ = 169.7 (C_q), 97.4 (C_q), 70.8 (2x CH_2), 60.7 (CH_2), 41.5 (quin, $^1J_{C,D}$ = 19.9 Hz, C^2H_2), 30.0 (C_q), 22.9 (CH_3), 22.7 (CH_3), 14.2 (CH_3) ppm. IR (diamond ATR): ṽ = 2980 (w), 2955 (m), 2870 (m), 1731 (s), 1473 (m), 1447 (m), 1254 (s), 1209 (s), 1140 (s), 1113 (m), 1018 (m), 950 (m), 908 (m) cm^{-1}. EI-MS (70 eV): *m/z* (%) = 218 (5), 203 (45), 129 (100), 117 (20), 105 (15), 87 (24), 69 (55), 56 (42), 43 (80). HR-ESIMS analysis (positive); calcd. for $C_{11}H_{19}D_2O_4$ $[M+H]^+$: *m/z* 219.1560, found: *m/z* 219.1561.

Synthesis of 2-(2,5,5-trimethyl-1,3-dioxan-2-yl)-(2,2-$^2H_2$)ethanol (S4)

A solution of ester **S3** (2.73 g, 12.5 mmol, 1.0 eq) in THF (10 mL) was added to a cooled (0 °C) suspension of lithium aluminium hydride (0.47 g. 12.5 mmol, 1.0 eq) in THF (5 mL). After 3 h of stirring a saturated aqueous potassium carbonate solution was added, the mixture was filtrated, and the aqueous layer was extracted three times with diethyl ether (50 mL). The pooled organic layers were dried with $MgSO_4$ and concentrated under reduced pressure. The residue was subjected to column chromatography (petroleum ether/ethyl acetate, 7:3) to obtain **S4** (1.99 g, 11.3 mmol, 90%) as a colourless oil. GC (HP5-MS): *I* = 1238. ^{1}H NMR (500 MHz, C_6D_6): δ = 3.93 (m, 2H), 3.28 (d, $^2J_{H,H}$ = 11.4 Hz, 2H), 3.11 (d, $^2J_{H,H}$ = 11.5 Hz, 2H), 1.17 (s, 3H), 0.96 (s, 3H), 0.41 (s, 3H) ppm. ^{13}C NMR (126 MHz, C_6D_6): δ = 100.0 (C_q), 70.2 (CH_2), 42.1 (quin, $^1J_{C,D}$ = 19.5 Hz, C^2H_2), 29.7 (C_q), 22.8 (CH_3), 22.1 (CH_3), 19.1 (CH_3), 19.0 (CH_3) ppm. IR (diamond ATR): $\tilde{\nu}$ = 3428 (s), 2954 (m), 2868 (m), 1740 (w), 1473 (m), 1395 (m), 1275 (m), 1208 (s), 1124 (s), 1080 (s), 1038 (s), 1016 (s), 952 (m), 505 (m) cm^{-1}. EI-MS (70 eV): *m/z* (%) = 176 (3), 161 (55), 129 (65), 91 (30), 75 (40), 69 (60), 56 (55), 43 (100). HR-APCI-MS analysis (positive); calcd. for $C_9H_{17}D_2O_3$ $[M+H]^+$: *m/z* 177.1460, found: *m/z* 177.1458.

Synthesis of tert-butyldiphenyl(2-(2,5,5-trimethyl-1,3-dioxan-2-yl)-(2,2-$^2H_2$)ethoxy)silane (S5)

To a cooled (0 °C) solution of **S4** (1.99 g, 11.3 mmol, 1.0 eq) in dichloromethane (25 mL) were added TBDPSCl (3.44 g, 12.5 mmol, 1.1 eq) and imidazole (1.70 g, 25.0 mmol, 2.2 eq). The mixture was stirred overnight, water was added and the phases were separated. The aqueous layer was extracted three times with ethyl acetate (40 mL) and the pooled organic layers were dried with $MgSO_4$ and concentrated under reduced pressure. The residue was purified by column chromatography (petroleum ether/ethyl acetate, 8:2) to obtain **S5** (3.20 g, 7.72 mmol, 68%) as a colourless oil. GC (HP5-MS): *I* = 2577. ^{1}H NMR (500 MHz, C_6D_6): δ = 7.82 – 7.80 (m, 4H), 7.22 – 7.20 (m, 6H), 4.05 (d, $^2J_{H,C}$ = 1.4 Hz, 2H), 3.25 (d, $^2J_{H,C}$ = 11.4 Hz, 2H), 3.17 (d, $^2J_{H,C}$ = 11.4 Hz, 2H), 1.33 (d, $^3J_{H,C}$ = 1.2 Hz, 3H), 1.19 (s, 9H), 0.72 (s, 3H), 0.64 (s, 3H) ppm. ^{13}C NMR (126 MHz, C_6D_6): δ = 136.1 (4x CH), 134.4 (2x C_q), 129.9 (2 x CH), 128.1 (4x CH), 98.2 (C_q), 70.2 (2x CH_2), 60.4 (CH_2), 40.1 (quin, $^1J_{H,C}$ = 19.5 Hz, C^2H_2), 29.8 (C_q), 27.2 (3x CH_3), 22.7 (CH_3), 22.5 (CH_3), 21.8 (CH_3), 19.4 (C_q) ppm. IR (diamond ATR): $\tilde{\nu}$ = 3049 (w), 2954 (m), 2930 (m), 2857 (m), 1589 (m), 1463 (m), 1187 (w), 1149 (s), 1110 (s), 1040 (w), 1018 (m), 950 (w), 822 (m), 701 (s), 504 (s) cm^{-1}. EI-MS (70 eV): *m/z* (%) = 399 (5), 271 (100), 241 (35), 199 (35), 197 (28), 181 (15), 129 (40), 69 (25), 56 (40), 41 (32). HR-ESIMS analysis (positive); calcd. for $C_{25}H_{35}D_2O_3Si$ $[M+H]^+$: *m/z* 415.2638, found: *m/z* 415.2632.

Synthesis of 4-((*tert*-butyldiphenylsilyl)oxy)-(3,3-$^2H_2$)butan-2-one (S6)[25]

To a solution of silyl ether **S5** (1.60 g, 3.86 mmol, 1.0 eq) in methanol (15 mL) was added HCl (1 M, 3.86 mL, 3.86 mmol, 1.0 eq) and the mixture was stirred for 1 h. An aqueous $NaHCO_3$ solution was added to neutralise the mixture and the product was extracted three times with hexane (30 mL). The combined organic layers were dried with $MgSO_4$ and concentrated under reduced pressure. The residue was purified by column chromatography (petroleum ether/ethyl acetate, 8:2) to obtain **S6** (810 mg, 2.46 mmol, 64%) as a colourless oil. GC (HP5-MS): *I* = 2199. ^{1}H NMR (500 MHz, C_6D_6): δ = 7.77 – 7.75 (m, 4H), 7.24 – 7.22 (m, 6H), 3.83 (br d, $^2J_{H,C}$ = 2.0 Hz, 2H), 1.70 (d, $^3J_{H,C}$ = 1.1 Hz, 3H), 1.14 (s, 9H) ppm. ^{13}C NMR (126 MHz, C_6D_6): δ = 136.0 (4x CH), 134.0 (2x C_q), 130.1 (2x CH), 128.2 (4x CH), 59.9 (CH_2), 45.6 (quin, $^1J_{C,D}$ = 19.6 Hz, C^2H_2), 30.1 (C_q), 27.1 (3x CH_3), 19.4 (C_q) ppm. EI-MS (70 eV): *m/z* (%) = 271 (100), 241 (65), 223 (10), 211 (12), 199 (80), 192 (48), 181 (36), 166 (15), 152 (10), 140 (15), 121 (12), 105 (20), 91 (12), 77 (35), 57 (80), 43 (55), 41 (52).

Synthesis of *tert*-butyl((3-methyl(2,2-$^2H_2$)but-3-en-1-yl)oxy)diphenylsilane (S7)[25]

To a cooled (0 °C) suspension of methyltriphenylphosphonium iodide (1.98 g, 4.87 mmol, 2.0 eq) in THF (20 mL) was added nBuLi (1.6 M in hexane, 3.04 mL, 4.87 mmol, 2.0 eq) to obtain a dark red solution. After 1.5 h the ketone **S6** (800 mg, 2.44 mmol, 1.0 eq) was added and the mixture was allowed to warm to room temperature overnight. Water was added and the phases

were separated. The aqueous layer was extracted three times with pentane (30 mL), the organic layers were dried with $MgSO_4$ and concentrated under reduced pressure. The residue was subjected to column chromatography (petroleum ether/ethyl acetate, 10 :1) to yield **S7** (308 mg, 0.94 mmol, 39%) as a colourless oil. GC (HP5-MS): *I* = 2074. ^{1}H NMR (500 MHz, C_6D_6): δ = 7.79 – 7.77 (m, 4H), 7.23 – 7.21 (m, 6H), 4.80 (dt, $J_{H,C}$ = 2.9, 1.5 Hz, 1H), 4.78 (d, $J_{H,C}$ = 1.8, 1.0 Hz, 1H), 3.74 (br s, 2H), 1.57 (br s, 3H), 1.17 (s, 9H) ppm. ^{13}C NMR (126 MHz, C_6D_6): δ = 142.9 (C_q), 136.0 (4x CH), 134.4 (2x C_q), 130.0 (2x CH), 128.1 (4x CH), 112.3 (CH_2), 62.9 (CH_2), 40.6 (quin, $J_{C,D}$ = 19.5 Hz, C^2H_2), 27.1 (3x CH_3), 22.7 (CH_3), 19.5 (C_q) ppm. EI-MS (70 eV): *m*/*z* (%) = 281 (1), 269 (85), 240 (12), 239 (30), 227 (35), 211 (25), 191 (70), 181 (40), 147 (10), 161 (28), 135 (25), 121 (15), 105 (30), 91 (12), 77 (20), 57 (100), 41 (55).

Synthesis of (2,2-$^2H_2$)-3-methylbut-3-en-1-yl 4-methylbenzenesulfonate (S8)[25]

To a cooled (0 °C) solution of silyl ether **S7** (308 mg, 0.94 mmol, 1.0 eq) in THF (0.6 mL) was added tetrabutylammonium fluoride solution (1.0 M in THF, 1.4 mL, 1.4 mmol, 1.2 eq). The mixture was kept at 0 °C overnight under stirring, and saturated aqueous ammonium chloride solution was added and the mixture extracted three times with diethyl ether (30 mL).[3] The organic layer was dried with $MgSO_4$, cautiously concentrated under reduced pressure (650 mbar) and then diluted with dichloromethane (4 mL). The solution was cooled to 0 °C and DMAP (380 mg, 3.11 mmol, 3.3 eq) and tosyl chloride (450 mg, 2.36 mmol, 2.5 eq) were added. After stirring for 3 h saturated aqueous ammonium chloride solution was added. The phases were separated and the aqueous layer was extracted three times with diethyl ether (15 mL). The combined organic layers were dried with $MgSO_4$ and concentrated under reduced pressure. The crude product was purified by column chromatography (petroleum ether/ethyl acetate, 4:1) to obtain **S8** (159 mg, 0.66 mmol, 70%) as a colourless oil. GC (HP5- MS): *I* = 1818. ^{1}H NMR (500 MHz, C_6D_6): δ = 7.74 (dd, $^3J_{H,H}$ = 8.3 Hz, $^4J_{H,H}$ = 3.4 Hz, 2H), 6.66 (d, $^3J_{H,H}$ = 8.3 Hz, 2H), 4.66 (ddq, $^4J_{H,H}$ = 1.3 Hz, $^2J_{H,H}$ = 10.7 Hz, 1H), 4.55 (dt, $^2J_{H,H}$ = 1.8 Hz, $^4J_{H,H}$ = 1.0 Hz, 1H), 3.92 (br s, 2H), 1.81 (s, 3H), 1.36 (d, $^4J_{H,C}$ = 1.3 Hz, 3H) ppm. ^{13}C NMR (126 MHz, C_6D_6): δ = 144.1 (C_q), 135.3 (C_q), 134.6 (C_q), 129.7 (2x CH), 128.2 (2x CH), 113.1 (CH_2), 68.2 (CH_2), 36.5 (quin, $^1J_{C,D}$ = 19.6 Hz, C^2H_2), 26.8 (CH_3), 22.1 (CH_3) ppm. EI-MS (70 eV): *m*/*z* (%) = 173 (8), 155 (45), 107 (5), 91 (100), 89 (15), 69 (75), 65 (45), 57 (12), 39 (20).

Synthesis of trisammonium (2,2-^{2}H)IPP (S9)[25]

A solution of tosylate **S8** (154 mg, 0.636 mmol, 1.0 eq) in acetonitrile (6 mL) was added to a solution of $(NBu_4)_3HP_2O_7$ (686.5 g, 0.76 mmol, 1.2 eq) in acetonitrile (5 mL). The mixture was stirred at room temperature overnight and the solvent was removed under reduced pressure. The residue was dissolved in NH_4HCO_3 solution (25 mM in water, 2% iPrOH) and loaded onto a cation exchange column (DOWEX 50WX8), prepared by flushing first with 1 CV of ammonia solution (25% in water) and subsequently with water to pH 7. The desired product was eluted with 1.5 CV of NH_4HCO_3 solution and subjected to lyophilisation. The resulting salt was dissolved in NH_4HCO_3 solution (0.1 M in water) and a mixture of acetonitrile/iPrOH (1:1) was added until a precipitate was formed. After centrifugation the supernatant was collected and the procedure was repeated twice with the precipitate. The pooled supernatant fractions were concentrated to remove acetonitrile and iPrOH. Residual water was removed by lyophilisation to obtain **S9** as a colourless solid (180 mg, 0.608 mmol, 96%). ^{1}H NMR (500 MHz, D_2O): δ = 7.62 (d, $^2J_{H,H}$ = 8.3 Hz, 1H), 7.30 (d, $^2J_{H,H}$ = 8.3 Hz, 1H), 4.00 – 3.97 (m, 2H), 1.76 (s, 3H) ppm. ^{13}C NMR (126 MHz, D_2O): δ = 143.9 (C_q), 111.6 (CH_2), 64.1 (CH_2), 37.5 (dquin, $^3J_{P,C}$ = 7.4 Hz, $^1J_{P,C}$ = 22.0 Hz, C^2H_2), 21.7 (CH_3) ppm. ^{31}P-NMR (126 MHz, D_2O): δ = –7.2 (d, $^2J_{P,P}$ = 21.5 Hz), –10.4 (d, $^2J_{P,P}$ = 21.9 Hz) ppm.

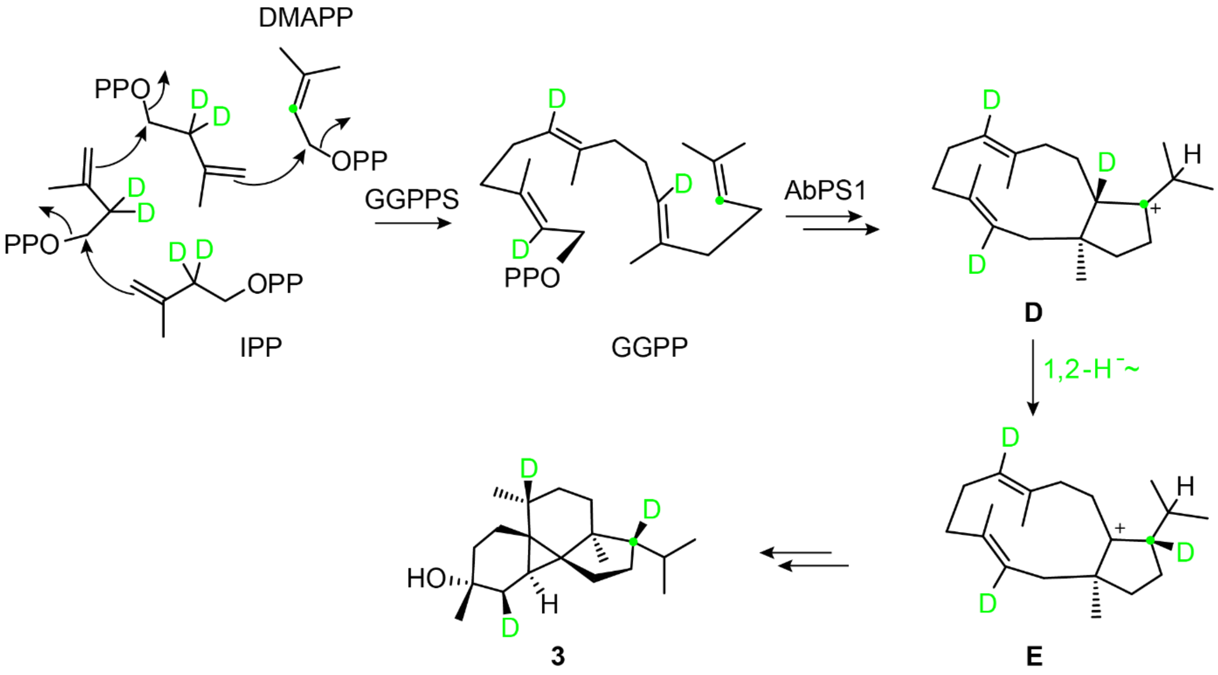

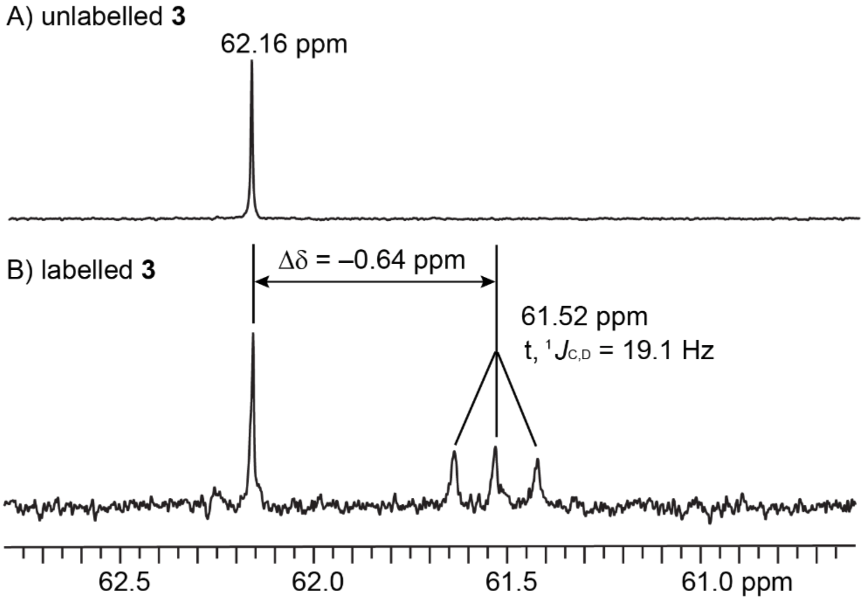

Figure S32. The 1,2-hydride shift from **D** to **E** in the formation of **3**. A) ^{13}C-NMR signal of C14 of unlabelled **3**. B) ^{13}C-NMR signal for C14 of labelled **3** obtained from (2-^{13}C)DMAPP and (2,2-^{2}H)IPP with GGPPS and AbPS1. Green dots represent ^{13}C-labelled carbons. The slightly upfield shifted triplet in B) is indicative for a direct ^{13}C-^{2}H bond and supports the proposed 1,2-hydride shift.

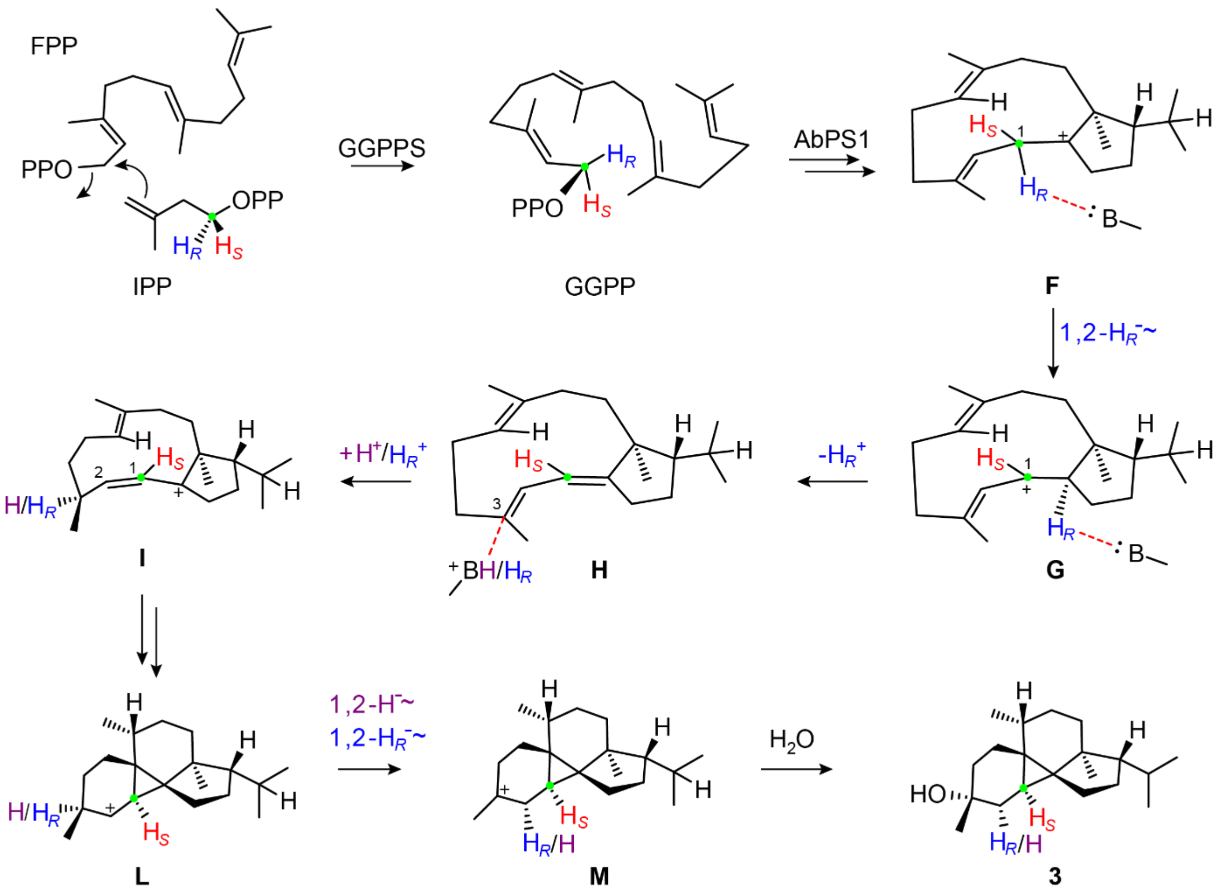

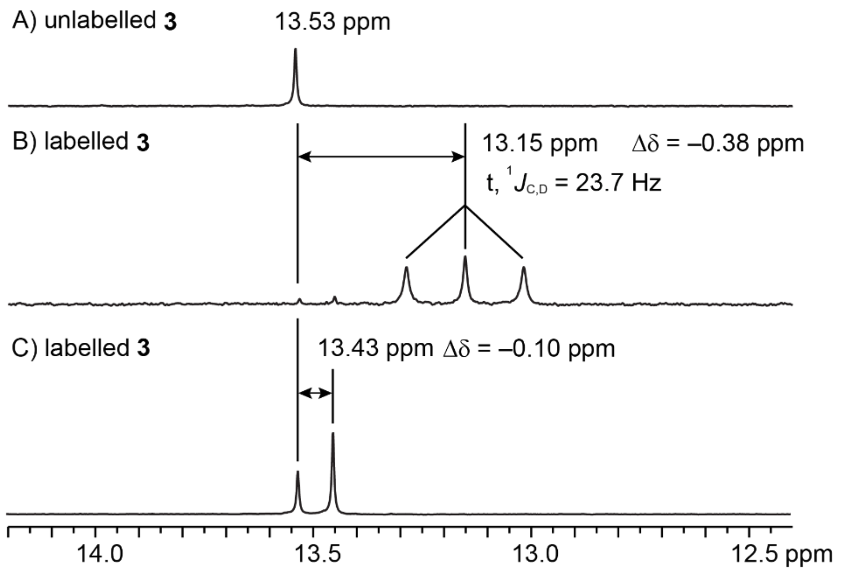

Figure S33. The deprotonation-reprotonation sequence **G**-**H**-**I** and the 1,2-hydride shift from **L** to **M** in the formation of **3**. A) ^{13}C-NMR signal of C1 of unlabelled **3**. B) ^{13}C-NMR signal for C1 of labelled **3** obtained from FPP and (*S*)-(1-^{13}C,1-^{2}H)IPP (H_S=^{2}H) with GGPPS and AbPS1. C) ^{13}C-NMR signal for C1 of labelled **3** obtained from FPP and (*R*)-(1-^{13}C,1-^{2}H)IPP (H_R=^{2}H) with GGPPS and AbPS1. Green dots represent ^{13}C-labelled carbons. The slightly upfield shifted triplet in B) indicates that the 1-*pro*-*S* hydrogen stays at C1, while the unchanged singlet in C) is in agreement with the loss of the 1-*pro*-*R* hydrogen and the slightly upfield shifted singlet indicates a deuterium at C2.

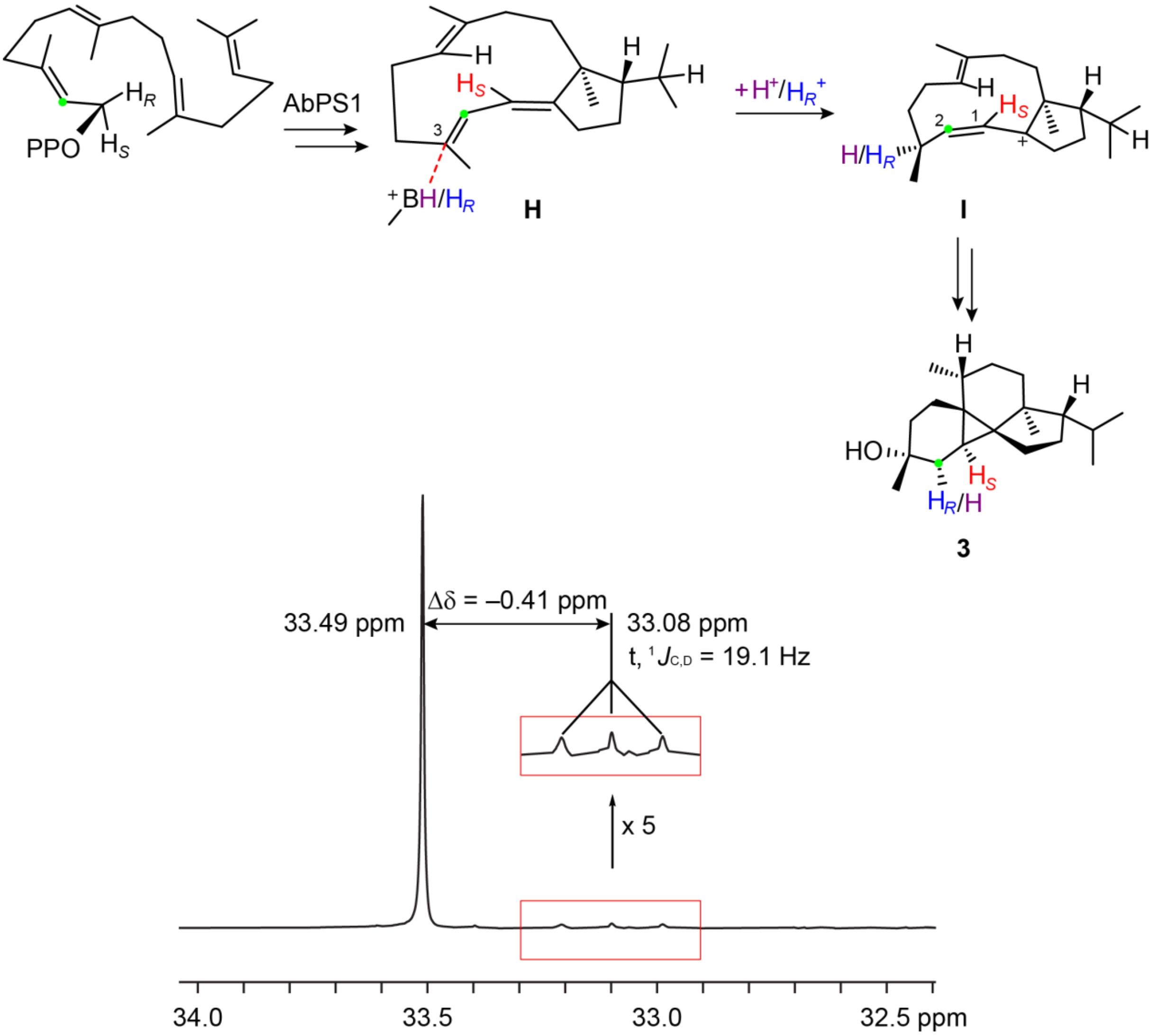

Figure S34. The deprotonation-reprotonation sequence **G**-**H**-**I** and the 1,2-hydride shift from **L** to **M** in the formation of **3**. ^{13}C-NMR signal for C2 of labelled **3** obtained from (2-^{13}C)GGPP in a deuterium oxide buffer with AbPS1 (purple hydrogen is substituted by deuterium). Green dots represent ^{13}C-labelled carbons. The slightly upfield shifted triplet is indicative for a direct ^{13}C-^{2}H bond and supports the proposed reprotonation at C3 in **I** and the 1,2-hydride shift from **L** to **M**.

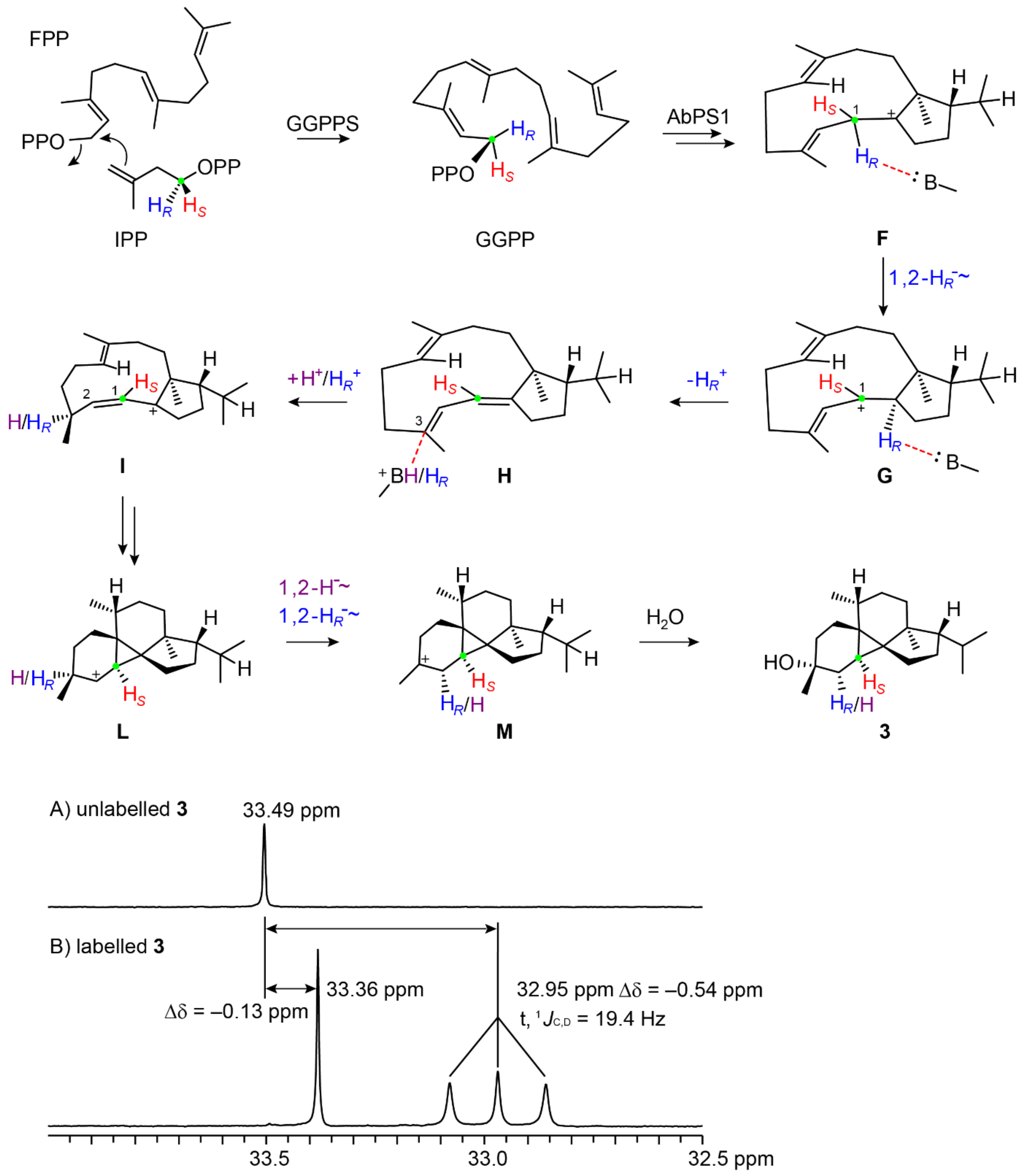

Figure S35. The deprotonation-reprotonation sequence **G-H-I** and the 1,2-hydride shift from **L** to **M** in the formation of **3**. A) ^{13}C-NMR signal for C2 of unlabelled **3**. B) ^{13}C-NMR signal for C2 of labelled **3** obtained from FPP and (2-^{13}C,1,1-^{2}H)DMAPP (H_R=H_S=^{2}H) with IDI, GGPPS and AbPS1. Green dots represent ^{13}C-labelled carbons. The slightly upfield shifted triplet in B) is indicative for a direct ^{13}C-^{2}H bond and supports the retainment of the 1-*pro*-*R* hydrogen with incorporation at C2. The upfield shifted singlet arises from **3** that has lost deuterium from the 1-*pro*-*R* position, but retained it from the 1-*pro*-*S* position at a neighbouring carbon (C1).

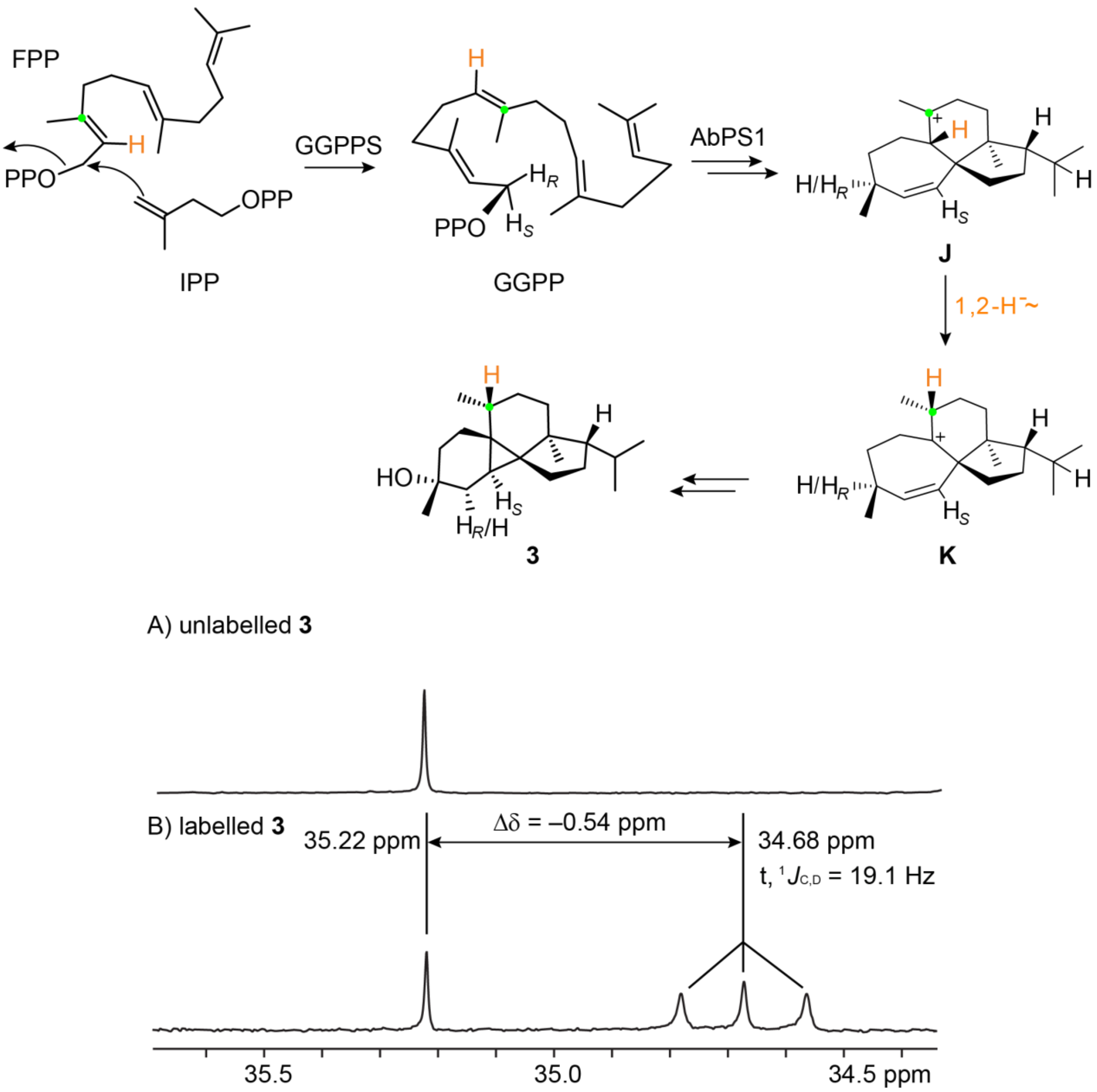

Figure S36. The 1,2-hydride shift from **J** to **K** in the formation of **3**. A) ^{13}C-NMR signal of C7 of unlabelled **3**. B) ^{13}C-NMR signal for C7 of labelled **3** obtained from (3-^{13}C,2-^{2}H)FPP (orange H is substituted by deuterium) and IPP with GGPPS and AbPS1. Green dots represent ^{13}C-labelled carbons. The slightly upfield shifted triplet in B) is indicative for a direct ^{13}C-^{2}H bond and supports the proposed hydride shift.

Computational methods

All computed structures were geometry optimized without restrictions and were characterized as minima or as transition state structures by frequency analyses using the B97D3/6-31g(d,p) method with the density fitting approximation for s- and p-functions, including Grimme's empirical D3-dispersion correction[26] in Gaussian16.[27] Frequency computations also provided Gibbs corrections, which include Grimme's quasi-RRHO approach with a frequency cut-off value of 100.0 wave numbers using GoodVibes.[28,29] For single point energies, the mPW1PW91 functional was applied with the 6-311+G(d,p) basis set without density fitting and the ultra-fine integration grid, as this method was shown to be very reliable for examining carbocation cyclization and rearrangement reactions.[30-34] Conformational analyses were performed with xTB-GFN2 in the CREST 2.12 program (github.com/crest-lab), developed by the Grimme group.[35-39]

Table S7. Results of DFT calculations for the cyclisation cascade from GGPP to **3 – 5** (Scheme 1 of main text).

Structure[a]	Gibbs energy (298.15K) in Hartree	energy relative to A in kcal/mol	reaction barrier in kcal/mol	Gibbs free energy in kcal/mol
**A**	–781.679303	0.00		
**A-B-TS**	–781.673773	3.47	3.47	
**B**	–781.696468	–10.77		–10.77
**B**	–781.696458	–10.76		
**B-C-TS**	–781.698413	–11.99	–1.23	
**C**	–781.710044	–19.29		–8.53
**C**	–781.710040	–19.29		
**C-D-TS**	–781.700998	–13.61	5.67	
**D**	–781.721520	–26.49		–7.20
**D**	–781.720938	–26.13		
**D-E-TS**	–781.712048	–20.55	5.58	
**E**	–781.718507	–24.60		1.53
**E**	–781.718502	–24.60		
**E-F-TS**	–781.708571	–18.37	6.23	
**F**	–781.718626	–24.68		–0.08
**F**	–781.708263	–18.17		
**F-G-TS**	–781.675855	2.16	20.34	
**G**	–781.702814	–14.75		3.42
**G***	–781.712098	–20.58		
**G-H-TS***	–781.701818	–14.13	6.45	
**H***	–781.712743	–20.98		–0.40
**H***	–781.718408	–24.54		
**H-I-TS***	–781.711354	–20.11	4.43	
**I***	–781.718873	–24.83		–0.29
**I**	–781.725886	–29.23		
**I-J-TS**	–781.727175	–30.04	–0.81	
**J**	–781.734031	–34.34		–5.11
**J**	–781.734032	–34.34		
**J-K-TS**	–781.730340	–32.03	2.32	
**K**	–781.751201	–45.12		–10.77
**K**	–781.751204	–45.12		
**K-L-TS**	–781.754319	–47.07	–1.95	
**L**	–781.756135	–48.21		–3.09
**L**	–781.759897	–50.57		
**L-M-TS**	–781.738678	–37.26	13.32	
**M**	–781.744058	–40.63		9.94

[a] Intermediates and transition states marked with an asterisk have been computed with NH_3 as surrogate base, and the given Gibbs energies equal the computed Gibbs energies reduced by –56.563161 Hartree (computed Gibbs energy of NH_3) to allow for a direct comparison to the computed energies for steps without NH_3.

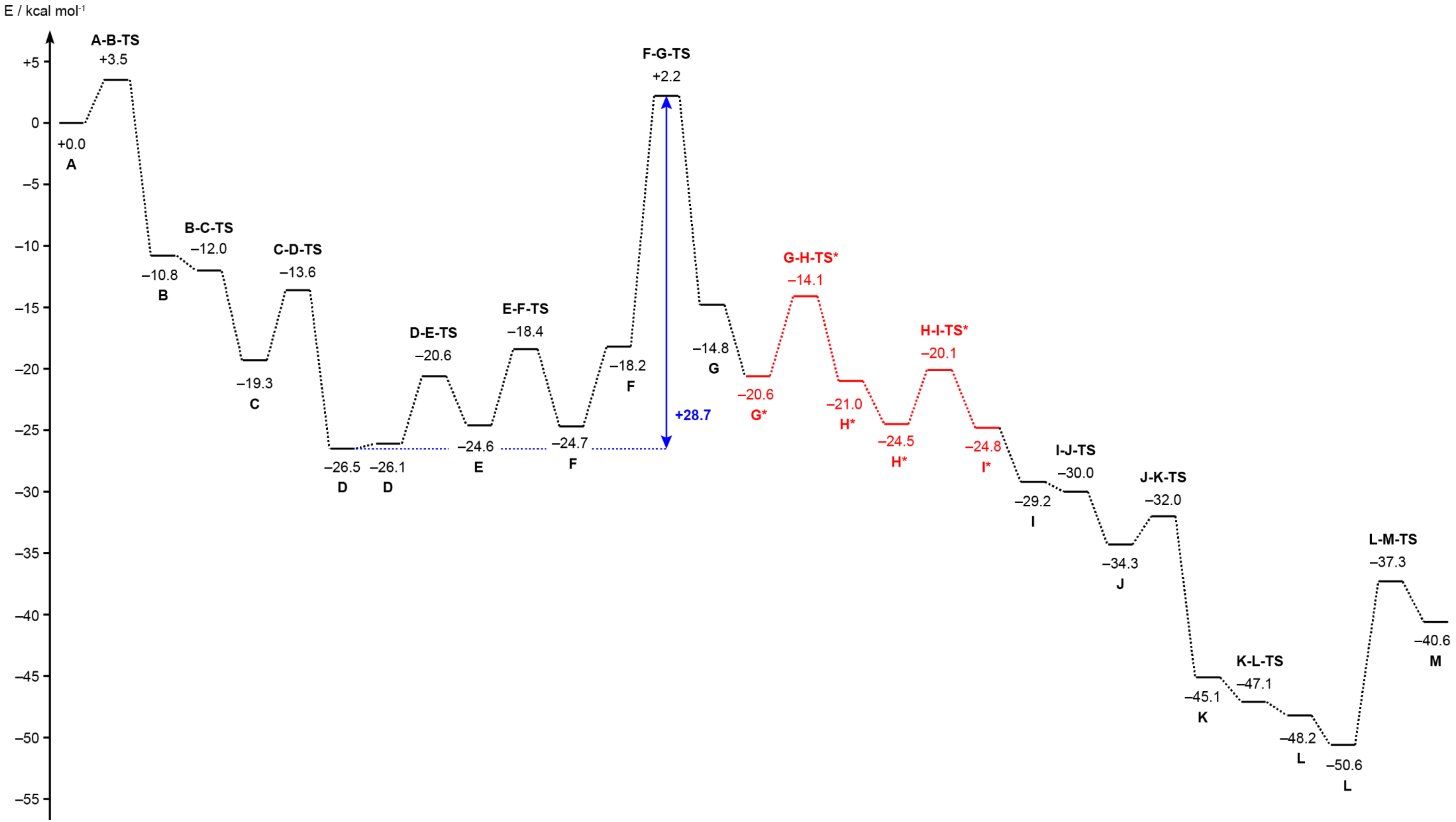

Figure S37. Computed energy profile for the transformations of **A** to **M** (Scheme 1 of main text, mPW1PW91/6-311+G(d,p)//B97D3/6-31g(d,p), 298 K). Intermediates and transition states marked with an asterisk and shown in red have been computed with NH_3 as surrogate base. The blue arrow shows a reaction barrier that is too high to explain the biosynthesis of **3** via this mechanism.

Molecular simulation

AlphaFold2 v2.3.0 was used to construct the structural models of AbPS1 and AbPS2 and to predict their substrate pockets.[40] The structural model of the ligand GGPP was downloaded from the PubChem database (https://pubchem.ncbi.nlm.nih.gov/) and docked into the cofactor-binding site of AbPS1 using AutoDock Vina v1.5.6.[41] The three Mg^{2+} cations were taken from the aligned crystal structure of VenA (PDB: 7Y9G). PyMOL 2.1 (http://www.pymol.org) was used to view the molecular interactions and to process the image.

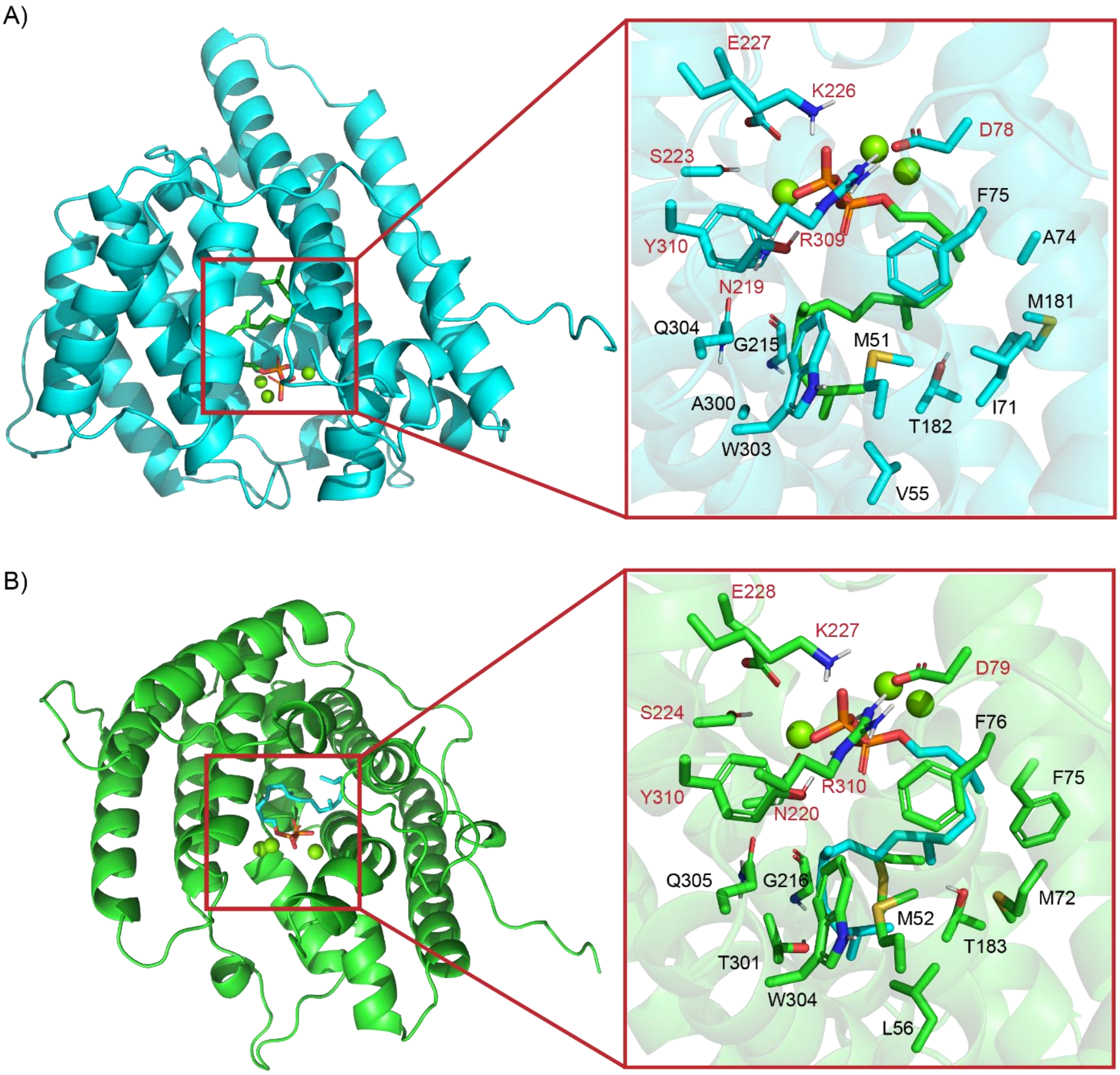

Figure S38. AlphaFold2 models and close-up views on the active site architectures of A) AbPS1 and B) AbPS2 docked with GGPP. The three Mg^{2+} cations were added from an aligned structure of VenA (PDB: 7Y9G). The amino acid residues labelled in red belong to the highly conserved motifs for protein functionality, the amino acids labelled in black contour the hydrophobic pocket.

Site-directed mutagenesis of AbPS1 and AbPS2

Site-directed mutagenesis was performed through PCR using pairs of reverse complement mutational primers (Table S1) to amplify the entire expression plasmids pESC-AbPS1 or pESC-AbPS2, respectively. Through the same strategy the *in vitro* expression plasmids pET32a-AbPS1-T182A, pMALc6T-AbPS2-F76A, and pMALc6T-AbPS2-L56V-F75A were derived from the templates pET32a-AbPS1 or pMALc6T-AbPS2. The resulting PCR product was incubated with DpnI to digest the parent vector, followed by transformation into *E. coli* DH5α. All mutated plasmids were isolated and mutations were verified by DNA sequencing. The pMALc6T-derived plasmids were introduced into *S. cerevisiae* for in vivo production and the pET32a-derived plasmids were introduced into *E. coli* BL21(DE3) by electroporation for heterologous expression and in vitro incubations.

Heterologous expression of AbPS1 and AbPS2 enzyme variants in the *S. cerevisiae* production strain

Seed cultures of *S. cerevisiae* containing the pESC-derived plasmids with the mutated *AbPS1* or *AbPS2* gene were cultured overnight at 30 °C and 220 rpm. The seed cultures were used to inoculate production cultures in YPD medium (50 mL) with an initial OD_{600} = 0.1. Fermentation was performed at 30 °C and 220 rpm for 3 days. The cultures were extracted with hexane (2x 50 mL), the extracts were combined and concentrated. The residue was redissolved in hexane (1 mL) and the obtained solution was analysed by GC/MS. All experiments were performed in triplicates for the determination of relative productions (mean ± standard deviation). The relative production of **3** and **4** are shown in Figure S39 and the production of other compounds is summarised in Tables S8 and S9. Compound production by additional AbPS1 enzyme variants (alanine scanning of active site residues) is shown in Figure S40.

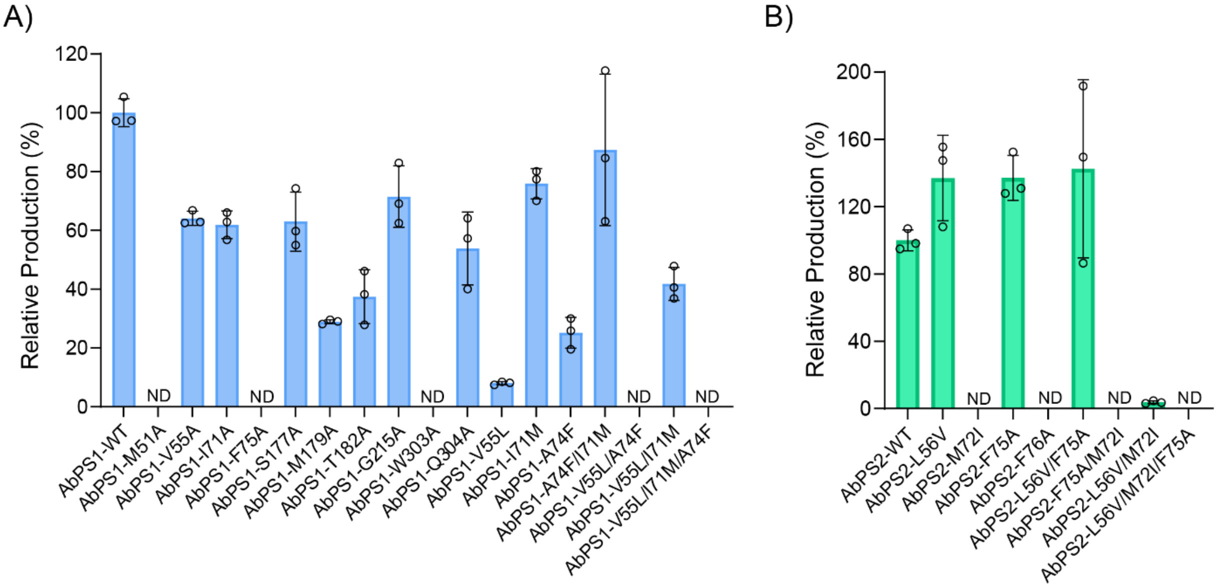

Figure S39. Relative compound production of A) **3** by AbPS1 and B) **4** by AbPS2 and their enzyme variants in vivo. Black circles indicate the results from three independent experiments, the error bars indicate mean and standard deviations. ND denotes no production detected.

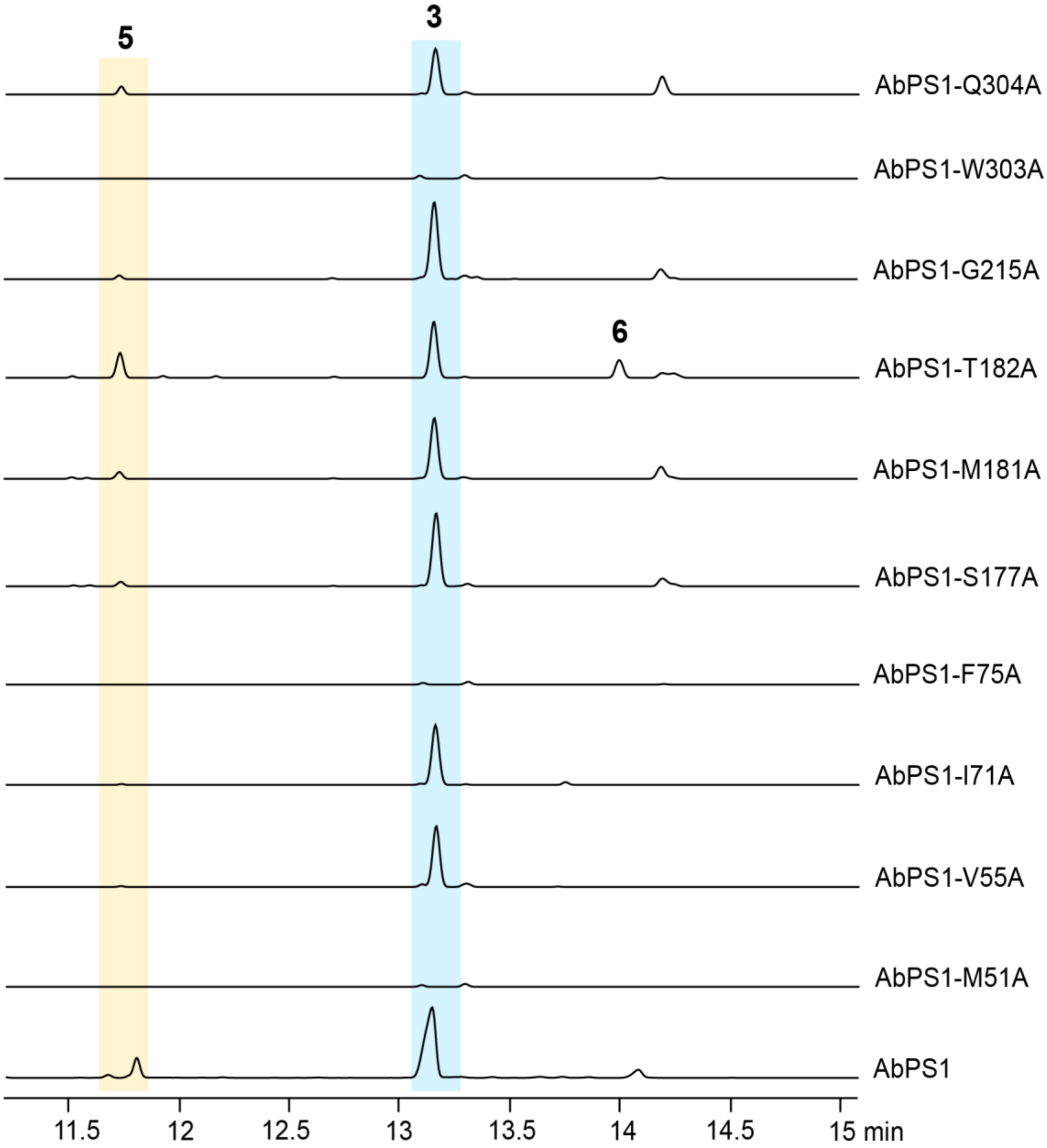

Figure S40. Alanine scanning of AbPS1 active site residues. Extracted ion chromatograms (*m*/*z* 272) of extracts from *S. cerevisiae* cultures expressing AbPS1 and its variants.

Table S8. Relative compound production by AbPS1 and its enzyme variants in vivo.

**Variant**	**3**[a]	**5**	**6**
AbPS1-WT	100±4%	2.5±0.4%	–
AbPS1-M51A	–	–	–
AbPS1-V55A	64±2%	–	–
AbPS1-I71A	62±4%	1.9±0.4%	–
AbPS1-F75A	–	–	–
AbPS1-S177A	63±8%	2.3±0.4%	–
AbPS1-M181A	29±1%	3.0±1.0%	–
AbPS1-T182A	37±7%	4.0±1.5%	3.9±1.7%
AbPS1-G215A	71±9%	1.6±1.6%	–
AbPS1-W303A	–	–	–
AbPS1-Q304A	54±10%	2.8±0.1%	–
AbPS1-V55L	8.0±0.4%	–	–
AbPS1-I71M	76±4%	2.2±0.3%	–
AbPS1-A74F	25±4%	–	–
AbPS1-V55L/I71M	42±5%	–	–
AbPS1-V55L/A74F	–	–	–
AbPS1-I71M/A74F	87±21%	1.7±0.2%	–
AbPS1-V55L/I71M/A74F	–	–	–

[a] The production of **3** by wildtype AbPS1 was arbitrarily set to 100%. The data are mean and standard deviations from triplicates.

Table S9. Relative compound production by AbPS2 and its enzyme variants in vivo.

**Variant**	**3**	**4**	**5**	**7**
AbPS2-WT	–	100±5%	6.5±1.4%	–
AbPS2-L56V	1.8±0.1%	137±21%	2.5±0.2%	11±1%
AbPS2-M72I	–	–	–	–
AbPS2-F75A	3.4±0.3%	137±11%	21±1%	19±2%
AbPS2-F76A	–	–	–	–
AbPS2-L56V/M72I	–	3.8±0.7%	–	–
AbPS2-L56V/F75A	10±1%	143±43%	29±2%	15±1%
AbPS2-M72I/F75A	–	–	–	–
AbPS2-L56V/M72I/F75A	–	–	–	–

[a] The production of **4** by wildtype AbPS2 was arbitrarily set to 100%. The data are mean and standard deviations from triplicates.

Heterologous expression of recombinant AbPS1 and AbPS2 enzyme variants in *E. coli* and protein purification

The AbPS1 and AbPS2 enzyme variants (AbPS1-T182A, pMALc6T-AbPS2-L56V/F75A and pMALc6T-AbPS2-F76A) were expressed following the same protocol as the wild type described above. The fractions were analysed by SDS-PAGE, and those containing pure protein were pooled (Figure S41) for subsequent incubation experiments.

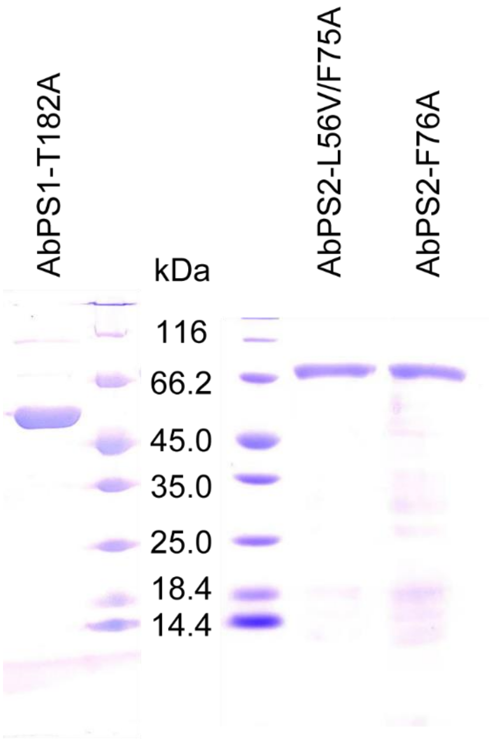

Figure S41. SDS-PAGE analysis of purified recombinant AbPS1 and AbPS2 enzyme variants.

Small scale incubation of GGPP with recombinant AbPS1 and AbPS2 enzyme variants

Analytical scale incubations were performed with substrate (GGPP, 0.5 mg) dissolved in substrate buffer (0.1 mL, 25 mM NH_4HCO_3). After dilution with incubation buffer (0.8 mL, 50 mM Tris, 10 mM $MgCl_2$, 20% glycerol, pH = 7.4), enzyme elution fraction (0.1 mL, containing 5.0 mg mL^{-1} enzyme) was added. The reaction mixture was incubated at 30 °C with shaking for 15 h, followed by extraction with hexane (200 μL). The organic layer was dried with $MgSO_4$ and analysed by GC/MS (Figures S42 and S43).

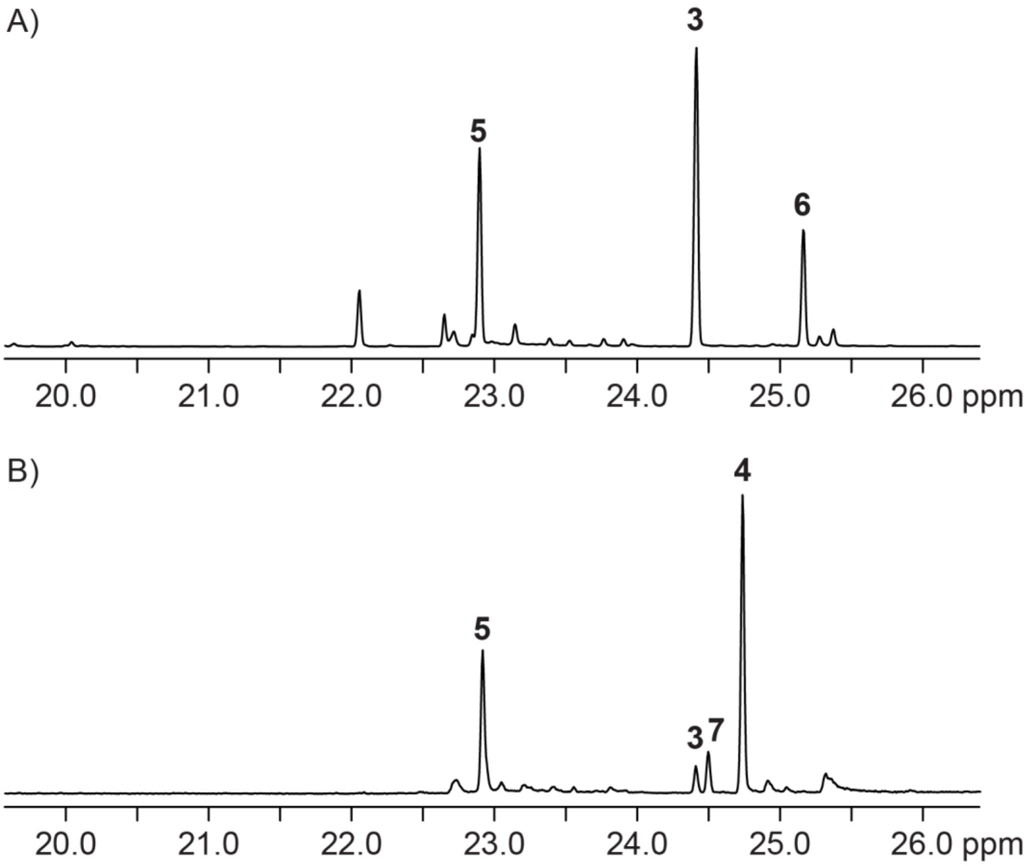

Figure S42. Total ion chromatograms of extracts from enzyme incubations of GGPP with A) AbPS1-T182A and B) AbPS2-L56V-F75A.

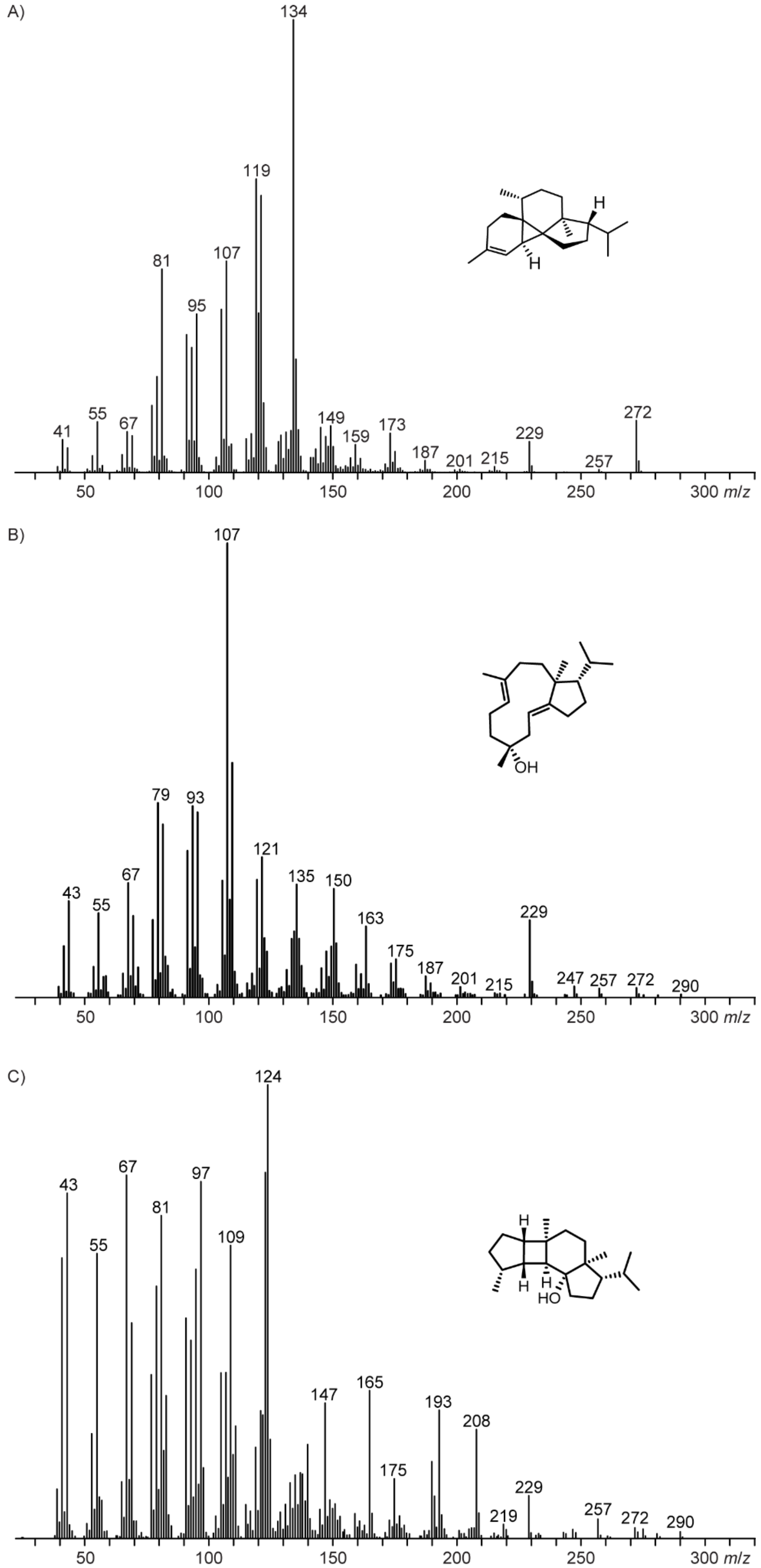

Figure S43. EI mass spectra of A) peyssonnosene (**5**), B) anaerol (**6**) and C) anaerol B (**7**).

Preparative scale incubations with recombinant AbPS1 and AbPS2 enzyme variants and compound isolation of 5 and 7

For preparative scale incubations, GGPP (50 mg, 111 µmol) in NH_4HCO_3 (25 mM, 5 mL) and an enzyme preparation of AbPS1-T182A, AbPS2-L56V-F75A, or AbPS2-F76A (5 mL; from 2 L expression culture, 5.0 mg mL^{-1}) were added to incubation buffer (40 mL). The reaction mixture was stirred overnight at 30 °C and then extracted with Et_2O (3 x 15 mL). The combined extracts were dried with $MgSO_4$ and concentrated in vacuo. Peyssonnosene (**5**, 0.8 mg, 2.8 µmol, 3 %) was obtained from an enzyme reaction with AbPS1-T182A by column chromatography on silica gel with n-pentane, and anaerol B (**7**, 0.8 mg, 2.8 µmol, 2.5 %) was isolated from an enzyme reaction with AbPS2-L56V-F75A by column chromatography on silica gel with petroleum ether and EtOAc (10 : 1) from the AbPS2-L56V-F75A enzyme variant.

Cultivation of a *Saccharomyces cerevisiae* production strain and compound isolation of 6

A culture of *S. cerevisiae* expressing AbPS1-T182A in YPD medium (10 L) was grown for 3 days at 30 °C and 220 rpm. The culture was extracted hexane/ethyl acetate = 4/1 (6x 1.5 L) and the combined extracts were concentrated to obtain a dark brown oil (953.4 mg). Purification by semipreparative HPLC (Prevail C18 column, ACN/H_2O=100/0, flow rate=3.0 mL/min, λ=210 nm) afforded **6** (4.5 mg, t_R = 15.5 min, yield 0.75 mg/L).

Peyssonnosene (5). TLC (petrol ether): R_f = 0.84. GC (HP-5MS): *I* = 1943. IR (diamond ATR): ṽ = 2954 (s), 2924 (s), 2866 (s), 1666 (w), 1460 (m), 1374 (m), 1259 (w), 1164 (m), 1095 (w), 1016 (m), 845 (w), 800 (m) cm^{-1}. HR-MS (EI): calc. $[C_{20}H_{32}]^+$ *m*/*z* = 272.2499; found: *m*/*z* = 272.2497. Optical rotation: $[\alpha]_D^{25}$ = –18.8 (*c* 0.08, CH_2Cl_2). NMR data are given in Table S10.

Anaerol (6). TLC TLC (petrol ether/ethyl acetate = 5/1): R_f = 0.51. GC (HP-5MS): *I* = 2179. IR (diamond ATR): ṽ = 2957(s), 2930(s), 2864(s), 1682(w), 1475(m), 1445(m), 1382(m), 1363(m), 1190(w), 1105(m), 1078(m), 906(m), 858(m), 824(w) cm^{-1}. HR-MS (EI): calc. $C_{20}H_{35}O$ $[M+H]^+$ *m*/*z* = 291.2688; found: *m*/*z* = 291.2686. Optical rotation: $[\alpha]_D^{25}$ = +69.4 (*c* 0.17, CH_2Cl_2). NMR data are given in Table S11.

Anaerol B (7). TLC (petrol ether/ethyl acetate = 5/1): R_f = 0.60. GC (HP-5MS): *I* = 2114. IR (diamond ATR): ṽ = 3510 (br), 2950 (s), 2868 (s), 1673 (w), 1459 (m), 1375 (m), 1260 (w), 1155 (w), 1016 (w), 992 (w), 970 (w), 951 (m), 936 (w), 863 (w) cm^{-1}. HR-MS (EI): calc. $[C_{20}H_{34}O]^+$ *m*/*z* = 290.2604; found: *m*/*z* = 290.2616. Optical rotation: $[\alpha]_D^{25}$ = +22.7 (*c* 0.15, CH_2Cl_2). NMR data are given in Table S12.

Table S10. NMR data of peyssonnosene (**5**) in C_6D_6 recorded at 298 K.

C[a]	type	^{13}C[b]	^{1}H[b]
1	CH	20.17	0.99 (m)
2	CH	120.67	5.65 (br d, *J* = 3.8)
3	C_q	132.46	–
4	CH_2	28.78	1.92 (m, H_α)<br>1.57 (m, H_β)
5	CH_2	23.91	1.73 (m, H_α)<br>1.53 (m, H_β)
6	C_q	28.91	–
7	CH	35.64	1.52 (m)
8	CH_2	27.41	1.25 (m, H_β)<br>0.79 (m, H_α)
9	CH_2	39.50	1.57 (m, H_α)<br>0.95 (m, H_β)
10	C_q	42.30	–
11	C_q	46.51	–
12	CH_2	27.17	1.37 (m, 2H)
13	CH_2	28.72	1.73 (m, H_β)<br>1.28 (m, H_α)
14	CH	62.41	1.20 (ddd, *J* = 13.2, 8.1, 5.1)
15	CH	29.26	1.60 (m)
16	CH_3	23.40	0.94 (d, *J* = 5.9)
17	CH_3	23.64	0.97 (d, *J* = 6.7)
18	CH_3	19.27	0.80 (s)
19	CH_3	19.39	1.00 (d, *J* = 6.3)
20	CH_3	23.74	1.66 (br s)

[a] Carbon numbering as shown in Figure S44 indicates the origin of each carbon from GGPP by same number. [b] Chemical shifts δ in ppm. Multiplicity: s = singlet, d = doublet, m = multiplet, br = broad. Coupling constants *J* are given in Hertz.

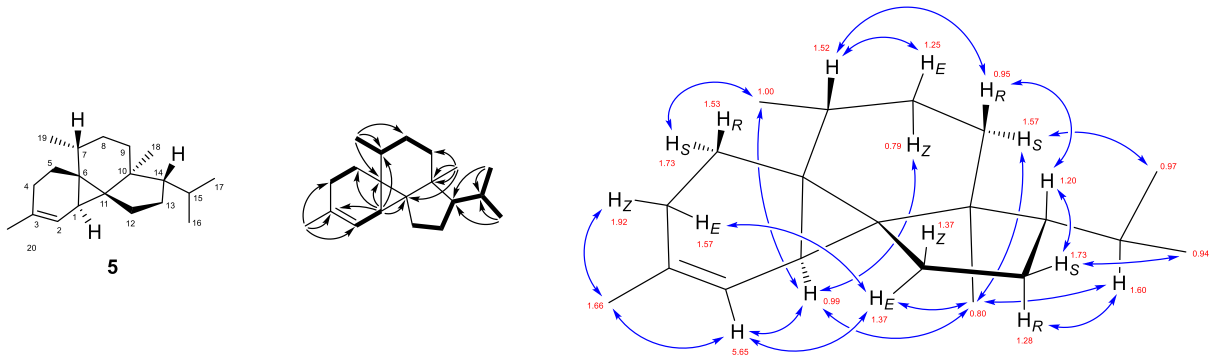

Figure S44. Structure elucidation of peyssonnosene (**5**). Bold: ^{1}H,^{1}H-COSY, single headed arrows: key HMBC, and blue double headed arrows: NOESY correlations. H_R, H_S, H_E and H_Z indicate the results from stereoselective labelling experiments.

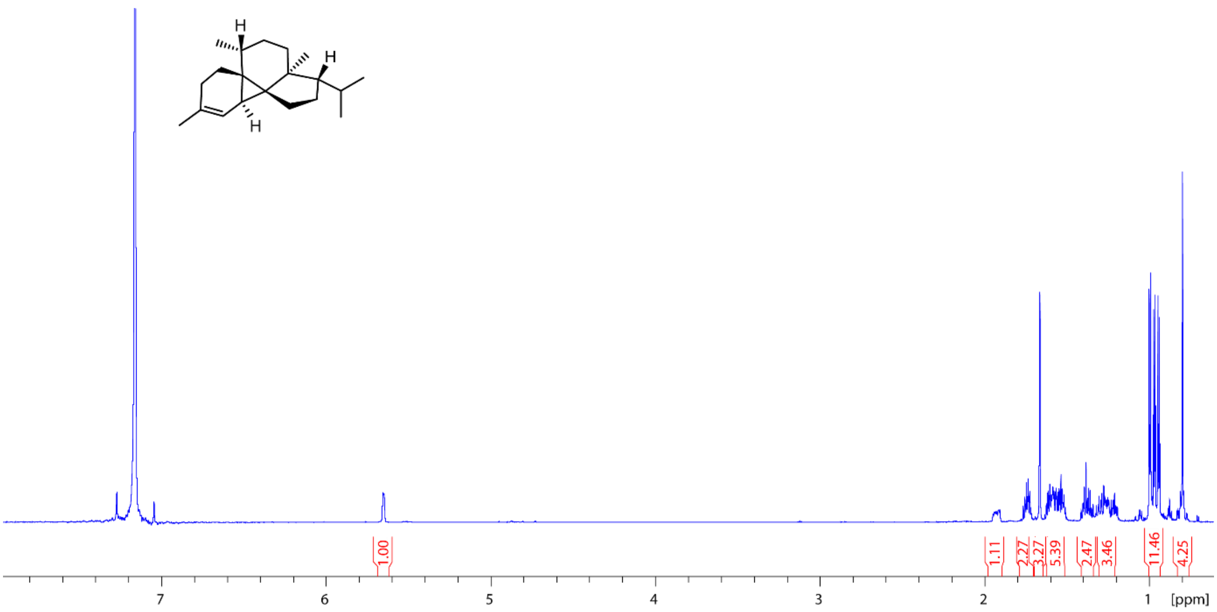

Figure S45. ^{1}H-NMR spectrum of **5** (700 MHz, C_6D_6).

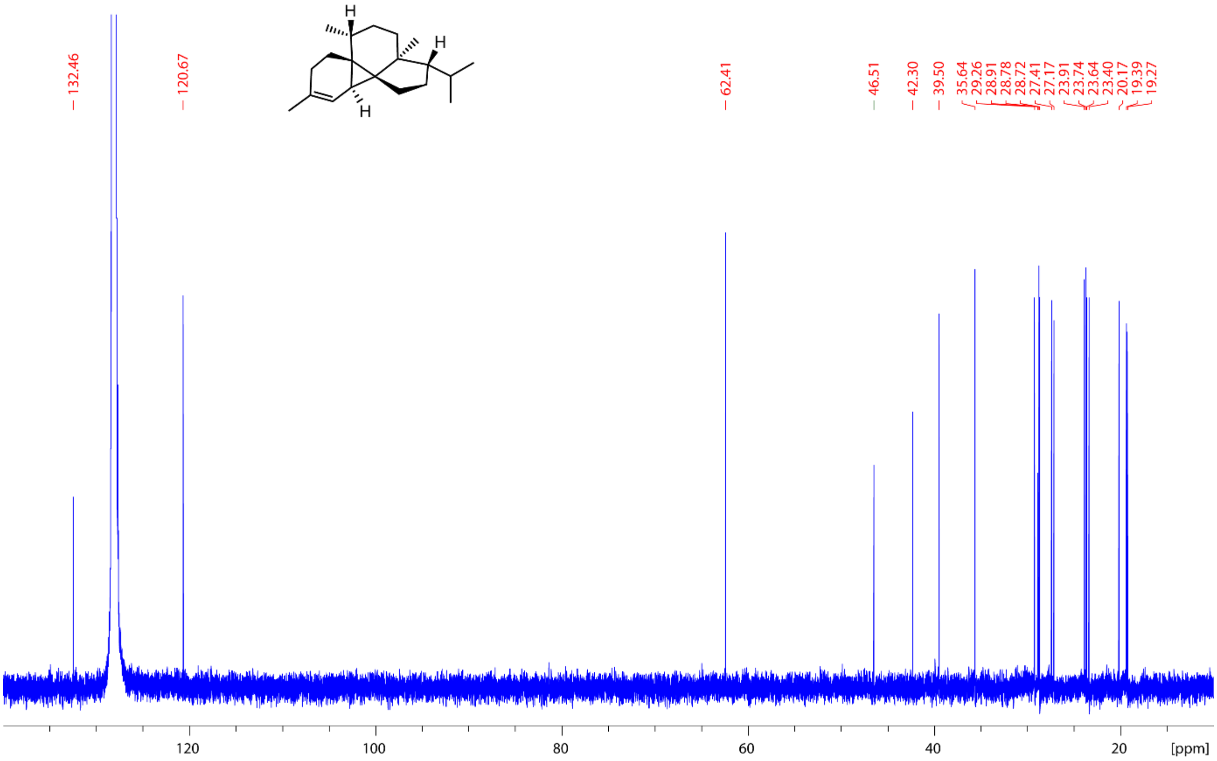

Figure S46. ^{13}C-NMR spectrum of **5** (176 MHz, C_6D_6).

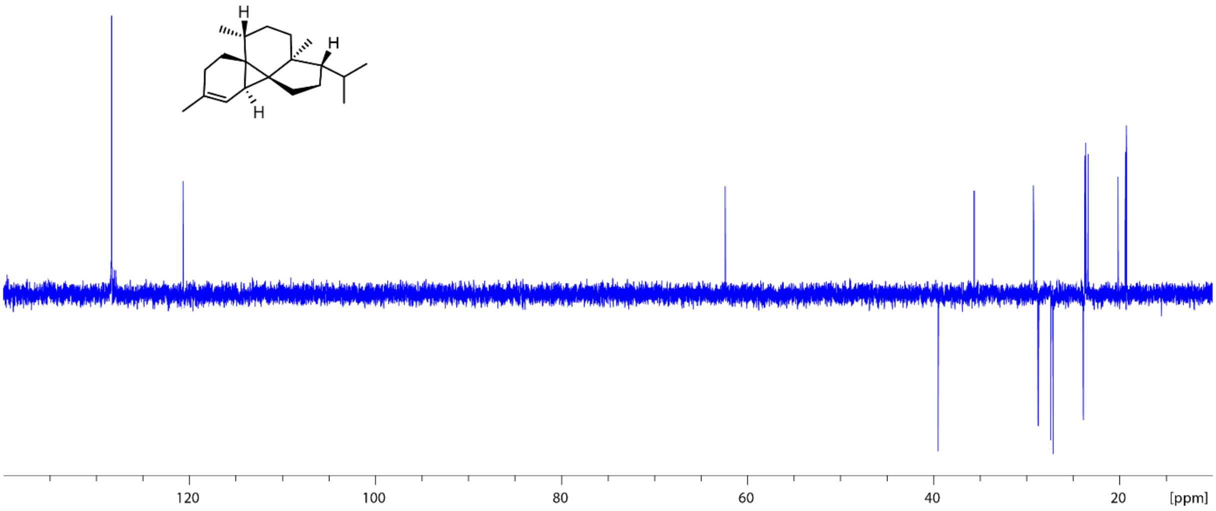

Figure S47. ^{13}C-DEPT135 spectrum of **5** (176 MHz, C_6D_6).

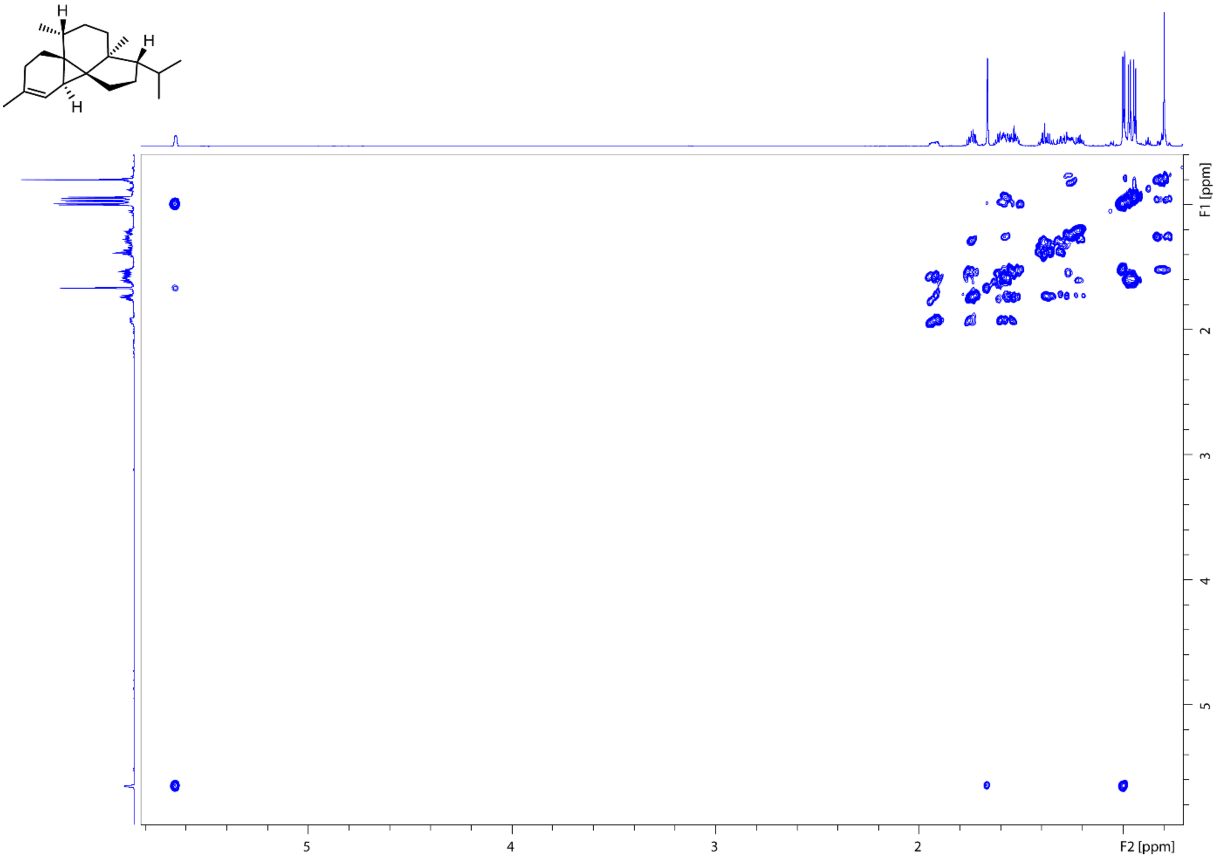

Figure S48. ^{1}H-^{1}H-COSY spectrum of **5** (700 MHz, C_6D_6).

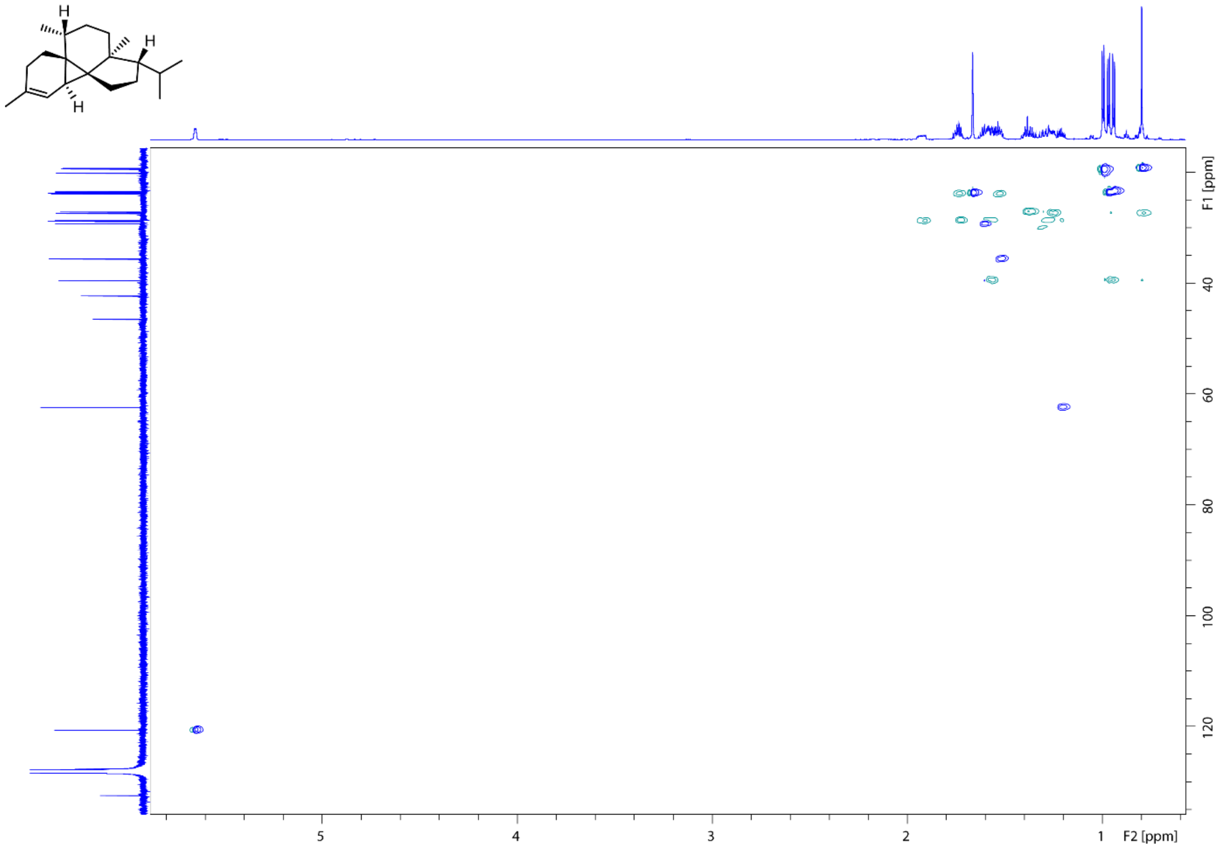

Figure S49. HSQC spectrum of **5** (700 MHz, C_6D_6).

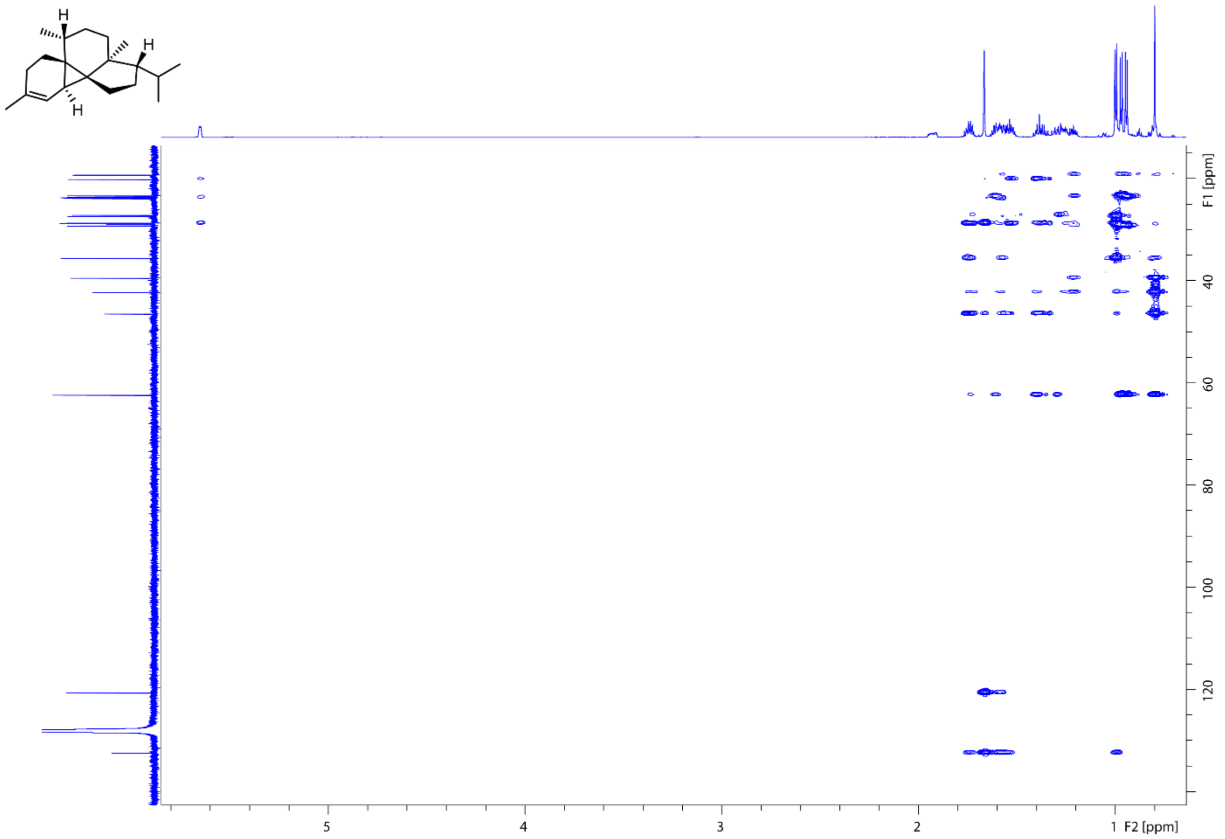

Figure S50. HMBC spectrum of **5** (700 MHz, C_6D_6).

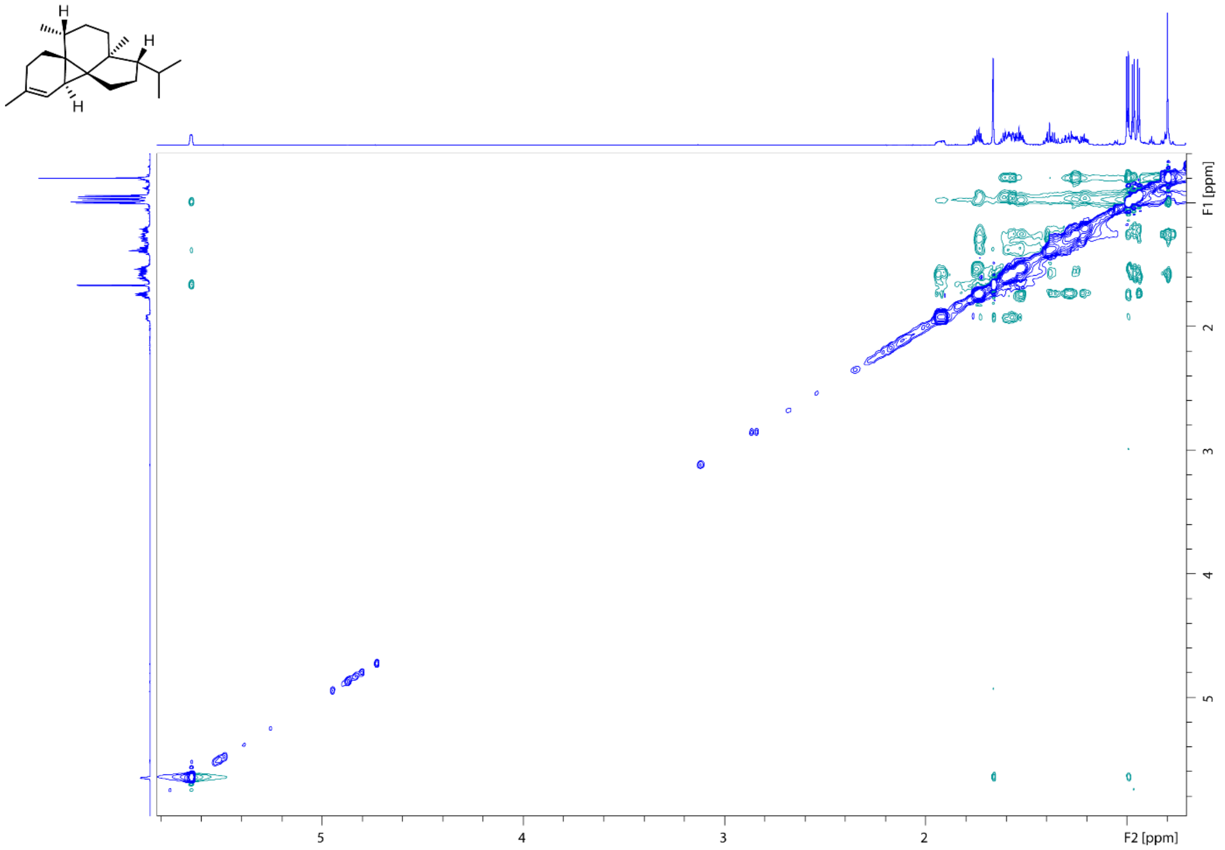

Figure S51. NOESY spectrum of **5** (700 MHz, C_6D_6).

Table S11. NMR data of **6** in C_6D_6 recorded at 298 K.

C[a]	type	^{13}C[b]	^{1}H[b]
1	CH	118.77	4.84 (m)
2	CH_2	45.29	2.22 (dd, *J* = 14.4, 10.4, H_β)<br>2.06 (m, H_α)
3	C_q	73.85	–
4	CH_2	42.78	1.63 (m, H_α)<br>1.58 (m, H_β)
5	CH_2	24.26	2.04 (m, H_β)<br>2.00 (m, H_α)
6	CH	128.18	4.96 (t, *J* = 7.9)
7	C_q	133.60	–
8	CH_2	34.99	2.07 (m, H_β)<br>1.98 (m, H_α)
9	CH_2	34.09	1.62 (m, H_β)<br>1.44 (m, H_α)
10	C_q	47.97	–
11	C_q	148.44	–
12	CH_2	30.00	2.33 (ddd, *J* = 16.5, 8.1, 2.0, H_α)<br>1.95 (m, H_β)
13	CH_2	27.08	1.75 (m, H_β)<br>1.21 (m, H_α)
14	CH	52.72	1.56 (m)
15	CH	28.67	1.58 (m)
16	CH_3	21.96	0.86 (d, *J* = 6.0 Hz)
17	CH_3	23.73	0.93 (d, overlap)
18	CH_3	25.16	0.94 (s)
19	CH_3	16.47	1.56 (s)
20	CH_3	28.24	1.16 (s)

[a] Carbon numbering as shown in Figure S52 indicates the origin of each carbon from GGPP by same number. [b] Chemical shifts δ in ppm. Multiplicity: s = singlet, d = doublet, m = multiplet. Coupling constants *J* are given in Hertz.

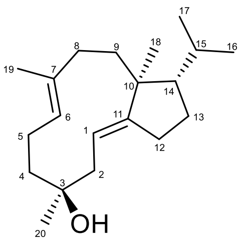

6

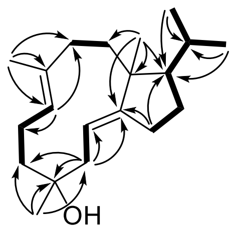

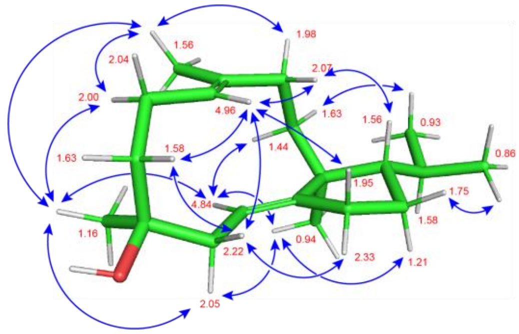

Figure S52. Structure elucidation of **6**. Bold: ^{1}H,^{1}H-COSY, single headed arrows: key HMBC, and blue double headed arrows: NOESY correlations.

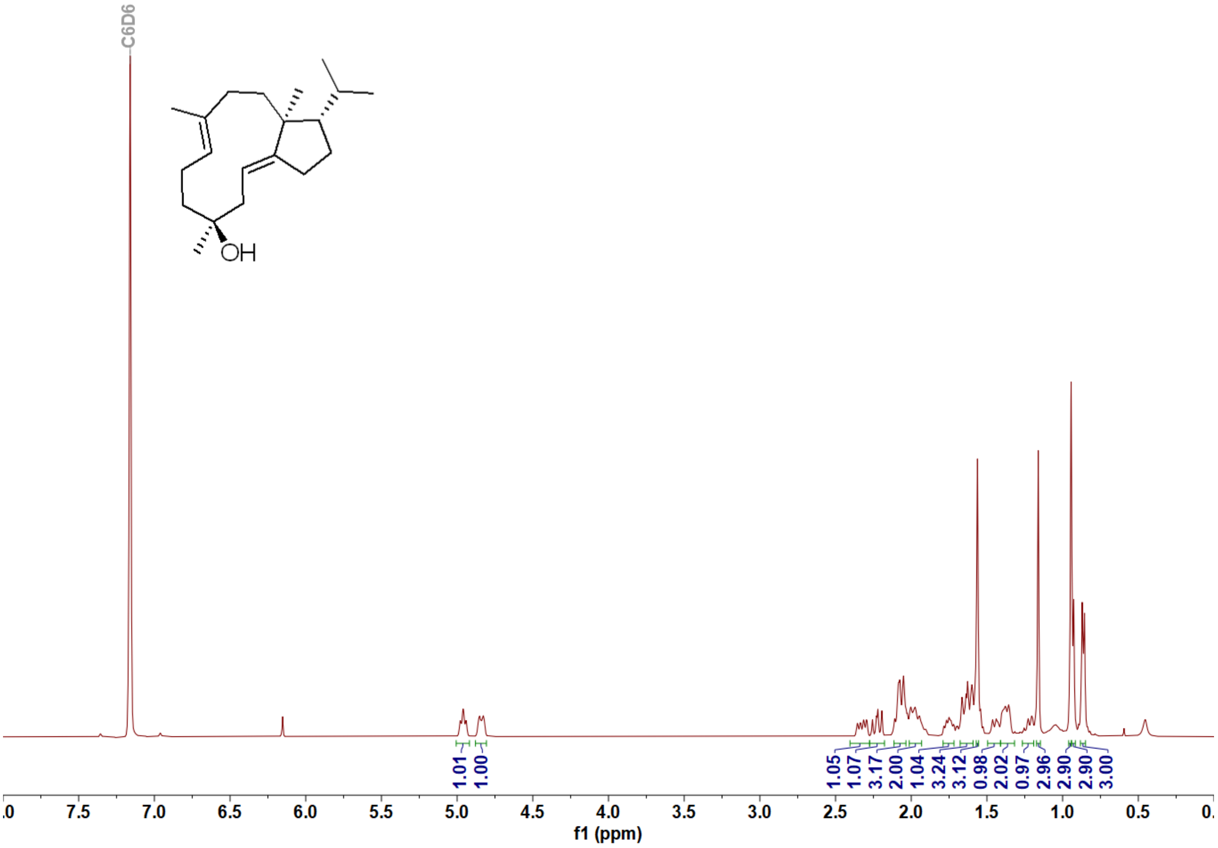

Figure S53. ^{1}H-NMR spectrum of **6** (400 MHz, C_6D_6).

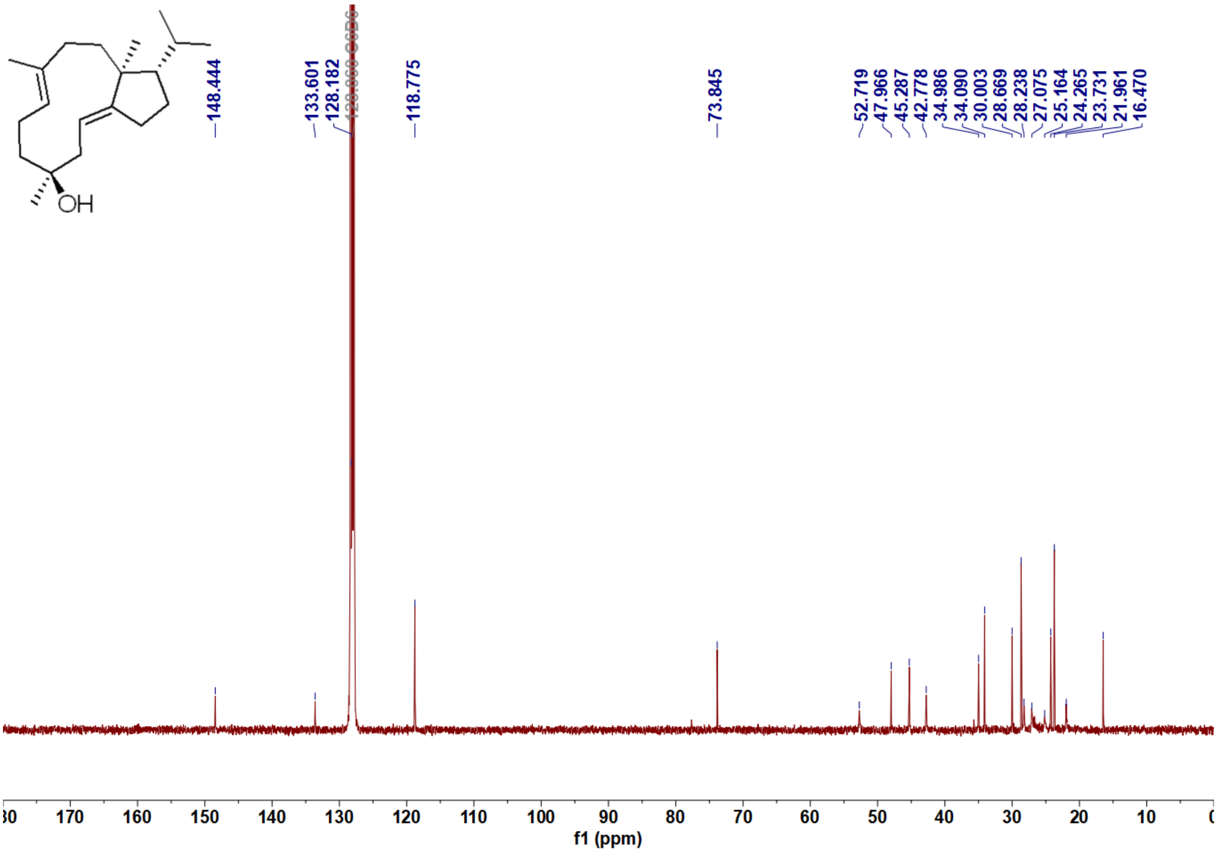

Figure S54. ^{13}C-NMR spectrum of **6** (101 MHz, C_6D_6).

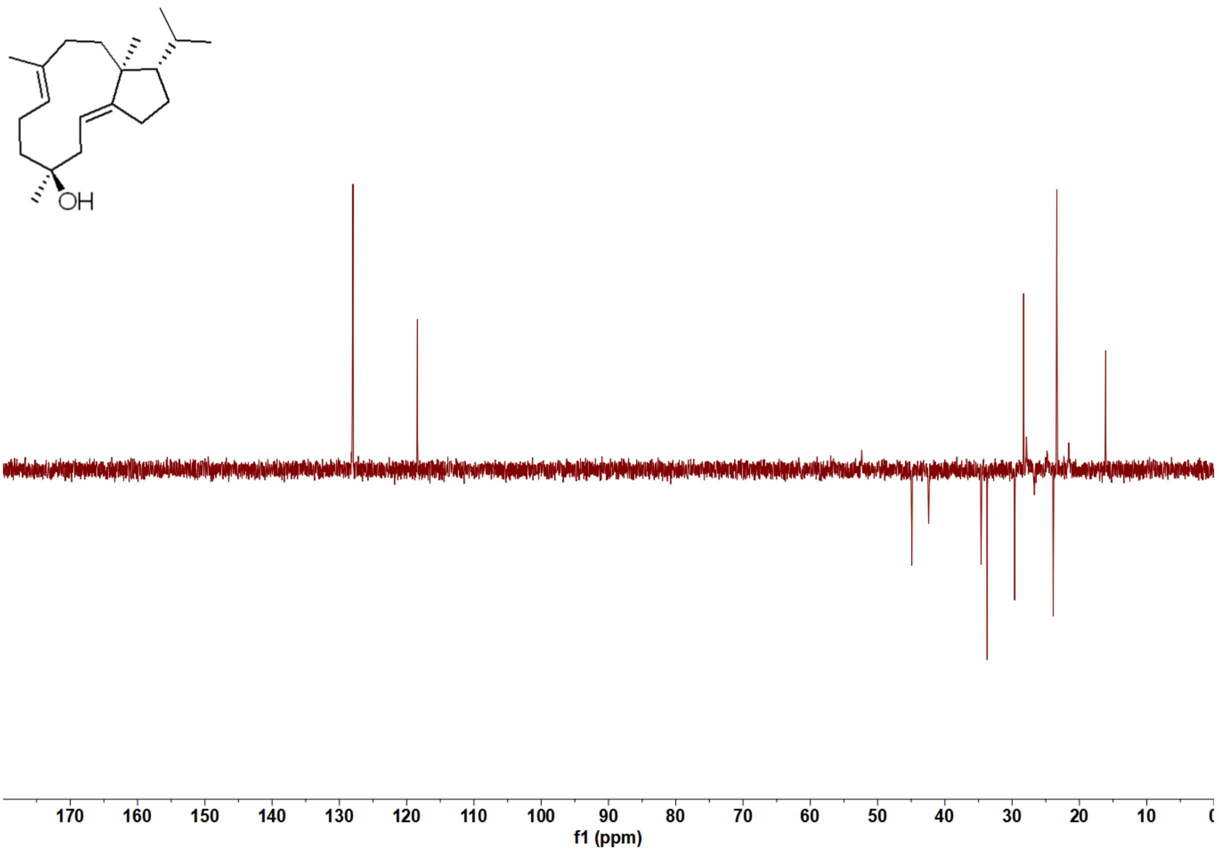

Figure S55. ^{13}C-DEPT135 spectrum of **6** (101 MHz, C_6D_6).

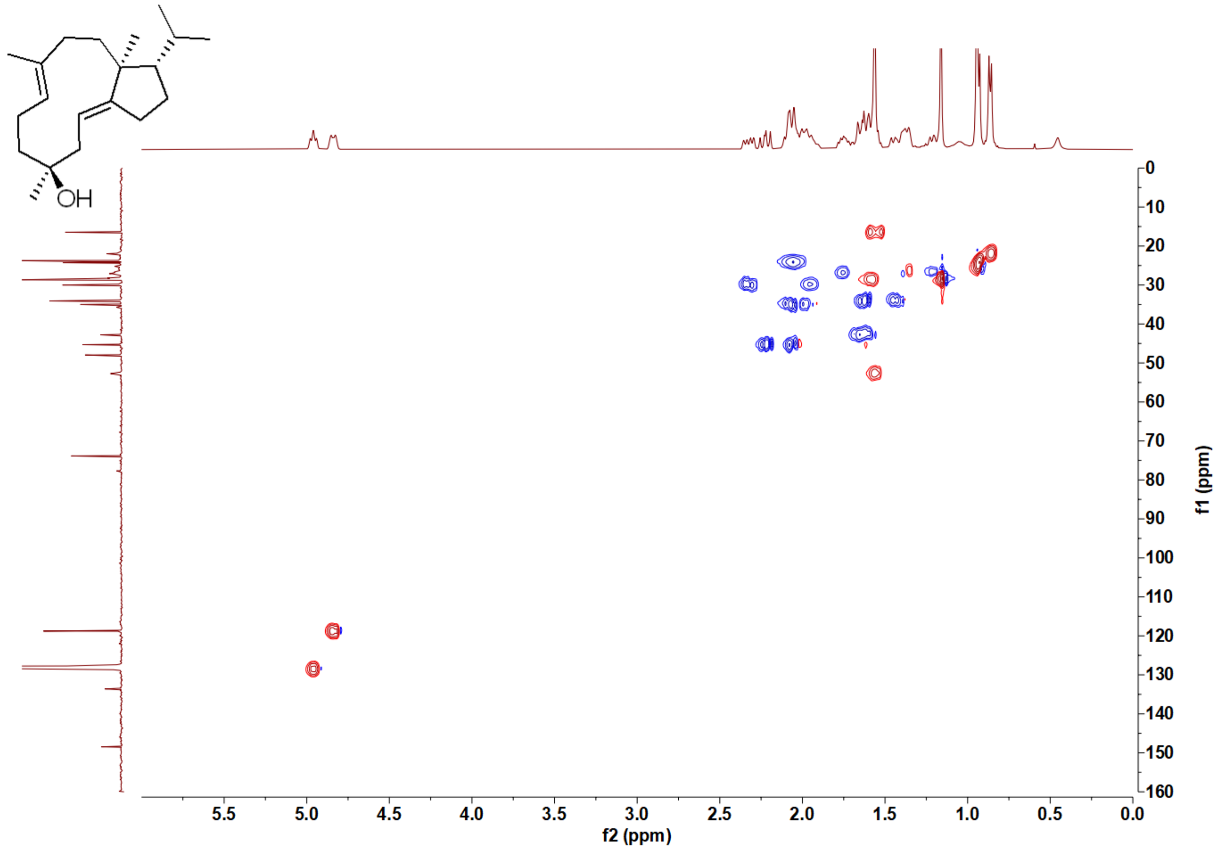

Figure S56. HSQC spectrum of **6** (400 MHz, C_6D_6).

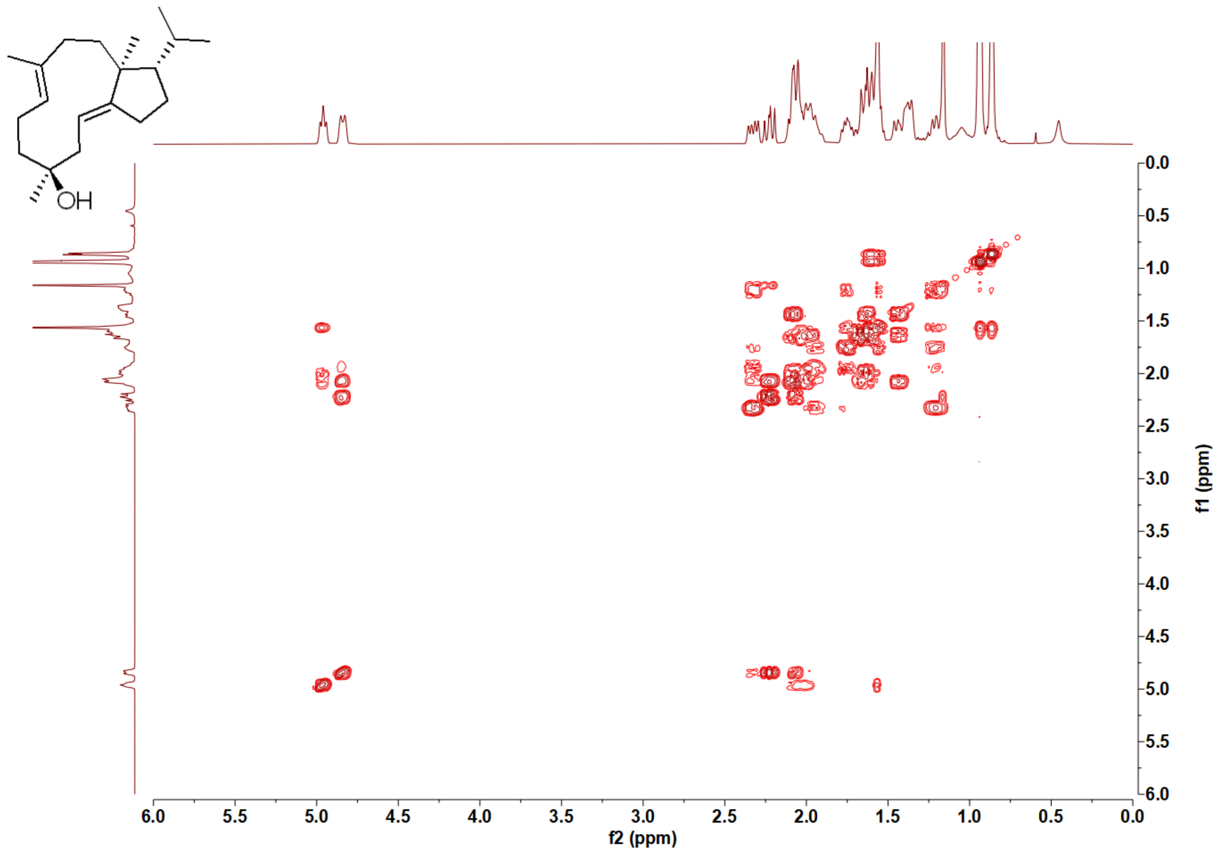

Figure S57. ^{1}H-^{1}H-COSY spectrum of **6** (400 MHz, C_6D_6).

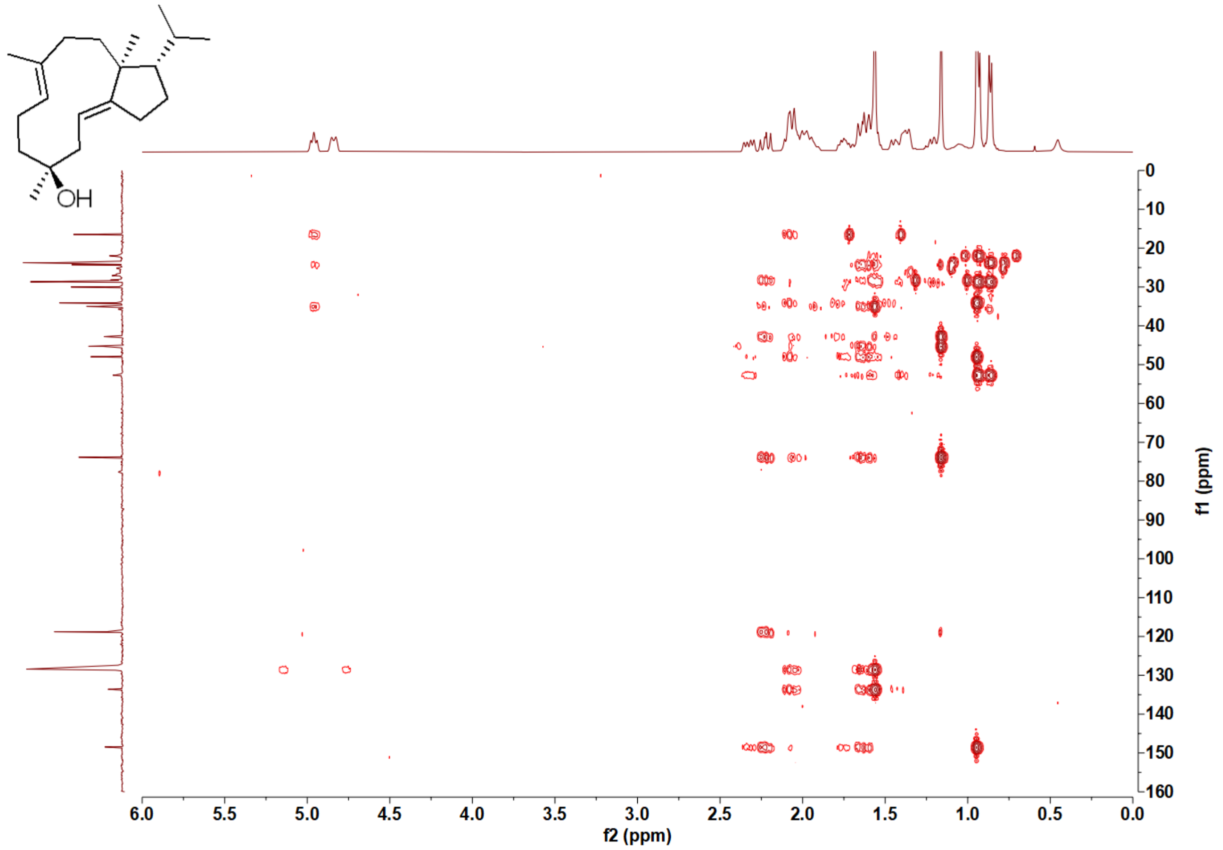

Figure S58. HMBC spectrum of **6** (400 MHz, C_6D_6).

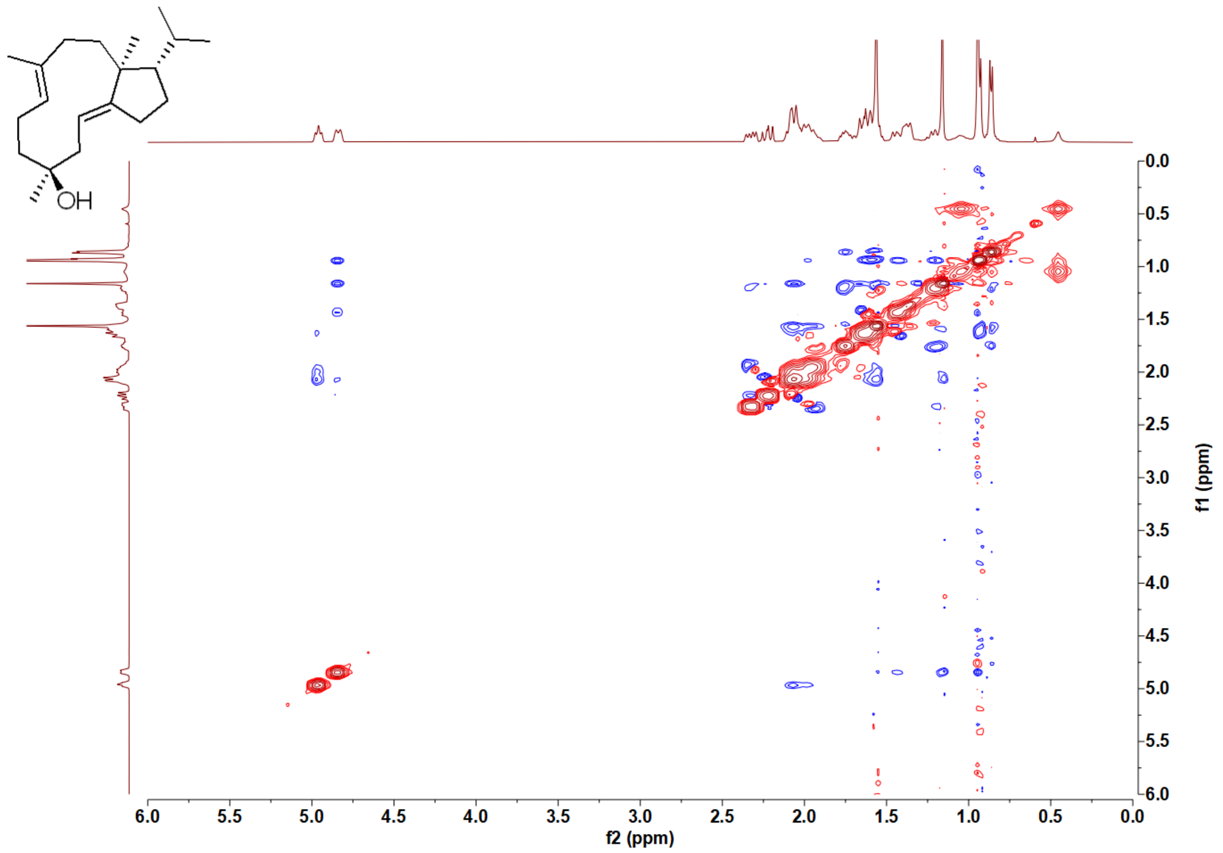

Figure S59. NOESY spectrum of **6** (400 MHz, C_6D_6).

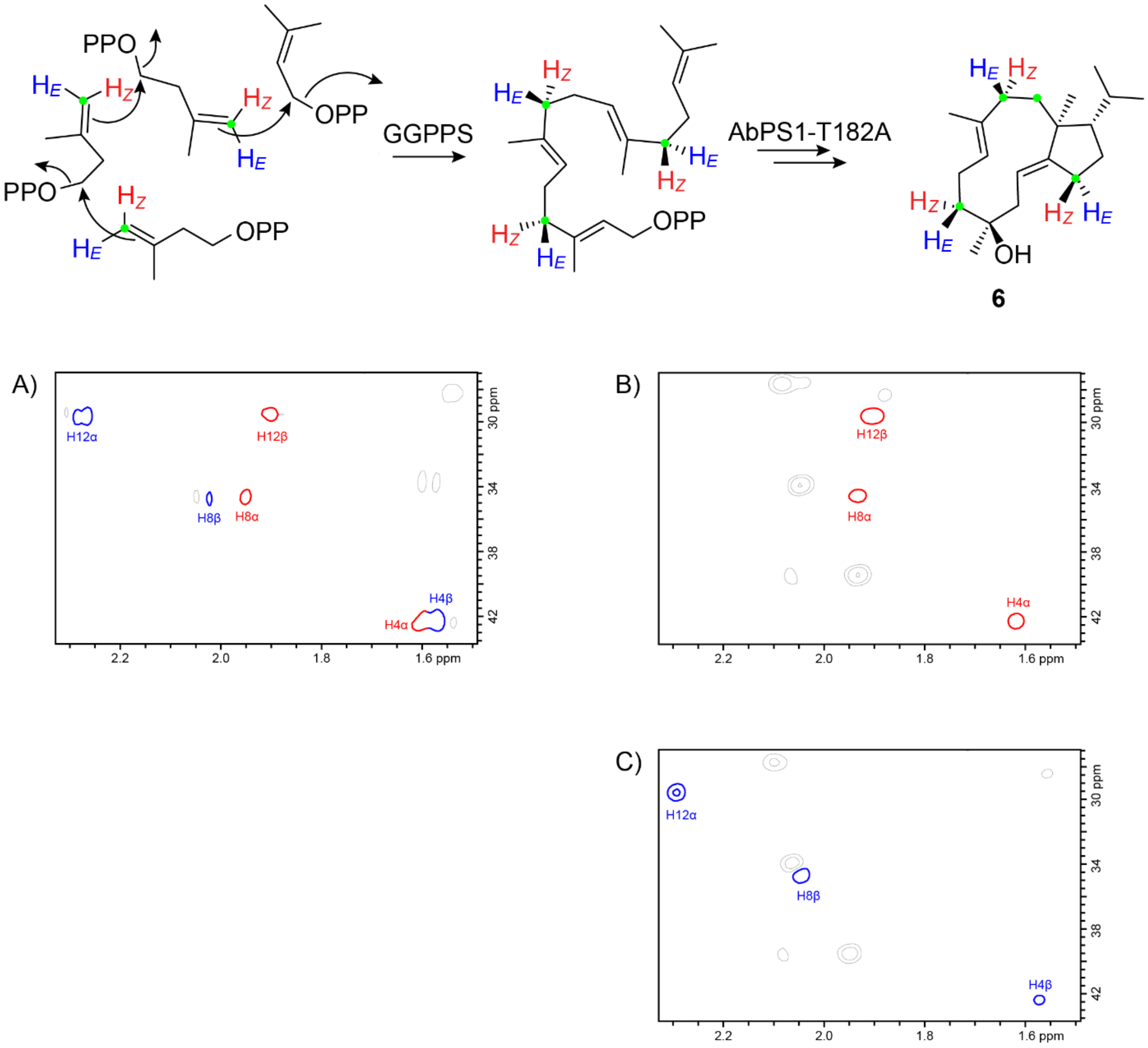

Figure S60. The absolute configuration of **6**. Partial HSQC spectra of A) unlabelled **6**, B) labelled **6** obtained from DMAPP and (*E*)-(4-^{13}C,4-^{2}H)IPP (blue H = ^{2}H) incubated with GGPPS and AbPS1-T182A and C) labelled **6** obtained from DMAPP and (*Z*)-(4-^{13}C,4-^{2}H)IPP (red H = ^{2}H) incubated with GGPPS and AbPS1-T182A. The specific incorporation at C4, C8 and C12 with known configuration at these carbons in experiments B) and C) together with the NOESY based assignments of relative orientations of H4α, H4β, H8α, H8β, H12α, and H12β (Figure S52) with respect to the naturally present stereogenic centers in **6** allows to assign the shown absolute configuration. Green dots represent ^{13}C-labelled carbons.

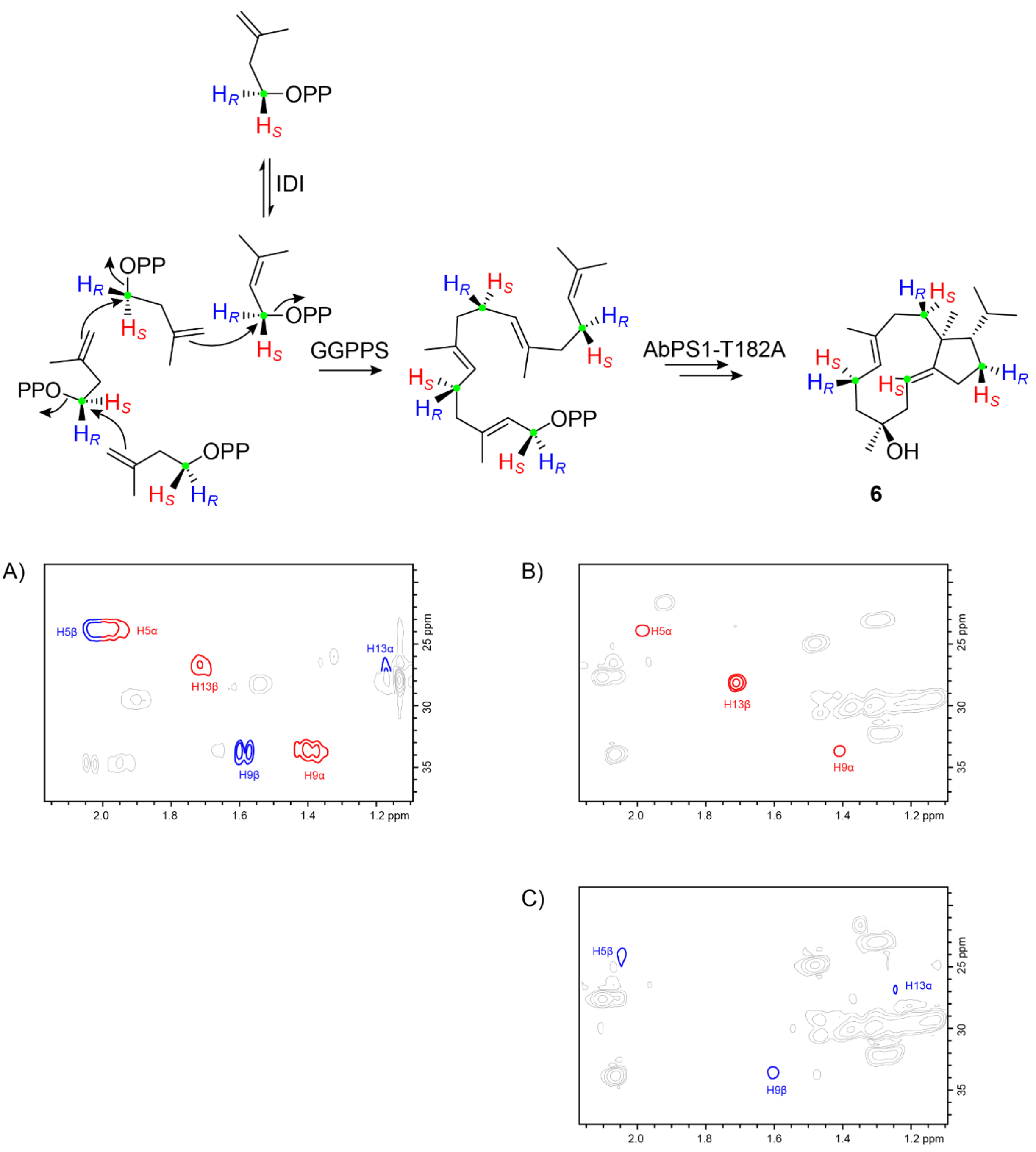

Figure S61. The absolute configuration of **6**. Partial HSQC spectra of A) unlabelled **6**, B) labelled **6** obtained from (*R*)-(1-^{13}C,1-^{2}H)IPP (blue H = ^{2}H) incubated with IDI, GGPPS and AbPS1-T182A and C) labelled **6** obtained from (*S*)-(1-^{13}C,1-^{2}H)IPP (red H = ^{2}H) incubated with IDI, GGPPS and AbPS1-T182A. The specific incorporation at C5, C9 and C13 with known configuration at these carbons in experiments B) and C) together with the NOESY based assignments of relative orientations of H5α, H5β, H9α, H9β, H13α and H13β (Figure S2) with respect to the naturally present stereogenic centers in **6** allows to assign the shown absolute configuration. Green dots represent ^{13}C-labelled carbons.

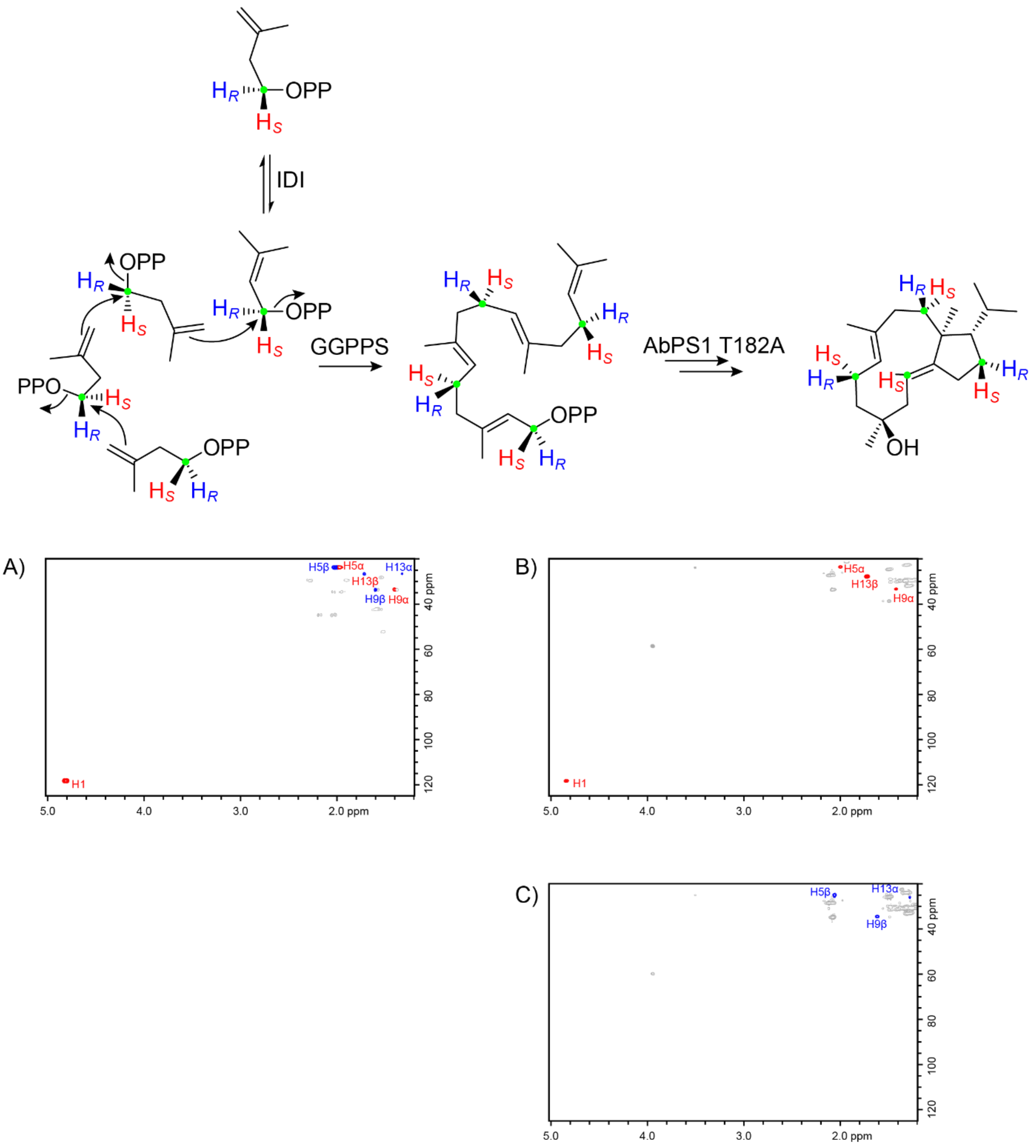

Figure S62. The loss of the 1-*pro*-*R* hydrogen in the biosynthesis of **6**. Green dots represent ^{13}C-labelled carbons. Partial HSQC spectra of A) unlabelled **6**, B) labelled **6** obtained from (*R*)-(1-^{13}C,1-^{2}H)IPP (blue H = ^{2}H) incubated with IDI, GGPPS and AbPS1-T182A and C) labelled **6** obtained from (*S*)-(1-^{13}C,1-^{2}H)IPP (red H = ^{2}H) incubated with IDI, GGPPS and AbPS1-T182A. The signal for H1 in B) indicates the loss of the 1-*pro*-*R* hydrogen.

Table S12. NMR data of anaerol B (**7**) in C_6D_6 recorded at 298 K.

C[a]	type	^{13}C[b]	^{1}H[b]
1	CH	49.93	1.57 (m)
2	CH	42.67	2.27 (ddd, J = 7.0, 7.0, 7.0)
3	CH	37.53	1.70 (m)
4	CH$_2$	34.68	1.61 (m)
			1.30 (m)
5	CH$_2$	27.16	1.40 (m, H$_\beta$)
			1.32 (m, H$_\alpha$)
6	CH	43.72	2.12 (dd, J = 8.0, 8.0)
7	C$_q$	34.72	–
8	CH$_2$	34.39	1.66 (m, H$_\alpha$)
			1.26 (m, H$_\beta$)
9	CH$_2$	35.22	1.81 (m, H$_\beta$)
			1.63 (m, H$_\alpha$)
10	C$_q$	46.37	–
11	C$_q$	84.25	–
12	CH$_2$	38.52	1.69 (m, H$_\beta$)
			1.36 (m, H$_\alpha$)
13	CH$_2$	27.22	1.65 (m, H$_\alpha$)
			1.61 (m, H$_\beta$)
14	CH	60.96	1.32 (m)
15	CH	29.96	1.57 (m)
16*	CH$_3$	23.07	0.99 (d, J = 6.6)
17*	CH$_3$	23.45	0.91 (d, J = 6.6)
18	CH$_3$	17.83	0.97 (s)
19	CH$_3$	23.95	0.95 (s)
20	CH$_3$	15.04	0.95 (d, J = 6.6)

[a] Carbon numbering as shown in Figure S63 indicates the origin of each carbon from GGPP by same number. Asterisks indicate geminal Me groups for which signals may be interchanged. [b] Chemical shifts δ in ppm. Multiplicity: s = singlet, d = doublet, m = multiplet. Coupling constants J are given in Hertz.

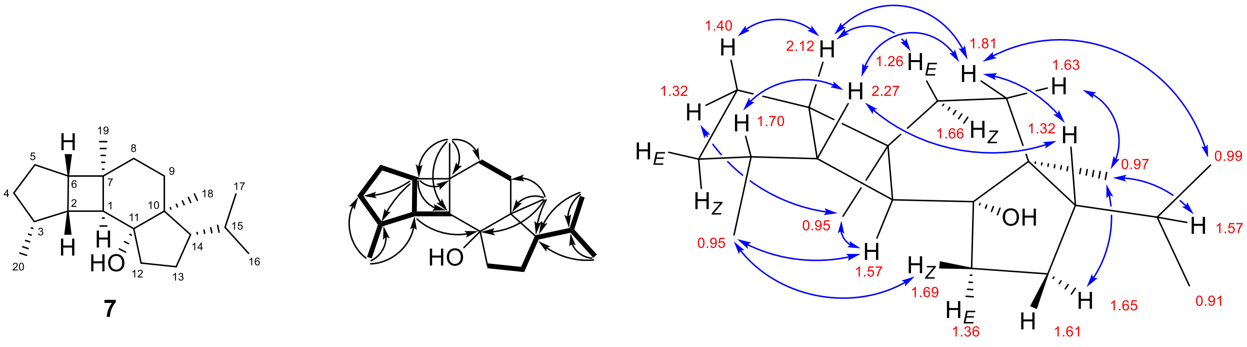

Figure S63. Structure elucidation of anaerol B (**7**). Bold: ^{1}H,^{1}H-COSY, single headed arrows: key HMBC, and blue double headed arrows: NOESY correlations. H$_E$ and H$_Z$ indicate the results from stereoselective labelling experiments (Figure S71).

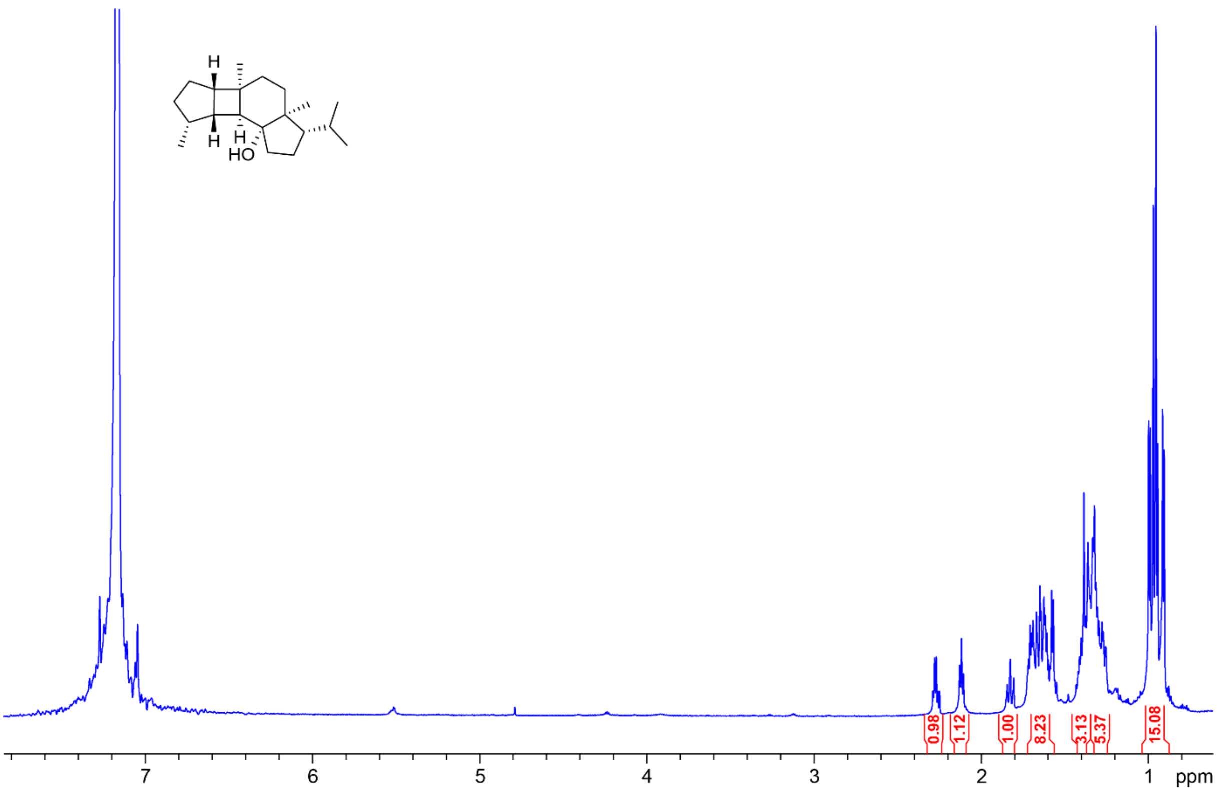

Figure S64. ^{1}H-NMR spectrum of **7** (700 MHz, C_6D_6).

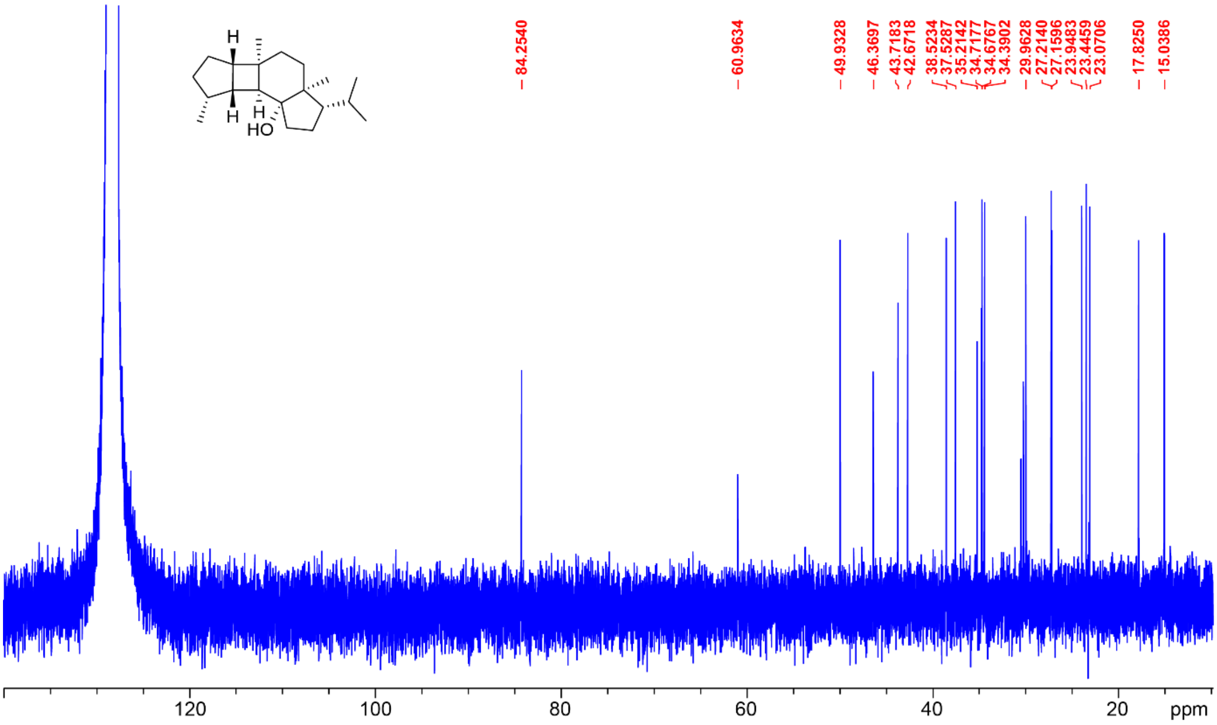

Figure S65. ^{13}C-NMR spectrum of **7** (176 MHz, C_6D_6).

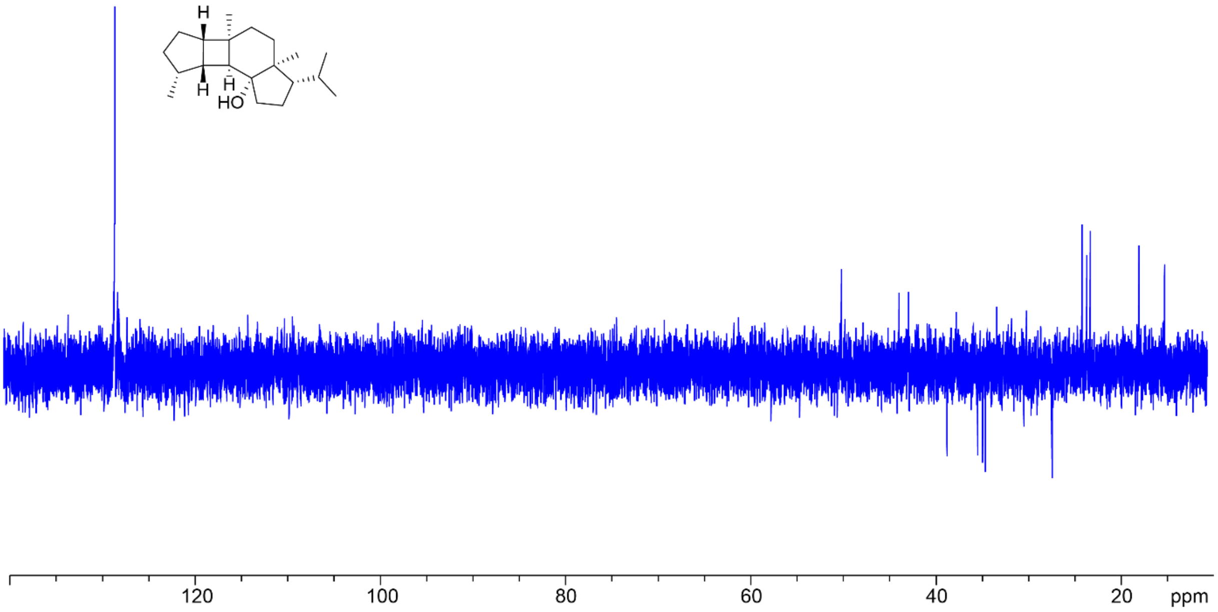

Figure S66. ^{13}C-DEPT135 spectrum of **7** (176 MHz, C_6D_6).

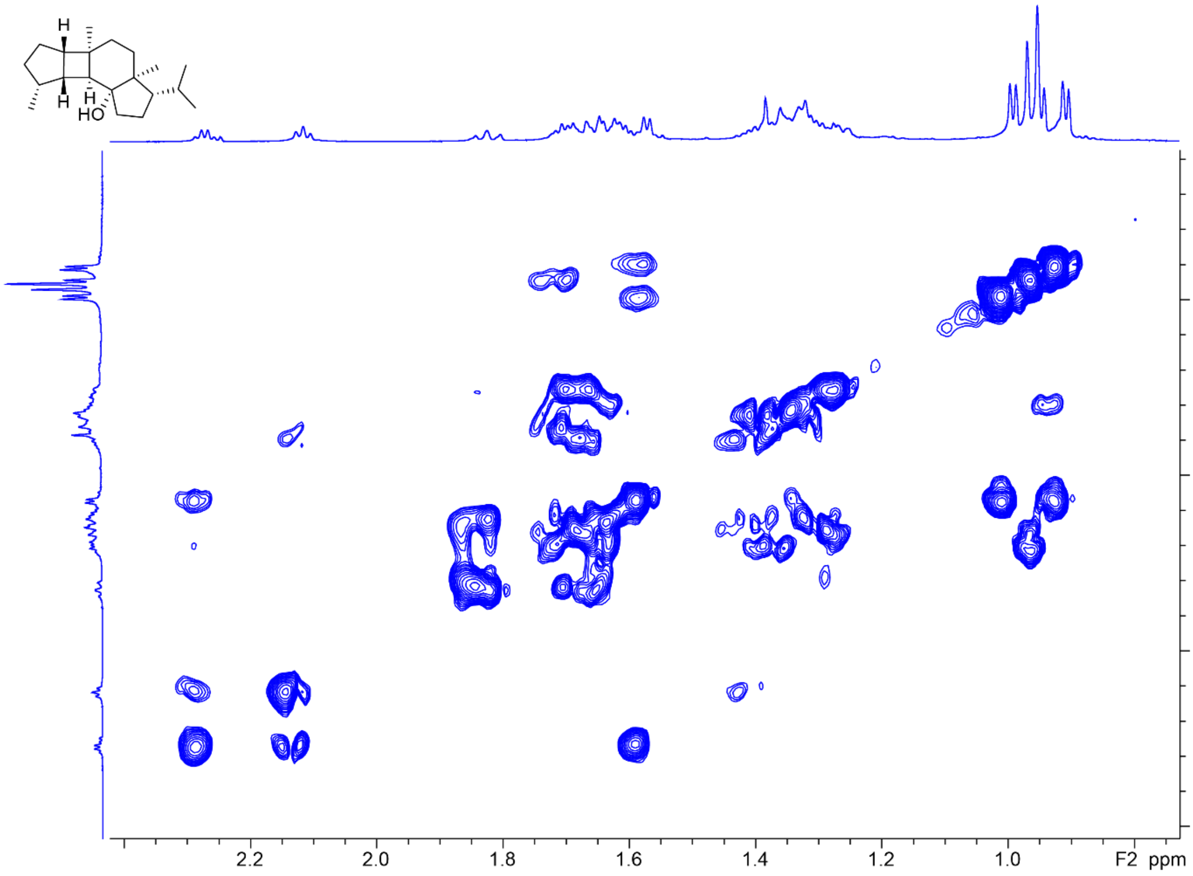

Figure S67. ^{1}H,^{1}H-COSY spectrum of **7** (700 MHz, C_6D_6).

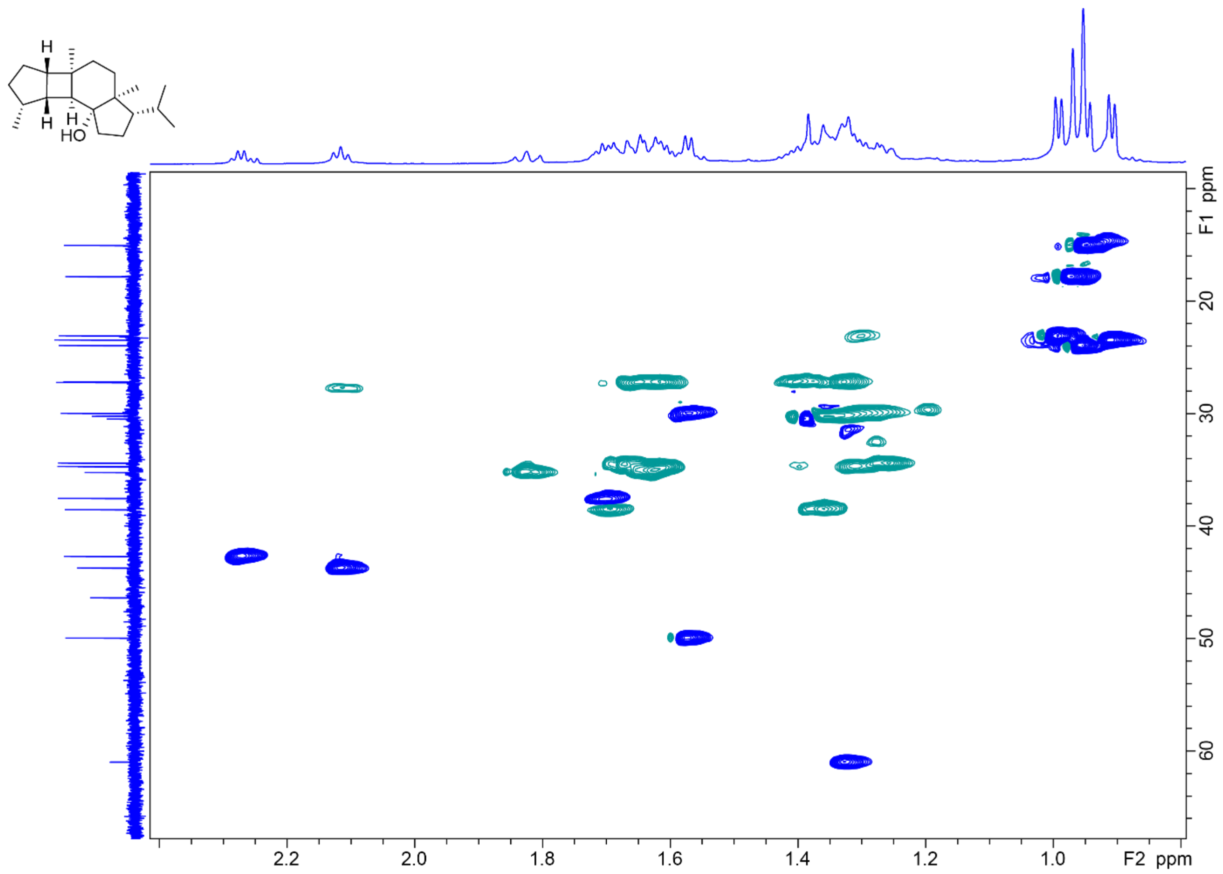

Figure S68. HSQC spectrum of **7** (700 MHz, C_6D_6).

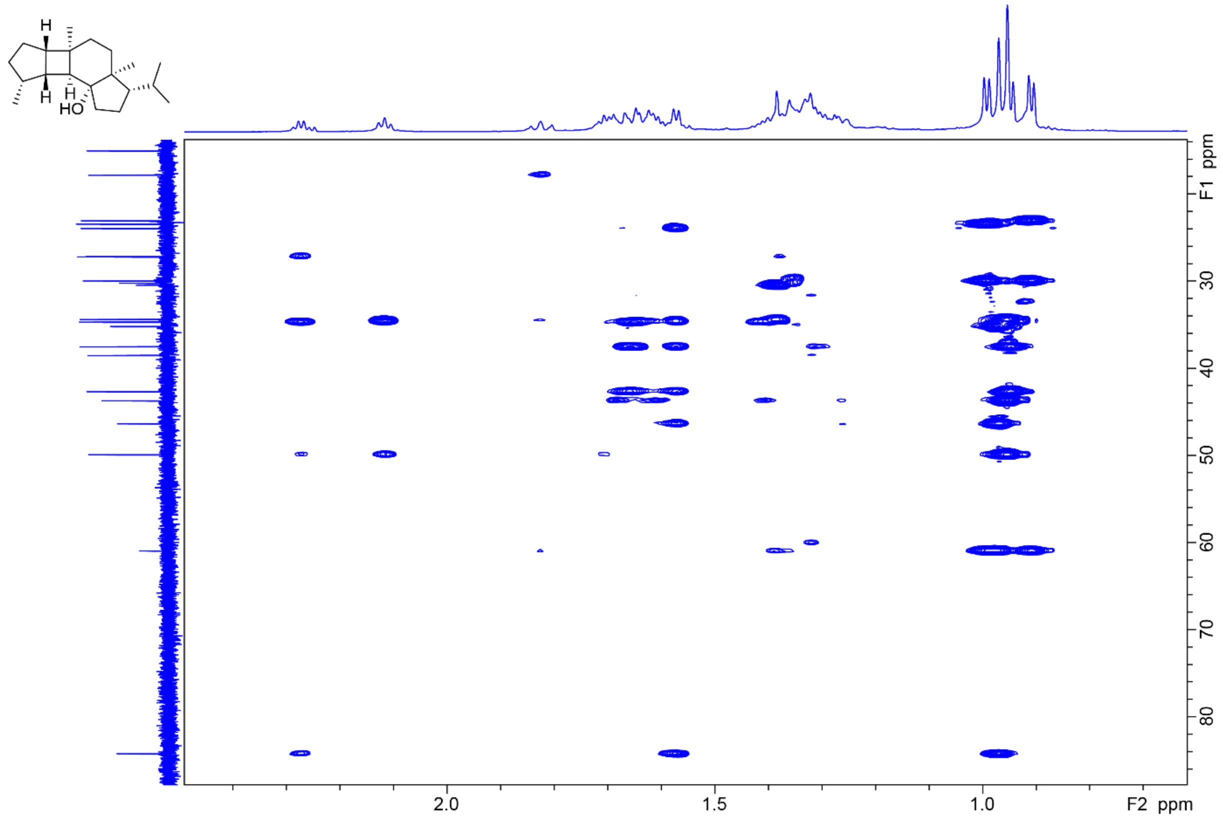

Figure S69. HMBC spectrum of **7** (700 MHz, C_6D_6).

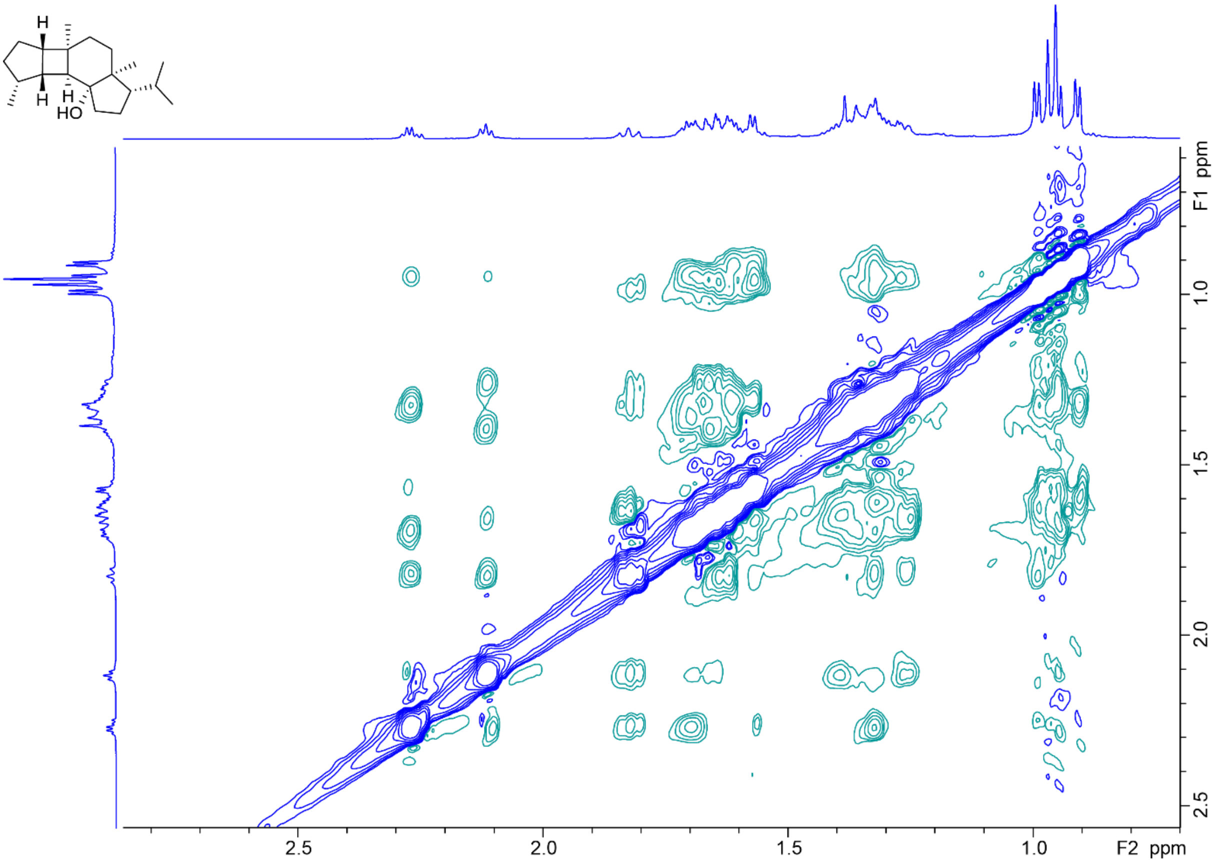

Figure S70. NOESY spectrum of **7** (700 MHz, C_6D_6).

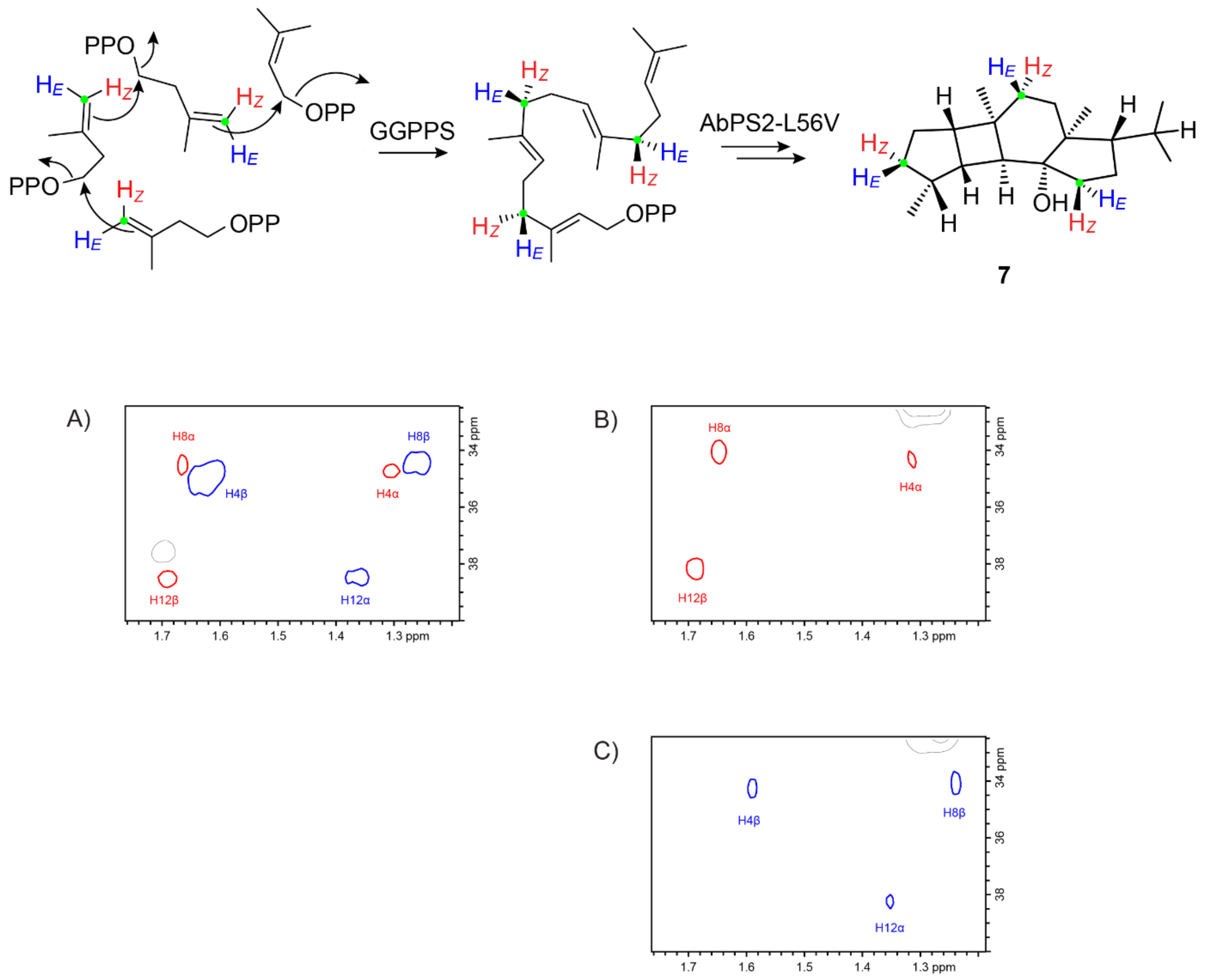

Figure S71. The absolute configuration of **7**. Partial HSQC spectra of A) unlabelled **7**, B) labelled **7** obtained from DMAPP and (*E*)-(4-^{13}C,4-^{2}H)IPP (blue H = ^{2}H) incubated with GGPPS and AbPS2-L56V/F75A and C) labelled **7** obtained from DMAPP and (*Z*)-(4-^{13}C,4-^{2}H)IPP (red H = ^{2}H) incubated with GGPPS and AbPS2-L56V/F75A. The specific incorporation at C4, C8 and C12 with known configuration at these carbons in experiments B) and C) together with the NOESY based assignments of relative orientations of H4α, H4β, H8α, H8β, H12α and H12β (Figure S63) with respect to the naturally present stereogenic centers in **7** allows to assign the shown absolute configuration. Green dots represent ^{13}C-labelled carbons.

Table S13. Results of DFT calculations for the cyclisation cascade from GGPP to **7** (Scheme 2 of main text).

Structure[a]	Gibbs energy (298.15K) in Hartree	energy relative to A' in kcal/mol	reaction barrier in kcal/mol	Gibbs free energy in kcal/mol
**A'**	–781.674472	0.00		
**A'-C'-TS**	–781.674200	0.17	0.17	
**C'**	–781.717088	–26.74		–26.74
**C'**	–781.717081	–26.74		
**C'-D'-TS**	–781.703422	–18.17	8.57	
**D'**	–781.726115	–32.41		–5.67
**D'**	–781.719808	–28.45		
**D'-E'-TS**	–781.712621	–23.94	4.51	
**E'**	–781.720285	–28.75		–0.30
**E'**	–781.719130	–28.02		
**E'-F'-TS**	–781.707808	–20.92	7.10	
**F'**	–781.713593	–24.55		3.47
**F'**	–781.709261	–21.83		
**F'-G'-TS**	–781.697086	–14.19	7.64	
**G'**	–781.724721	–31.53		–9.70
**G'***	–781.733464	–37.02		
**G'-H'-TS***	–781.735422	–38.25	–1.23	
**H'***	–781.734945	–37.95		–0.93
**H'***	not localised[b]			
**H'-I'-TS***	–781,735196	–38.10		
**I'***	–781,746982	–45.50		
**I'**	–781.731107	–35.54		
**I'-N'-TS**	–781.719552	–28.29	7.25	
**N'**	–781.761497	–54.61		–19.07
**H'***	–781.734258	–37.52		
**H'-H-TS***	–781.690833	–10.27	27.25	
**H***	–781.718189	–27.43		10.08

[a] Intermediates and transition states marked with an asterisk have been computed with NH_3 as surrogate base, and the given Gibbs energies equal the computed Gibbs energies reduced by –56.563161 Hartree (computed Gibbs energy of NH_3) to allow for a direct comparison to the computed energies for steps without NH_3. [b] Because of the high reactivity of diene **H'** towards NH_4^+, **H'*** could not be localised. The approximated transition state did not fully converge and **H'-I'-TS*** is an estimate for a barrierless reaction.

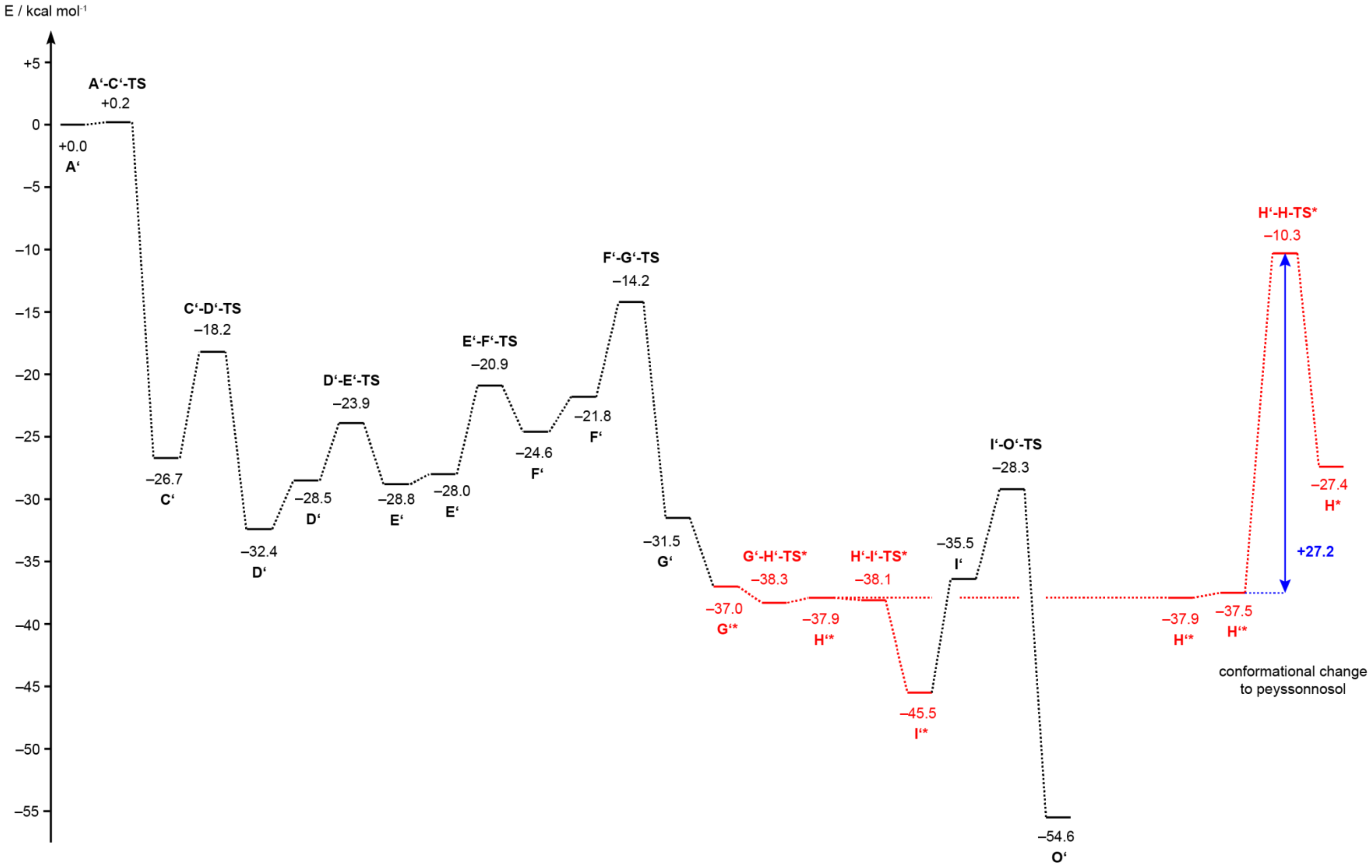

Figure S72. Computed energy profile for the transformations of **A’** to **O’** (Scheme 2 of main text, mPW1PW91/6-311+G(d,p)//B97D3/6-31g(d,p), 298 K). Intermediates and transition states marked with an asterisk and shown in red have been computed with NH_3 as surrogate base. The blue arrow shows a reaction barrier that is too high to explain the biosynthesis of **3** via this mechanism.

Table S14. Results of DFT calculations for the alternative cyclisation cascade from GGPP through (*R*)-GLPP to **3 – 5** (Scheme 3 of main text).

Structure[a]	Gibbs energy (298.15K) in Hartree	energy relative to A in kcal/mol	reaction barrier in kcal/mol	Gibbs free energy in kcal/mol
**A”**	–781.685176	0.00		
**A”-C”-TS**	–781.683893	0.81	0.81	
**C”**	–781.719900	–21.79		–21.79
**C”**	–781.719898	–21.79		
**C”-D”-TS**	–781.706221	–13.21	8.58	
**D”**	–781.727285	–26.42		–4.64
**D”**	–781.711576	–16.57		
**D”-E”-TS**	–781.707253	–13.85	2.71	
**E”**	–781.714289	–18.27		–1.70
**E”**	–781.709203	–15.08		
**E”-F”-TS**	–781.704503	–12.13	2.95	
**F”**	–781.724156	–24.46		–9.38
**F”***	–781.732918	–29.96		
**F”-H”-TS***	–781.712647	–17.24	12.72	
**H”***	–781.739808	–34.28		–4.32
**H”***	–781.740827	–34.92		
**H”-I-TS***	–781.723391	–23.98	10.94	
**I***	–781.733067	–30.05		4.87
**I**	–781.725886	–25.55		
**I-J-TS**	–781.727175	–26.35	–0.81	
**J**	–781.734031	–30.66		–5.11
**J**	–781.734032	–30.66		
**J-K-TS**	–781.730340	–28.34	2.32	
**K**	–781.751201	–41.43		–10.77
**K**	–781.751204	–41.43		
**K-L-TS**	–781.754319	–43.39	–1.95	
**L**	–781.756135	–44.53		–3.09
**L**	–781.759897	–46.89		
**L-M-TS**	–781.738678	–33.57	13.32	
**M**	–781.744058	–36.95		9.94

[a] Intermediates and transition states marked with an asterisk have been computed with NH_3 as surrogate base, and the given Gibbs energies equal the computed Gibbs energies reduced by –56.563161 Hartree (computed Gibbs energy of NH_3) to allow for a direct comparison to the computed energies for steps without NH_3.

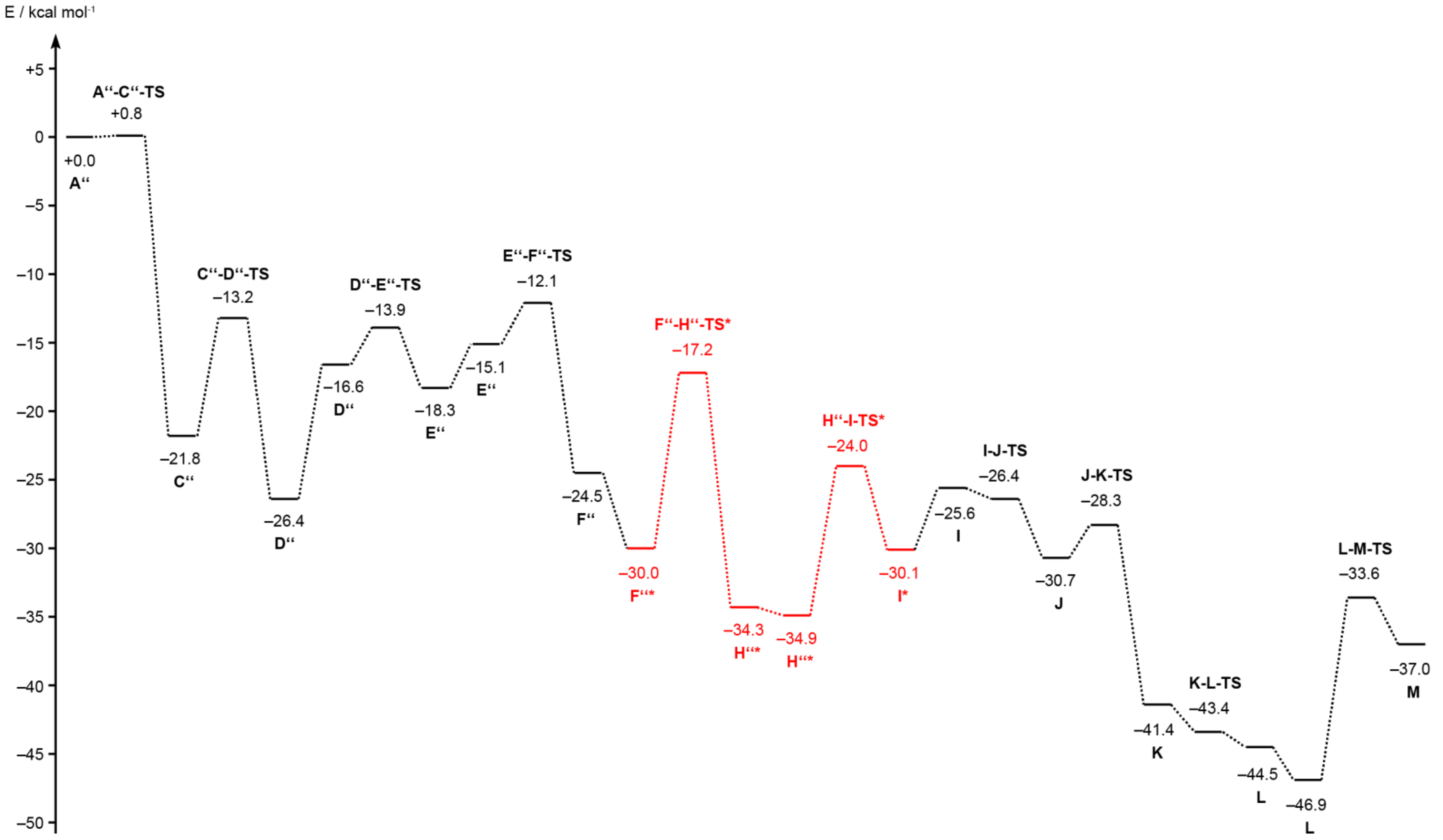

Figure S73. Computed energy profile for the transformations of **A’’** to **M** (Scheme 3 of main text, mPW1PW91/6-311+G(d,p)//B97D3/6-31g(d,p), 298 K). Intermediates and transition states marked with an asterisk and shown in red have been computed with NH_3 as surrogate base.

Cartesian coordinates of computed structures (Scheme 1 of main text, Table S7)

Gibbs energies (G"..." in Hartree) and imagininary frequencies of TS (T-"..." in cm^{-1}), mPW1PW91/6-311+G(d,p)//B97D3/6-31G(d,p)-sp-density-fitting, 1 bar, 298.15 K. From the structures labelled with asterisks the energy of NH_3 (G56.563161, page 98) is subtracted for direct comparability.

**A**	G781.679303		
C	-3.308470	0.712698	1.373169
C	-2.936840	1.761386	0.251929
C	-1.891921	1.246769	-0.717362
C	1.605517	-1.833414	0.163508
C	-1.833173	-1.274416	1.251619
C	-2.077031	-0.035395	1.802095
H	-3.872143	2.036915	-0.256374
H	-2.562220	2.674544	0.733907
H	-3.767311	1.247623	2.217156
H	-4.048177	0.008017	0.972548
C	-1.105846	0.699701	2.677675
H	-0.298494	0.075401	3.075017
H	-0.644384	1.521952	2.105718
H	-1.629946	1.171479	3.520684
C	-0.615188	-2.011812	1.359387
H	-2.597503	-1.683820	0.589778
C	-2.043860	0.247965	-1.612389
H	-0.891686	1.681361	-0.617395
C	-0.837769	-0.330072	-2.329511
H	-0.034508	0.417395	-2.403509
H	-1.104835	-0.623921	-3.355840
C	-3.364659	-0.419119	-1.915141
H	-3.340332	-1.497432	-1.686143
H	-4.209681	0.019732	-1.373697
H	-3.583948	-0.345229	-2.991447
C	0.450078	-1.214137	-0.322112
C	-0.275852	-1.590654	-1.604079
H	-1.094735	-2.301626	-1.415913
H	0.424433	-2.107188	-2.276893
C	2.503080	-1.072940	1.090327
H	1.921043	-0.362020	1.692781
H	3.044833	-1.749921	1.766109
C	3.548377	-0.232633	0.261576
H	4.187158	0.271545	0.999912
H	4.198061	-0.926630	-0.290892
C	2.919878	0.751049	-0.694156
H	0.325469	-0.174533	-0.011194
C	2.022845	-3.211959	-0.236586
H	3.049249	-3.186176	-0.636697
H	2.071034	-3.861099	0.653006
H	1.366275	-3.676154	-0.978660
C	2.476401	1.991538	-0.392972
H	2.813178	0.417607	-1.731453
C	1.890479	2.887793	-1.459829

H	1.846691	2.397033	-2.440360
H	0.874541	3.220534	-1.191635
H	2.491483	3.804707	-1.566137
C	2.539025	2.598687	0.989833
H	3.117380	3.535432	0.973338
H	1.529186	2.875510	1.335708
H	2.987230	1.942450	1.744836
H	0.065164	-1.795752	2.181128
H	-0.622917	-3.053942	1.040728

A-B-TS G781.673773, T-67

C	-3.561085	-0.525602	0.963208
C	-3.238649	0.979076	1.264507
C	-2.018813	1.496103	0.527526
C	1.214414	-1.438543	-0.843884
C	-1.805203	-1.741541	-0.297988
C	-2.307902	-1.365424	0.917957
H	-4.135819	1.566187	1.016911
H	-3.079950	1.103305	2.344515
H	-4.247275	-0.896849	1.739832
H	-4.082445	-0.598769	0.000178
C	-1.634450	-1.640636	2.233724
H	-0.787407	-2.332227	2.168297
H	-1.265811	-0.698523	2.671157
H	-2.356932	-2.048052	2.955913
C	-0.579906	-2.463807	-0.535529
H	-2.369282	-1.439663	-1.183290
C	-1.882965	1.655440	-0.806985
H	-1.146161	1.725496	1.149443
C	-0.528484	1.998414	-1.402282
H	0.091805	2.527919	-0.666972
H	-0.642270	2.665238	-2.269757
C	-3.000157	1.450536	-1.801413
H	-2.781326	0.620678	-2.494391
H	-3.967944	1.246618	-1.331576
H	-3.114966	2.347200	-2.429455
C	0.650645	-0.152782	-0.735609
C	0.226164	0.724974	-1.862889
H	-0.410051	0.133181	-2.546704
H	1.112788	0.980269	-2.469165
C	2.040553	-1.858482	0.366432
H	1.489883	-1.620697	1.287872
H	2.237927	-2.939864	0.361324
C	3.381906	-1.062064	0.359710
H	3.861542	-1.186786	1.341209
H	4.062994	-1.505220	-0.379324
C	3.180829	0.398783	0.028711
H	0.341876	0.154566	0.265074
C	1.712304	-1.930521	-2.191023
H	2.657910	-1.431216	-2.452037
H	1.909919	-3.009583	-2.164429
H	1.001328	-1.727877	-3.001101

C	2.751752	1.368354	0.885976
H	3.467018	0.719695	-0.977599
C	2.694666	2.813108	0.455667
H	2.821070	2.935820	-0.626871
H	1.752108	3.290129	0.762814
H	3.496893	3.379591	0.955862
C	2.367885	1.104156	2.320205
H	2.934139	1.766581	2.992450
H	1.305355	1.348337	2.484397
H	2.535325	0.070421	2.640721
H	-0.170904	-3.050766	0.285812
H	-0.502355	-2.952386	-1.507242

B G781.696468

C	-3.336366	-0.793343	0.850715
C	-3.220884	0.725671	1.212977
C	-2.070507	1.406657	0.507189
C	1.201272	-1.512108	-0.752539
C	-1.419692	-1.819418	-0.340434
C	-1.997935	-1.503386	0.839038
H	-4.181614	1.206393	0.972231
H	-3.089175	0.833993	2.298819
H	-4.014583	-1.268566	1.577356
H	-3.804812	-0.892349	-0.137117
C	-1.355143	-1.726397	2.187641
H	-0.481862	-2.388746	2.152222
H	-1.031051	-0.768771	2.630527
H	-2.076521	-2.158731	2.896286
C	-0.076255	-2.471707	-0.562226
H	-1.987883	-1.587285	-1.245875
C	-1.922472	1.564632	-0.825201
H	-1.232884	1.720943	1.142488
C	-0.582793	1.994915	-1.393452
H	-0.053671	2.623995	-0.669123
H	-0.715267	2.596550	-2.305166
C	-2.991245	1.243358	-1.841033
H	-2.700636	0.397898	-2.487001
H	-3.955619	0.996891	-1.385747
H	-3.144980	2.102523	-2.512027
C	0.764988	-0.057815	-0.588077
C	0.294863	0.767088	-1.755708
H	-0.303297	0.077675	-2.378415
H	1.139893	1.046232	-2.403036
C	2.196129	-1.806258	0.394645
H	1.658673	-1.750161	1.353208
H	2.598918	-2.824475	0.310272
C	3.305960	-0.743432	0.351586
H	3.832371	-0.678799	1.313985
H	4.059400	-1.006435	-0.401762
C	2.709330	0.603416	-0.037180
H	0.203779	0.116609	0.333993
C	1.847536	-1.785296	-2.127007

H	2.706509	-1.132514	-2.332778
H	2.201817	-2.823361	-2.166409
H	1.127113	-1.652600	-2.944048
C	2.250772	1.541660	0.891545
H	3.019725	1.016406	-1.000949
C	2.164084	2.992670	0.529848
H	2.176911	3.167603	-0.550935
H	1.305749	3.496169	0.991821
H	3.065946	3.475131	0.948906
C	1.927573	1.192934	2.311156
H	2.570550	1.786289	2.981349
H	0.897032	1.497909	2.548055
H	2.050636	0.134170	2.550881
H	0.157866	-3.153890	0.265799
H	-0.134328	-3.089747	-1.468716

**B**	G781.696458		
C	-3.335954	-0.793476	0.851204
C	-3.220695	0.725633	1.212991
C	-2.070592	1.406636	0.506727
C	1.201505	-1.512200	-0.752128
C	-1.419392	-1.819920	-0.339871
C	-1.997368	-1.503232	0.839553
H	-4.181581	1.206118	0.972371
H	-3.088702	0.834311	2.298762
H	-4.013948	-1.268643	1.578099
H	-3.804534	-0.892913	-0.136520
C	-1.354087	-1.725199	2.188102
H	-0.480992	-2.387796	2.152899
H	-1.029605	-0.767277	2.630063
H	-2.075306	-2.156742	2.897393
C	-0.075789	-2.471977	-0.561588
H	-1.987917	-1.588661	-1.245321
C	-1.922685	1.563944	-0.825749
H	-1.233039	1.721535	1.141823
C	-0.583176	1.994247	-1.394349
H	-0.054070	2.623774	-0.670402
H	-0.715872	2.595394	-2.306353
C	-2.991486	1.241887	-1.841310
H	-2.700882	0.395999	-2.486713
H	-3.955815	0.995692	-1.385779
H	-3.145322	2.100589	-2.512871
C	0.764906	-0.057966	-0.588090
C	0.294686	0.766397	-1.756073
H	-0.303380	0.076627	-2.378476
H	1.139658	1.045402	-2.403531
C	2.196525	-1.805851	0.395035
H	1.659143	-1.749809	1.353640
H	2.599631	-2.823947	0.310754
C	3.306019	-0.742662	0.351832
H	3.832286	-0.677642	1.314286
H	4.059659	-1.005579	-0.401345

C	2.709015	0.603904	-0.037291
H	0.203558	0.116705	0.333842
C	1.847793	-1.785600	-2.126562
H	2.706585	-1.132647	-2.332532
H	2.202355	-2.823581	-2.165662
H	1.127318	-1.653359	-2.943629
C	2.250151	1.542253	0.891175
H	3.019364	1.016735	-1.001140
C	2.162992	2.993133	0.529092
H	2.175912	3.167783	-0.551736
H	1.304419	3.496454	0.990820
H	3.064637	3.476010	0.948141
C	1.926930	1.193795	2.310847
H	2.569035	1.788103	2.981019
H	0.895981	1.497798	2.547252
H	2.050959	0.135254	2.551046
H	0.158469	-3.154020	0.266517
H	-0.133790	-3.090134	-1.468000

B-C-TS G781.698413, T-267

C	-3.341268	-0.761663	0.836738
C	-3.188449	0.748271	1.222391
C	-2.028053	1.416400	0.520401
C	1.188374	-1.531955	-0.756512
C	-1.445243	-1.789463	-0.382071
C	-2.020323	-1.503836	0.806421
H	-4.139879	1.254356	0.996848
H	-3.046612	0.835666	2.308923
H	-4.028373	-1.231862	1.558161
H	-3.815782	-0.832552	-0.150593
C	-1.389573	-1.792069	2.148286
H	-0.539920	-2.483156	2.091365
H	-1.034098	-0.861265	2.623401
H	-2.127088	-2.222999	2.840988
C	-0.117076	-2.460915	-0.632979
H	-2.006516	-1.509612	-1.278153
C	-1.881773	1.593946	-0.809880
H	-1.181133	1.702072	1.156800
C	-0.536021	2.008187	-1.376391
H	0.002254	2.621741	-0.644752
H	-0.661143	2.623163	-2.280215
C	-2.961415	1.314689	-1.826566
H	-2.687869	0.481101	-2.494858
H	-3.927250	1.073137	-1.371725
H	-3.104660	2.192543	-2.475436
C	0.794389	-0.060017	-0.584735
C	0.327205	0.773362	-1.752419
H	-0.281896	0.096147	-2.376774
H	1.172743	1.052963	-2.398774
C	2.134115	-1.853424	0.422670
H	1.561588	-1.812366	1.361425
H	2.540870	-2.870003	0.338210

C	3.241457	-0.787729	0.436204
H	3.710784	-0.704912	1.425955
H	4.040603	-1.051948	-0.268331
C	2.658296	0.552633	-0.014575
H	0.200760	0.099466	0.320393
C	1.880862	-1.804108	-2.108294
H	2.775502	-1.186045	-2.263657
H	2.192203	-2.855586	-2.156894
H	1.202051	-1.622521	-2.951159
C	2.236976	1.537749	0.892294
H	3.022445	0.939079	-0.971000
C	2.239878	2.981747	0.501897
H	2.265461	3.132882	-0.582264
H	1.418778	3.550824	0.954336
H	3.174629	3.409731	0.910070
C	1.876754	1.226908	2.309883
H	2.631366	1.687290	2.970527
H	0.922671	1.702729	2.578215
H	1.827536	0.158300	2.532568
H	0.093942	-3.197720	0.154142
H	-0.188645	-3.022915	-1.574310

**C**	G781.710044		
C	3.455089	0.454691	0.646501
C	3.109218	-0.975582	1.177826
C	1.826775	-1.513063	0.584844
C	-1.040611	1.697934	-0.717754
C	1.616925	1.595661	-0.556553
C	2.255635	1.384934	0.616822
H	3.962242	-1.637197	0.961487
H	3.012809	-0.948235	2.272608
H	4.253656	0.874790	1.278191
H	3.862512	0.364077	-0.369218
C	1.814831	1.951184	1.944558
H	1.054904	2.735836	1.850058
H	1.404436	1.159601	2.595136
H	2.671235	2.376071	2.488938
C	0.379408	2.410382	-0.831176
H	2.057497	1.115210	-1.435008
C	1.596465	-1.819802	-0.711489
H	0.973204	-1.563964	1.268389
C	0.180435	-2.050090	-1.207000
H	-0.425984	-2.489340	-0.394035
H	0.170672	-2.772877	-2.036786
C	2.668167	-1.859307	-1.772700
H	2.500666	-1.089714	-2.544303
H	3.676234	-1.717459	-1.370043
H	2.646882	-2.827379	-2.297178
C	-0.924634	0.157644	-0.506466
C	-0.493225	-0.730257	-1.671975
H	0.223878	-0.170514	-2.290561
H	-1.349493	-0.943920	-2.330610

C	-1.768339	2.121061	0.576419
H	-1.060181	2.102981	1.417394
H	-2.176218	3.139490	0.514337
C	-2.872773	1.074410	0.804865
H	-3.065678	0.893482	1.869896
H	-3.830857	1.374228	0.361036
C	-2.409753	-0.221650	0.040172
H	-0.216607	0.010548	0.322400
C	-1.879241	2.055064	-1.961765
H	-2.902390	1.655633	-1.920005
H	-1.961489	3.145986	-2.055768
H	-1.409798	1.677713	-2.879838
C	-2.344352	-1.428577	0.843134
H	-3.033072	-0.399275	-0.846671
C	-2.979253	-2.678923	0.356090
H	-2.948509	-2.760332	-0.737522
H	-2.595642	-3.587005	0.833769
H	-4.053678	-2.596696	0.619622
C	-1.751325	-1.429666	2.203837
H	-2.567817	-1.604422	2.929546
H	-1.074091	-2.286646	2.330788
H	-1.242875	-0.498182	2.467359
H	0.341640	3.287677	-0.168405
H	0.465263	2.803553	-1.854145

**C**	G781.710040		
C	3.455222	0.454681	0.646334
C	3.109385	-0.975575	1.177749
C	1.826880	-1.513051	0.584904
C	-1.040627	1.697913	-0.717707
C	1.616981	1.595615	-0.556632
C	2.255752	1.384896	0.616709
H	3.962384	-1.637201	0.961335
H	3.013103	-0.948185	2.272540
H	4.253840	0.874811	1.277941
H	3.862567	0.363989	-0.369411
C	1.815085	1.951200	1.944468
H	1.055190	2.735887	1.850024
H	1.404724	1.159660	2.595116
H	2.671569	2.376067	2.488735
C	0.379422	2.410295	-0.831172
H	2.057532	1.115217	-1.435122
C	1.596487	-1.819839	-0.711399
H	0.973370	-1.563915	1.268531
C	0.180416	-2.050138	-1.206793
H	-0.425923	-2.489410	-0.393792
H	0.170594	-2.772929	-2.036576
C	2.668111	-1.859378	-1.772682
H	2.646419	-2.827244	-2.297521
H	2.500889	-1.089436	-2.544002
H	3.676253	-1.718054	-1.370022
C	-0.924674	0.157651	-0.506326

C	-0.493300	-0.730334	-1.671780
H	0.223750	-0.170592	-2.290436
H	-1.349591	-0.944045	-2.330374
C	-1.768416	2.121208	0.576369
H	-1.060327	2.103276	1.417400
H	-2.176316	3.139615	0.514098
C	-2.872848	1.074563	0.804954
H	-3.065609	0.893672	1.870017
H	-3.830992	1.374372	0.361247
C	-2.409957	-0.221531	0.040301
H	-0.216712	0.010539	0.322591
C	-1.879198	2.054989	-1.961781
H	-2.902392	1.655680	-1.919975
H	-1.961329	3.145910	-2.055887
H	-1.409792	1.677503	-2.879818
C	-2.344559	-1.428488	0.843154
H	-3.033216	-0.399057	-0.846604
C	-2.979477	-2.678779	0.355943
H	-2.947861	-2.760396	-0.737647
H	-2.596457	-3.586879	0.834047
H	-4.054105	-2.596162	0.618468
C	-1.751338	-1.429840	2.203764
H	-1.073869	-2.286706	2.330286
H	-1.243023	-0.498343	2.467509
H	-2.567648	-1.605071	2.929560
H	0.341683	3.287582	-0.168383
H	0.465206	2.803492	-1.854141

C-D-TS G781.700998, T-658

C	-3.373630	-0.102304	-1.163990
C	-3.480771	-1.254264	-0.105854
C	-2.291108	-1.350672	0.826230
C	0.989943	1.689916	-0.429685
C	-1.291592	1.214884	-1.473990
C	-1.970220	0.070580	-1.711611
H	-4.413423	-1.102107	0.460406
H	-3.596462	-2.215138	-0.626202
H	-4.080572	-0.329527	-1.978982
H	-3.704275	0.841980	-0.710993
C	-1.421285	-1.114403	-2.473031
H	-0.403688	-0.961515	-2.851098
H	-1.424281	-2.025082	-1.853893
H	-2.064911	-1.333792	-3.338856
C	0.160042	1.503561	-1.747202
H	-1.835854	2.005823	-0.948576
C	-1.893715	-0.421891	1.721090
H	-1.678718	-2.254839	0.730552
C	-0.564855	-0.546931	2.441615
H	-0.147242	-1.558799	2.308500
H	-0.700195	-0.404400	3.526315
C	-2.683336	0.838211	1.999215
H	-2.589992	1.138754	3.052783

H	-2.327244	1.684464	1.391305
H	-3.749599	0.712126	1.777640
C	0.829340	0.371388	0.450846
C	0.445279	0.528311	1.957222
H	-0.000036	1.514834	2.121469
H	1.345471	0.506882	2.594739
C	2.520856	1.762124	-0.771888
H	2.709318	1.405623	-1.795532
H	2.917036	2.782803	-0.703581
C	3.230703	0.826501	0.234770
H	4.231540	0.517065	-0.080215
H	3.308119	1.323575	1.213475
C	2.197004	-0.277723	0.305186
H	0.041550	-0.238654	-0.009659
C	0.569899	2.983993	0.288715
H	1.188045	3.183102	1.174950
H	0.694548	3.831667	-0.398813
H	-0.480148	2.972297	0.604300
C	2.451509	-1.648453	0.015218
H	2.396681	-1.135536	1.272448
C	1.336717	-2.638863	-0.191986
H	0.355887	-2.267302	0.108695
H	1.302299	-2.849149	-1.272875
H	1.549709	-3.588823	0.312719
C	3.850325	-2.161894	-0.236939
H	3.957689	-3.194782	0.112737
H	4.004477	-2.166683	-1.327558
H	4.633549	-1.544467	0.210726
H	0.617854	0.693466	-2.335566
H	0.276234	2.423705	-2.344950

**D**	G781.721520		
C	-3.309187	0.044515	-1.119911
C	-3.267885	-1.376063	-0.447881
C	-2.062010	-1.628593	0.424663
C	0.885434	1.892819	0.146258
C	-1.351843	1.555341	-0.996917
C	-1.956989	0.508001	-1.612251
H	-4.197220	-1.485057	0.133252
H	-3.302724	-2.146499	-1.229680
H	-4.026008	-0.010106	-1.954809
H	-3.705506	0.773934	-0.401698
C	-1.364314	-0.277725	-2.755305
H	-0.339627	0.015105	-3.008841
H	-1.371131	-1.358020	-2.544452
H	-1.980934	-0.142679	-3.657655
C	0.047997	2.064484	-1.173052
H	-1.932501	2.058830	-0.217928
C	-1.741773	-0.981674	1.572203
H	-1.365041	-2.399654	0.078435
C	-0.408267	-1.238084	2.237742
H	0.032276	-2.165535	1.843900

H	-0.536977	-1.388756	3.322452
C	-2.622500	0.073382	2.199324
H	-2.558301	0.038029	3.295987
H	-2.317996	1.087277	1.896122
H	-3.675074	-0.043964	1.916615
C	0.813594	0.362991	0.553971
C	0.573807	-0.053100	2.064037
H	0.168936	0.834254	2.563706
H	1.527199	-0.279021	2.562515
C	2.387409	2.116874	-0.177859
H	2.537193	2.908960	-0.922039
H	2.936024	2.411592	0.729482
C	2.899644	0.747197	-0.653474
H	2.804614	0.610550	-1.751265
H	3.960972	0.543099	-0.447839
C	2.006346	-0.252338	-0.013678
H	-0.100582	-0.083884	0.050491
C	0.406156	2.884110	1.216545
H	1.017038	2.825336	2.127143
H	0.490844	3.909606	0.832126
H	-0.642135	2.728539	1.500839
C	2.288646	-1.709516	-0.022665
H	1.790094	-2.168109	0.844744
C	1.573150	-2.287668	-1.295407
H	0.498721	-2.081256	-1.285612
H	2.009114	-1.871313	-2.211993
H	1.726979	-3.373735	-1.299796
C	3.782401	-2.085601	-0.023294
H	3.879321	-3.175993	0.043379
H	4.282826	-1.764109	-0.945912
H	4.309336	-1.646709	0.833769
H	0.552733	1.547079	-2.002717
H	0.048644	3.136090	-1.431016

**D**	G781.720938		
C	-3.483266	0.116468	-0.800433
C	-2.881323	-1.213791	-1.379288
C	-1.619270	-1.681680	-0.694150
C	0.716176	1.718347	0.785680
C	-1.832572	1.301556	0.594431
C	-2.471256	1.227744	-0.603385
H	-3.671388	-1.978392	-1.316320
H	-2.672679	-1.076799	-2.449705
H	-4.281854	0.442653	-1.482779
H	-3.956044	-0.108002	0.163946
C	-2.235835	2.150705	-1.770925
H	-1.575108	2.992370	-1.534872
H	-1.807969	1.603780	-2.627672
H	-3.192539	2.564105	-2.123704
C	-0.744755	2.225114	1.062894
H	-2.159621	0.579587	1.345958
C	-1.469815	-2.024455	0.608509

H	-0.719215	-1.689593	-1.318511
C	-0.077119	-2.206035	1.188047
H	0.582416	-2.664686	0.438492
H	-0.103579	-2.895170	2.044607
C	-2.612496	-2.165772	1.583304
H	-2.543198	-1.437970	2.408421
H	-3.597125	-2.057848	1.117649
H	-2.572371	-3.160340	2.054070
C	0.797691	0.150843	0.543776
C	0.532636	-0.858336	1.678837
H	-0.159097	-0.386058	2.390331
H	1.462377	-1.050600	2.234896
C	1.235753	2.235252	-0.580912
H	0.433502	2.187177	-1.329077
H	1.589002	3.272511	-0.531240
C	2.344232	1.243769	-0.961687
H	2.559279	1.120508	-2.035338
H	3.313343	1.547331	-0.519340
C	1.971571	-0.030846	-0.291360
H	-0.086083	0.008173	-0.181243
C	1.641912	2.149759	1.940166
H	2.697587	1.901721	1.758161
H	1.576966	3.236372	2.086065
H	1.344539	1.670178	2.882284
C	2.773791	-1.271041	-0.466513
H	2.510617	-1.986344	0.323637
C	2.400126	-1.918346	-1.840037
H	1.334351	-2.164236	-1.908313
H	2.664517	-1.255226	-2.672804
H	2.974209	-2.847419	-1.942319
C	4.298315	-1.000796	-0.406664
H	4.827557	-1.960013	-0.458125
H	4.634241	-0.384649	-1.250053
H	4.583108	-0.504862	0.530135
H	-0.831915	3.226124	0.613705
H	-0.864800	2.364859	2.147315

D-E-TS G781.712048, T-693

C	-3.468896	-0.075918	-0.830985
C	-2.718951	-1.316838	-1.420740
C	-1.453693	-1.701102	-0.683563
C	0.610106	1.733669	0.758749
C	-1.897367	1.143790	0.619189
C	-2.562893	1.114856	-0.556601
H	-3.427332	-2.160562	-1.439081
H	-2.462384	-1.115970	-2.470923
H	-4.267307	0.199773	-1.535291
H	-3.961147	-0.372187	0.104378
C	-2.474782	2.160235	-1.641804
H	-1.864239	3.027578	-1.364502
H	-2.069946	1.738753	-2.577004
H	-3.482320	2.528568	-1.888119

C	-0.857743	2.106258	1.129398
H	-2.133888	0.340774	1.317966
C	-1.331478	-2.041358	0.619343
H	-0.531334	-1.659160	-1.279258
C	0.046382	-2.149255	1.247507
H	0.750277	-2.584065	0.527275
H	0.024194	-2.829209	2.110531
C	-2.495743	-2.293741	1.546665
H	-2.530989	-1.571876	2.379407
H	-3.464228	-2.269430	1.038044
H	-2.386409	-3.286939	2.009489
C	0.981414	0.239918	0.729398
C	0.603440	-0.780943	1.779177
H	-0.126712	-0.329060	2.461211
H	1.513582	-0.980685	2.368586
C	0.969281	2.182814	-0.683583
H	0.107097	1.996553	-1.337271
H	1.206946	3.251808	-0.732238
C	2.132012	1.279293	-1.115283
H	2.199884	1.106766	-2.196064
H	3.104692	1.684427	-0.794806
C	1.906617	0.003460	-0.323273
H	0.598192	-0.244217	-0.483702
C	1.584860	2.364732	1.795094
H	2.640338	2.205619	1.535731
H	1.405052	3.447014	1.832453
H	1.412833	1.960735	2.801263
C	2.788302	-1.214822	-0.514321
H	2.533930	-1.961367	0.248702
C	2.630600	-1.847619	-1.911069
H	1.604541	-2.194312	-2.092960
H	2.900873	-1.138904	-2.704724
H	3.297156	-2.714201	-1.997609
C	4.255864	-0.787391	-0.252857
H	4.887726	-1.684131	-0.253936
H	4.625576	-0.115306	-1.037185
H	4.372160	-0.291448	0.720303
H	-1.018216	3.131397	0.761186
H	-0.942579	2.163027	2.225496

**E**	G781.718507		
C	3.731475	0.111792	0.544231
C	3.166554	-1.077956	1.391874
C	1.808355	-1.543210	0.923652
C	-0.629530	1.535065	-0.738286
C	1.916874	1.068148	-0.818430
C	2.702554	1.199260	0.272759
H	3.901382	-1.897260	1.357232
H	3.093883	-0.774626	2.445668
H	4.604453	0.524907	1.070622
H	4.093454	-0.283546	-0.414138
C	2.619935	2.318714	1.280324

H	1.906570	3.104131	1.004769
H	2.340717	1.937967	2.276751
H	3.606986	2.789994	1.401863
C	0.770853	1.928974	-1.293420
H	2.144888	0.226400	-1.474854
C	1.500199	-2.095893	-0.270947
H	0.974788	-1.324313	1.601894
C	0.049678	-2.279497	-0.649997
H	-0.570841	-2.478321	0.232212
H	-0.072680	-3.128720	-1.335376
C	2.515733	-2.516534	-1.306587
H	2.430962	-1.925833	-2.234102
H	3.546975	-2.427866	-0.950456
H	2.349963	-3.566022	-1.594724
C	-0.985312	0.099891	-0.575802
C	-0.568540	-1.022707	-1.428433
H	0.104883	-0.702130	-2.230436
H	-1.497732	-1.404230	-1.890300
C	-0.959748	2.145026	0.654724
H	-0.089686	2.003146	1.310475
H	-1.158964	3.221499	0.582865
C	-2.149809	1.323343	1.169647
H	-2.222942	1.312722	2.262300
H	-3.103549	1.709125	0.785048
C	-1.869606	-0.088579	0.597990
H	-1.107959	-0.552447	1.280409
C	-1.733952	2.001291	-1.784116
H	-2.760304	1.819604	-1.450923
H	-1.594582	3.084955	-1.897135
H	-1.581067	1.521471	-2.757411
C	-3.053432	-1.099590	0.505831
H	-2.647073	-2.053847	0.134929
C	-3.645071	-1.349790	1.902329
H	-2.884070	-1.713862	2.606506
H	-4.085203	-0.433442	2.318543
H	-4.442402	-2.101599	1.846227
C	-4.132364	-0.628065	-0.486059
H	-4.923854	-1.384319	-0.559028
H	-4.606314	0.305797	-0.154495
H	-3.732884	-0.467249	-1.497536
H	0.909679	2.989347	-1.032241
H	0.743442	1.890466	-2.392860

E G781.718502

C	3.731685	0.111889	0.543993
C	3.167050	-1.077885	1.391739
C	1.808780	-1.543137	0.923740
C	-0.629902	1.535150	-0.738457
C	1.916582	1.067960	-0.818187
C	2.702672	1.199290	0.272691
H	3.901929	-1.897132	1.356877
H	3.094609	-0.774593	2.445556

H	4.604743	0.525134	1.070157
H	4.093486	-0.283398	-0.414470
C	2.620373	2.318915	1.280104
H	1.907235	3.104496	1.004478
H	2.340927	1.938260	2.276508
H	3.607517	2.789968	1.401792
C	0.770675	1.928995	-1.293203
H	2.144327	0.226013	-1.474455
C	1.500443	-2.095806	-0.270863
H	0.975297	-1.324373	1.602126
C	0.049878	-2.279675	-0.649343
H	-0.570324	-2.478033	0.233172
H	-0.072843	-3.129038	-1.334463
C	2.515786	-2.516353	-1.306689
H	2.430706	-1.925734	-2.234226
H	3.547089	-2.427441	-0.950785
H	2.350151	-3.565899	-1.594707
C	-0.985290	0.099731	-0.575577
C	-0.568536	-1.022875	-1.427993
H	0.104719	-0.702529	-2.230205
H	-1.497832	-1.404554	-1.889557
C	-0.960366	2.145333	0.654229
H	-0.090339	2.004054	1.310194
H	-1.159878	3.221750	0.582112
C	-2.150193	1.323489	1.169536
H	-2.223152	1.313173	2.262196
H	-3.104125	1.708821	0.784972
C	-1.869623	-0.088577	0.598306
H	-1.108340	-0.552155	1.281131
C	-1.734103	2.000784	-1.784599
H	-2.760490	1.818612	-1.451790
H	-1.595188	3.084496	-1.897719
H	-1.580435	1.520838	-2.757681
C	-3.053441	-1.099750	0.506154
H	-2.646841	-2.054243	0.136115
C	-3.645734	-1.348958	1.902488
H	-2.884905	-1.712011	2.607375
H	-4.086634	-0.432534	2.317745
H	-4.442615	-2.101267	1.846630
C	-4.131865	-0.628874	-0.486618
H	-4.923713	-1.384797	-0.558985
H	-4.605448	0.305611	-0.156289
H	-3.732018	-0.469345	-1.498161
H	0.909712	2.989275	-1.031730
H	0.743450	1.890696	-2.392631

E-F-TS G781.708571, T-345

C	-3.783131	-0.050060	0.567139
C	-3.079471	0.997829	1.502531
C	-1.697206	1.411974	1.046619
C	0.338880	-1.369871	-0.696795
C	-2.089550	-0.947749	-0.957758

C	-2.842015	-1.159302	0.142770
H	-3.746846	1.869901	1.579737
H	-3.005360	0.579345	2.516350
H	-4.659901	-0.451673	1.095510
H	-4.153161	0.470404	-0.325923
C	-2.750399	-2.363573	1.044652
H	-2.093464	-3.153204	0.660455
H	-2.394744	-2.079706	2.049307
H	-3.748518	-2.802488	1.190998
C	-0.919191	-1.759308	-1.466640
H	-2.273999	-0.030945	-1.517708
C	-1.375940	2.085957	-0.076983
H	-0.870726	1.065244	1.677591
C	0.080184	2.274008	-0.471156
H	0.711851	2.367168	0.421458
H	0.195376	3.214521	-1.029568
C	-2.387907	2.677386	-1.030235
H	-2.326207	2.222176	-2.033346
H	-3.420333	2.573869	-0.680988
H	-2.189800	3.751247	-1.172197
C	1.017067	-0.119394	-0.668703
C	0.656222	1.152075	-1.405341
H	-0.054079	0.942238	-2.216289
H	1.574857	1.541472	-1.869459
C	0.778003	-2.199555	0.498204
H	-0.030168	-2.086186	1.238505
H	0.857924	-3.270409	0.268689
C	2.074055	-1.537312	0.997981
H	2.212289	-1.651851	2.077710
H	2.952858	-1.983618	0.510432
C	1.902714	-0.059258	0.581977
H	1.203738	0.391420	1.315184
C	1.747681	-1.304697	-1.918675
H	2.773634	-1.009135	-1.700830
H	1.722074	-2.400311	-1.915673
H	1.377717	-0.912746	-2.864480
C	3.163421	0.843559	0.590850
H	2.826736	1.865605	0.349109
C	3.749974	0.878294	2.015598
H	2.988523	1.144298	2.762342
H	4.174336	-0.096098	2.295897
H	4.557497	1.618367	2.079641
C	4.254449	0.473496	-0.430901
H	5.137575	1.105239	-0.271646
H	4.590085	-0.568670	-0.323282
H	3.939171	0.639150	-1.470967
H	-1.063234	-2.842177	-1.346480
H	-0.792737	-1.569435	-2.540894

**F**	G781.718626		
C	3.732971	-0.037681	-0.535702
C	2.989924	0.960772	-1.513435

C	1.613883	1.376917	-1.058598
C	-0.101899	-1.310821	0.593774
C	2.017873	-0.930414	0.970614
C	2.829244	-1.161712	-0.105375
H	3.656986	1.828885	-1.623393
H	2.920918	0.492024	-2.504701
H	4.626612	-0.419251	-1.047362
H	4.064403	0.527823	0.343962
C	2.816311	-2.405457	-0.941665
H	2.222034	-3.222421	-0.518944
H	2.423007	-2.186616	-1.950202
H	3.844059	-2.765018	-1.094582
C	0.918062	-1.832708	1.523930
H	2.094004	0.032466	1.469678
C	1.312377	2.086343	0.051105
H	0.779213	0.993535	-1.653914
C	-0.127487	2.246957	0.502103
H	-0.799986	2.316213	-0.362328
H	-0.230316	3.201066	1.041219
C	2.346166	2.724710	0.949766
H	2.317279	2.308008	1.970978
H	3.371332	2.632021	0.576161
H	2.129434	3.798258	1.060079
C	-1.104888	-0.215697	0.879833
C	-0.635232	1.147528	1.478037
H	0.112599	0.962469	2.265211
H	-1.509446	1.557770	2.003647
C	-0.437593	-1.998679	-0.695465
H	0.258270	-1.626552	-1.467133
H	-0.288673	-3.085924	-0.642671
C	-1.866648	-1.523649	-1.043317
H	-2.056200	-1.554334	-2.122250
H	-2.605558	-2.176673	-0.559721
C	-1.900846	-0.096706	-0.468760
H	-1.254564	0.506442	-1.128071
C	-1.972811	-0.906846	2.007696
H	-2.872211	-0.298791	2.152106
H	-2.287832	-1.925171	1.750824
H	-1.418303	-0.939982	2.952080
C	-3.281442	0.618102	-0.429932
H	-3.253897	1.348404	0.398321
C	-3.496060	1.411811	-1.734453
H	-2.716655	2.172336	-1.884574
H	-3.483791	0.740201	-2.605952
H	-4.467255	1.923485	-1.727647
C	-4.485143	-0.316817	-0.198516
H	-5.401386	0.277734	-0.089818
H	-4.634129	-0.985602	-1.058171
H	-4.391794	-0.942934	0.697174
H	1.118076	-2.901885	1.391497
H	0.733196	-1.603710	2.578661

**F**	G781.708263		
C	3.655631	-1.139944	-0.711076
C	3.766355	0.400538	-0.872632
C	2.420832	1.028536	-1.125628
C	-0.401179	-0.801902	0.696530
C	1.805215	-0.922145	0.986104
C	2.508269	-1.698776	0.109751
H	4.235914	0.832032	0.021249
H	4.462026	0.580038	-1.708988
H	3.565478	-1.603750	-1.709738
H	4.607152	-1.530534	-0.310289
C	2.223760	-3.154584	-0.146666
H	1.522037	-3.607732	0.561488
H	1.829243	-3.307000	-1.165736
H	3.163853	-3.725466	-0.109634
C	0.528798	-1.325146	1.724941
H	2.088151	0.115511	1.123033
C	1.868364	2.038285	-0.421185
H	1.787464	0.508708	-1.854611
C	0.381690	2.309027	-0.514903
H	-0.041757	1.846494	-1.415967
H	0.203522	3.392043	-0.611115
C	2.628041	2.886386	0.577803
H	2.350164	2.666119	1.622387
H	3.713663	2.775076	0.495295
H	2.392457	3.949185	0.418013
C	-1.185023	0.484082	0.766348
C	-0.375191	1.844935	0.766851
H	0.310586	1.832645	1.625793
H	-1.144168	2.590854	1.013195
C	-0.822171	-1.603697	-0.481145
H	-0.037029	-2.235341	-0.908869
H	-1.570070	-2.314288	-0.068703
C	-1.501388	-0.610478	-1.436092
H	-0.737928	-0.145632	-2.075851
H	-2.221348	-1.102779	-2.099084
C	-2.155755	0.434590	-0.498191
H	-2.113509	1.425723	-0.971729
C	-1.899965	0.475033	2.164103
H	-2.629373	1.291780	2.189174
H	-2.429734	-0.460056	2.371865
H	-1.176995	0.650057	2.969112
C	-3.669385	0.169501	-0.238093
H	-3.982937	0.828784	0.587700
C	-4.483369	0.597678	-1.476352
H	-4.315459	1.654789	-1.723988
H	-4.220086	-0.001143	-2.360517
H	-5.557674	0.459191	-1.297745
C	-4.039642	-1.275049	0.148678
H	-5.112581	-1.339274	0.370152
H	-3.845129	-1.976287	-0.676402
H	-3.507728	-1.639485	1.037496

H	0.452733	-0.821278	2.693372
H	0.435740	-2.410205	1.854773

F-G-TS G781.675855, T-1044

C	-3.772447	1.476367	-0.271558
C	-3.920903	-0.038400	-0.660898
C	-2.619824	-0.651257	-1.133885
C	0.389150	0.217035	0.536417
C	-1.975020	0.846876	1.295907
C	-2.460441	1.781398	0.428066
H	-4.330969	-0.591553	0.193897
H	-4.682808	-0.092461	-1.454657
H	-3.816721	2.104189	-1.174845
H	-4.637212	1.781719	0.341030
C	-1.781095	3.080626	0.106247
H	-0.839075	3.220825	0.648261
H	-1.613113	3.208811	-0.972795
H	-2.454067	3.900656	0.406646
C	-0.653122	0.301447	1.503439
H	-2.698393	0.129738	1.698319
C	-1.923494	-1.664615	-0.568242
H	-2.174871	-0.162932	-2.009707
C	-0.558674	-2.084719	-1.106465
H	-0.277292	-1.461151	-1.964818
H	-0.661373	-3.108278	-1.507606
C	-2.445805	-2.498371	0.583559
H	-1.900381	-2.315129	1.523874
H	-3.509198	-2.335534	0.784736
H	-2.315081	-3.568815	0.364575
C	1.391418	-0.925353	0.502783
C	0.592993	-2.192677	-0.061235
H	0.197316	-2.727285	0.814069
H	1.372531	-2.842808	-0.483092
C	0.477454	1.031659	-0.727181
H	-0.493681	0.996642	-1.241811
H	0.655852	2.089196	-0.491741
C	1.651923	0.430987	-1.534808
H	1.270352	-0.181054	-2.360636
H	2.260268	1.220815	-1.989689
C	2.464904	-0.437681	-0.537940
H	2.857018	-1.326466	-1.056078
C	1.968089	-1.341735	1.868929
H	2.680311	-2.165970	1.736365
H	2.494469	-0.519551	2.367249
H	1.183192	-1.705965	2.545398
C	3.700597	0.300683	0.052793
H	4.129030	-0.355043	0.828351
C	4.788560	0.471309	-1.024959
H	5.072885	-0.494679	-1.464167
H	4.456510	1.127916	-1.842085
H	5.690516	0.923679	-0.591979
C	3.387773	1.659144	0.707257

H	4.297071	2.088555	1.147081
H	3.013463	2.386501	-0.027699
H	2.648273	1.587700	1.520468
H	-0.561969	-0.403251	2.335577
H	0.474924	1.014629	1.628143

**G**	G781.702814		
C	-3.681047	1.406820	-0.484297
C	-3.793442	-0.093982	-0.967647
C	-2.463898	-0.725569	-1.271725
C	0.477439	0.536251	0.833887
C	-2.061896	0.737465	1.290220
C	-2.502019	1.717520	0.376776
H	-4.347563	-0.667068	-0.214631
H	-4.430146	-0.060301	-1.865454
H	-3.685698	2.107049	-1.332775
H	-4.599276	1.639325	0.087535
C	-1.904341	3.079182	0.318912
H	-0.986781	3.174455	0.905423
H	-1.753709	3.425032	-0.712617
H	-2.649800	3.768592	0.758587
C	-0.776665	0.321822	1.583125
H	-2.846382	0.084404	1.692160
C	-1.873768	-1.736409	-0.592217
H	-1.886941	-0.249697	-2.072815
C	-0.449126	-2.159601	-0.906578
H	-0.068435	-1.608438	-1.775521
H	-0.478494	-3.219152	-1.211715
C	-2.572680	-2.554365	0.473572
H	-2.147182	-2.390060	1.475549
H	-3.648836	-2.363719	0.535921
H	-2.442891	-3.626099	0.262663
C	1.346322	-0.795540	0.656450
C	0.539042	-2.088079	0.293962
H	0.010314	-2.429047	1.198024
H	1.312360	-2.851060	0.121571
C	0.431498	1.241325	-0.534003
H	-0.572389	1.137712	-0.977443
H	0.618427	2.317343	-0.430261
C	1.498602	0.567374	-1.424154
H	1.013030	0.019563	-2.240678
H	2.142904	1.312625	-1.905707
C	2.312705	-0.401869	-0.523671
H	2.516794	-1.325096	-1.090240
C	2.067789	-1.128114	1.979145
H	2.713956	-2.008232	1.862614
H	2.688823	-0.301333	2.341311
H	1.344655	-1.371042	2.772112
C	3.710771	0.170944	-0.139368
H	4.151419	-0.514839	0.602610
C	4.641701	0.147879	-1.367618
H	4.724976	-0.863225	-1.789763

H	4.284321	0.816049	-2.164764
H	5.650887	0.482554	-1.092257
C	3.694952	1.582620	0.477198
H	4.718833	1.904578	0.707821
H	3.273794	2.326446	-0.214816
H	3.126700	1.636550	1.416024
H	-0.695919	-0.408264	2.395317
H	1.096625	1.150354	1.524396

NH_3 G56.563161

N	-0.000000	-0.000000	0.123483
H	0.000000	0.935817	-0.288128
H	-0.810441	-0.467909	-0.288128
H	0.810441	-0.467909	-0.288128

G* G838.275259

G*–NH_3 G781.712098

C	-4.077298	0.774340	-0.896976
C	-4.055231	-0.790990	-0.725330
C	-2.668498	-1.365060	-0.841828
C	0.263752	0.872672	0.462206
C	-2.306649	0.984472	0.832027
C	-2.885003	1.489972	-0.334543
H	-4.517760	-1.054403	0.234037
H	-4.718919	-1.188189	-1.509753
H	-4.172302	1.056332	-1.956935
H	-4.992511	1.163526	-0.413648
C	-2.426184	2.730269	-1.021888
H	-1.586138	3.215593	-0.519374
H	-2.186650	2.540692	-2.078990
H	-3.274608	3.437011	-1.035534
C	-0.974125	0.835140	1.217419
H	-2.994212	0.428474	1.481216
C	-1.962271	-1.987764	0.127578
H	-2.150507	-1.149309	-1.784177
C	-0.503274	-2.350271	-0.089940
H	-0.200882	-2.077744	-1.111012
H	-0.419204	-3.448860	-0.034016
C	-2.544986	-2.402513	1.461923
H	-2.158118	-1.791219	2.293193
H	-3.638192	-2.351115	1.494399
H	-2.258464	-3.439444	1.691159
C	1.219013	-0.410427	0.730394
C	0.504330	-1.782226	0.953900
H	0.039236	-1.791001	1.952130
H	1.333640	-2.503803	1.026674
C	0.319440	1.145883	-1.040513
H	-0.383508	0.487997	-1.579410
H	0.074073	2.181992	-1.295180
C	1.778744	0.813358	-1.383359
H	1.952139	0.705413	-2.461465
H	2.419159	1.633649	-1.027183

C	2.062014	-0.483736	-0.607872
H	1.588885	-1.284720	-1.199042
C	2.033709	-0.142239	2.010358
H	2.727734	-0.962836	2.230435
H	2.609331	0.785553	1.943699
H	1.362129	-0.051314	2.877872
C	3.558163	-0.898371	-0.496783
H	3.633470	-1.641157	0.316516
C	4.000759	-1.605901	-1.794019
H	3.391585	-2.498234	-1.995731
H	3.913752	-0.933979	-2.660805
H	5.050679	-1.921926	-1.728401
C	4.534815	0.250616	-0.182915
H	5.543023	-0.149911	-0.012925
H	4.604371	0.952148	-1.026634
H	4.256270	0.823115	0.710414
H	-0.847729	0.461075	2.240047
N	1.927683	3.364100	1.343353
H	1.571666	4.222115	0.920157
H	2.897232	3.275427	1.036661
H	1.960692	3.530003	2.350122
H	0.849542	1.726995	0.948987

G-H-TS* G838.264979, T-1296

G-H-TS*–NH_3 G781.701818

C	-4.122397	0.806141	-0.800189
C	-4.054827	-0.763027	-0.722421
C	-2.656524	-1.297838	-0.923292
C	0.210305	0.702261	0.462433
C	-2.315145	0.942509	0.887285
C	-2.912551	1.503679	-0.218752
H	-4.467387	-1.092636	0.239658
H	-4.738121	-1.145051	-1.498099
H	-4.215395	1.129855	-1.848856
H	-5.049196	1.148593	-0.307555
C	-2.450893	2.755802	-0.899430
H	-1.611935	3.235804	-0.385104
H	-2.191464	2.581600	-1.954555
H	-3.289157	3.472494	-0.910399
C	-0.958422	0.703747	1.241748
H	-2.969726	0.297855	1.486644
C	-1.912494	-1.989122	-0.031550
H	-2.175299	-1.000013	-1.862649
C	-0.452016	-2.317966	-0.308427
H	-0.158281	-1.933399	-1.296338
H	-0.364090	-3.415863	-0.376984
C	-2.456807	-2.520402	1.278820
H	-2.105783	-1.936670	2.145486
H	-3.550877	-2.535151	1.314787
H	-2.108805	-3.550654	1.444574
C	1.251061	-0.461095	0.701521
C	0.567837	-1.872619	0.784379

H	0.105727	-1.985584	1.777406
H	1.404326	-2.589662	0.773505
C	0.279736	1.094088	-1.006601
H	-0.408003	0.454871	-1.587342
H	-0.018362	2.130451	-1.200799
C	1.749038	0.846085	-1.383533
H	1.897141	0.737932	-2.465200
H	2.354897	1.712837	-1.073281
C	2.153680	-0.420493	-0.604085
H	1.805155	-1.267771	-1.216388
C	2.004223	-0.285538	2.037215
H	2.667163	-1.135876	2.238836
H	2.623753	0.617120	2.070816
H	1.290830	-0.234032	2.873014
C	3.685029	-0.649399	-0.448272
H	3.824557	-1.405752	0.343082
C	4.263191	-1.244986	-1.748364
H	3.777745	-2.196631	-2.005455
H	4.125251	-0.556206	-2.595046
H	5.341032	-1.432310	-1.650074
C	4.504207	0.596932	-0.058994
H	5.542918	0.313717	0.155997
H	4.535041	1.327444	-0.880266
H	4.122582	1.106440	0.837184
H	-0.842818	0.240995	2.229096
N	1.521639	2.945407	1.438572
H	1.081369	3.798595	1.089945
H	2.469424	2.888292	1.061062
H	1.580657	2.999241	2.456948
H	0.806269	1.828732	1.069395

H* G838.275904
H*–NH_3 G781.712743

C	-4.058738	0.354918	-1.023717
C	-3.843724	-1.169669	-0.726917
C	-2.390057	-1.595713	-0.808317
C	0.141232	0.577023	0.293989
C	-2.342786	0.840971	0.677249
C	-2.957703	1.248341	-0.460401
H	-4.263550	-1.407034	0.259532
H	-4.449093	-1.732332	-1.456945
H	-4.063304	0.512037	-2.115252
H	-5.064718	0.655800	-0.684365
C	-2.588970	2.454652	-1.282880
H	-1.831285	3.085987	-0.797219
H	-2.231779	2.192458	-2.290762
H	-3.483688	3.081286	-1.430019
C	-0.940564	0.789688	1.114893
H	-2.897281	0.102692	1.265499
C	-1.629194	-2.135811	0.171603
H	-1.899002	-1.376779	-1.764654
C	-0.143762	-2.419111	-0.027844

H	0.150560	-2.167190	-1.057715
H	0.007476	-3.508244	0.067133
C	-2.172032	-2.531585	1.529189
H	-1.856305	-1.831817	2.321079
H	-3.265109	-2.585587	1.556537
H	-1.785663	-3.519065	1.822963
C	1.331663	-0.296971	0.708807
C	0.833692	-1.759023	0.996079
H	0.384492	-1.792321	2.000098
H	1.742850	-2.378658	1.055971
C	0.303957	0.997508	-1.144455
H	-0.213312	0.260844	-1.785260
H	-0.140099	1.970403	-1.380332
C	1.829736	0.949532	-1.364010
H	2.107758	0.906749	-2.424411
H	2.294260	1.858625	-0.943860
C	2.256298	-0.297620	-0.573914
H	1.908920	-1.155559	-1.175057
C	2.010046	0.245245	1.985745
H	2.869155	-0.370383	2.279324
H	2.375343	1.272867	1.844811
H	1.304724	0.241164	2.828988
C	3.780925	-0.524108	-0.382279
H	3.903209	-1.220170	0.466748
C	4.368575	-1.222419	-1.625530
H	3.881238	-2.189502	-1.812488
H	4.240343	-0.600665	-2.524404
H	5.444798	-1.404122	-1.503301
C	4.597602	0.745954	-0.077217
H	5.643044	0.480036	0.128253
H	4.602544	1.428397	-0.939547
H	4.226680	1.303756	0.791717
H	-0.829942	0.393715	2.132257
N	0.080666	3.460699	1.582218
H	-0.784788	3.992158	1.717703
H	0.628771	3.885037	0.828085
H	0.629794	3.472196	2.446996
H	-0.168889	2.407510	1.315940

H* G838.281569

H*–NH_3 G781.718408

C	-3.669732	0.563275	-1.159751
C	-3.668306	-0.986011	-0.911523
C	-2.284499	-1.592719	-1.026994
C	0.456082	0.225276	0.311880
C	-1.952806	0.785561	0.625524
C	-2.462850	1.269869	-0.552066
H	-4.108387	-1.192831	0.073180
H	-4.352391	-1.433448	-1.650853
H	-3.642097	0.758633	-2.243804
H	-4.626954	0.990437	-0.813743
C	-1.850404	2.393648	-1.350648

H	-0.985530	2.847118	-0.847829
H	-1.521596	2.066068	-2.346785
H	-2.607478	3.176347	-1.531460
C	-0.574315	0.622869	1.110992
H	-2.622241	0.115103	1.178038
C	-1.555354	-2.188375	-0.054815
H	-1.807733	-1.462338	-2.005626
C	-0.118280	-2.638240	-0.299151
H	0.184135	-2.391674	-1.324574
H	-0.108264	-3.741681	-0.245962
C	-2.101589	-2.516386	1.318787
H	-1.652930	-1.884096	2.101027
H	-3.189952	-2.413818	1.386470
H	-1.854313	-3.554874	1.586337
C	1.562304	-0.712663	0.776107
C	0.949692	-2.184466	0.746523
H	0.525199	-2.368724	1.744407
H	1.808324	-2.867281	0.658576
C	0.578407	0.424372	-1.179659
H	-0.326500	0.069965	-1.694379
H	0.652333	1.498323	-1.402358
C	1.869159	-0.305009	-1.623257
H	1.629468	-1.251250	-2.124023
H	2.432005	0.292021	-2.351590
C	2.675351	-0.568364	-0.325135
H	3.213668	-1.526722	-0.404139
C	2.068342	-0.479088	2.211320
H	2.871824	-1.187455	2.455896
H	2.456448	0.536196	2.352815
H	1.266155	-0.639396	2.944789
C	3.756113	0.514895	-0.046949
H	4.176981	0.302698	0.949764
C	4.920777	0.393069	-1.047440
H	5.364067	-0.612139	-1.025416
H	4.594773	0.592805	-2.078595
H	5.712947	1.116773	-0.810788
C	3.219856	1.958931	-0.028506
H	4.007769	2.655726	0.288323
H	2.893693	2.275276	-1.030366
H	2.366824	2.082349	0.654717
H	-0.502823	0.362908	2.172521
N	-2.108628	3.363729	1.860100
H	-2.398484	4.191839	1.332287
H	-1.097810	3.394514	2.029205
H	-2.605944	3.332064	2.754758
H	-2.292471	2.441784	1.277042

H-I-TS* G838.274515, T-1123

H-I-TS*–NH_3 G781.711354

C	3.585135	-0.430570	-1.066008
C	3.461144	1.123799	-1.005021
C	2.032390	1.578600	-1.184140

C	-0.579178	-0.242652	0.383295
C	1.848678	-0.470294	0.744670
C	2.516033	-1.208827	-0.246380
H	3.881995	1.492952	-0.059446
H	4.102231	1.530733	-1.804259
H	3.453822	-0.754745	-2.110728
H	4.615600	-0.720960	-0.798858
C	1.898865	-2.415096	-0.945618
H	1.133516	-2.893659	-0.321537
H	1.451986	-2.164475	-1.916046
H	2.680137	-3.162829	-1.154672
C	0.499652	-0.391066	1.220507
H	2.499047	0.235432	1.277230
C	1.256463	2.265299	-0.311244
H	1.563687	1.246033	-2.117728
C	-0.216083	2.532705	-0.610493
H	-0.485788	2.104345	-1.583806
H	-0.325663	3.625096	-0.728628
C	1.773403	2.871918	0.974481
H	1.361862	2.364567	1.861187
H	2.865661	2.855084	1.052797
H	1.455396	3.922558	1.050033
C	-1.751088	0.653789	0.742664
C	-1.248354	2.149308	0.498859
H	-0.843510	2.502384	1.458063
H	-2.157700	2.741294	0.319534
C	-0.700075	-0.658151	-1.058839
H	0.163167	-0.307782	-1.640041
H	-0.678289	-1.756014	-1.115682
C	-2.051203	-0.105244	-1.571385
H	-1.892993	0.776207	-2.205564
H	-2.569538	-0.843373	-2.195118
C	-2.860953	0.273216	-0.305756
H	-3.471908	1.168839	-0.502308
C	-2.220758	0.580160	2.207406
H	-3.082519	1.243591	2.361996
H	-2.515186	-0.434748	2.495834
H	-1.433425	0.911494	2.897541
C	-3.846617	-0.842032	0.144178
H	-4.272360	-0.526423	1.110751
C	-5.028290	-0.950551	-0.838164
H	-5.550521	0.010294	-0.945751
H	-4.699777	-1.267944	-1.838582
H	-5.756666	-1.693148	-0.485036
C	-3.193457	-2.221554	0.348461
H	-3.918068	-2.927770	0.775136
H	-2.853753	-2.648299	-0.606822
H	-2.327376	-2.187073	1.025586
H	0.396942	0.030427	2.223813
N	4.215004	-2.653715	1.421371
H	4.811254	-3.189117	0.788244
H	3.704760	-3.311332	2.013872

H	4.814363	-2.081261	2.018519
H	3.320350	-1.870916	0.722041

I* G838.282034

I*–NH_3 G781.718873

C	3.467465	-0.395298	-1.198154
C	3.370370	1.147443	-1.095479
C	1.934468	1.584527	-1.207533
C	-0.613105	-0.254005	0.391853
C	1.836907	-0.381478	0.763116
C	2.573399	-1.211547	-0.185312
H	3.839103	1.504011	-0.167842
H	3.964778	1.566966	-1.923994
H	3.137737	-0.699140	-2.204252
H	4.515276	-0.712709	-1.105488
C	1.914108	-2.445279	-0.811610
H	1.217246	-2.925587	-0.112271
H	1.384895	-2.226799	-1.745730
H	2.697423	-3.176097	-1.054200
C	0.503054	-0.297048	1.211762
H	2.507534	0.256386	1.351301
C	1.183742	2.293793	-0.326978
H	1.428229	1.224844	-2.111317
C	-0.302067	2.522811	-0.594696
H	-0.578529	2.089240	-1.563797
H	-0.438612	3.611972	-0.708835
C	1.738925	2.960891	0.908807
H	1.303082	2.543550	1.829813
H	2.828534	2.887981	0.986975
H	1.477574	4.029912	0.906017
C	-1.800631	0.607211	0.758868
C	-1.310346	2.111846	0.528007
H	-0.894676	2.452043	1.486578
H	-2.231064	2.690672	0.368780
C	-0.739761	-0.714177	-1.027358
H	0.125863	-0.392090	-1.621628
H	-0.713022	-1.814888	-1.036218
C	-2.091928	-0.177962	-1.551668
H	-1.932529	0.693817	-2.198797
H	-2.604064	-0.927965	-2.165375
C	-2.905471	0.214264	-0.292832
H	-3.515926	1.107337	-0.500325
C	-2.262738	0.518118	2.226247
H	-3.129747	1.173469	2.384227
H	-2.547932	-0.500686	2.508141
H	-1.478186	0.852323	2.917444
C	-3.888377	-0.899352	0.166948
H	-4.317614	-0.575610	1.129063
C	-5.067419	-1.020777	-0.817437
H	-5.592688	-0.062874	-0.934845
H	-4.736338	-1.347348	-1.813932
H	-5.793418	-1.762221	-0.457883

C	-3.229045	-2.273647	0.384956
H	-3.949117	-2.977530	0.822177
H	-2.890148	-2.711303	-0.565599
H	-2.361675	-2.231120	1.061008
H	0.389823	0.183526	2.186745
N	4.877115	-2.495612	1.362221
H	5.344282	-3.134885	0.718047
H	4.575251	-3.061305	2.156622
H	5.599579	-1.866353	1.714427
H	3.359972	-1.634253	0.540368

**I**	G781.725886		
C	3.118842	-0.620513	-1.290655
C	2.634667	0.802651	-1.672593
C	1.181925	1.138205	-1.429210
C	0.220801	-0.420302	0.547532
C	2.643852	-0.363776	1.311710
C	3.525760	-0.837436	0.185458
H	3.288379	1.539578	-1.185855
H	2.800618	0.922511	-2.756226
H	2.414180	-1.392006	-1.628617
H	4.034521	-0.813922	-1.868720
C	3.930097	-2.313614	0.455353
H	4.706602	-2.617423	-0.258951
H	4.328228	-2.447877	1.469370
H	3.069557	-2.984496	0.334293
C	1.302306	-0.110690	1.427642
H	1.013991	0.431607	2.332680
H	4.450136	-0.244811	0.323374
H	3.216457	-0.136765	2.217596
C	0.724827	2.118018	-0.591626
H	0.451057	0.646143	-2.077148
C	-0.748038	2.364258	-0.417887
H	-1.318246	2.017004	-1.289062
H	-0.939239	3.442572	-0.311976
C	1.663870	2.927207	0.267418
H	1.122863	3.531741	1.004468
H	2.382951	2.290706	0.805416
H	2.257937	3.613199	-0.355715
C	-1.184870	0.113266	0.795653
C	-1.249689	1.661050	0.866460
H	-0.648510	2.005836	1.722935
H	-2.278508	1.968831	1.080494
C	0.151838	-1.651850	-0.321581
H	0.610632	-1.459058	-1.297368
H	0.729733	-2.473443	0.122963
C	-1.350616	-1.940572	-0.512216
H	-1.544677	-2.392784	-1.489993
H	-1.709830	-2.648596	0.249824
C	-2.044514	-0.572882	-0.335749
H	-1.887706	0.004694	-1.266594
C	-1.610531	-0.440259	2.204321

H	-2.653233	-0.159934	2.389528
H	-1.534264	-1.531707	2.264568
H	-0.997908	-0.007739	3.002977
C	-3.577432	-0.702835	-0.140918
H	-3.756409	-1.331334	0.749153
C	-4.194992	-1.434868	-1.351150
H	-3.806928	-2.453986	-1.470283
H	-5.283525	-1.510625	-1.233295
H	-4.000800	-0.884662	-2.284660
C	-4.304262	0.639331	0.064112
H	-5.390818	0.488658	0.023835
H	-4.086199	1.102244	1.034533
H	-4.044089	1.359512	-0.725994

I-J-TS G781.727175, T-391

C	3.033378	-0.932233	-1.130707
C	2.074356	-0.004435	-1.907068
C	0.766423	0.526080	-1.297009
C	0.273374	-0.350822	0.410528
C	2.701115	-0.092506	1.300845
C	3.647362	-0.394821	0.169474
H	2.659885	0.863875	-2.248236
H	1.787464	-0.539679	-2.823555
H	2.578260	-1.909760	-0.924523
H	3.863442	-1.144290	-1.820877
C	4.749726	-1.357307	0.676879
H	5.516832	-1.500805	-0.094683
H	5.242006	-0.965773	1.576592
H	4.323257	-2.339394	0.924029
C	1.349712	-0.054871	1.385543
H	0.978619	0.316000	2.344438
H	4.148124	0.567965	-0.063197
H	3.223637	0.187748	2.221910
C	0.628101	1.855684	-0.829980
H	-0.121104	0.170863	-1.827923
C	-0.740600	2.359586	-0.523609
H	-1.456852	2.081523	-1.306848
H	-0.746918	3.450662	-0.415485
C	1.793747	2.650833	-0.379558
H	1.553690	3.705081	-0.211868
H	2.115704	2.206674	0.587738
H	2.661473	2.555098	-1.043466
C	-1.151022	0.173329	0.785451
C	-1.186919	1.712948	0.828383
H	-0.518120	2.071317	1.626560
H	-2.187576	2.085323	1.070466
C	0.051785	-1.795026	-0.072371
H	0.746565	-2.111512	-0.852523
H	0.263236	-2.443522	0.791996
C	-1.424169	-1.892508	-0.525293
H	-1.499822	-2.190272	-1.577810
H	-1.944182	-2.666636	0.056220

C	-2.088358	-0.509171	-0.277169
H	-2.026758	0.076983	-1.212734
C	-1.518351	-0.356774	2.206673
H	-2.548817	-0.075619	2.446552
H	-1.446594	-1.449202	2.269879
H	-0.878103	0.072526	2.984904
C	-3.605185	-0.636729	0.023038
H	-3.727241	-1.281855	0.910572
C	-4.305376	-1.339665	-1.158723
H	-3.905475	-2.343022	-1.350063
H	-5.378917	-1.445956	-0.956127
H	-4.200654	-0.750733	-2.083137
C	-4.308442	0.704713	0.299324
H	-5.395011	0.557172	0.350869
H	-4.006751	1.162279	1.249777
H	-4.117235	1.429117	-0.507336

**J**	G781.734031		
C	3.021667	-1.007312	-1.065477
C	1.956870	-0.219421	-1.849348
C	0.700284	0.365639	-1.148328
C	0.293025	-0.332005	0.329090
C	2.711047	-0.053858	1.291913
C	3.659727	-0.322665	0.149423
H	2.453998	0.603018	-2.386733
H	1.579951	-0.887290	-2.636881
H	2.628969	-1.978977	-0.734834
H	3.819738	-1.243373	-1.785079
C	4.852455	-1.161638	0.666432
H	5.595934	-1.304088	-0.128284
H	5.349576	-0.669576	1.512672
H	4.514986	-2.152401	1.001614
C	1.360955	-0.074236	1.364257
H	0.970800	0.175669	2.352717
H	4.074606	0.654884	-0.170813
H	3.220772	0.192099	2.229686
C	0.643658	1.793677	-0.868439
H	-0.180862	0.113148	-1.754289
C	-0.689119	2.368358	-0.604720
H	-1.416856	2.075823	-1.373020
H	-0.665302	3.460302	-0.519566
C	1.843605	2.569178	-0.539773
H	1.674513	3.647437	-0.471286
H	2.128357	2.179115	0.469220
H	2.706028	2.332805	-1.174006
C	-1.137405	0.203943	0.760627
C	-1.152682	1.742873	0.767623
H	-0.479783	2.111355	1.556941
H	-2.146295	2.150704	0.981869
C	0.005578	-1.828400	0.009965
H	0.712257	-2.261967	-0.701729
H	0.136516	-2.386887	0.947213

C	-1.454622	-1.890175	-0.497328
H	-1.498693	-2.177878	-1.555058
H	-2.010812	-2.659246	0.056545
C	-2.102926	-0.492260	-0.266738
H	-2.059131	0.073234	-1.217453
C	-1.500074	-0.270897	2.195959
H	-2.528613	0.018138	2.434707
H	-1.430590	-1.360183	2.299300
H	-0.860120	0.184814	2.959231
C	-3.615903	-0.596108	0.060382
H	-3.731062	-1.223173	0.961610
C	-4.344347	-1.310155	-1.097154
H	-3.953526	-2.317867	-1.283942
H	-5.414235	-1.407470	-0.871698
H	-4.254955	-0.733685	-2.031049
C	-4.296601	0.759707	0.322106
H	-5.383864	0.628543	0.398262
H	-3.970413	1.234074	1.256242
H	-4.113038	1.463417	-0.504929

**J**	G781.734032		
C	3.021502	-1.007147	-1.065624
C	1.956848	-0.218750	-1.849264
C	0.700161	0.365915	-1.147903
C	0.293152	-0.331816	0.328736
C	2.711084	-0.053751	1.291818
C	3.659741	-0.322943	0.149439
H	2.454204	0.603924	-2.386102
H	1.580030	-0.886164	-2.637236
H	2.628584	-1.978815	-0.735237
H	3.819470	-1.243150	-1.785340
C	4.852046	-1.162521	0.666453
H	5.595500	-1.305279	-0.128238
H	5.349314	-0.670701	1.512746
H	4.514096	-2.153141	1.001569
C	1.361002	-0.073990	1.364059
H	0.970847	0.176017	2.352509
H	4.075167	0.654432	-0.170657
H	3.220779	0.192179	2.229615
C	0.643568	1.794124	-0.868325
H	-0.180888	0.113617	-1.754232
C	-0.689188	2.368632	-0.604165
H	-1.416922	2.076335	-1.372590
H	-0.665435	3.460561	-0.518709
C	1.843470	2.569580	-0.539827
H	1.674607	3.647875	-0.471448
H	2.127246	2.179579	0.469615
H	2.706347	2.332865	-1.173236
C	-1.137341	0.203794	0.760609
C	-1.152762	1.742704	0.767950
H	-0.479897	2.111036	1.557376
H	-2.146418	2.150414	0.982243

C	0.005796	-1.828310	0.009722
H	0.712552	-2.261813	-0.701933
H	0.136746	-2.386713	0.947019
C	-1.454371	-1.890128	-0.497661
H	-1.498360	-2.177642	-1.555448
H	-2.010566	-2.659322	0.056048
C	-2.102835	-0.492312	-0.266834
H	-2.059171	0.073367	-1.217462
C	-1.499998	-0.271364	2.195812
H	-2.528530	0.017638	2.434629
H	-1.430553	-1.360679	2.298929
H	-0.860064	0.184152	2.959217
C	-3.615796	-0.596339	0.060329
H	-3.730850	-1.223553	0.961463
C	-4.344214	-1.310245	-1.097313
H	-3.953292	-2.317876	-1.284326
H	-5.414080	-1.407723	-0.871815
H	-4.254941	-0.733579	-2.031099
C	-4.296570	0.759389	0.322290
H	-5.383818	0.628134	0.398557
H	-3.970317	1.233679	1.256441
H	-4.113156	1.463220	-0.504676

J-K-TS G781.730340, T-515

C	3.081538	-0.338751	-1.284382
C	1.885038	0.586744	-1.606325
C	0.851893	0.664004	-0.492953
C	0.338182	-0.652232	0.123304
C	2.728333	-0.848930	1.169062
C	3.633924	-0.193396	0.143606
H	2.272008	1.586845	-1.826370
H	1.391804	0.227167	-2.521634
H	2.817809	-1.392495	-1.448032
H	3.865489	-0.092123	-2.014041
C	5.049192	-0.802040	0.234315
H	5.736290	-0.296888	-0.456688
H	5.455752	-0.705734	1.249620
H	5.030627	-1.871037	-0.021333
C	1.403509	-1.083188	1.130584
H	1.001190	-1.635902	1.981055
H	3.722496	0.885864	0.385528
H	3.249909	-1.229411	2.052700
C	0.443589	1.928345	0.021312
H	-0.185126	1.325533	-0.984038
C	-0.568433	2.050287	1.134645
H	-1.433879	2.601891	0.727653
H	-0.125502	2.740012	1.872175
C	1.036314	3.240470	-0.454746
H	1.173330	3.298567	-1.538909
H	0.401329	4.075805	-0.141394
H	2.023520	3.366624	0.014476
C	-1.081123	-0.449482	0.818484

C	-0.987963	0.738563	1.795995
H	-0.241483	0.474431	2.561224
H	-1.931748	0.894133	2.329349
C	-0.005281	-1.689920	-0.980828
H	0.728348	-1.729519	-1.792761
H	0.002275	-2.685987	-0.522380
C	-1.423878	-1.300797	-1.470111
H	-1.395523	-0.863033	-2.477085
H	-2.063773	-2.190465	-1.541024
C	-2.016564	-0.290832	-0.434461
H	-1.893675	0.738574	-0.843013
C	-1.514962	-1.692576	1.630214
H	-2.523785	-1.530357	2.026451
H	-1.543516	-2.611794	1.034232
H	-0.867125	-1.870079	2.495942
C	-3.549300	-0.444220	-0.263299
H	-3.749299	-1.478556	0.066475
C	-4.248249	-0.244163	-1.623351
H	-3.902506	-0.954838	-2.384112
H	-5.333145	-0.373849	-1.518128
H	-4.074439	0.773576	-2.007201
C	-4.158587	0.521330	0.768399
H	-5.254398	0.461136	0.743308
H	-3.849446	0.301757	1.797222
H	-3.886843	1.564847	0.543948

**K**	G781.751201		
C	3.010684	-0.925526	-1.229762
C	1.980123	0.122620	-1.709382
C	0.925048	0.606137	-0.752810
C	0.295816	-0.311186	0.224230
C	2.788969	-0.038476	1.156713
C	3.701593	-0.530137	0.075776
H	2.503057	0.998400	-2.121472
H	1.405202	-0.304070	-2.554780
H	2.560325	-1.916605	-1.110460
H	3.766871	-1.028561	-2.019501
C	4.593445	-1.682217	0.610287
H	5.324769	-1.972742	-0.153919
H	5.141486	-1.377387	1.510508
H	3.982418	-2.560195	0.858377
C	1.435222	0.099464	1.210297
H	1.052032	0.688478	2.045386
H	4.377530	0.326962	-0.121945
H	3.317332	0.309242	2.051601
C	0.531124	2.052477	-0.898879
H	0.610627	2.239227	-1.986749
C	-0.903837	2.367078	-0.427420
H	-1.616791	2.079871	-1.212865
H	-1.002816	3.453672	-0.299925
C	1.583659	2.996867	-0.243510
H	2.610056	2.735386	-0.529366

H	1.385348	4.025692	-0.565351
H	1.508780	2.949854	0.848947
C	-1.126612	0.111749	0.780614
C	-1.246280	1.648332	0.875592
H	-0.582729	2.010165	1.680469
H	-2.254660	1.924567	1.198479
C	0.130891	-1.807931	-0.157222
H	0.740368	-2.097643	-1.015504
H	0.448550	-2.431825	0.687183
C	-1.374388	-1.975053	-0.480704
H	-1.519536	-2.346183	-1.501792
H	-1.831435	-2.715014	0.191219
C	-2.047309	-0.590313	-0.277274
H	-1.943017	-0.028108	-1.227730
C	-1.364973	-0.491029	2.183294
H	-2.375625	-0.237913	2.524565
H	-1.276453	-1.584133	2.201742
H	-0.669736	-0.089561	2.932143
C	-3.566200	-0.729033	0.004347
H	-3.683707	-1.367438	0.898000
C	-4.246094	-1.455907	-1.175387
H	-3.837070	-2.459341	-1.345705
H	-5.321629	-1.566668	-0.985952
H	-4.131785	-0.879707	-2.106624
C	-4.297616	0.599442	0.269563
H	-5.383070	0.434725	0.280943
H	-4.035655	1.043422	1.237608
H	-4.088620	1.339290	-0.517708

**K**	G781.751204		
C	3.010630	-0.926022	-1.229542
C	1.979773	0.121852	-1.709462
C	0.924931	0.605693	-0.752893
C	0.295689	-0.311359	0.224402
C	2.788878	-0.038452	1.156693
C	3.701608	-0.529855	0.075695
H	2.502715	0.997436	-2.122018
H	1.404882	-0.305296	-2.554582
H	2.560447	-1.917110	-1.109674
H	3.766674	-1.029258	-2.019386
C	4.594426	-1.681055	0.610290
H	5.325853	-1.971235	-0.153938
H	5.142409	-1.375643	1.510363
H	3.984136	-2.559474	0.858717
C	1.435114	0.099385	1.210267
H	1.051988	0.688412	2.045383
H	4.376806	0.327767	-0.122439
H	3.317274	0.309357	2.051528
C	0.531246	2.052109	-0.899154
H	0.610754	2.238903	-1.987020
C	-0.903661	2.366963	-0.427668
H	-1.616677	2.079632	-1.213030

H	-1.002547	3.453592	-0.300475
C	1.584044	2.996219	-0.243895
H	2.610272	2.734823	-0.530477
H	1.385651	4.025183	-0.565228
H	1.509896	2.948822	0.848590
C	-1.126717	0.111876	0.780664
C	-1.246115	1.648518	0.875477
H	-0.582512	2.010338	1.680282
H	-2.254460	1.924918	1.198315
C	0.130534	-1.808176	-0.156518
H	0.740500	-2.098600	-1.014199
H	0.447415	-2.431767	0.688414
C	-1.374622	-1.974951	-0.480793
H	-1.519372	-2.345592	-1.502143
H	-1.832116	-2.715126	0.190581
C	-2.047433	-0.590130	-0.277206
H	-1.943181	-0.027991	-1.227686
C	-1.365119	-0.490743	2.183358
H	-2.375650	-0.237315	2.524728
H	-1.276934	-1.583867	2.201830
H	-0.669680	-0.089459	2.932123
C	-3.566306	-0.728822	0.004418
H	-3.683825	-1.367292	0.898018
C	-4.246189	-1.455601	-1.175398
H	-3.836947	-2.458908	-1.345950
H	-5.321674	-1.566621	-0.985887
H	-4.132064	-0.879193	-2.106534
C	-4.297753	0.599640	0.269690
H	-5.383186	0.434847	0.281297
H	-4.035636	1.043752	1.237644
H	-4.088944	1.339422	-0.517704

K-L-TS G781.754319, T-187

C	3.028773	-0.941695	-1.232012
C	2.057181	0.171396	-1.677035
C	0.992170	0.646696	-0.711950
C	0.280502	-0.290960	0.167206
C	2.780599	-0.049928	1.137138
C	3.695688	-0.624528	0.107878
H	2.633101	1.050155	-2.004202
H	1.498403	-0.168905	-2.568561
H	2.535347	-1.917011	-1.167107
H	3.803146	-1.045154	-2.003930
C	4.452797	-1.853427	0.681835
H	5.187589	-2.206246	-0.052047
H	4.985282	-1.601152	1.607139
H	3.753127	-2.672044	0.895257
C	1.432282	0.173248	1.118476
H	1.052604	0.817894	1.911553
H	4.456336	0.168197	-0.045850
H	3.290483	0.312449	2.037052
C	0.599477	2.103443	-0.865602

H	0.722705	2.287647	-1.948998
C	-0.868138	2.379628	-0.477738
H	-1.528016	2.058165	-1.296200
H	-1.008408	3.464258	-0.372181
C	1.593376	3.065062	-0.163277
H	2.637717	2.816568	-0.392855
H	1.402634	4.091126	-0.498886
H	1.465977	3.031880	0.925476
C	-1.129911	0.131804	0.740181
C	-1.262850	1.668873	0.815082
H	-0.640333	2.044470	1.646047
H	-2.286817	1.937418	1.090784
C	0.134317	-1.779263	-0.235879
H	0.693650	-2.018028	-1.142920
H	0.517081	-2.425318	0.563891
C	-1.382795	-1.976234	-0.479943
H	-1.568363	-2.383110	-1.480327
H	-1.798047	-2.698344	0.236961
C	-2.065070	-0.594922	-0.288614
H	-1.988357	-0.046951	-1.249009
C	-1.334760	-0.454860	2.155872
H	-2.338957	-0.204221	2.517280
H	-1.237430	-1.547121	2.185167
H	-0.623860	-0.039320	2.882069
C	-3.575759	-0.747392	0.031902
H	-3.664610	-1.370668	0.939341
C	-4.273687	-1.504271	-1.118171
H	-3.858349	-2.506773	-1.278207
H	-5.343321	-1.622466	-0.901623
H	-4.187041	-0.945355	-2.062720
C	-4.318323	0.576449	0.289731
H	-5.401286	0.398767	0.322982
H	-4.045427	1.038187	1.246560
H	-4.131772	1.306275	-0.512412

**L**	G781.756135		
C	3.104677	-0.880208	-1.214728
C	2.236828	0.344281	-1.566958
C	1.144248	0.765644	-0.568291
C	0.228834	-0.220555	-0.005059
C	2.703207	-0.243595	1.209976
C	3.627927	-0.861028	0.228140
H	2.894567	1.218932	-1.688203
H	1.754810	0.185662	-2.544055
H	2.564062	-1.819852	-1.379536
H	3.962574	-0.910974	-1.900356
C	4.144142	-2.245138	0.700364
H	4.904835	-2.608899	-0.000470
H	4.594658	-2.189595	1.699294
H	3.325193	-2.975629	0.727462
C	1.519560	0.401636	0.939045
H	1.086562	0.999782	1.737264

H	4.502754	-0.173501	0.286740
H	3.065088	-0.200716	2.243708
C	0.694115	2.239074	-0.779975
H	0.897321	2.417459	-1.849876
C	-0.820050	2.413814	-0.554460
H	-1.367221	2.023650	-1.426367
H	-1.053156	3.486256	-0.493750
C	1.542670	3.251738	0.009336
H	2.618802	3.055579	-0.095624
H	1.349983	4.266555	-0.359802
H	1.302893	3.242757	1.081168
C	-1.122388	0.167343	0.648445
C	-1.302112	1.701515	0.709972
H	-0.759096	2.095842	1.585757
H	-2.352913	1.936480	0.900018
C	0.151116	-1.677371	-0.461319
H	0.636174	-1.798840	-1.435112
H	0.646777	-2.359926	0.239465
C	-1.368335	-1.956508	-0.569469
H	-1.614261	-2.386712	-1.546283
H	-1.687514	-2.683682	0.189741
C	-2.090033	-0.601427	-0.336127
H	-2.095936	-0.046414	-1.293256
C	-1.250035	-0.428286	2.073678
H	-2.244464	-0.206051	2.476949
H	-1.117340	-1.517096	2.094379
H	-0.517356	0.011170	2.761886
C	-3.570344	-0.814977	0.085414
H	-3.572872	-1.441221	0.994747
C	-4.304665	-1.603057	-1.020863
H	-3.861641	-2.589088	-1.205742
H	-5.352725	-1.763017	-0.735949
H	-4.300785	-1.043629	-1.968667
C	-4.352243	0.474965	0.390539
H	-5.419925	0.245279	0.503011
H	-4.033573	0.957261	1.322852
H	-4.258976	1.204999	-0.427347

**L**	G781.759897		
C	2.953618	-1.156083	-1.190471
C	2.234312	0.150942	-1.581004
C	1.204524	0.709545	-0.603287
C	0.190610	-0.157481	-0.012934
C	2.589182	-0.418700	1.223403
C	3.471122	-1.121799	0.252958
H	2.984669	0.927857	-1.778565
H	1.696693	-0.003466	-2.531211
H	2.308031	-2.027923	-1.336508
H	3.807488	-1.300942	-1.865865
C	3.870582	-2.529600	0.778520
H	4.606456	-2.971797	0.096494
H	4.316906	-2.475912	1.779105

H	2.997034	-3.192498	0.821882
C	1.358311	0.162392	1.030277
H	1.025432	0.841777	1.811607
H	4.399510	-0.513046	0.285837
H	3.043697	-0.238163	2.204625
C	1.059410	2.232265	-0.621582
H	0.926691	2.445055	-1.702128
C	-0.193639	2.736372	0.110266
H	-0.394402	3.765514	-0.218248
H	0.006801	2.807481	1.191909
C	2.323012	2.979401	-0.150512
H	3.225475	2.689357	-0.702144
H	2.184185	4.058537	-0.288240
H	2.503826	2.799841	0.918445
C	-1.235373	0.412152	0.395488
C	-1.407490	1.846180	-0.158937
H	-2.301610	2.304102	0.278759
H	-1.582358	1.795866	-1.246100
C	0.013362	-1.630430	-0.451766
H	0.186334	-1.699952	-1.535777
H	0.716567	-2.306824	0.044671
C	-1.447372	-1.968829	-0.143760
H	-1.820005	-2.751898	-0.812800
H	-1.559949	-2.339679	0.886965
C	-2.188155	-0.631107	-0.310082
H	-2.183063	-0.379441	-1.390188
C	-1.454686	0.396250	1.924921
H	-2.500745	0.622937	2.155673
H	-1.214956	-0.576448	2.373608
H	-0.856532	1.159366	2.438926
C	-3.673433	-0.699206	0.135156
H	-3.697847	-0.995309	1.198571
C	-4.409098	-1.797516	-0.662029
H	-4.003061	-2.799178	-0.475090
H	-5.470978	-1.818322	-0.384293
H	-4.353880	-1.602755	-1.743948
C	-4.435238	0.630081	-0.015113
H	-5.503319	0.476261	0.186657
H	-4.087924	1.405505	0.678254
H	-4.346533	1.025134	-1.037970

L-M-TS G781.738678, T-889

C	3.026007	-1.046151	-1.174494
C	2.332757	0.304567	-1.468263
C	1.284287	0.804698	-0.462501
C	0.194616	-0.119995	0.012230
C	2.417072	-0.724130	1.265608
C	3.197231	-1.396768	0.276200
H	3.119474	1.068418	-1.541369
H	1.876290	0.248230	-2.466952
H	2.442872	-1.884761	-1.590365
H	4.001946	-1.099566	-1.679054

C	4.095096	-2.545825	0.645480
H	5.060468	-2.484741	0.128695
H	4.256660	-2.625786	1.726658
H	3.605748	-3.473093	0.308740
C	1.394917	0.262146	0.958477
H	1.153379	0.928588	1.786143
H	3.598752	-0.283150	0.976659
H	2.545655	-1.062425	2.299756
C	1.017372	2.319972	-0.593617
H	0.845118	2.497058	-1.671986
C	-0.255429	2.759164	0.148655
H	-0.492026	3.789818	-0.151456
H	-0.061103	2.807936	1.233749
C	2.220530	3.169425	-0.144861
H	3.130073	2.976780	-0.728768
H	1.988697	4.236634	-0.254201
H	2.452808	2.992013	0.916095
C	-1.243790	0.401068	0.385399
C	-1.442836	1.841411	-0.145275
H	-2.353755	2.270950	0.288642
H	-1.603026	1.805488	-1.235190
C	0.060575	-1.554395	-0.527751
H	0.235746	-1.548875	-1.616678
H	0.776112	-2.264822	-0.090037
C	-1.389085	-1.962260	-0.244069
H	-1.735946	-2.727528	-0.947342
H	-1.485626	-2.383854	0.769451
C	-2.176020	-0.643835	-0.344637
H	-2.196681	-0.352351	-1.414442
C	-1.467808	0.354378	1.914515
H	-2.503855	0.612716	2.159652
H	-1.262157	-0.640238	2.332909
H	-0.831869	1.075033	2.443759
C	-3.649795	-0.776551	0.118775
H	-3.649241	-1.086837	1.178738
C	-4.361083	-1.887644	-0.682247
H	-3.918862	-2.877517	-0.513894
H	-5.418355	-1.947723	-0.392167
H	-4.325944	-1.677928	-1.762360
C	-4.457431	0.528648	-0.001483
H	-5.516078	0.340327	0.221016
H	-4.117201	1.307931	0.690768
H	-4.401171	0.935741	-1.022016

M G781.744058

C	3.004096	-1.139755	-1.074419
C	2.425714	0.264333	-1.395974
C	1.454739	0.866158	-0.371997
C	0.271117	-0.006291	0.070537
C	2.335494	-0.882259	1.367405
C	2.620024	-1.777755	0.220646
H	3.262137	0.966484	-1.519844

H	1.932463	0.220129	-2.378482
H	2.838305	-1.854011	-1.893476
H	4.109851	-1.077546	-0.995403
C	3.016127	-3.199631	0.461635
H	2.921496	-3.817753	-0.438583
H	4.085511	-3.202200	0.742575
H	2.465964	-3.649059	1.296015
C	1.440277	0.290912	1.010824
H	1.256692	0.975350	1.841009
H	3.331356	-0.476217	1.647224
H	1.970235	-1.445636	2.237471
C	1.225262	2.374836	-0.559485
H	1.127631	2.533353	-1.650155
C	-0.093931	2.851772	0.082035
H	-0.290759	3.877642	-0.259984
H	0.028472	2.926194	1.175890
C	2.415826	3.205948	-0.050580
H	3.361209	2.930240	-0.537603
H	2.248872	4.274207	-0.240500
H	2.546274	3.078789	1.034303
C	-1.166458	0.535331	0.355223
C	-1.290713	1.951597	-0.260305
H	-2.216871	2.429324	0.084460
H	-1.370661	1.864458	-1.356560
C	0.148116	-1.388966	-0.514415
H	0.371908	-1.430693	-1.591531
H	0.962060	-2.082605	-0.075461
C	-1.263796	-1.865334	-0.201459
H	-1.593233	-2.666031	-0.871419
H	-1.346504	-2.231439	0.833827
C	-2.073339	-0.546813	-0.358379
H	-2.079181	-0.296862	-1.438208
C	-1.451989	0.576169	1.873238
H	-2.482435	0.893935	2.068726
H	-1.308443	-0.405623	2.345523
H	-0.797978	1.287176	2.391872
C	-3.551384	-0.690577	0.081108
H	-3.568835	-0.928700	1.159019
C	-4.234039	-1.857592	-0.661510
H	-3.790652	-2.831008	-0.415444
H	-5.297508	-1.907074	-0.393592
H	-4.174099	-1.720604	-1.752093
C	-4.359230	0.601210	-0.145087
H	-5.417960	0.430165	0.090706
H	-4.016105	1.432895	0.480892
H	-4.300687	0.922967	-1.195419

Cartesian coordinates of computed structures (Scheme 2 of main text, Table S13)

Gibbs energies (G"..." in Hartree) and imagininary frequencies of TS (T-"..." in cm^{-1}), mPW1PW91/6-311+G(d,p)//B97D3/6-31G(d,p)-sp-density-fitting, 1 bar, 298.15 K. From the structures labelled with asterisks the energy of NH_3 (G56.563161, page 98) is subtracted for direct comparability.

**A'**	G781.674472		
C	-2.914828	-0.620636	-1.995018
C	-3.016559	-1.689311	-0.847638
C	-1.853312	-1.544528	0.106376
C	1.594463	1.785173	0.275035
C	-1.201791	1.159818	-1.654182
C	-2.478272	0.695334	-1.400352
H	-3.976602	-1.573811	-0.330291
H	-3.029824	-2.684160	-1.317428
H	-2.185190	-0.955355	-2.745439
H	-3.893709	-0.531278	-2.490778
C	-3.489204	1.439449	-0.582930
H	-3.069547	2.230070	0.047386
H	-4.202432	1.915716	-1.277038
H	-4.079508	0.761869	0.042985
C	-0.544606	2.212665	-0.972907
H	-0.613697	0.615944	-2.398398
C	-1.887409	-1.061506	1.368473
H	-0.871617	-1.780889	-0.321603
C	-0.601923	-0.723510	2.105172
H	0.221678	-1.381496	1.794952
H	-0.738264	-0.865846	3.187032
C	-3.144999	-0.742270	2.143081
H	-3.167827	0.314765	2.455016
H	-4.070529	-0.956454	1.599221
H	-3.165767	-1.334062	3.071170
C	0.572684	0.889194	0.555590
C	-0.182273	0.758339	1.858102
H	-1.079163	1.397635	1.878157
H	0.456520	1.110920	2.682070
C	2.553686	1.463537	-0.838481
H	2.034918	0.908898	-1.633232
H	2.977050	2.380199	-1.274794
C	3.718209	0.556427	-0.307492
H	4.365593	0.339169	-1.169449
H	4.327947	1.137695	0.398836
C	3.239427	-0.713806	0.359711
H	0.520639	0.019333	-0.098378
C	1.865314	3.002466	1.104681
H	2.875973	2.934225	1.538413
H	1.873771	3.902773	0.470506
H	1.147384	3.149572	1.917775
C	2.788421	-1.823288	-0.265803
H	3.250963	-0.722638	1.454211
C	2.327537	-3.030178	0.518798

H	2.388011	-2.870705	1.602920
H	1.288674	-3.299097	0.264443
H	2.938675	-3.912062	0.270217
C	2.699638	-1.963724	-1.768560
H	3.178179	-2.897853	-2.099168
H	1.645686	-2.033965	-2.089049
H	3.164421	-1.136999	-2.318457
H	-1.052497	2.797661	-0.208155
H	0.274871	2.722368	-1.473595

A‘-C‘-TS G781.674200, T-35

C	-3.126904	-0.024932	-1.998850
C	-3.208664	-1.391549	-1.232563
C	-1.970758	-1.606168	-0.393760
C	1.352862	1.619389	0.546635
C	-1.357804	1.576244	-1.265636
C	-2.614862	1.040951	-1.063388
H	-4.117429	-1.409817	-0.618659
H	-3.321647	-2.188436	-1.982993
H	-2.453600	-0.125923	-2.861484
H	-4.128101	0.229729	-2.380684
C	-3.541800	1.488867	0.027074
H	-3.042115	1.968677	0.875181
H	-4.240469	2.227647	-0.400880
H	-4.157693	0.664812	0.400959
C	-0.660362	2.412201	-0.362051
H	-0.837067	1.298524	-2.185950
C	-1.875270	-1.559094	0.954015
H	-1.044169	-1.748991	-0.963082
C	-0.516228	-1.583523	1.634720
H	0.217029	-2.136259	1.030884
H	-0.588217	-2.099555	2.603282
C	-3.042325	-1.430396	1.904793
H	-2.952593	-0.528181	2.531983
H	-4.017955	-1.402080	1.409892
H	-3.050171	-2.284770	2.598873
C	0.621132	0.449220	0.645132
C	0.027077	-0.149951	1.884577
H	-0.775887	0.492925	2.285097
H	0.802585	-0.162218	2.669302
C	2.272900	1.773926	-0.645170
H	1.811531	1.315917	-1.531824
H	2.464243	2.834228	-0.869207
C	3.624905	1.045339	-0.357616
H	4.208141	1.049598	-1.289918
H	4.201358	1.633843	0.369961
C	3.437165	-0.359745	0.171736
H	0.524777	-0.139548	-0.267723
C	1.584582	2.521706	1.729569
H	2.548906	2.270981	2.199769
H	1.652572	3.572837	1.417271
H	0.808243	2.429378	2.497434

C	3.107241	-1.452907	-0.557221
H	3.588447	-0.501694	1.246467
C	2.965000	-2.809498	0.091652
H	3.048464	-2.761621	1.184623
H	2.001249	-3.277130	-0.166920
H	3.742503	-3.495845	-0.280012
C	2.874223	-1.426063	-2.049363
H	3.453683	-2.219471	-2.544860
H	1.815490	-1.637941	-2.278562
H	3.137577	-0.472166	-2.520475
H	-1.101654	2.702664	0.589577
H	0.089311	3.095781	-0.753022

C‘ G781.717088

C	-3.053606	0.221351	-1.773699
C	-3.096977	-1.252895	-1.247965
C	-1.788081	-1.627173	-0.603264
C	0.996752	1.821450	0.407335
C	-1.267944	1.652523	-0.816741
C	-2.524277	1.163401	-0.707891
H	-3.933450	-1.367429	-0.546842
H	-3.312987	-1.914430	-2.101541
H	-2.406431	0.263771	-2.663134
H	-4.068302	0.505977	-2.094367
C	-3.452273	1.440765	0.448242
H	-2.974285	1.966495	1.282280
H	-4.287830	2.069406	0.100735
H	-3.904631	0.519156	0.837979
C	-0.465023	2.366068	0.231976
H	-0.748343	1.450253	-1.760651
C	-1.533139	-1.795894	0.713531
H	-0.933384	-1.665156	-1.291384
C	-0.104784	-1.895895	1.224796
H	0.535789	-2.349065	0.447687
H	-0.059091	-2.560192	2.101155
C	-2.579132	-1.819813	1.802766
H	-2.427797	-0.997302	2.520622
H	-3.603253	-1.751686	1.424266
H	-2.499000	-2.753267	2.381752
C	1.012284	0.258845	0.397319
C	0.468917	-0.502410	1.605296
H	-0.338192	0.096408	2.053328
H	1.241955	-0.592004	2.384347
C	1.889720	2.157232	-0.806943
H	1.325630	2.026759	-1.741807
H	2.240016	3.198152	-0.787311
C	3.063189	1.159136	-0.760839
H	3.401039	0.865734	-1.762807
H	3.939686	1.573579	-0.246340
C	2.574413	-0.064872	0.094380
H	0.428267	-0.048040	-0.483749
C	1.594216	2.402546	1.703326

H	2.642258	2.108921	1.854897
H	1.567629	3.499754	1.669039
H	1.027149	2.083866	2.587737
C	2.636608	-1.364799	-0.548969
H	3.085193	-0.099757	1.066272
C	3.206481	-2.526014	0.178513
H	3.023487	-2.467198	1.258752
H	2.898568	-3.497162	-0.223527
H	4.306663	-2.450653	0.058524
C	2.214130	-1.555192	-1.959135
H	3.113899	-1.797798	-2.555009
H	1.564803	-2.437806	-2.054584
H	1.731516	-0.677924	-2.398711
H	-0.974452	2.331125	1.204888
H	-0.350957	3.437356	-0.014706

**C‘**	G781.717081		
C	-3.053643	0.221653	-1.773513
C	-3.096807	-1.252742	-1.248167
C	-1.787876	-1.626937	-0.603478
C	0.996840	1.821634	0.407211
C	-1.267988	1.652951	-0.816724
C	-2.524169	1.163505	-0.707581
H	-3.933299	-1.367587	-0.547121
H	-3.312642	-1.914092	-2.101930
H	-2.406655	0.264358	-2.663067
H	-4.068428	0.506282	-2.093894
C	-3.451905	1.440378	0.448875
H	-4.287568	2.069136	0.101832
H	-3.904128	0.518586	0.838330
H	-2.973745	1.965777	1.283025
C	-0.464923	2.366501	0.231871
H	-0.748625	1.450964	-1.760827
C	-1.533043	-1.796001	0.713299
H	-0.933120	-1.664525	-1.291540
C	-0.104757	-1.895645	1.224787
H	0.536072	-2.348676	0.447764
H	-0.059020	-2.559943	2.101142
C	-2.579209	-1.820528	1.802375
H	-2.498634	-2.753927	2.381380
H	-2.428471	-0.997907	2.520228
H	-3.603302	-1.753007	1.423680
C	1.012085	0.259109	0.397280
C	0.468592	-0.502026	1.605269
H	-0.338708	0.096690	2.053094
H	1.241509	-0.591413	2.384463
C	1.889857	2.157138	-0.807127
H	1.325701	2.026761	-1.741962
H	2.240451	3.197959	-0.787556
C	3.063077	1.158741	-0.761043
H	3.400747	0.865109	-1.762997
H	3.939719	1.572990	-0.246634

C	2.574046	-0.065079	0.094434
H	0.428065	-0.047757	-0.483783
C	1.594420	2.402771	1.703134
H	2.642382	2.108892	1.854774
H	1.568105	3.499978	1.668695
H	1.027249	2.084337	2.587569
C	2.636292	-1.365136	-0.548758
H	3.084855	-0.099852	1.066315
C	2.213701	-1.555685	-1.958854
H	3.113773	-1.796849	-2.554900
H	1.565494	-2.439060	-2.054601
H	1.730091	-0.678804	-2.398124
C	3.206672	-2.526091	0.178759
H	3.023853	-2.467185	1.259020
H	2.898958	-3.497380	-0.223093
H	4.306805	-2.450407	0.058534
H	-0.974303	2.331738	1.204818
H	-0.350751	3.437750	-0.014921

C‘-D‘-TS G781.703422, T-807

C	-3.369568	0.143560	-1.761517
C	-3.358799	-1.366940	-1.331658
C	-2.004588	-1.801548	-0.838457
C	0.709836	1.643141	0.514676
C	-1.577611	1.387047	-0.610689
C	-2.877933	1.028991	-0.635958
H	-4.131287	-1.524413	-0.566281
H	-3.656076	-1.967062	-2.204990
H	-2.723697	0.260572	-2.645189
H	-4.394336	0.405517	-2.066747
C	-3.872994	1.359987	0.448936
H	-4.614823	2.076671	0.061503
H	-4.439152	0.472388	0.765539
H	-3.416261	1.811764	1.337527
C	-0.804300	1.994604	0.531442
H	-0.999389	1.151999	-1.510839
C	-1.599686	-1.877326	0.447978
H	-1.253207	-1.988824	-1.616386
C	-0.146719	-2.150104	0.791397
H	0.374393	-2.532627	-0.099076
H	-0.086510	-2.934243	1.564644
C	-2.504866	-1.655257	1.639783
H	-2.289952	-2.387557	2.432362
H	-2.361075	-0.656389	2.080448
H	-3.565747	-1.740151	1.385325
C	0.993888	0.119757	0.230926
C	0.575721	-0.880093	1.331664
H	-0.110970	-0.360307	2.011577
H	1.438553	-1.149433	1.960605
C	1.436964	2.328628	-0.667597
H	0.815934	2.290944	-1.572871
H	1.649632	3.385314	-0.463488

C	2.725247	1.513705	-0.887179
H	2.994843	1.400075	-1.947047
H	3.587795	1.986710	-0.396437
C	2.437173	0.137749	-0.269917
H	0.409644	-0.138877	-0.678123
C	1.345361	2.081906	1.851181
H	2.434676	1.916219	1.882921
H	1.182055	3.155521	2.011959
H	0.907814	1.548760	2.705141
C	3.400986	-0.911775	-0.374524
H	3.236323	-0.057340	0.724379
C	4.724004	-0.631842	-1.050048
H	5.050226	0.409312	-0.976292
H	5.511961	-1.290598	-0.670180
H	4.590650	-0.855029	-2.121542
C	3.187025	-2.344601	0.028075
H	3.034713	-2.905958	-0.908783
H	4.089446	-2.753270	0.498007
H	2.323759	-2.519812	0.664519
H	-1.237464	1.674432	1.492255
H	-0.867321	3.098156	0.534480

D' G781.726115

C	-3.023512	-0.340279	-1.834417
C	-3.025903	-1.700532	-1.039217
C	-1.730240	-1.964525	-0.323008
C	0.721397	1.853774	0.307268
C	-1.422070	1.302432	-0.919737
C	-2.691150	0.821299	-0.930130
H	-3.872771	-1.696522	-0.339590
H	-3.223017	-2.500269	-1.768140
H	-2.287691	-0.413017	-2.649190
H	-4.017563	-0.222924	-2.292021
C	-3.795014	1.328414	-0.038758
H	-4.503751	1.912316	-0.648240
H	-4.375358	0.507775	0.404017
H	-3.442739	1.983020	0.766361
C	-0.775834	2.214319	0.087419
H	-0.777594	0.955332	-1.735368
C	-1.458369	-1.694611	0.976681
H	-0.917823	-2.372199	-0.937330
C	-0.056280	-1.845379	1.530428
H	0.554422	-2.469446	0.861843
H	-0.085299	-2.359344	2.504078
C	-2.475693	-1.159085	1.960146
H	-2.326037	-1.608339	2.952394
H	-2.383104	-0.069868	2.086736
H	-3.506915	-1.359145	1.653519
C	0.930181	0.285460	0.423309
C	0.614127	-0.462970	1.745339
H	-0.058000	0.176754	2.331699
H	1.531182	-0.568378	2.343334

C	1.574160	2.201391	-0.947781
H	0.990533	2.081504	-1.871095
H	1.931879	3.237738	-0.925032
C	2.725651	1.184087	-0.935281
H	3.182585	0.935641	-1.905645
H	3.573934	1.549741	-0.318082
C	2.213667	0.002429	-0.200744
H	0.161564	-0.125198	-0.319391
C	1.269645	2.585001	1.547771
H	2.340788	2.392342	1.705838
H	1.141637	3.669195	1.430238
H	0.741187	2.287730	2.462762
C	2.947220	-1.285871	-0.157628
H	2.606612	-1.868328	0.709947
C	4.482598	-1.134399	-0.107702
H	4.799651	-0.557117	0.770288
H	4.937861	-2.129919	-0.041731
H	4.875596	-0.647104	-1.009086
C	2.517041	-2.077053	-1.442968
H	2.853053	-1.561872	-2.351303
H	2.996624	-3.062798	-1.404530
H	1.431368	-2.218179	-1.496293
H	-1.310154	2.164520	1.048243
H	-0.812398	3.272948	-0.225169

**D'**	G781.719808		
C	-3.086068	-0.217187	-1.751467
C	-2.994020	-1.650146	-1.102432
C	-1.649841	-1.926550	-0.487428
C	0.740373	1.880985	0.377962
C	-1.466159	1.353628	-0.751182
C	-2.727686	0.854238	-0.751177
H	-3.798533	-1.751878	-0.361573
H	-3.203551	-2.380267	-1.897912
H	-2.402949	-0.179601	-2.613176
H	-4.110716	-0.088980	-2.131865
C	-3.789311	1.242429	0.244577
H	-4.539782	1.871926	-0.260652
H	-4.331533	0.367044	0.627011
H	-3.403649	1.815308	1.095506
C	-0.784915	2.169751	0.313265
H	-0.858766	1.103181	-1.628175
C	-1.315475	-1.776616	0.816404
H	-0.855892	-2.221433	-1.184722
C	0.121970	-1.919384	1.274108
H	0.708386	-2.459349	0.518310
H	0.166712	-2.520275	2.196228
C	-2.294830	-1.400847	1.906165
H	-2.092330	-1.978300	2.819906
H	-2.210868	-0.337671	2.178384
H	-3.336429	-1.577786	1.622137
C	1.043721	0.320165	0.321352

C	0.767450	-0.542787	1.577755
H	0.083731	0.032703	2.215260
H	1.689002	-0.664733	2.159802
C	1.464501	2.407484	-0.893128
H	0.816639	2.343633	-1.778195
H	1.764955	3.456946	-0.788389
C	2.662174	1.467849	-1.064343
H	3.052158	1.326910	-2.085694
H	3.541155	1.826256	-0.484326
C	2.306199	0.189344	-0.400578
H	0.282181	-0.046192	-0.449509
C	1.349138	2.516120	1.643534
H	2.438721	2.377107	1.697414
H	1.152073	3.596262	1.651245
H	0.916154	2.095277	2.559990
C	3.203963	-0.983974	-0.607435
H	4.136950	-0.580455	-1.034576
C	2.558716	-1.849841	-1.743503
H	2.330590	-1.253396	-2.635803
H	3.282152	-2.625517	-2.023495
H	1.639837	-2.334906	-1.397557
C	3.585086	-1.841664	0.617007
H	4.373858	-2.539066	0.307814
H	3.985924	-1.222880	1.429133
H	2.751761	-2.435250	1.003001
H	-1.239761	1.972253	1.295836
H	-0.898084	3.255144	0.141895

D‘-E‘-TS G781.712621, T-595

C	-3.125649	-0.390122	-1.714620
C	-3.024769	-1.779138	-0.988514
C	-1.659991	-1.991250	-0.393415
C	0.713736	1.908213	0.320966
C	-1.474942	1.123889	-0.694220
C	-2.766381	0.746276	-0.779286
H	-3.809526	-1.837049	-0.221494
H	-3.246434	-2.563634	-1.726781
H	-2.444325	-0.399566	-2.579206
H	-4.147402	-0.278815	-2.106577
C	-3.869716	1.300148	0.086359
H	-4.598724	1.842533	-0.536156
H	-4.429328	0.493429	0.583150
H	-3.511224	1.993315	0.856702
C	-0.830949	2.040246	0.317805
H	-0.798154	0.666637	-1.426150
C	-1.289054	-1.703690	0.872945
H	-0.877938	-2.316690	-1.091330
C	0.161895	-1.797875	1.292265
H	0.755950	-2.295370	0.514422
H	0.251786	-2.414128	2.200885
C	-2.243359	-1.258850	1.958879
H	-1.998363	-1.743437	2.915832

H	-2.192781	-0.172017	2.128148
H	-3.286147	-1.492039	1.723859
C	1.226090	0.464905	0.499872
C	0.797583	-0.413735	1.650059
H	0.086689	0.154229	2.261073
H	1.689507	-0.581141	2.269330
C	1.332748	2.355846	-1.039598
H	0.597187	2.242584	-1.846928
H	1.624260	3.412314	-1.016326
C	2.529154	1.422770	-1.283067
H	2.718029	1.176467	-2.336461
H	3.468320	1.846872	-0.886662
C	2.266588	0.192111	-0.433283
H	1.005207	-0.148500	-0.674835
C	1.325239	2.717061	1.496375
H	2.423074	2.670934	1.497668
H	1.031320	3.769168	1.390449
H	0.963672	2.363127	2.470163
C	3.216152	-0.992632	-0.575977
H	4.126546	-0.519369	-0.981792
C	2.722312	-1.972018	-1.670075
H	2.479640	-1.449608	-2.605070
H	3.509455	-2.704255	-1.886014
H	1.832509	-2.525414	-1.341470
C	3.625773	-1.709867	0.721905
H	4.478518	-2.364924	0.502851
H	3.945699	-0.999465	1.495259
H	2.831127	-2.343605	1.130894
H	-1.220246	1.839964	1.329553
H	-1.052666	3.103732	0.120764

E‘ G781.720285

C	-3.575325	0.061254	-1.474755
C	-3.528785	-1.434036	-1.002742
C	-2.107405	-1.828714	-0.732437
C	0.741154	1.636451	0.327883
C	-1.608229	1.147327	-0.447013
C	-2.947572	0.981619	-0.442164
H	-4.163402	-1.552728	-0.114116
H	-3.961951	-2.059060	-1.797453
H	-3.036176	0.142030	-2.430933
H	-4.622397	0.335451	-1.668082
C	-3.863445	1.555959	0.608207
H	-4.599709	2.231052	0.144765
H	-4.444440	0.758012	1.097196
H	-3.336291	2.119164	1.387223
C	-0.765796	1.839895	0.602710
H	-1.056044	0.698358	-1.279526
C	-1.492371	-1.849810	0.474666
H	-1.482739	-1.989079	-1.619622
C	-0.012191	-2.078442	0.553122
H	0.417892	-2.328493	-0.423172

H	0.213692	-2.906627	1.240378
C	-2.206560	-1.635371	1.790861
H	-1.758782	-2.244560	2.589124
H	-2.151239	-0.583453	2.112788
H	-3.269391	-1.891711	1.733401
C	1.211280	0.217579	0.256751
C	0.802014	-0.837512	1.186643
H	0.219150	-0.431070	2.020435
H	1.722884	-1.286928	1.586538
C	1.240484	2.279653	-0.997876
H	0.463928	2.182330	-1.769789
H	1.441105	3.350655	-0.870954
C	2.486202	1.467138	-1.378800
H	2.680762	1.452427	-2.457326
H	3.387343	1.869992	-0.894409
C	2.205091	0.039316	-0.833281
H	1.569187	-0.482725	-1.593530
C	1.578770	2.208714	1.541378
H	2.658738	2.075213	1.413537
H	1.369814	3.285599	1.585273
H	1.274859	1.748369	2.488332
C	3.483541	-0.844546	-0.636961
H	4.076764	-0.618613	-1.538864
C	3.235440	-2.361320	-0.639500
H	2.635151	-2.676738	-1.504241
H	4.195426	-2.891061	-0.690501
H	2.732269	-2.709873	0.272536
C	4.315969	-0.410235	0.583439
H	5.265759	-0.959280	0.598945
H	4.557330	0.660416	0.562576
H	3.805206	-0.624261	1.533579
H	-1.011177	1.469747	1.611900
H	-0.947097	2.927924	0.633210

**E‘**	G781.719130		
C	-3.439355	-0.025188	-1.713884
C	-3.427813	-1.500378	-1.180512
C	-2.036090	-1.865926	-0.758512
C	0.656243	1.661195	0.466985
C	-1.594967	1.118623	-0.531863
C	-2.926608	0.938932	-0.658213
H	-4.144591	-1.591852	-0.353296
H	-3.776227	-2.161203	-1.987890
H	-2.806528	0.025843	-2.613013
H	-4.463689	0.228887	-2.022221
C	-3.948596	1.537208	0.274294
H	-4.646660	2.178748	-0.285724
H	-4.562163	0.748512	0.738073
H	-3.508254	2.141100	1.076390
C	-0.872582	1.848642	0.580051
H	-0.955260	0.651076	-1.288045
C	-1.541614	-1.841435	0.502873

H	-1.325491	-2.043139	-1.574975
C	-0.073136	-2.047847	0.728183
H	0.447148	-2.319831	-0.196654
H	0.096006	-2.853140	1.457749
C	-2.383215	-1.597839	1.735980
H	-2.006688	-2.173446	2.593696
H	-2.374923	-0.535401	2.026573
H	-3.431611	-1.874691	1.584298
C	1.155355	0.250216	0.477375
C	0.663998	-0.776855	1.398003
H	-0.007761	-0.347359	2.148799
H	1.544827	-1.192993	1.910721
C	1.286236	2.281960	-0.808622
H	0.615831	2.124852	-1.665864
H	1.430847	3.364164	-0.701277
C	2.596207	1.509167	-0.997410
H	2.949977	1.505807	-2.033234
H	3.398730	1.941476	-0.381411
C	2.278713	0.071400	-0.487874
H	1.765538	-0.469643	-1.324071
C	1.354185	2.268622	1.751692
H	2.440710	2.123084	1.758985
H	1.155438	3.348247	1.735798
H	0.934562	1.842282	2.669759
C	3.522491	-0.797228	-0.084237
H	3.847805	-0.438264	0.909267
C	4.673856	-0.556779	-1.082458
H	5.005514	0.486751	-1.109702
H	5.538072	-1.169952	-0.797599
H	4.377981	-0.851405	-2.099829
C	3.248813	-2.309256	-0.001839
H	4.186885	-2.840058	0.203853
H	2.546070	-2.596448	0.787375
H	2.860983	-2.689789	-0.958530
H	-1.217193	1.499917	1.567598
H	-1.068623	2.934468	0.564336

E‘-F‘-TS G781.707808, T-319

C	3.709092	-0.309217	-1.510634
C	3.515094	1.242857	-1.343899
C	2.076988	1.591952	-1.074686
C	-0.508623	-1.494310	0.306971
C	1.805299	-1.399908	-0.420526
C	3.103365	-1.049566	-0.335048
H	4.173066	1.598456	-0.539402
H	3.862403	1.721842	-2.271564
H	3.223647	-0.623751	-2.446512
H	4.783873	-0.518802	-1.612357
C	3.961090	-1.215851	0.892056
H	4.783649	-1.916618	0.680101
H	4.432167	-0.263601	1.176556
H	3.416357	-1.603944	1.761832

C	0.896993	-1.866984	0.710209
H	1.319303	-1.238096	-1.385780
C	1.530073	1.847686	0.132395
H	1.399813	1.520542	-1.934542
C	0.032598	2.015498	0.291980
H	-0.469351	2.016677	-0.684071
H	-0.179516	2.994952	0.750184
C	2.330297	1.978561	1.412355
H	1.917383	2.771813	2.053070
H	2.316592	1.049966	2.005589
H	3.382465	2.216975	1.227058
C	-1.182872	-0.261480	0.528833
C	-0.611832	0.945271	1.243257
H	0.136518	0.625161	1.978345
H	-1.428562	1.404151	1.814632
C	-1.228889	-2.295410	-0.756630
H	-0.628405	-2.183363	-1.674908
H	-1.256538	-3.370598	-0.534006
C	-2.601292	-1.618929	-0.900041
H	-3.002336	-1.693679	-1.915254
H	-3.332778	-2.095362	-0.230051
C	-2.358895	-0.149021	-0.468482
H	-1.908783	0.386032	-1.329353
C	-1.618087	-1.447127	1.852551
H	-2.607335	-0.995883	1.979662
H	-1.774007	-2.522759	1.725184
H	-0.957218	-1.232420	2.690189
C	-3.630240	0.638445	-0.042621
H	-3.898622	0.330990	0.986996
C	-4.821305	0.285262	-0.957870
H	-5.105248	-0.772225	-0.900894
H	-5.699592	0.877645	-0.672878
H	-4.590344	0.521892	-2.006994
C	-3.436618	2.167663	-0.058728
H	-4.376759	2.665789	0.210138
H	-2.671013	2.527331	0.635994
H	-3.161724	2.509082	-1.067502
H	1.186964	-1.382623	1.652655
H	0.940522	-2.957929	0.869238

**F‘**	G781.713593		
C	3.891671	-0.729029	-0.985690
C	3.765040	0.819325	-1.263196
C	2.309517	1.178514	-1.275827
C	-0.490039	-1.579579	0.179426
C	1.828750	-1.826119	-0.175954
C	2.952751	-1.131647	0.132381
H	4.320061	1.375625	-0.497056
H	4.255041	1.017161	-2.228791
H	3.640643	-1.274976	-1.905951
H	4.936398	-0.962275	-0.732056
C	3.324032	-0.647623	1.506011

H	4.351925	-0.958365	1.743310
H	3.316477	0.451106	1.536918
H	2.669991	-1.012034	2.305586
C	0.705131	-2.201928	0.797333
H	1.683932	-2.146567	-1.209585
C	1.626800	1.913329	-0.372640
H	1.712689	0.611771	-2.001254
C	0.119899	1.767942	-0.312512
H	-0.229874	1.309179	-1.246018
H	-0.382846	2.743626	-0.233102
C	2.243186	2.821594	0.665526
H	1.899946	3.855108	0.499483
H	1.928448	2.556648	1.687420
H	3.338240	2.833889	0.637214
C	-1.201351	-0.340099	0.615250
C	-0.297163	0.894672	0.919170
H	0.604622	0.528028	1.428990
H	-0.822853	1.512992	1.656741
C	-1.202148	-2.232790	-0.940886
H	-0.643269	-1.901470	-1.843793
H	-1.140271	-3.330023	-0.933277
C	-2.603290	-1.591863	-0.968424
H	-3.035996	-1.611409	-1.973121
H	-3.282095	-2.147984	-0.303414
C	-2.365063	-0.158879	-0.440036
H	-1.954322	0.435480	-1.275681
C	-1.783675	-0.850242	2.005476
H	-2.492670	-0.088868	2.348387
H	-2.322440	-1.802574	1.929239
H	-0.983896	-0.950279	2.746197
C	-3.664090	0.550272	0.014060
H	-4.108669	-0.035495	0.838085
C	-4.687238	0.563810	-1.140267
H	-4.983632	-0.445842	-1.451266
H	-5.598378	1.091779	-0.831332
H	-4.283345	1.087828	-2.019797
C	-3.430047	1.989486	0.506641
H	-4.389285	2.471558	0.734409
H	-2.819858	2.040878	1.416735
H	-2.933732	2.592171	-0.267580
H	0.910835	-1.806309	1.796472
H	0.582133	-3.295242	0.852835

**F‘**	G781.709261		
C	4.084377	-0.748300	-1.032830
C	3.714499	0.720574	-1.459542
C	2.228625	0.984465	-1.442194
C	-0.350374	-1.125568	0.344447
C	2.072799	-1.540680	0.069440
C	3.324204	-1.075793	0.239202
H	4.246947	1.421093	-0.802967
H	4.108083	0.880869	-2.475076

H	3.795282	-1.438332	-1.839193
H	5.173776	-0.822876	-0.905620
C	3.936284	-0.645888	1.544196
H	4.809788	-1.280094	1.761503
H	4.309611	0.386525	1.491990
H	3.253915	-0.728876	2.398970
C	0.917619	-1.386660	1.053774
H	1.792258	-1.871406	-0.932047
C	1.537593	1.668488	-0.493778
H	1.654209	0.546213	-2.267220
C	0.033154	1.841725	-0.570123
H	-0.365862	1.356097	-1.473101
H	-0.177081	2.918540	-0.688353
C	2.195062	2.350985	0.687297
H	1.695618	3.304361	0.912490
H	2.131109	1.739244	1.600785
H	3.256002	2.560517	0.519618
C	-1.342509	-0.065800	0.656113
C	-0.737879	1.379403	0.698126
H	-0.096945	1.472470	1.585055
H	-1.591621	2.042609	0.876810
C	-0.821206	-1.992601	-0.752016
H	-0.327911	-1.554913	-1.650929
H	-0.467816	-3.031693	-0.697881
C	-2.337165	-1.742244	-0.846148
H	-2.720301	-1.941190	-1.851573
H	-2.869729	-2.408381	-0.149315
C	-2.490916	-0.265291	-0.416160
H	-2.225921	0.366283	-1.284944
C	-1.832142	-0.424894	2.114888
H	-2.591499	0.309250	2.404923
H	-2.282165	-1.422922	2.162503
H	-1.007296	-0.370936	2.833940
C	-3.942066	0.106084	-0.017542
H	-4.222255	-0.496116	0.864666
C	-4.910851	-0.269723	-1.158232
H	-4.920039	-1.347217	-1.364738
H	-5.935613	0.021302	-0.894514
H	-4.647706	0.254272	-2.089670
C	-4.124867	1.594993	0.332256
H	-5.188954	1.817630	0.483053
H	-3.606023	1.893567	1.252199
H	-3.766570	2.239108	-0.484681
H	1.124606	-0.637165	1.825680
H	0.734661	-2.357483	1.570605

F‘-G‘-TS G781.697086, T-1006

C	4.186774	-0.919896	-0.798370
C	3.809421	0.504648	-1.388043
C	2.321009	0.756755	-1.466689
C	-0.326905	-1.008435	0.353695
C	2.102317	-1.613393	0.258006

C	3.363006	-1.116077	0.444951
H	4.313741	1.269461	-0.784131
H	4.255029	0.552916	-2.392960
H	3.950661	-1.689980	-1.546476
H	5.267524	-0.943635	-0.603050
C	3.868943	-0.504654	1.717165
H	4.776355	-1.047903	2.024649
H	4.175264	0.539803	1.563900
H	3.159230	-0.555338	2.550457
C	0.969597	-1.035474	0.942962
H	1.890574	-2.100329	-0.695361
C	1.585230	1.578684	-0.682900
H	1.785718	0.166267	-2.219935
C	0.074059	1.702480	-0.856726
H	-0.271302	1.053109	-1.676391
H	-0.132331	2.732505	-1.192119
C	2.177634	2.483991	0.378284
H	1.721789	3.483891	0.328254
H	1.985778	2.111157	1.397375
H	3.260375	2.609861	0.278992
C	-1.383441	0.046618	0.669258
C	-0.780937	1.475098	0.429952
H	-0.199943	1.764661	1.317516
H	-1.640302	2.155836	0.406083
C	-0.768553	-1.911288	-0.759930
H	-0.357606	-1.475094	-1.691125
H	-0.372687	-2.932797	-0.687832
C	-2.300490	-1.781043	-0.734875
H	-2.750392	-2.059377	-1.693181
H	-2.722997	-2.446286	0.036071
C	-2.538718	-0.300138	-0.364640
H	-2.334209	0.297301	-1.273750
C	-1.828830	-0.080029	2.148835
H	-2.557100	0.696393	2.405062
H	-2.289415	-1.056377	2.348198
H	-0.972005	0.042356	2.826478
C	-3.995783	0.017434	0.057101
H	-4.201366	-0.495611	1.012883
C	-4.987988	-0.535273	-0.986704
H	-4.953221	-1.629375	-1.061125
H	-6.014987	-0.257375	-0.716665
H	-4.784471	-0.118221	-1.984779
C	-4.250843	1.524574	0.247999
H	-5.308609	1.703001	0.480926
H	-3.664454	1.966026	1.063647
H	-4.018550	2.079850	-0.673361
H	1.152706	-0.313684	1.742248
H	0.078193	-1.810506	1.453516

**G'**	G781.724721		
C	4.164503	-0.794350	-0.918824
C	3.753661	0.696564	-1.436159

C	2.283686	0.946312	-1.507937
C	-0.403249	-1.279270	0.568984
C	2.061667	-1.446636	0.112658
C	3.403342	-1.070426	0.314449
H	4.269881	1.408272	-0.781612
H	4.218585	0.754162	-2.429896
H	3.896335	-1.494639	-1.720610
H	5.250937	-0.791744	-0.771899
C	4.009430	-0.746567	1.635308
H	4.811150	-1.479245	1.831957
H	4.507292	0.234117	1.605482
H	3.308751	-0.785798	2.474800
C	1.008037	-1.088674	0.932909
H	1.812571	-1.834929	-0.875805
C	1.551419	1.613681	-0.579511
H	1.750444	0.477795	-2.341423
C	0.043361	1.726334	-0.698812
H	-0.314890	1.137002	-1.554838
H	-0.183011	2.777706	-0.943905
C	2.172114	2.338111	0.598271
H	1.625771	3.267744	0.808584
H	2.125664	1.738016	1.520116
H	3.222441	2.601929	0.435236
C	-1.401546	-0.022371	0.760970
C	-0.753015	1.381033	0.596654
H	-0.128725	1.587942	1.479326
H	-1.585577	2.091862	0.670044
C	-0.743847	-1.912432	-0.782875
H	-0.269667	-1.345921	-1.601189
H	-0.413561	-2.957524	-0.858561
C	-2.264470	-1.738310	-0.841484
H	-2.669880	-1.921248	-1.842585
H	-2.751574	-2.449568	-0.152824
C	-2.490553	-0.287156	-0.365189
H	-2.225696	0.364703	-1.218588
C	-1.954237	-0.105798	2.199890
H	-2.606954	0.739211	2.446311
H	-2.525132	-1.030380	2.358660
H	-1.128411	-0.096024	2.927982
C	-3.968214	0.040190	-0.017073
H	-4.239375	-0.498099	0.907527
C	-4.908969	-0.457631	-1.133871
H	-4.891024	-1.548975	-1.242506
H	-5.944318	-0.165444	-0.913861
H	-4.639774	-0.013556	-2.104562
C	-4.210699	1.545145	0.205726
H	-5.277449	1.736623	0.382241
H	-3.666278	1.950785	1.067180
H	-3.915282	2.123460	-0.683099
H	1.211577	-0.618558	1.900572
H	-0.749354	-1.986183	1.357236

G‘*		G838.296625

G’*–NH_3		G781.733464

C	-4.242259	0.434580	-1.152319
C	-3.946949	-1.152249	-1.078714
C	-2.491999	-1.508409	-1.056783
C	0.373280	1.065734	-0.029407
C	-2.078529	1.317068	-0.467492
C	-3.412705	1.096568	-0.115236
H	-4.481467	-1.539547	-0.203015
H	-4.439683	-1.559859	-1.972804
H	-3.967251	0.777319	-2.158797
H	-5.319869	0.573359	-0.999614
C	-3.971844	1.253412	1.259107
H	-4.763586	2.020513	1.222904
H	-4.467736	0.328514	1.588483
H	-3.237061	1.566370	2.007488
C	-1.005962	1.137248	0.402475
H	-1.850944	1.321977	-1.534725
C	-1.773139	-1.857577	0.038555
H	-1.954059	-1.392562	-2.003616
C	-0.276539	-2.104503	-0.047206
H	0.084172	-1.895319	-1.064582
H	-0.118920	-3.185220	0.108364
C	-2.392776	-2.088774	1.402218
H	-1.926188	-2.955081	1.892188
H	-2.234379	-1.232879	2.076269
H	-3.471192	-2.274333	1.359409
C	1.318762	-0.042662	0.631296
C	0.589208	-1.362934	1.017784
H	-0.013727	-1.181011	1.920823
H	1.379340	-2.052101	1.342575
C	0.705725	1.122182	-1.523388
H	0.171930	0.327733	-2.071643
H	0.435715	2.085388	-1.978539
C	2.212704	0.847561	-1.526358
H	2.594014	0.595051	-2.522144
H	2.755451	1.743805	-1.181985
C	2.388734	-0.304287	-0.513481
H	2.073367	-1.227075	-1.035046
C	1.916312	0.549741	1.924739
H	2.515586	-0.180913	2.479850
H	2.549950	1.419427	1.714587
H	1.109997	0.882984	2.596376
C	3.859351	-0.541021	-0.077922
H	4.176077	0.302328	0.560707
C	4.794368	-0.567915	-1.304411
H	4.818981	0.391939	-1.835128
H	5.821869	-0.799974	-0.993744
H	4.482970	-1.343925	-2.020418
C	4.042658	-1.848225	0.716430

H	5.102431	-2.002065	0.960119
H	3.489268	-1.861649	1.663044
H	3.712467	-2.713613	0.121692
H	-1.200925	1.011535	1.472493
H	0.769149	2.068305	0.387991
N	1.530549	3.825289	0.690339
H	0.995811	4.564028	0.231682
H	1.554576	4.054679	1.684587
H	2.489625	3.911626	0.352474

G‘-H‘-TS* G838.289583, T-1194

G‘-H‘-TS*–NH_3 G781.735422

C	-4.225048	0.451985	-1.141039
C	-3.921799	-1.114464	-1.126734
C	-2.451289	-1.440102	-1.100892
C	0.334138	1.003874	-0.025913
C	-2.089098	1.393256	-0.419151
C	-3.387802	1.082140	-0.070335
H	-4.450200	-1.552792	-0.270630
H	-4.392989	-1.519613	-2.034756
H	-3.958120	0.849404	-2.130513
H	-5.303058	0.593052	-0.983104
C	-3.916949	1.057545	1.330871
H	-4.791372	1.725555	1.388161
H	-4.282559	0.055815	1.599400
H	-3.192983	1.389426	2.082767
C	-0.988954	1.105703	0.425691
H	-1.873343	1.470536	-1.487181
C	-1.731131	-1.878912	-0.042774
H	-1.904138	-1.210756	-2.022391
C	-0.223244	-2.070662	-0.143163
H	0.125374	-1.781956	-1.145760
H	-0.026276	-3.153149	-0.062061
C	-2.342733	-2.267240	1.287748
H	-1.896922	-3.203523	1.654970
H	-2.152775	-1.510960	2.065487
H	-3.426393	-2.415702	1.235323
C	1.332195	-0.019112	0.622156
C	0.632338	-1.374066	0.963109
H	0.020929	-1.232410	1.867586
H	1.429861	-2.065282	1.264657
C	0.727817	1.220592	-1.484502
H	0.230172	0.470704	-2.124275
H	0.440046	2.209898	-1.871598
C	2.243315	0.980183	-1.470385
H	2.647260	0.787969	-2.470398
H	2.774119	1.867411	-1.078146
C	2.427540	-0.215577	-0.511359
H	2.132922	-1.116538	-1.080584
C	1.904542	0.557285	1.937338
H	2.498511	-0.181018	2.487884
H	2.555282	1.424137	1.758342

H	1.086881	0.872929	2.603704
C	3.895863	-0.448494	-0.069282
H	4.198545	0.379238	0.597299
C	4.842463	-0.427771	-1.287122
H	4.861164	0.547613	-1.789450
H	5.869922	-0.657269	-0.974743
H	4.546027	-1.185401	-2.028557
C	4.088434	-1.775582	0.689054
H	5.148427	-1.925062	0.934197
H	3.529262	-1.821438	1.631146
H	3.770146	-2.626872	0.068462
H	-1.185607	0.841481	1.469292
H	0.646913	2.277035	0.492047
N	1.106890	3.549460	0.793304
H	0.430244	4.252683	0.490982
H	1.245850	3.640368	1.800818
H	1.997063	3.734777	0.328590

H‘* G838.298106

H‘*–NH_3 G781.734945

C	-4.205066	0.411734	-1.134405
C	-3.889243	-1.140522	-1.130703
C	-2.407932	-1.443972	-1.104474
C	0.302724	0.863313	-0.089712
C	-2.085622	1.391952	-0.406616
C	-3.359938	1.044988	-0.057847
H	-4.406005	-1.596106	-0.275755
H	-4.350113	-1.564591	-2.036569
H	-3.945717	0.823761	-2.121029
H	-5.284512	0.552278	-0.977188
C	-3.862650	0.940419	1.355036
H	-4.782735	1.537047	1.458100
H	-4.138658	-0.093737	1.607573
H	-3.146118	1.301576	2.102219
C	-0.942538	1.104234	0.431183
H	-1.879524	1.464267	-1.478234
C	-1.679852	-1.929380	-0.073792
H	-1.862487	-1.163508	-2.013489
C	-0.168553	-2.111166	-0.186816
H	0.172795	-1.799813	-1.185783
H	0.036254	-3.193881	-0.128798
C	-2.278554	-2.381140	1.242200
H	-1.840895	-3.341263	1.555143
H	-2.069687	-1.667514	2.055124
H	-3.364654	-2.512378	1.195677
C	1.343526	-0.046586	0.589200
C	0.695687	-1.431422	0.925769
H	0.092099	-1.317268	1.838730
H	1.512595	-2.111322	1.199710
C	0.746572	1.200490	-1.497196
H	0.324247	0.463152	-2.204380
H	0.405763	2.187292	-1.849786

C	2.274871	1.044354	-1.429470
H	2.730928	0.921649	-2.417834
H	2.739768	1.940470	-0.972657
C	2.480262	-0.179004	-0.509572
H	2.231896	-1.070981	-1.115303
C	1.846845	0.600855	1.901223
H	2.514940	-0.062853	2.462626
H	2.407936	1.528487	1.706289
H	0.999449	0.838023	2.562038
C	3.940708	-0.369298	-0.027548
H	4.196717	0.464040	0.651966
C	4.919602	-0.308945	-1.218136
H	4.919515	0.670247	-1.713823
H	5.945350	-0.506244	-0.879319
H	4.668797	-1.070349	-1.972357
C	4.154274	-1.692630	0.731332
H	5.212406	-1.812051	0.999889
H	3.574696	-1.756549	1.660152
H	3.874326	-2.550950	0.102273
H	-1.133468	0.781393	1.459186
H	0.292641	2.623300	0.614749
N	0.610146	3.672533	0.807544
H	-0.164908	4.309395	0.600069
H	0.883337	3.769954	1.789556
H	1.411264	3.897653	0.210498

H‘-I‘-TS* G838.298357, T-1122

H‘-I‘-TS*–NH_3 G781.735196

C	3.886344	0.065839	-1.050679
C	3.308286	1.499618	-1.307382
C	1.801887	1.500489	-1.317581
C	-0.478005	-0.962634	0.174595
C	1.917676	-1.043925	-0.074921
C	3.224649	-0.578290	0.182642
H	3.708201	2.185295	-0.549186
H	3.702115	1.843077	-2.278132
H	3.701330	-0.558372	-1.940757
H	4.977753	0.135771	-0.919745
C	3.521345	0.107555	1.512433
H	4.600969	0.274146	1.631967
H	3.033740	1.090733	1.573883
H	3.172675	-0.487873	2.367010
C	0.770974	-0.861886	0.737082
H	1.756935	-1.477638	-1.067666
C	0.957883	2.136323	-0.470082
H	1.346796	0.859888	-2.083139
C	-0.551065	1.936312	-0.578857
H	-0.780134	1.309078	-1.453113
H	-0.998423	2.920210	-0.797173
C	1.396841	3.129863	0.581798
H	0.895824	4.095866	0.412426
H	1.104495	2.811640	1.594422

H	2.475991	3.314492	0.585648
C	-1.665897	-0.140833	0.673945
C	-1.261341	1.371366	0.698365
H	-0.628767	1.543307	1.580971
H	-2.178368	1.945687	0.876168
C	-0.851794	-1.732603	-1.062701
H	-0.568486	-1.136811	-1.951958
H	-0.340184	-2.701296	-1.145955
C	-2.387777	-1.818093	-0.978937
H	-2.842271	-2.017839	-1.955395
H	-2.685367	-2.635128	-0.300941
C	-2.801964	-0.454612	-0.383518
H	-2.706265	0.294766	-1.193337
C	-2.033667	-0.599611	2.107602
H	-2.851600	0.004631	2.518060
H	-2.345280	-1.651824	2.124845
H	-1.173313	-0.493843	2.781885
C	-4.272916	-0.398186	0.097435
H	-4.383840	-1.083364	0.956092
C	-5.224244	-0.890814	-1.012023
H	-5.054899	-1.943483	-1.271579
H	-6.269288	-0.795682	-0.688336
H	-5.106152	-0.291687	-1.928161
C	-4.704042	1.011190	0.544619
H	-5.770506	1.018863	0.806609
H	-4.153639	1.371625	1.422439
H	-4.558526	1.740243	-0.267175
H	0.883772	-0.301118	1.666727
H	3.942410	-1.809137	0.265014
N	4.808260	-2.823244	0.406525
H	5.573589	-2.736489	-0.264543
H	5.201473	-2.818726	1.349452
H	4.340388	-3.718705	0.253984

I‘* G838.310143

I‘*–NH$_3$ G781.746982

C	3.871975	0.167253	-1.040284
C	3.267738	1.580744	-1.252319
C	1.766069	1.536681	-1.296836
C	-0.499122	-0.940449	0.171911
C	1.902944	-0.988715	-0.074506
C	3.273905	-0.568548	0.201476
H	3.630085	2.255564	-0.466569
H	3.666150	1.968676	-2.204873
H	3.698492	-0.447020	-1.938491
H	4.960718	0.238410	-0.906781
C	3.513416	0.158594	1.527141
H	4.581602	0.379470	1.645281
H	2.969278	1.110424	1.577309
H	3.203089	-0.457450	2.381252
C	0.768986	-0.748692	0.702382
H	1.764349	-1.521978	-1.021148

C	0.884266	2.174276	-0.484834
H	1.346166	0.881748	-2.071205
C	-0.613564	1.922722	-0.622370
H	-0.802936	1.261510	-1.481241
H	-1.083916	2.884514	-0.884706
C	1.275417	3.196014	0.555529
H	0.738364	4.139452	0.371327
H	0.987136	2.878192	1.569902
H	2.346356	3.420069	0.566113
C	-1.699831	-0.145843	0.658226
C	-1.334955	1.376267	0.657362
H	-0.729668	1.590663	1.548945
H	-2.272844	1.925253	0.796904
C	-0.865771	-1.804247	-0.996006
H	-0.587702	-1.258401	-1.919648
H	-0.330311	-2.763091	-1.019427
C	-2.401606	-1.903873	-0.911670
H	-2.847304	-2.151874	-1.880528
H	-2.691566	-2.694625	-0.200972
C	-2.834444	-0.519732	-0.379248
H	-2.750437	0.195796	-1.220642
C	-2.036559	-0.602315	2.104570
H	-2.871554	-0.015104	2.504970
H	-2.317122	-1.662401	2.139055
H	-1.176127	-0.457716	2.770644
C	-4.304407	-0.466906	0.105393
H	-4.403679	-1.132376	0.980786
C	-5.250189	-1.000382	-0.989932
H	-5.066498	-2.056570	-1.223906
H	-6.295435	-0.912857	-0.665645
H	-5.143156	-0.421920	-1.920446
C	-4.753020	0.947032	0.519524
H	-5.820998	0.947287	0.774224
H	-4.214492	1.331727	1.394700
H	-4.611445	1.661020	-0.306175
H	0.873922	-0.144867	1.604703
H	3.874889	-1.543011	0.221637
N	5.293115	-2.886638	0.344470
H	5.748660	-3.086178	-0.546807
H	6.028054	-2.574197	0.980057
H	4.960593	-3.780966	0.707069

**I'**	G781.731107		
C	4.064943	0.098271	0.105432
C	3.755178	0.993145	-1.115040
C	2.267819	1.178992	-1.292556
C	-0.283787	-1.016861	0.019370
C	2.069517	-1.442267	-0.336983
C	3.532672	-1.361759	-0.036057
H	4.261482	1.959110	-0.965475
H	4.192677	0.551068	-2.022138
H	5.150755	0.028677	0.266363

H	3.632216	0.543505	1.013840
C	3.845929	-2.169516	1.255756
H	4.930857	-2.171408	1.419713
H	3.366416	-1.715273	2.132587
H	3.507779	-3.209395	1.174077
C	1.033236	-1.031933	0.488443
H	1.268192	-0.509488	1.417853
H	1.789809	-1.896736	-1.292209
C	1.467277	1.920906	-0.483190
H	1.789589	0.630160	-2.111965
C	-0.046844	1.876002	-0.615695
H	-0.332694	1.285341	-1.499259
H	-0.399705	2.899855	-0.818239
C	2.005030	2.813644	0.609937
H	1.466208	3.772209	0.623181
H	1.860001	2.368278	1.608259
H	3.074031	3.025876	0.502727
C	-1.343323	-0.108004	0.606519
C	-0.794791	1.356412	0.662508
H	-0.146820	1.457763	1.543749
H	-1.658741	2.002157	0.853336
C	-0.817318	-1.774483	-1.153777
H	-0.516380	-1.217839	-2.065229
H	-0.408387	-2.788932	-1.252810
C	-2.347705	-1.680614	-0.993321
H	-2.863985	-1.819376	-1.948480
H	-2.704635	-2.463966	-0.305776
C	-2.569633	-0.281495	-0.378329
H	-2.435763	0.460376	-1.190016
C	-1.657570	-0.608240	2.046828
H	-2.394460	0.052422	2.518541
H	-2.064277	-1.626902	2.042850
H	-0.754447	-0.603205	2.670294
C	-3.993606	-0.068857	0.192687
H	-4.134203	-0.766028	1.037164
C	-5.053169	-0.415288	-0.873416
H	-5.019293	-1.471016	-1.170470
H	-6.060664	-0.215899	-0.485942
H	-4.919284	0.197888	-1.777735
C	-4.234981	1.364187	0.702749
H	-5.280612	1.483719	1.014864
H	-3.611758	1.627206	1.566898
H	-4.043096	2.101550	-0.091608
H	4.065943	-1.825160	-0.880134

I‘-O‘-TS G781.719552, T-407

C	4.393579	0.029877	-0.002954
C	3.866156	0.729056	-1.265557
C	2.337221	0.608627	-1.231837
C	-0.308412	-0.929259	0.084467
C	2.118528	-0.956584	-0.391166
C	3.544561	-1.251265	0.149441

H	4.202798	1.770905	-1.359860
H	4.221567	0.199123	-2.161021
H	5.464611	-0.197433	-0.073993
H	4.267812	0.668517	0.885764
C	3.551418	-1.829468	1.572182
H	4.572547	-2.123180	1.848897
H	3.206207	-1.092680	2.310674
H	2.909461	-2.715912	1.651917
C	0.961260	-0.808764	0.521127
H	1.137025	-0.244817	1.443610
H	1.867578	-1.596542	-1.245021
C	1.542645	1.567645	-0.495223
H	1.858187	0.393064	-2.195922
C	0.076801	1.699744	-0.722288
H	-0.250510	1.106433	-1.583488
H	-0.050140	2.765636	-0.994245
C	2.142282	2.437213	0.552317
H	1.502972	3.289746	0.803274
H	2.255616	1.834417	1.469864
H	3.152294	2.774487	0.289259
C	-1.416558	-0.015903	0.633401
C	-0.871320	1.451453	0.529958
H	-0.332246	1.719997	1.449336
H	-1.707787	2.153557	0.440569
C	-0.807133	-1.700006	-1.118105
H	-0.544686	-1.182967	-2.061342
H	-0.377862	-2.709836	-1.179258
C	-2.337073	-1.675033	-0.932663
H	-2.873658	-1.876938	-1.866046
H	-2.641767	-2.441462	-0.201087
C	-2.616712	-0.265649	-0.368766
H	-2.490563	0.446702	-1.210941
C	-1.752669	-0.347750	2.102432
H	-2.478255	0.353184	2.533045
H	-2.167060	-1.360362	2.184530
H	-0.844993	-0.311851	2.720472
C	-4.051596	-0.055175	0.171576
H	-4.179120	-0.688143	1.066791
C	-5.098796	-0.504861	-0.866538
H	-5.039733	-1.579618	-1.078416
H	-6.112930	-0.295568	-0.501152
H	-4.970973	0.036792	-1.816492
C	-4.324501	1.407019	0.571248
H	-5.360166	1.523302	0.916818
H	-3.673596	1.761032	1.381253
H	-4.190681	2.079066	-0.291050
H	3.969968	-2.004156	-0.535045
**O‘**	G781.761497		
C	-4.217757	0.310534	0.454973
C	-4.056946	-0.952627	-0.419502
C	-2.629219	-0.879020	-1.009582

C	0.224188	0.577666	0.147143
C	-2.199367	0.617138	-0.836323
C	-3.407959	1.393854	-0.291879
H	-4.252133	-1.886960	0.122310
H	-4.774941	-0.910440	-1.251930
H	-5.266266	0.599517	0.601486
H	-3.794551	0.151715	1.462806
C	-3.076703	2.648624	0.519476
H	-3.992511	3.145035	0.864796
H	-2.488998	2.401935	1.419017
H	-2.506006	3.377892	-0.073198
C	-1.143989	0.117802	0.247464
H	-1.515467	0.409269	1.251723
H	-1.716102	1.097528	-1.698604
C	-1.423923	-1.376402	-0.123388
H	-2.583292	-1.273596	-2.034179
C	-0.254655	-1.870380	-1.001004
H	-0.223024	-1.267188	-1.927199
H	-0.430230	-2.909715	-1.314374
C	-1.742502	-2.340419	1.019343
H	-0.850394	-2.579716	1.614441
H	-2.496037	-1.931667	1.704583
H	-2.130160	-3.288072	0.621240
C	1.382614	-0.333183	0.274462
C	1.112419	-1.761544	-0.300840
H	1.192093	-2.509209	0.499298
H	1.905190	-1.995256	-1.021708
C	0.648815	1.960595	-0.151070
H	0.329789	2.102442	-1.207229
H	0.091248	2.729861	0.405621
C	2.186928	1.959197	-0.059156
H	2.636627	2.661647	-0.767851
H	2.509822	2.261796	0.948279
C	2.585085	0.485973	-0.335436
H	2.546483	0.314175	-1.429354
C	1.508296	-0.406573	1.861700
H	2.395765	-1.012302	2.077276
H	1.642030	0.575935	2.328453
H	0.631392	-0.893988	2.298739
C	4.011943	0.133108	0.144572
H	4.063530	0.340872	1.228382
C	5.042869	1.047055	-0.548189
H	4.888457	2.107436	-0.311504
H	6.059118	0.783020	-0.228710
H	4.998093	0.931123	-1.641543
C	4.387277	-1.341984	-0.081093
H	5.420415	-1.520705	0.243610
H	3.746786	-2.039684	0.473635
H	4.329673	-1.606274	-1.147650
H	-3.990877	1.697078	-1.180690

H‘* G838.297419

H‘*–NH_3 G781.734258

C	-3.940948	1.007577	-1.036271
C	-4.005763	-0.570730	-1.017928
C	-2.649305	-1.236148	-1.113199
C	0.518558	0.261932	-0.009071
C	-1.638097	1.389677	-0.339385
C	-2.951047	1.443721	0.019136
H	-4.539359	-0.882545	-0.109793
H	-4.634582	-0.879695	-1.867364
H	-3.608629	1.333331	-2.033950
H	-4.951787	1.407472	-0.868476
C	-3.455155	1.579363	1.429608
H	-2.660903	1.769901	2.161618
H	-4.177030	2.409051	1.489822
H	-4.001154	0.677288	1.743844
C	-0.547848	0.939595	0.522136
H	-1.446129	1.294910	-1.411976
C	-1.940071	-1.807267	-0.112465
H	-2.185204	-1.209620	-2.106620
C	-0.573304	-2.439095	-0.363427
H	-0.242051	-2.237608	-1.391619
H	-0.717120	-3.533935	-0.315671
C	-2.453879	-1.938662	1.304105
H	-1.946863	-1.236007	1.984387
H	-3.529214	-1.752854	1.388309
H	-2.259032	-2.947601	1.698419
C	1.354335	-0.770915	0.724529
C	0.550357	-2.154279	0.678684
H	0.112911	-2.302738	1.676022
H	1.305390	-2.945731	0.567891
C	0.894235	0.253976	-1.479592
H	0.048101	-0.082690	-2.098265
H	1.126188	1.273954	-1.835166
C	2.121630	-0.683844	-1.601818
H	1.813540	-1.646530	-2.030811
H	2.880767	-0.273446	-2.278689
C	2.651702	-0.893357	-0.158927
H	3.047617	-1.915837	-0.053179
C	1.625209	-0.459752	2.207693
H	2.241980	-1.248834	2.659022
H	2.146650	0.495161	2.350762
H	0.687197	-0.418576	2.777946
C	3.832560	0.045847	0.217260
H	4.062523	-0.136102	1.280098
C	5.099344	-0.321299	-0.578368
H	5.358575	-1.380437	-0.446703
H	4.973220	-0.139972	-1.655419
H	5.956169	0.279214	-0.243494
C	3.534815	1.550326	0.072979
H	4.406098	2.147066	0.374723
H	3.303346	1.811579	-0.972353

H	2.690432	1.846060	0.713145
H	-0.747612	0.832326	1.591644
N	0.670443	3.500997	0.096156
H	1.536457	3.573954	-0.445554
H	0.465232	2.449291	0.328657
H	0.757523	4.044602	0.959137
H	-0.130329	3.838620	-0.447526

H‘-H-TS* G838.253994, T-130

H‘-H-TS*–NH_3 G781.690833

C	-3.890669	0.757291	-0.868428
C	-3.870224	-0.817848	-0.773842
C	-2.539090	-1.519061	-1.040550
C	0.477662	0.222399	0.307420
C	-1.823651	0.863943	0.381125
C	-2.905405	1.562355	0.003647
H	-4.245124	-1.091720	0.220653
H	-4.617982	-1.170886	-1.502279
H	-3.726469	1.059214	-1.920015
H	-4.921945	1.070444	-0.638308
C	-3.222587	3.014791	0.255641
H	-2.491282	3.486213	0.929762
H	-3.268320	3.591124	-0.683940
H	-4.210623	3.127241	0.727173
C	-0.500409	0.842439	1.064929
H	-1.896476	-0.139671	-0.004453
C	-1.798016	-2.224289	-0.143978
H	-2.103145	-1.328791	-2.028885
C	-0.355727	-2.643147	-0.421292
H	-0.054555	-2.339268	-1.431733
H	-0.323279	-3.747187	-0.423125
C	-2.312259	-2.538027	1.247113
H	-2.088269	-1.720577	1.953224
H	-3.396760	-2.691822	1.265222
H	-1.842421	-3.447542	1.645614
C	1.477710	-0.816854	0.736396
C	0.703562	-2.235349	0.664083
H	0.228710	-2.363001	1.646239
H	1.516581	-2.976142	0.623750
C	0.551865	0.369441	-1.201682
H	-0.362956	-0.003431	-1.682003
H	0.588533	1.439260	-1.472432
C	1.823678	-0.387422	-1.658478
H	1.551071	-1.291673	-2.216103
H	2.427813	0.220274	-2.343374
C	2.597507	-0.761857	-0.363981
H	3.039057	-1.765585	-0.469378
C	1.984985	-0.700219	2.184994
H	2.673736	-1.522255	2.421695
H	2.514238	0.242859	2.365203
H	1.150882	-0.756395	2.896976
C	3.783309	0.197618	-0.061478

H	4.198754	-0.096226	0.916321
C	4.912561	0.020444	-1.093201
H	5.246187	-1.024990	-1.139701
H	4.594976	0.314856	-2.103852
H	5.780725	0.639782	-0.829661
C	3.384642	1.683212	0.044376
H	4.259127	2.300839	0.288482
H	2.980141	2.052473	-0.912725
H	2.635295	1.839323	0.835321
H	-0.478521	0.711142	2.154381
N	0.345069	3.469451	0.482935
H	1.273010	3.510894	0.051005
H	0.114324	2.391735	0.754051
H	0.330637	4.054476	1.322327
H	-0.367829	3.799699	-0.172836

H* G838.281350

H*–NH_3 G781.718189

C	-3.774861	1.197373	-0.812480
C	-3.972588	-0.353236	-0.682428
C	-2.696213	-1.126986	-0.943258
C	0.354051	0.152985	0.332239
C	-1.941193	1.038448	0.833699
C	-2.438343	1.679466	-0.252236
H	-4.372919	-0.583090	0.313824
H	-4.755129	-0.647295	-1.401033
H	-3.800426	1.475246	-1.878673
H	-4.635309	1.714701	-0.355132
C	-1.748940	2.775892	-1.025317
H	-0.765726	3.045264	-0.615008
H	-1.605473	2.521963	-2.086102
H	-2.374076	3.684113	-1.010926
C	-0.576761	0.637735	1.225384
H	-2.662195	0.439535	1.399671
C	-2.010068	-1.916014	-0.084608
H	-2.256922	-0.964019	-1.935087
C	-0.669579	-2.534601	-0.468061
H	-0.391220	-2.236436	-1.486961
H	-0.809089	-3.629806	-0.509350
C	-2.519443	-2.295857	1.289936
H	-1.961607	-1.789111	2.094019
H	-3.581005	-2.068458	1.431079
H	-2.389686	-3.375508	1.460130
C	1.332741	-0.966804	0.640053
C	0.503537	-2.329639	0.539183
H	0.108308	-2.530442	1.545403
H	1.252378	-3.113465	0.350042
C	0.432348	0.494274	-1.131886
H	-0.549655	0.345532	-1.603895
H	0.653414	1.565680	-1.257542
C	1.565463	-0.369600	-1.732233
H	1.149586	-1.208736	-2.302720

H	2.175059	0.207153	-2.438792
C	2.388416	-0.890857	-0.525196
H	2.751698	-1.910121	-0.731141
C	1.946511	-0.945636	2.053078
H	2.628784	-1.795816	2.187356
H	2.515822	-0.028644	2.250720
H	1.168764	-1.033505	2.823348
C	3.657158	-0.039603	-0.233869
H	4.104640	-0.430601	0.694687
C	4.710880	-0.224758	-1.341752
H	4.965091	-1.285132	-1.473604
H	4.357759	0.157553	-2.310121
H	5.635029	0.315226	-1.094153
C	3.380682	1.460194	-0.012674
H	4.305656	1.988221	0.255496
H	2.981326	1.934307	-0.921290
H	2.658538	1.612500	0.804908
H	-0.515339	0.224763	2.239963
N	0.591675	3.166727	1.780527
H	1.343267	3.461721	1.150076
H	0.263049	2.135124	1.506978
H	0.933639	3.181023	2.745682
H	-0.210543	3.796815	1.689019

Cartesian coordinates of computed structures (Scheme 3 of main text, Table S14)

Gibbs energies (G"..." in Hartree) and imagininary frequencies of TS (T-"..." in cm^{-1}), mPW1PW91/6-311+G(d,p)//B97D3/6-31G(d,p)-sp-density-fitting, 1 bar, 298.15 K. From the structures labelled with asterisks the energy of NH_3 (G56.563161, page 98) is subtracted for direct comparability.

**A’’**	G781.685176		
C	3.078745	1.013924	-0.740625
C	2.933998	1.172853	0.806887
C	1.874711	0.257437	1.369522
C	-1.550796	-1.374974	-1.195866
C	0.915452	0.569574	-2.031487
C	1.862147	1.434126	-1.531817
H	3.920889	0.965965	1.245845
H	2.699381	2.218532	1.051221
H	3.925406	1.635932	-1.071088
H	3.345584	-0.027053	-0.958458
C	1.755350	2.902277	-1.838838
H	1.846113	3.490806	-0.912137
H	2.592549	3.222023	-2.479602
H	0.814761	3.169584	-2.334005
C	0.855467	-0.847448	-1.800046
H	0.475483	-1.476664	-2.605061
H	0.157347	0.995009	-2.693352
C	1.969766	-1.083837	1.497197
H	0.919887	0.724375	1.632116
C	0.741592	-1.899750	1.850605
H	0.030352	-1.291454	2.428922
H	1.010212	-2.767668	2.470948
C	3.226035	-1.876952	1.217833
H	3.499219	-2.471379	2.103108
H	3.077426	-2.604972	0.402304
H	4.088886	-1.255442	0.955546
C	-0.511430	-1.261028	-0.260125
C	0.015053	-2.416341	0.577867
H	0.681871	-3.070333	-0.005571
H	-0.833353	-3.049162	0.882983
C	-2.338700	-0.158027	-1.543381
H	-1.687974	0.727015	-1.533945
H	-2.806727	-0.251451	-2.533028
C	-3.467304	0.098732	-0.465837
H	-4.017682	0.981309	-0.822252
H	-4.173792	-0.742715	-0.491479
C	-2.954390	0.299295	0.937976
H	-0.429368	-0.278308	0.204964
C	-1.953222	-2.682318	-1.797704
H	-2.968324	-2.934866	-1.445582
H	-2.032658	-2.600821	-2.892302
H	-1.286368	-3.509997	-1.537540
C	-2.378007	1.421654	1.423619
H	-3.049672	-0.552627	1.618456

C	-1.909460	1.497398	2.858156
H	-2.045670	0.548755	3.392592
H	-0.845952	1.780815	2.917667
H	-2.461984	2.278261	3.404220
C	-2.146660	2.670378	0.603348
H	-1.066882	2.821682	0.433560
H	-2.641272	2.654742	-0.375075
H	-2.499179	3.561348	1.143910
H	1.651407	-1.313083	-1.221590

A''-C''-TS G781.683893, T-61

C	3.431124	-0.740120	-0.229376
C	3.270860	0.387432	-1.294163
C	1.996790	1.172770	-1.094526
C	-1.200024	-0.581720	1.533184
C	1.339752	-1.998545	0.537847
C	2.400937	-1.841182	-0.323829
H	4.155295	1.036187	-1.217221
H	3.294052	-0.045797	-2.303857
H	4.422414	-1.202582	-0.363256
H	3.428028	-0.281530	0.766608
C	2.640046	-2.873940	-1.390429
H	2.767137	-2.389064	-2.370827
H	3.582220	-3.411208	-1.197179
H	1.829296	-3.607506	-1.464979
C	0.976971	-1.118887	1.607139
H	0.591108	-1.567327	2.522386
H	0.750815	-2.912878	0.436405
C	1.772143	2.079768	-0.119529
H	1.171760	0.947440	-1.778795
C	0.380267	2.644167	0.087653
H	-0.195689	2.606215	-0.848614
H	0.423455	3.697695	0.400546
C	2.815403	2.534156	0.875699
H	2.919386	3.629395	0.838771
H	2.521440	2.294961	1.911784
H	3.805817	2.099486	0.704167
C	-0.622429	0.417320	0.757410
C	-0.384371	1.839046	1.167072
H	0.138726	1.886377	2.136129
H	-1.372131	2.296760	1.353920
C	-1.844185	-1.743829	0.818913
H	-1.287613	-1.968330	-0.100092
H	-1.855051	-2.646089	1.447548
C	-3.307142	-1.347564	0.424936
H	-3.690251	-2.135982	-0.239234
H	-3.939066	-1.363690	1.323548
C	-3.388938	0.016578	-0.224636
H	-0.408926	0.162429	-0.280690
C	-1.607993	-0.328245	2.962501
H	-2.602886	0.144506	2.970233
H	-1.691328	-1.265051	3.527740

H	-0.923439	0.347850	3.487636
C	-3.000661	0.329361	-1.483448
H	-3.789013	0.827831	0.391941
C	-3.110829	1.742962	-2.004093
H	-3.449294	2.447361	-1.233947
H	-2.144546	2.095571	-2.399954
H	-3.818846	1.791336	-2.846385
C	-2.443117	-0.677332	-2.462451
H	-1.373563	-0.482881	-2.654694
H	-2.542315	-1.716961	-2.129396
H	-2.945601	-0.585722	-3.436808
H	1.540982	-0.201982	1.771328

C'' G781.719900

C	-2.954066	-0.942643	-0.518794
C	-3.251440	-0.006269	0.690024
C	-2.077397	0.889051	1.002854
C	1.262231	-0.246902	-1.532876
C	-0.630023	-1.912348	-0.919989
C	-1.836404	-1.939267	-0.289810
H	-4.145502	0.584925	0.444731
H	-3.514429	-0.606676	1.572533
H	-3.869325	-1.516335	-0.742105
H	-2.749465	-0.319754	-1.398181
C	-2.163619	-3.072030	0.658607
H	-2.436619	-2.691951	1.655273
H	-3.037303	-3.636591	0.298017
H	-1.328643	-3.774375	0.777017
C	-0.104230	-0.910617	-1.912496
H	0.051206	-1.422924	-2.878705
H	0.015336	-2.779664	-0.746307
C	-1.701065	1.979253	0.299799
H	-1.441093	0.582916	1.841889
C	-0.369119	2.648638	0.578710
H	-0.062947	2.468313	1.618032
H	-0.449287	3.739680	0.456100
C	-2.487631	2.547219	-0.859648
H	-2.692313	3.615785	-0.692009
H	-1.920150	2.488550	-1.802732
H	-3.445861	2.043272	-1.022387
C	1.051723	0.637724	-0.237709
C	0.718910	2.133001	-0.402243
H	0.338405	2.282192	-1.421913
H	1.624640	2.752323	-0.334730
C	2.356563	-1.279749	-1.158381
H	1.926557	-2.154189	-0.654738
H	2.874650	-1.660493	-2.047977
C	3.306151	-0.541429	-0.209450
H	3.917069	-1.212670	0.409899
H	4.004065	0.093659	-0.771050
C	2.419363	0.389466	0.645859
H	0.184398	0.188152	0.275335

C	1.762616	0.599468	-2.726083
H	2.651611	1.191871	-2.472287
H	2.028572	-0.064161	-3.559099
H	0.994184	1.290364	-3.094162
C	1.802476	-0.140857	1.852510
H	2.880198	1.364038	0.846011
C	1.557552	0.748456	3.014438
H	1.695790	1.810844	2.795019
H	0.588485	0.561571	3.496595
H	2.320258	0.459738	3.765675
C	1.364375	-1.549244	1.962279
H	0.482510	-1.689717	1.294965
H	2.122631	-2.247687	1.584532
H	1.063425	-1.817497	2.980014
H	-0.832857	-0.109037	-2.098861

**C''**	G781.719898		
C	-2.953927	-0.942466	-0.519425
C	-3.251607	-0.006606	0.689737
C	-2.077636	0.888622	1.003138
C	1.262362	-0.246093	-1.532867
C	-0.629815	-1.911882	-0.920674
C	-1.836260	-1.939115	-0.290629
H	-4.145590	0.584691	0.444409
H	-3.514820	-0.607342	1.571942
H	-3.869114	-1.516113	-0.743181
H	-2.749195	-0.319153	-1.398475
C	-2.163473	-3.072191	0.657387
H	-2.436158	-2.692478	1.654283
H	-3.037353	-3.636456	0.296798
H	-1.328596	-3.774736	0.775315
C	-0.104034	-0.909796	-1.912812
H	0.051472	-1.421674	-2.879245
H	0.015636	-2.779189	-0.747242
C	-1.701129	1.979038	0.300504
H	-1.441501	0.582150	1.842171
C	-0.369251	2.648347	0.579872
H	-0.063157	2.467605	1.619146
H	-0.449504	3.739440	0.457765
C	-2.487365	2.547230	-0.859078
H	-2.690958	3.616084	-0.691996
H	-1.920137	2.487473	-1.802258
H	-3.446105	2.044120	-1.021396
C	1.051778	0.637859	-0.237283
C	0.718953	2.133235	-0.401181
H	0.338615	2.282870	-1.420841
H	1.624655	2.752540	-0.333220
C	2.356889	-1.278958	-1.158909
H	1.927017	-2.153776	-0.655794
H	2.875066	-1.659090	-2.048698
C	3.306261	-0.541005	-0.209478
H	3.917225	-1.212512	0.409540

H	4.004139	0.094519	-0.770634
C	2.419275	0.389258	0.646306
H	0.184362	0.188099	0.275453
C	1.762627	0.600862	-2.725720
H	2.651562	1.193253	-2.471681
H	2.028664	-0.062409	-3.558996
H	0.994114	1.291800	-3.093529
C	1.802281	-0.141814	1.852560
H	2.879957	1.363781	0.847065
C	1.556707	0.746859	3.014839
H	1.695294	1.809342	2.796151
H	0.587122	0.559889	3.495976
H	2.318503	0.457438	3.766696
C	1.364450	-1.550354	1.961513
H	0.482802	-1.690596	1.293880
H	2.122961	-2.248460	1.583683
H	1.063177	-1.819137	2.979014
H	-0.832665	-0.108154	-2.098899

C''-D''-TS G781.706221

C	3.524856	-0.708423	-0.294895
C	3.561143	0.733656	-0.880790
C	2.213971	1.408484	-0.800284
C	-0.610502	-1.027034	1.317877
C	1.274651	-1.873630	-0.255732
C	2.424265	-1.562092	-0.894059
H	4.327659	1.307953	-0.339976
H	3.886009	0.696902	-1.930395
H	4.497937	-1.188091	-0.488703
H	3.420642	-0.642324	0.795510
C	2.680751	-2.064837	-2.297338
H	2.865777	-1.229561	-2.991527
H	3.583494	-2.694902	-2.327801
H	1.841188	-2.652345	-2.690741
C	0.868696	-1.479257	1.142817
H	1.016102	-2.335631	1.826550
H	0.587128	-2.538085	-0.790206
C	1.656913	1.970969	0.292600
H	1.611803	1.394217	-1.715945
C	0.248076	2.519278	0.216910
H	-0.026600	2.678563	-0.836061
H	0.203030	3.509900	0.699810
C	2.349484	2.096428	1.630063
H	2.385045	3.150574	1.947002
H	1.807401	1.556897	2.423641
H	3.375650	1.714131	1.619914
C	-0.982841	0.179911	0.361400
C	-0.808952	1.633628	0.920907
H	-0.558046	1.576978	1.986046
H	-1.773665	2.170195	0.890569
C	-1.610595	-2.132736	0.879259
H	-1.273020	-2.633066	-0.038628

H	-1.738558	-2.906453	1.646194
C	-2.928565	-1.396327	0.582373
H	-3.621702	-1.965473	-0.046866
H	-3.448616	-1.149527	1.520195
C	-2.415465	-0.131287	-0.089810
H	-0.334669	0.076295	-0.532113
C	-0.844808	-0.711425	2.812264
H	-1.840800	-0.291023	3.012414
H	-0.748104	-1.632749	3.401402
H	-0.102953	-0.004843	3.205042
C	-3.105543	0.526771	-1.147582
H	-3.164878	0.890249	0.174881
C	-4.494602	0.102593	-1.561115
H	-5.054298	-0.404264	-0.769787
H	-5.071979	0.960928	-1.924760
H	-4.386594	-0.599572	-2.403432
C	-2.466524	1.612726	-1.972631
H	-2.854582	2.604014	-1.699245
H	-1.377405	1.623722	-1.894424
H	-2.753661	1.447748	-3.020160
H	1.514802	-0.675738	1.518196

**D’’**	G781.727285		
C	3.408997	-0.439436	-0.210393
C	3.338441	0.955296	-0.906271
C	1.972071	1.569282	-0.759733
C	-0.663398	-1.307895	1.258273
C	1.374686	-1.969341	-0.172547
C	2.458796	-1.453594	-0.803847
H	4.108573	1.597671	-0.455024
H	3.598919	0.856981	-1.968676
H	4.437332	-0.822465	-0.317239
H	3.229298	-0.306024	0.863643
C	2.797519	-1.918187	-2.203780
H	2.787053	-1.079438	-2.917465
H	3.814920	-2.337552	-2.238666
H	2.099708	-2.680881	-2.570583
C	0.845916	-1.702357	1.212416
H	0.962916	-2.613842	1.824961
H	0.839977	-2.745486	-0.728367
C	1.486537	2.125043	0.376444
H	1.303188	1.511832	-1.625846
C	0.026494	2.500712	0.476091
H	-0.404840	2.642296	-0.526210
H	-0.095260	3.451910	1.017452
C	2.307402	2.317646	1.632537
H	2.154518	3.329801	2.035958
H	2.011694	1.618309	2.430771
H	3.380520	2.179930	1.465943
C	-0.900636	0.054944	0.486057
C	-0.751441	1.410370	1.250300
H	-0.226344	1.193645	2.188218

H	-1.744494	1.779569	1.546347
C	-1.582315	-2.298054	0.479935
H	-1.069623	-2.737322	-0.383969
H	-1.928602	-3.126019	1.109353
C	-2.742063	-1.433923	-0.042715
H	-3.286401	-1.803332	-0.924446
H	-3.527932	-1.304986	0.735147
C	-2.181346	-0.077005	-0.206789
H	-0.102948	0.049389	-0.322964
C	-1.126892	-1.226444	2.728322
H	-2.165507	-0.877339	2.823255
H	-1.066728	-2.220244	3.190362
H	-0.495332	-0.554867	3.323723
C	-2.843180	0.980843	-1.002948
H	-2.498635	1.967019	-0.660405
C	-4.383545	0.928668	-0.990570
H	-4.778264	1.016294	0.029807
H	-4.775949	1.766528	-1.579326
H	-4.767447	0.001720	-1.435559
C	-2.291416	0.794975	-2.464731
H	-2.740690	1.579636	-3.085914
H	-1.200563	0.893801	-2.505811
H	-2.578792	-0.180692	-2.875550
H	1.421984	-0.915093	1.720766

**D’’**	G781.711576		
C	2.404424	-0.259987	-1.646365
C	2.429983	1.274214	-1.347877
C	1.056127	1.819505	-1.072950
C	-0.089672	-0.919461	1.376757
C	2.436812	-1.309969	0.723116
C	2.877764	-1.223007	-0.552724
H	3.120012	1.479079	-0.520753
H	2.839090	1.787705	-2.233511
H	1.383235	-0.545773	-1.960942
H	3.028711	-0.443594	-2.531040
C	3.928376	-2.202338	-1.037194
H	3.562483	-2.775933	-1.904384
H	4.829005	-1.667591	-1.379313
H	4.228937	-2.914683	-0.259332
C	1.421849	-0.477285	1.477761
H	1.709964	-0.503862	2.538283
H	2.888060	-2.109276	1.319271
C	0.637925	2.577929	-0.020135
H	0.309036	1.582760	-1.842863
C	-0.844809	2.701329	0.244808
H	-1.417271	2.657973	-0.693903
H	-1.091867	3.650190	0.741397
C	1.552586	3.174055	1.018534
H	1.209867	2.955351	2.040583
H	2.594156	2.849741	0.927204
H	1.537278	4.271199	0.916945

C	-0.947192	0.136785	0.572576
C	-1.268403	1.526074	1.174643
H	-0.727615	1.607388	2.126588
H	-2.335912	1.602738	1.423734
C	-0.322419	-2.195751	0.522861
H	0.391003	-2.226414	-0.312007
H	-0.185851	-3.118164	1.099280
C	-1.754762	-2.049272	-0.022575
H	-1.993033	-2.599372	-0.944764
H	-2.500255	-2.406490	0.719919
C	-1.996986	-0.589110	-0.107793
H	-0.257605	0.371332	-0.322127
C	-0.676123	-1.096625	2.793863
H	-1.749277	-1.340186	2.779817
H	-0.152157	-1.910242	3.312561
H	-0.551498	-0.189624	3.399947
C	-3.122862	0.011933	-0.867267
H	-3.251480	1.059097	-0.556417
C	-4.453602	-0.750499	-0.672812
H	-4.743632	-0.787962	0.384880
H	-5.246094	-0.232065	-1.225916
H	-4.391187	-1.776677	-1.056751
C	-2.710362	0.020518	-2.376942
H	-3.514553	0.504107	-2.945018
H	-1.783225	0.581357	-2.547267
H	-2.581926	-0.999923	-2.758189
H	1.491677	0.573590	1.182380

D''-E''-TS G781.707253, T-594

C	2.573954	-0.109646	-1.617069
C	2.426173	1.419334	-1.337750
C	1.014219	1.824413	-1.003266
C	-0.068134	-1.047903	1.216823
C	2.503309	-1.213589	0.735489
C	3.035612	-1.056622	-0.498795
H	3.128582	1.720329	-0.550519
H	2.735537	1.951541	-2.252302
H	1.618889	-0.483876	-2.031094
H	3.292873	-0.222770	-2.440119
C	4.188662	-1.945646	-0.919126
H	3.932516	-2.520205	-1.824115
H	5.070404	-1.338338	-1.179064
H	4.480712	-2.654851	-0.135205
C	1.380291	-0.444862	1.390146
H	1.592763	-0.374217	2.466520
H	2.953376	-1.995991	1.354343
C	0.573174	2.513876	0.072953
H	0.259711	1.477573	-1.725978
C	-0.909327	2.542123	0.385197
H	-1.506560	2.466743	-0.535561
H	-1.199226	3.477019	0.885220
C	1.463685	3.167206	1.103276

H	1.210948	2.845151	2.125556
H	2.529077	2.973665	0.944375
H	1.315506	4.258147	1.076595
C	-1.100606	-0.012245	0.747597
C	-1.280964	1.364554	1.348891
H	-0.652550	1.426581	2.245922
H	-2.327093	1.468471	1.672613
C	-0.181433	-2.150735	0.121913
H	0.560939	-1.968482	-0.665549
H	0.024906	-3.147922	0.526276
C	-1.601198	-2.023488	-0.459892
H	-1.690246	-2.335920	-1.506833
H	-2.334468	-2.613542	0.114607
C	-1.965585	-0.571665	-0.228430
H	-0.869500	0.111848	-0.570465
C	-0.601122	-1.575110	2.577240
H	-1.607285	-2.009566	2.490986
H	0.077929	-2.358243	2.938311
H	-0.632120	-0.781700	3.335347
C	-3.202276	0.080175	-0.805672
H	-3.251908	1.117058	-0.442262
C	-4.445931	-0.667675	-0.264266
H	-4.455100	-0.704854	0.833351
H	-5.349732	-0.141477	-0.595108
H	-4.494113	-1.693867	-0.650227
C	-3.177007	0.099321	-2.347846
H	-4.074919	0.604312	-2.723957
H	-2.300183	0.637778	-2.734178
H	-3.167279	-0.917237	-2.762237
H	1.374633	0.575947	1.010273

**E’’**	G781.714289		
C	3.120888	-0.183844	-1.366051
C	2.859695	1.322720	-1.100249
C	1.402957	1.700750	-1.024859
C	-0.177195	-0.922465	0.993378
C	2.356878	-1.331012	0.862398
C	3.127938	-1.234198	-0.251190
H	3.399641	1.635445	-0.196565
H	3.322172	1.869561	-1.939775
H	2.416685	-0.537632	-2.144214
H	4.107400	-0.249377	-1.848920
C	4.124996	-2.340522	-0.530355
H	3.966155	-2.761939	-1.536295
H	5.153264	-1.945584	-0.522428
H	4.065026	-3.158070	0.197627
C	1.287586	-0.411860	1.371216
H	1.350212	-0.349327	2.466204
H	2.516275	-2.228876	1.468443
C	0.810601	2.522180	-0.133273
H	0.759801	1.191365	-1.754813
C	-0.703169	2.556670	-0.040430

H	-1.180128	2.353361	-1.008198
H	-1.065941	3.532584	0.310562
C	1.538281	3.334221	0.911651
H	1.217329	3.068773	1.932510
H	2.626340	3.226525	0.864530
H	1.305749	4.402694	0.783014
C	-1.112349	0.114500	0.510706
C	-1.215394	1.493931	1.025982
H	-0.647830	1.618447	1.955690
H	-2.274903	1.716725	1.223524
C	-0.260256	-2.029349	-0.103270
H	0.476266	-1.813704	-0.889578
H	-0.024271	-3.021425	0.298624
C	-1.684688	-1.909891	-0.663623
H	-1.776663	-2.302713	-1.681647
H	-2.411204	-2.447916	-0.038967
C	-1.957252	-0.386211	-0.601638
H	-1.418304	0.062883	-1.474231
C	-0.874959	-1.436453	2.320244
H	-1.888417	-1.816970	2.151043
H	-0.249494	-2.260312	2.688820
H	-0.910309	-0.648777	3.081241
C	-3.428153	0.116728	-0.688296
H	-3.396327	1.219306	-0.677850
C	-4.266116	-0.357916	0.513567
H	-3.825537	-0.067957	1.478641
H	-5.272555	0.075851	0.463827
H	-4.382747	-1.450146	0.511922
C	-4.063172	-0.320021	-2.018458
H	-5.081569	0.080509	-2.101074
H	-3.486296	0.038932	-2.882061
H	-4.133454	-1.414312	-2.085270
H	1.420926	0.594323	0.980969

**E’’**	G781.709203		
C	3.085341	-0.055761	-1.456563
C	2.753768	1.432084	-1.168885
C	1.283954	1.726047	-1.021157
C	-0.085788	-0.982517	1.029112
C	2.455715	-1.255390	0.786584
C	3.179914	-1.113471	-0.352949
H	3.317233	1.771180	-0.289231
H	3.144731	2.007741	-2.025409
H	2.377702	-0.439583	-2.217254
H	4.059246	-0.065078	-1.968722
C	4.216881	-2.169597	-0.678463
H	4.042182	-2.589295	-1.682479
H	5.225453	-1.727347	-0.702331
H	4.220624	-2.995414	0.042621
C	1.362152	-0.394364	1.345114
H	1.470613	-0.331992	2.436588
H	2.681433	-2.148707	1.378256

C	0.688491	2.511798	-0.099992
H	0.633437	1.172396	-1.711721
C	-0.817725	2.455845	0.053645
H	-1.315310	2.230263	-0.896552
H	-1.225705	3.405952	0.426084
C	1.414354	3.362772	0.914213
H	1.157994	3.072880	1.946770
H	2.503543	3.320566	0.815488
H	1.112428	4.415887	0.805157
C	-1.114675	-0.009140	0.599113
C	-1.235530	1.359050	1.132559
H	-0.614609	1.492046	2.026420
H	-2.287304	1.541784	1.399658
C	-0.151661	-2.088709	-0.063159
H	0.503020	-1.808078	-0.900288
H	0.196494	-3.058374	0.310745
C	-1.617964	-2.087500	-0.503597
H	-1.768436	-2.506616	-1.503719
H	-2.240688	-2.670832	0.190010
C	-2.021924	-0.588707	-0.434435
H	-1.650140	-0.107132	-1.372223
C	-0.691447	-1.535231	2.388027
H	-1.697205	-1.956058	2.276603
H	-0.013694	-2.335673	2.713730
H	-0.714820	-0.754011	3.155798
C	-3.559172	-0.270265	-0.348198
H	-3.854339	-0.389916	0.709752
C	-4.354657	-1.292063	-1.185616
H	-4.223800	-2.322837	-0.838824
H	-5.424798	-1.056695	-1.124872
H	-4.063205	-1.244144	-2.244698
C	-3.930596	1.149007	-0.814058
H	-5.020307	1.273320	-0.780825
H	-3.501952	1.950559	-0.202815
H	-3.613403	1.311384	-1.855119
H	1.426989	0.618495	0.955636

E“-F“-TS G781.704503, T-330

C	3.078101	-0.228694	-1.410854
C	2.730785	1.272831	-1.248161
C	1.261595	1.561522	-1.092508
C	0.044267	-0.784501	0.839681
C	2.562619	-1.188322	0.963508
C	3.265121	-1.133609	-0.190921
H	3.311627	1.694600	-0.416916
H	3.091378	1.779599	-2.160063
H	2.331383	-0.700402	-2.078774
H	4.018017	-0.281173	-1.979372
C	4.383504	-2.137777	-0.394573
H	4.229531	-2.706472	-1.325683
H	5.349907	-1.621229	-0.504957
H	4.468022	-2.852011	0.433139

C	1.389611	-0.355147	1.446090
H	1.340724	-0.456842	2.539820
H	2.875589	-1.961981	1.670046
C	0.679624	2.441437	-0.252157
H	0.603499	0.964500	-1.737899
C	-0.829424	2.440178	-0.089338
H	-1.334317	2.154057	-1.020808
H	-1.201059	3.438391	0.183900
C	1.425491	3.384688	0.663095
H	1.090942	3.287936	1.709098
H	2.512051	3.249664	0.644098
H	1.218579	4.426478	0.372273
C	-1.103637	0.029803	0.632412
C	-1.243466	1.467880	1.063216
H	-0.607614	1.669151	1.935069
H	-2.280989	1.654698	1.368527
C	-0.046898	-2.004401	-0.061761
H	0.587258	-1.778354	-0.933072
H	0.367443	-2.910815	0.396610
C	-1.528630	-2.086168	-0.456621
H	-1.679109	-2.546654	-1.437885
H	-2.088234	-2.692785	0.270926
C	-2.021927	-0.616978	-0.415350
H	-1.697285	-0.128950	-1.357956
C	-1.165764	-1.003041	2.221035
H	-2.255429	-1.047769	2.128629
H	-0.812483	-2.025387	2.384814
H	-0.854088	-0.327417	3.015005
C	-3.559975	-0.411688	-0.288349
H	-3.833859	-0.522383	0.778720
C	-4.326897	-1.489492	-1.081364
H	-4.138541	-2.505092	-0.713839
H	-5.406440	-1.307808	-1.008605
H	-4.058049	-1.455709	-2.147303
C	-4.026191	0.976825	-0.769188
H	-5.116281	1.060158	-0.674678
H	-3.588551	1.811320	-0.211321
H	-3.778002	1.116977	-1.831821
H	1.526931	0.709618	1.242940

F‘‘ G781.724156

C	3.049558	-0.908921	-1.240742
C	2.292193	0.302027	-1.819222
C	0.898946	0.678920	-1.336428
C	0.235756	-0.329887	0.512437
C	2.765522	-0.453346	1.289011
C	3.495675	-0.815244	0.212792
H	2.952331	1.173484	-1.715918
H	2.185275	0.133040	-2.902985
H	2.505959	-1.851770	-1.412756
H	3.954119	-1.007493	-1.858308
C	4.944066	-1.212866	0.417836

H	5.119933	-2.231356	0.036166
H	5.615336	-0.548994	-0.149749
H	5.244188	-1.188883	1.472031
C	1.326227	-0.045409	1.507318
H	0.995039	-0.614223	2.399513
H	3.307725	-0.447631	2.239485
C	0.627683	1.903974	-0.733403
H	0.071077	0.224102	-1.885667
C	-0.784654	2.349433	-0.541354
H	-1.428803	2.034603	-1.370795
H	-0.834118	3.443798	-0.461046
C	1.700739	2.770451	-0.146952
H	1.320951	3.358826	0.698106
H	2.592333	2.213287	0.158473
H	2.011559	3.498422	-0.917225
C	-1.195848	0.189883	0.780319
C	-1.291734	1.727879	0.788501
H	-0.695486	2.126619	1.623947
H	-2.320659	2.054854	0.965224
C	0.104197	-1.738237	-0.035354
H	0.801477	-1.953905	-0.847265
H	0.409466	-2.420927	0.776722
C	-1.369851	-1.927010	-0.458592
H	-1.448111	-2.307831	-1.482902
H	-1.857756	-2.667502	0.191209
C	-2.068279	-0.550058	-0.301338
H	-1.960253	0.000128	-1.254495
C	-1.590553	-0.309391	2.213011
H	-2.637313	-0.052183	2.404620
H	-1.487454	-1.396241	2.318717
H	-0.982901	0.175915	2.984979
C	-3.595147	-0.699164	-0.067914
H	-3.744278	-1.312066	0.838388
C	-4.223856	-1.465758	-1.250458
H	-3.801347	-2.470325	-1.374685
H	-5.304679	-1.580201	-1.097471
H	-4.080049	-0.917415	-2.194268
C	-4.342668	0.633345	0.123458
H	-5.426653	0.460388	0.117269
H	-4.108662	1.126262	1.075455
H	-4.121175	1.337434	-0.693060
H	1.285100	1.004068	1.840738

F‘‘* G838.296079

F‘‘*–NH_3 G781.732918

C	-2.758089	-2.072268	0.403765
C	-1.971544	-1.541602	1.621152
C	-0.725549	-0.674092	1.449504
C	-0.112040	-0.336698	-0.576897
C	-2.707160	-0.055507	-1.175129
C	-3.370369	-1.020649	-0.508262
H	-2.682039	-0.987375	2.251632

H	-1.657537	-2.411918	2.217213
H	-2.156311	-2.776823	-0.187969
H	-3.571356	-2.683861	0.819679
C	-4.872158	-1.133645	-0.667380
H	-5.145502	-2.126130	-1.059987
H	-5.377290	-1.037366	0.307392
H	-5.283456	-0.375245	-1.344695
C	-1.259285	0.363078	-1.267126
H	-0.989183	0.291316	-2.340722
H	-3.325417	0.600622	-1.796542
C	-0.708166	0.702100	1.698786
H	0.213903	-1.187825	1.668180
C	0.610722	1.397125	1.821188
H	1.339366	0.780311	2.362174
H	0.495744	2.348461	2.356930
C	-1.926089	1.560955	1.715283
H	-1.796130	2.385546	0.991468
H	-2.851646	1.028962	1.485102
H	-2.005946	2.035091	2.707756
C	1.232153	0.430578	-0.481026
C	1.131645	1.699841	0.387716
H	0.446823	2.417917	-0.086673
H	2.104260	2.195235	0.467294
C	0.233474	-1.771799	-0.951993
H	-0.389563	-2.517627	-0.453918
H	0.001264	-1.882142	-2.025145
C	1.737639	-1.971250	-0.649937
H	1.904955	-2.839144	-0.002005
H	2.286056	-2.165302	-1.582686
C	2.257418	-0.659680	0.000700
H	2.157540	-0.753612	1.098097
C	1.592403	0.890975	-1.934094
H	2.578035	1.367684	-1.926001
H	1.632317	0.051502	-2.639216
H	0.870889	1.625391	-2.308424
C	3.770955	-0.444583	-0.265380
H	3.925873	-0.416813	-1.358456
C	4.567106	-1.647420	0.282752
H	4.271553	-2.596285	-0.181419
H	5.640282	-1.510231	0.097018
H	4.430029	-1.744893	1.370837
C	4.343030	0.852661	0.335166
H	5.437307	0.857951	0.246454
H	3.980737	1.757108	-0.169367
H	4.102502	0.936791	1.405986
H	-1.072154	4.139481	-1.711784
H	-1.358745	4.759522	-0.240027
H	-1.217616	1.443360	-1.050656
N	-1.542151	3.942618	-0.826056
H	-2.541061	3.983288	-1.037865

F‘‘-H‘‘-TS* G838.275808, T-780

F‘‘-H‘‘-TS*–NH$_3$ G781.712647

C	2.965462	-0.843752	-1.407472
C	2.690322	0.683145	-1.533216
C	1.237179	1.064934	-1.421028
C	-0.023189	-0.738163	0.510588
C	2.357623	-1.445564	1.067593
C	3.138486	-1.520864	-0.042839
H	3.314136	1.235012	-0.813748
H	3.061142	0.979445	-2.530060
H	2.185147	-1.388003	-1.971061
H	3.892118	-1.040949	-1.963958
C	4.342123	-2.442328	-0.005143
H	4.275135	-3.194791	-0.806991
H	5.271008	-1.879633	-0.191283
H	4.441598	-2.972921	0.949340
C	1.134916	-0.630555	1.315296
H	0.854577	-0.560469	2.379815
H	2.710675	-2.006425	1.938780
C	0.677378	2.188300	-0.927137
H	0.544301	0.324178	-1.822320
C	-0.821102	2.221258	-0.679533
H	-1.362002	1.724420	-1.496161
H	-1.179512	3.261333	-0.664465
C	1.436515	3.437948	-0.551854
H	1.154454	3.821926	0.443712
H	2.528177	3.327684	-0.615065
H	1.171674	4.245181	-1.253204
C	-1.335639	0.014152	0.750054
C	-1.197900	1.573300	0.680024
H	-0.464286	1.885783	1.442320
H	-2.157604	1.988282	1.011544
C	-0.211205	-1.825379	-0.501397
H	0.128186	-1.511416	-1.501000
H	0.399610	-2.703592	-0.245341
C	-1.737912	-2.036534	-0.534294
H	-2.058596	-2.494158	-1.475893
H	-2.052957	-2.705952	0.282044
C	-2.312875	-0.620622	-0.321726
H	-2.166437	-0.070654	-1.270689
C	-1.798202	-0.331713	2.201716
H	-2.785481	0.100553	2.395869
H	-1.860842	-1.414649	2.364457
H	-1.101944	0.086361	2.940209
C	-3.835201	-0.610314	-0.035225
H	-4.014449	-1.161138	0.904852
C	-4.589484	-1.356955	-1.155089
H	-4.311162	-2.416429	-1.214420
H	-5.672164	-1.311688	-0.979519
H	-4.393740	-0.897517	-2.136184
C	-4.422865	0.805649	0.113057
H	-5.517707	0.757152	0.178774

H	-4.072630	1.324490	1.013787
H	-4.172441	1.430855	-0.757112
H	2.259854	1.930013	2.808307
H	2.494841	2.470904	1.258328
H	1.621347	0.570350	1.411903
N	2.491989	1.645440	1.855936
H	3.425469	1.232718	1.858628

H‘‘* G838.302969

H‘‘*–NH_3 G781.739808

C	2.833261	-0.894741	-1.457374
C	2.583113	0.633265	-1.655792
C	1.209788	1.062502	-1.210445
C	-0.123653	-0.991351	0.526926
C	2.346213	-1.466851	1.039004
C	3.158233	-1.445468	-0.065575
H	3.394013	1.228183	-1.201615
H	2.672262	0.818372	-2.740068
H	1.960780	-1.430516	-1.863330
H	3.673995	-1.163606	-2.111092
C	4.522338	-2.099779	0.023271
H	4.571516	-2.962805	-0.659799
H	5.317548	-1.410286	-0.302244
H	4.757224	-2.458751	1.032880
C	1.009609	-0.897812	1.256845
H	0.927899	-0.316247	2.187955
H	2.799465	-1.907598	1.937465
C	0.746442	2.205302	-0.652453
H	0.457107	0.298037	-1.387017
C	-0.720887	2.251791	-0.214395
H	-1.365927	2.001103	-1.069094
H	-0.974076	3.289477	0.048413
C	1.543403	3.487059	-0.507899
H	1.497052	3.902712	0.512862
H	2.594989	3.382802	-0.809514
H	1.104974	4.258351	-1.159700
C	-1.394032	-0.166058	0.847344
C	-1.105233	1.367334	1.005872
H	-0.328157	1.480848	1.783592
H	-2.003659	1.817542	1.447065
C	-0.394307	-1.962483	-0.598890
H	0.000766	-1.590658	-1.562491
H	0.084872	-2.935828	-0.426108
C	-1.930875	-2.000781	-0.688354
H	-2.274757	-2.319370	-1.678363
H	-2.346065	-2.708779	0.046749
C	-2.362094	-0.558006	-0.338449
H	-2.095052	0.072149	-1.211063
C	-1.952007	-0.656975	2.210874
H	-2.884441	-0.141886	2.473702
H	-2.149557	-1.735936	2.197770
H	-1.227816	-0.466507	3.014825

C	-3.888638	-0.402727	-0.127525
H	-4.181319	-1.027850	0.734134
C	-4.658299	-0.925754	-1.357901
H	-4.500014	-1.998080	-1.525976
H	-5.737425	-0.768966	-1.228380
H	-4.353418	-0.390131	-2.270450
C	-4.322987	1.046052	0.161576
H	-5.418529	1.118373	0.184708
H	-3.956993	1.416911	1.126397
H	-3.965747	1.730660	-0.622883
H	2.563646	1.543395	2.424285
H	3.619185	2.050026	1.225530
H	2.040928	1.558534	0.799717
N	2.856978	1.408288	1.454427
H	3.129560	0.401185	1.290200

H‘‘* G838.303988

H‘‘*–NH$_3$ G781.740827

C	2.745647	-1.097860	-1.447192
C	2.616835	0.403488	-1.864342
C	1.219296	0.943925	-1.733291
C	-0.192123	-0.643573	0.562062
C	2.232356	-1.117575	1.109603
C	2.999629	-1.445203	0.019395
H	3.354696	1.011935	-1.316005
H	2.923754	0.456243	-2.922120
H	1.846230	-1.631834	-1.792952
H	3.578118	-1.525750	-2.023304
C	4.237316	-2.295467	0.235534
H	4.125054	-3.258773	-0.286482
H	5.133083	-1.823317	-0.200330
H	4.426022	-2.514572	1.294506
C	0.958929	-0.397600	1.225477
H	0.933146	0.368359	2.012849
H	2.635585	-1.466529	2.073944
C	0.756796	1.954559	-0.975860
H	0.473148	0.372781	-2.294208
C	-0.736073	2.171049	-0.808348
H	-1.277466	1.676859	-1.626982
H	-0.963702	3.246436	-0.894508
C	1.638166	2.887958	-0.175460
H	1.561232	2.680495	0.909944
H	2.689526	2.858624	-0.501436
H	1.313856	3.932028	-0.291658
C	-1.493282	0.160056	0.782318
C	-1.291942	1.690035	0.559551
H	-0.643355	2.070354	1.366837
H	-2.259000	2.183706	0.716223
C	-0.442539	-1.788965	-0.385558
H	-0.147010	-1.497831	-1.409397
H	0.134882	-2.688235	-0.129185
C	-1.969853	-1.965047	-0.355155

H	-2.341532	-2.482061	-1.246841
H	-2.274862	-2.559392	0.521790
C	-2.503698	-0.520275	-0.227901
H	-2.360922	-0.039024	-1.215783
C	-1.945112	-0.046712	2.252230
H	-2.875638	0.493515	2.466488
H	-2.107449	-1.108590	2.476507
H	-1.178570	0.323216	2.946613
C	-4.019410	-0.449270	0.086174
H	-4.189401	-0.919557	1.070661
C	-4.822043	-1.257764	-0.954060
H	-4.572283	-2.325859	-0.937514
H	-5.899183	-1.168579	-0.759079
H	-4.638166	-0.881805	-1.972605
C	-4.569856	0.988157	0.140714
H	-5.663854	0.974026	0.237845
H	-4.180419	1.562732	0.989937
H	-4.327942	1.538577	-0.781352
H	3.427445	1.969149	1.072877
H	4.944715	1.259542	0.934275
H	3.530202	0.270423	1.104967
N	4.029797	1.194796	1.389216
H	4.147228	1.235643	2.405633

H‘‘-I-TS* G838.286552, T-1016
H‘‘-I-TS*–NH_3 G781.723391

C	2.687425	-0.957139	-1.384410
C	2.404609	0.489566	-1.888877
C	1.017786	1.031451	-1.652138
C	-0.221963	-0.596644	0.582810
C	2.182981	-0.960152	1.223374
C	3.025969	-1.185943	0.105948
H	3.164381	1.169381	-1.475856
H	2.581775	0.470069	-2.976672
H	1.873352	-1.630450	-1.694587
H	3.569241	-1.308336	-1.940783
C	4.105479	-2.264201	0.309674
H	3.742673	-3.229748	-0.072125
H	5.021974	-2.020732	-0.245078
H	4.361374	-2.405818	1.368250
C	0.891695	-0.388370	1.363826
H	0.750277	0.211332	2.269135
H	2.697677	-1.129079	2.180155
C	0.641936	1.965357	-0.754260
H	0.233617	0.602268	-2.283281
C	-0.824637	2.276617	-0.535232
H	-1.421471	1.930628	-1.390746
H	-0.967033	3.368018	-0.479585
C	1.617177	2.683811	0.156292
H	1.788262	2.121926	1.089956
H	2.589352	2.843254	-0.329641
H	1.230974	3.669776	0.446519

C	-1.551516	0.124550	0.830974
C	-1.381838	1.674507	0.778427
H	-0.736130	1.980137	1.617965
H	-2.359223	2.128851	0.982128
C	-0.404171	-1.702892	-0.421580
H	-0.067027	-1.358730	-1.412027
H	0.181574	-2.597447	-0.173715
C	-1.929649	-1.911366	-0.475519
H	-2.239509	-2.368738	-1.421123
H	-2.261652	-2.577123	0.337109
C	-2.501984	-0.491716	-0.272326
H	-2.323111	0.065297	-1.212166
C	-2.043417	-0.250381	2.260630
H	-3.011544	0.220789	2.465871
H	-2.158718	-1.334611	2.380708
H	-1.335594	0.099226	3.022792
C	-4.031097	-0.472891	-0.025024
H	-4.237220	-1.033731	0.903492
C	-4.764442	-1.198036	-1.172450
H	-4.492009	-2.258652	-1.240077
H	-5.850954	-1.146860	-1.022776
H	-4.541606	-0.726943	-2.142337
C	-4.611032	0.945302	0.130330
H	-5.707750	0.905251	0.168263
H	-4.277644	1.445700	1.047580
H	-4.333844	1.581828	-0.723587
H	4.378907	1.783137	0.831966
H	5.407335	0.980042	-0.185539
H	3.779934	-0.109219	0.438222
N	4.814739	0.880015	0.639693
H	5.410326	0.630621	1.430930

I* G838.296228

I*–NH$_3$ G781.733067

C	-2.570295	-1.311494	1.466594
C	-2.262622	0.092837	2.051535
C	-0.887321	0.658249	1.787759
C	0.121599	-0.468432	-0.543152
C	-2.353932	-0.588247	-1.083376
C	-3.063626	-1.350597	0.001786
H	-3.040590	0.791295	1.713834
H	-2.369616	0.021518	3.146711
H	-1.737309	-2.007844	1.633722
H	-3.400403	-1.731335	2.054522
C	-3.299306	-2.807217	-0.484874
H	-2.356834	-3.368937	-0.527435
H	-3.972835	-3.321619	0.213538
H	-3.757272	-2.833756	-1.482242
C	-1.063222	-0.152295	-1.259725
H	-0.934331	0.584311	-2.057810
H	-3.048915	-0.252035	-1.857126
C	-0.613154	1.778557	1.060630

H	-0.053636	0.174168	2.305404
C	0.805534	2.230919	0.835301
H	1.479428	1.825946	1.601934
H	0.864100	3.328193	0.902174
C	-1.702090	2.575225	0.382365
H	-2.435128	1.940213	-0.136620
H	-2.262530	3.156574	1.131723
H	-1.289098	3.288928	-0.340982
C	1.424513	0.284041	-0.778466
C	1.287691	1.817114	-0.575253
H	0.585156	2.213370	-1.325684
H	2.251324	2.291025	-0.790929
C	0.405277	-1.786290	0.128325
H	0.032258	-1.766824	1.158796
H	-0.125452	-2.608304	-0.369834
C	1.941160	-1.912893	0.150682
H	2.277782	-2.490397	1.017595
H	2.302397	-2.435051	-0.748422
C	2.452647	-0.456401	0.162530
H	2.307166	-0.060581	1.185694
C	1.796549	0.039172	-2.284730
H	2.772054	0.495684	-2.485595
H	1.861300	-1.027024	-2.529751
H	1.062459	0.501326	-2.954119
C	3.967298	-0.350478	-0.151222
H	4.142051	-0.796651	-1.146103
C	4.770889	-1.180803	0.871696
H	4.519431	-2.248076	0.838164
H	5.846491	-1.091054	0.672215
H	4.593269	-0.820840	1.896779
C	4.507652	1.091483	-0.169781
H	5.603705	1.082248	-0.230934
H	4.148189	1.673051	-1.027651
H	4.234838	1.631950	0.748950
H	-4.722681	2.270791	-1.764810
H	-5.285748	1.895493	-0.291743
H	-4.055276	-0.863733	0.012629
N	-4.905114	1.479814	-1.144041
H	-5.691393	0.984575	-1.570052

For intermediates and transition states from **I** to **M** cf. Table S6.

References

[1] R. D. Gietz, R. H. Schiestl, *Nat. Protoc.* **2007**, *2*, 38-41.
[2] B. Xu, C. Liu, M. Dai, *J. Am. Chem. Soc.* **2022**, *144*, 19700-19703.
[3] G. R. Fulmer, A. J. M. Miller, N. H. Sherden, H. E. Gottlieb, A. Nudelman, B. M. Stoltz, J. E. Bercaw and K. I. Goldberg, *Organometallics* **2010**, *29*, 2176-2179.
[4] L. J. Bourhis, O. V. Dolomanov, R. J. Gildea, J. A. K. Howard, H. Puschmann, *Acta Crystallogr. A* **2015**, *71*, 59-75.
[5] G. M. Sheldrick, *Acta Crystallogr. A* **2008**, *64*, 112-122.
[6] A. V. Lübben, G. M. Sheldrick, *J. Appl. Crystallogr.* **2019**, *52*, 669-673.
[7] M. M. Bradford, *Anal. Biochem.* **1976**, *72*, 248-254.
[8] L. Lauterbach, J. Rinkel, J. S. Dickschat, *Angew. Chem. Int. Ed.* **2018**, *57*, 8280-8283.
[9] P. Rabe, J. Rinkel, E. Dolja, T. Schmitz, B. Nubbemeyer, T. H. Luu, J. S. Dickschat, *Angew. Chem. Int. Ed.* **2017**, *56*, 2776-2779.
[10] J. Rinkel, J. S. Dickschat, *Org. Lett.* **2019**, *21*, 2426-2429.
[11] F. M. Hahn, A. P. Hurlburt, C. D. Poulter, *J. Bacteriol.* **1999**, *181*, 4499–4504.
[12] J. Rinkel, P. Rabe, X. Chen, T. G. Köllner, F. Chen, J. S. Dickschat, *Chem. Eur. J.* **2017**, *23*, 10501-10505.
[13] Z. Quan, J. S. Dickschat, *Org. Biomol. Chem.* **2020**, *18*, 6072-6076.
[14]P. Rabe, J. Rinkel, E. Dolja, T. Schmitz, B. Nubbemeyer, T. H. Luu, J. S. Dickschat, *Angew. Chem. Int. Ed.* **2017**, *56*, 2776-2779.
[15] P. Rabe, L. Barra, J. Rinkel, R. Riclea, C. A. Citron, T. A. Klapschinski, A. Janusko, J. S. Dickschat, *Angew. Chem. Int. Ed.* **2015**, *54*, 13448-13451.
[16] T. Lou, A. Li, H. Xu, J. Pan, B. Xing, R. Wu, J. S. Dickschat, D. Yang, M. Ma, *J. Am. Chem. Soc.* **2023**, *145*, 8474-8485.
[17] G. B. Tabekoueng, H. Li, B. Goldfuss, G. Schnakenburg, J. S. Dickschat, *Angew. Chem. Int. Ed.* **2024**, *63*, e202413860.
[18] G. Bian, J. Rinkel, Z. Wang, L. Lauterbach, A. Hou, Y. Yuan, Z. Deng, T. Liu, J. S. Dickschat, *Angew. Chem. Int. Ed.* **2018**, *57*, 15887-15890.
[19] Z. Quan, J. S. Dickschat, *Org. Lett.* **2020**, *22*, 7552-7555.
[20] J. Rinkel, J. S. Dickschat, *Beilstein J. Org. Chem.* **2019**, *15*, 1008-1019.
[21] P. Rabe, J. Rinkel, B. Nubbemeyer, T. G. Köllner, F. Chen, J. S. Dickschat, *Angew. Chem. Int. Ed.* **2016**, *55*, 15420-15423.
[22] T. A. Klapschinski, P. Rabe, J. S. Dickschat, *Angew. Chem. Int. Ed.* **2016**, *55*, 10141-10144.
[23] B. K. Chhetri, S. Lavoie, A. M. Sweeney-Jones, N. Mojib, V. Raghavan, K. Gagaring, B. Dale, C. W. McNamara, K. Soapi, C. L. Quave, P. L. Polavarapu, J. Kubanek, *J. Org. Chem.* **2019**, *84*, 8531-8541.
[24] H. Xu, L. Lauterbach, B. Goldfuss, G. Schnakenburg, J. S. Dickschat, *Nat. Chem.* **2023**, *15*, 1164-1171.
[25] S. S. Shinde, A. Minami, Z. Chen, T. Tokiwano, T. Toyomasu, N. Kato, T. Sassa, H. Oikawa, *J. Antibiot.* **2017**, *70*, 632-638.
[26] S. Grimme, S. Ehrlich, L. Goerigk, *J. Comp. Chem.* **2011**, *32*, 1456-1465.
[27] Gaussian 16, Revision C.01, M. J. Frisch, G. W. Trucks, H. B. Schlegel, G. E. Scuseria, M. A. Robb, J. R. Cheeseman, G. Scalmani, V. Barone, G. A. Petersson, H. Nakatsuji, X. Li, M. Caricato, A. V. Marenich, J. Bloino, B. G. Janesko, R. Gomperts, B. Mennucci, H. P. Hratchian, J. V. Ortiz, A. F. Izmaylov, J. L. Sonnenberg, D. Williams-Young, F. Ding, F. Lipparini, F. Egidi, J. Goings, B. Peng, A. Petrone, T. Henderson, D. Ranasinghe, V. G. Zakrzewski, J. Gao, N. Rega, G. Zheng, W. Liang, M. Hada, M. Ehara, K. Toyota, R. Fukuda, J. Hasegawa, M. Ishida, T. Nakajima, Y. Honda, O. Kitao, H. Nakai, T. Vreven, K. Throssell, J. A. Montgomery, Jr., J. E. Peralta, F. Ogliaro, M. J. Bearpark, J. J. Heyd, E. N. Brothers, K. N. Kudin, V. N. Staroverov, T. A. Keith, R. Kobayashi, J. Normand, K. Raghavachari, A. P. Rendell, J. C. Burant, S. S. Iyengar, J. Tomasi, M. Cossi, J. M. Millam,

M. Klene, C. Adamo, R. Cammi, J. W. Ochterski, R. L. Martin, K. Morokuma, O. Farkas, J. B. Foresman, and D. J. Fox, Gaussian, Inc., Wallingford CT, 2019.
[28] S. Grimme, *Chem. Eur. J.* **2012**, *18*, 9955-9964.
[29] GoodVibes v3.0.1, G. Luchini, J. V. Alegre-Requena, Y. Guan, I. Funes-Ardoiz, R. S. Paton, 2019.
[30] C. Adamo, V. Barone, *J. Chem. Phys.* **1998**, *108*, 664.
[31] S. P. T. Matsuda, W. K. Wilson, Q. Xiong, *Org. Biomol. Chem.* **2006**, *4*, 530-543.
[32] Y. J. Hong, D. J. Tantillo, *J. Org. Chem.* **2018**, *83*, 3780-3793.
[33] L. Lauterbach, B. Goldfuss, J. S. Dickschat, *Angew. Chem.* **2020**, *132*, 12041-12045; *Angew. Chem. Int. Ed.* **2020**, *59*, 11943-11947.
[34] H. Xu, B. Goldfuss, J. S. Dickschat, *Chem. Eur. J.* **2021**, *27*, 9758-9762.
[35] P. Pracht, F. Bohle, S. Grimme, *Phys. Chem. Chem. Phys.* **2020**, *22*, 7169-7192.
[36] S. Grimme, *J. Chem. Theory Comput.* **2019**, *155*, 2847-2862.
[37] P. Pracht, S. Grimme, *Chem. Sci.* **2021**, *12*, 6551-6568.
[38] P. Pracht, C.A. Bauer, S. Grimme, *J. Comput. Chem.* **2017**, *38*, 2618-2631.
[39] S. Spicher, C. Plett, P. Pracht, A. Hansen, S. Grimme, *J. Chem. Theory Comput.* **2022**, *18*, 3174-3189.
[40] J. Jumper, R. Evans, A. Pritzel, T. Green, M. Figurnov, O. Ronneberger, K. Tunyasuvunakool, R. Bates, A. Žídek, A. Potapenko, A. Bridgland, C. Meyer, S. A. A. Kohl, A. J. Ballard, A. Cowie, B. Romera-Paredes, S. Nikolov, R. Jain, J. Adler, T. Back, S. Petersen, D. Reiman, E. Clancy, M. Zielinski, M. Steinegger, M. Pacholska, T. Berghammer, S. Bodenstein, D. Silver, O. Vinyals, A. W. Senior, K. Kavukcuoglu, P. Kohli, D. Hassabis, *Nature* **2021**, *596*, 583-589.
[41] O. Trott, A. J. Olson, *J. Comput. Chem.* **2010**, *31*, 455-461.
